# Kinetic enantio-recognition of chiral viologen guests by planar-chiral porphyrin cages†

**DOI:** 10.1039/d3cc04934e

**Published:** 2023-11-02

**Authors:** Pieter J. Gilissen, Quentin Duez, Guilherme L. Tripodi, Magda M. J. Dekker, Jiangkun Ouyang, Kais Dhbaibi, Nicolas Vanthuyne, Jeanne Crassous, Jana Roithová, Johannes A. A. W. Elemans, Roeland J. M. Nolte

**Affiliations:** a Radboud University, Institute for Molecules and Materials Heyendaalseweg 135 6525 AJ Nijmegen The Netherlands R.Nolte@science.ru.nl J.Elemans@science.ru.nl J.Roithova@science.ru.nl; b Univ Rennes, CNRS, Institut des Sciences Chimiques de Rennes ISCR-UMR 6226 F-35000 Rennes France; c Aix-Marseille University, CNRS, Centrale Marseille, iSm2 13397 Marseille Cedex 20 France

## Abstract

The kinetic enantio-recognition of chiral viologen guests by planar-chiral porphyrin cage compounds, measured in terms of ΔΔ*G*^‡^_on_, is determined by the planar-chirality of the host and influenced by the size, as measured by ion mobility-mass spectrometry, but not the chirality of its substituents.

## Abstract 2

The enantio-kinetic threading of chiral guests by chiral porphyrin hosts is determined by the planar chirality of the host and influenced by the size but not the chirality of the substituent X.

## Graphical abstract

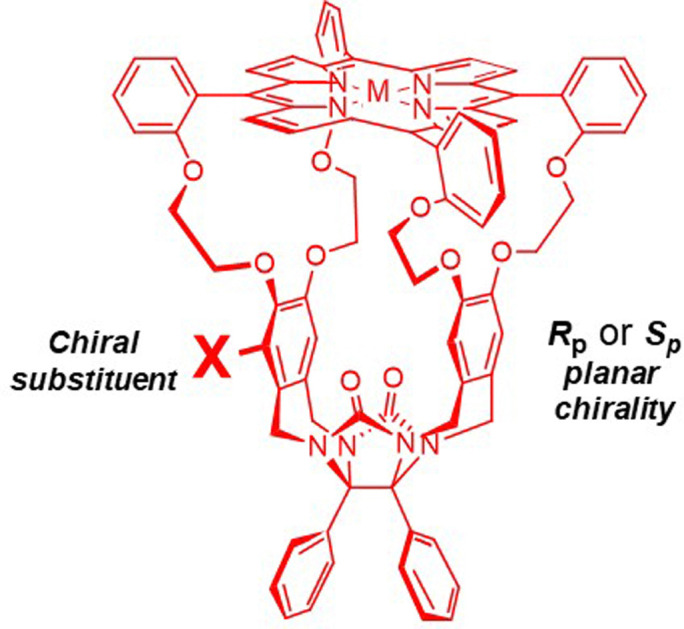

Chiral recognition and substrate selection are fundamental characteristics observed in enzymes and natural receptor molecules.^1^ Inspired by nature's mechanisms, researchers have developed various chiral receptors (hosts) capable of enantio-recognition of chiral guest molecules, predominantly relying on thermodynamic host–guest binding interactions.^2^ However, achieving kinetic enantio-recognition, specifically differences in host–guest threading rates, has proven to be more challenging with only a very limited number of reported examples.^3^ Our research aims to design chiral porphyrin cage catalysts capable of threading onto chiral polymer chains while encoding digital information in the form of chemical functions, such as (*R*,*R*)-epoxide representing the digit 0 and (*S*,*S*)-epoxide representing the digit 1, if the polymer chain contains alkene double bonds.^4^ This process involves selective binding and threading of a polymer chain through the catalytic porphyrin machine, requiring the alignment of chiral structural information in both the catalyst and the polymer. To identify critical chiral features for successful enantio-recognition, we have synthesized a series of chiral porphyrin cages incorporating different chiral groups. These compounds were screened to assess their ability to selectively thread onto the enantiomers of a chiral viologen guest, which strongly binds in the cavity of the porphyrin cage, representing an initial step towards threading onto chiral polymers. Our findings reveal that kinetic enantio-recognition is influenced by the porphyrin cage's planar chirality and the substituent's size on the cage, estimated using ion mobility-mass spectrometry (IMS-MS).^5^

The chemical structures of the compounds of interest, including the chiral viologen guest, are depicted in Fig. 1. Details of their syntheses can be found in previously published papers^3*b*,6^ and the ESI.† The porphyrin macrocycles and the guest form 1 : 1 host–guest complexes (see ESI,† Fig. S151), in line with previous studies.^4*d*^ The stereoselective threading of porphyrin cages 2–6, encompassing both free base and zinc derivatives, onto the enantiomers of guest 7 was quantified using a time-resolved approach-to-equilibrium fluorescence quenching method.^3*b*,4*b*,7^ In such an experiment, the host and guest are mixed in equimolar amounts, and the fluorescence intensity of the host is followed as a function of time until it is quenched when the viologen moiety binds inside the cavity of the porphyrin macrocycle. The initial *circa* 50% of the threading process can be approached by conventional second-order kinetics, and the linear fit slopes equal the threading *k*_on_-values (see ESI†).^3*b*,4*b*^

**Fig. 1:**
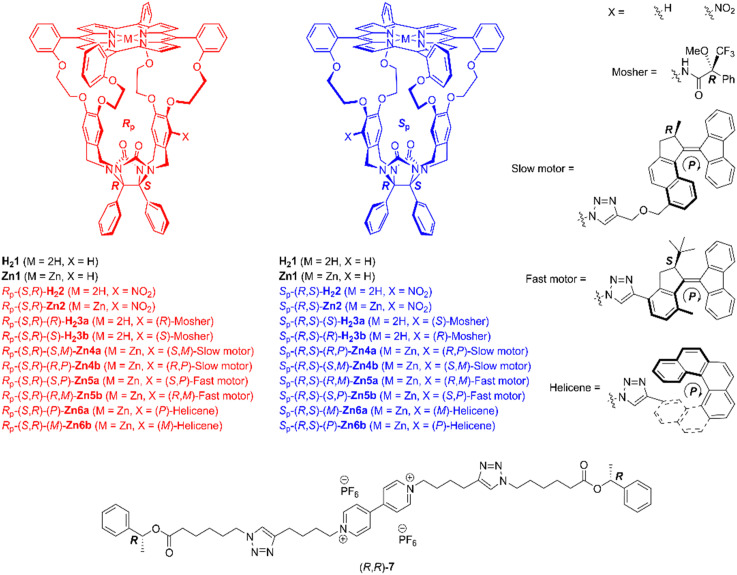
Chemical structures of chiral host and guest molecules. Only the (*R*)-isomer of the Mosher substituent and the (*P*)-isomers of the motor and helicene substituents are shown.

The results of threading experiments with all combinations of the chiral porphyrin hosts and the two enantiomeric guests are presented in Table 1. Porphyrin cages H_2_1 and Zn1 were tested as negative control compounds, as these should not display any kinetic preference for either of the enantiomers of guest 7, which was the case within the experimental error (∼10%). The results for the nitro-functionalized porphyrin cage H_2_2 revealed that the small nitro group could not induce any kinetic selectivity. The two diastereomeric Mosher's amide substituted cages H_2_3a and H_2_3b exhibited a small selectivity (factor 1.2–1.5), with the *R*_p_-enantiomers favoring threading onto (*S*,*S*)-7 over (*R*,*R*)-7 and the *S*_p_-enantiomers showing the opposite selectivity. The zinc(ii) derivative Zn2, displayed a small, yet opposite preference compared to the Mosher's amide substituted cages. *R*_p_-Zn2 and *S*_p_-Zn2 had small preferences (factor 1.3) for (*R*,*R*)-7 and (*S*,*S*)-7, respectively. The porphyrin cages with larger substituents, *i.e.*, molecular motor- and helicene-functionalized compounds Zn4–Zn6 do not only display planar chirality in the porphyrin cage framework but also helical chirality in the substituent. All investigated stereoisomers of Zn4–Zn6 with the *R*_p_-configuration favored threading onto (*S*,*S*)-7, whereas all stereoisomers of Zn4–Zn6 with the *S*_p_-configuration favored threading onto (*R*,*R*)-7. The extent of kinetic preference differed between the substituents (factor 2.9–3.3 for Zn4, 2.0–2.5 for Zn5, and 1.5–1.8 for Zn6), but was not dependent on the chirality present in the substituent. For example, *R*_p_-Zn4a (with (*S*,*M*)-motor substituent) and *R*_p_-Zn4b (with (*R*,*P*)-motor substituent) both displayed a factor ∼3 kinetic preference for threading onto guest (*S*,*S*)-7 over guest (*R*,*R*)-7. Interestingly, the threading rates of the achiral and chiral free base porphyrin cages were higher than those of the achiral and chiral zinc(ii) porphyrin cages. This deceleration in the case of the zinc(ii) porphyrin cages is attributed to the axial coordination of an acetonitrile solvent molecule to the zinc(ii) center on the inside of the cage, as reported before.^8^ This solvent molecule has to dissociate before a guest molecule can thread through the cavity.

**Table 1:** Kinetic and thermodynamic data for the threading of porphyrin macrocyclic hosts onto the enantiomers of viologen guest 7. The data were acquired using fluorescence spectroscopy in CHCl_3_/CH_3_CN (1 : 1, v/v) at 298 K

Host	Δ*ε*_420_ (M^−1^ cm^−1^)	(*R*,*R*)-7		(*S*,*S*)-7		(*R*,*R*)-7 : (*S*,*S*)-7	
		*k*_on_ a (M^−1^ s^−1^)	Δ*G*^‡^_on_ (kJ mol^−1^)	*k*_on_ a (M^−1^ s^−1^)	Δ*G*^‡^_on_ (kJ mol^−1^)	*k*_on_ : *k*_on_	ΔΔ*G*^‡^_on_ (kJ mol^−1^)
H_2_1^b^	0^c^	4.5 × 10^3^	52.1 ± 0.1	5.1 × 10^3^	51.8 ± 0.1	1.0 : 1.1	+0.3 ± 0.1
*R*_p_-H_2_2	−30	3.8 × 10^3^	52.6 ± 0.1	3.8 × 10^3^	52.6 ± 0.1	1.0 : 1.0	+0.1 ± 0.1
*S*_p_-H_2_2	+28	3.7 × 10^3^	52.6 ± 0.1	3.4 × 10^3^	52.8 ± 0.1	1.1 : 1.0	−0.2 ± 0.1
*R*_p_-H_2_3a	−22	2.0 × 10^3^	54.1 ± 0.1	3.0 × 10^3^	53.2 ± 0.1	1.0 : 1.5	+1.0 ± 0.1
*S*_p_-H_2_3a	+35	3.6 × 10^3^	52.7 ± 0.1	2.3 × 10^3^	53.8 ± 0.1	1.5 : 1.0	−1.1 ± 0.1
*R*_p_-H_2_3b	−28	1.4 × 10^3^	55.0 ± 0.2	1.9 × 10^3^	54.3 ± 0.1	1.0 : 1.3	+0.7 ± 0.3
*S*_p_-H_2_3b	+23	1.9 × 10^3^	54.3 ± 0.1	1.5 × 10^3^	54.8 ± 0.2	1.2 : 1.0	−0.5 ± 0.3
Zn1	0^c^	4.1 × 10^2^	58.1 ± 0.1	4.2 × 10^2^	58.1 ± 0.1	1.0 : 1.0	+0.1 ± 0.2
*R*_p_-Zn2	−26	1.9 × 10^3^	54.3 ± 0.1	1.5 × 10^3^	54.9 ± 0.1	1.3 : 1.0	−0.6 ± 0.2
*S*_p_-Zn2	+30	1.4 × 10^3^	55.1 ± 0.1	1.8 × 10^3^	54.4 ± 0.2	1.0 : 1.3	+0.7 ± 0.3
*R*_p_-Zn4a	−75	3.6 × 10^2^	58.4 ± 0.1	1.0 × 10^3^	55.8 ± 0.1	1.0 : 2.9	+2.7 ± 0.2
*S*_p_-Zn4a	+86	1.0 × 10^3^	55.8 ± 0.1	3.2 × 10^2^	58.7 ± 0.1	3.3 : 1.0	−2.9 ± 0.1
*R*_p_-Zn4b	−74	2.4 × 10^2^	59.4 ± 0.1	7.2 × 10^2^	56.7 ± 0.1	1.0 : 3.0	+2.7 ± 0.1
*S*_p_-Zn4b	+75	7.4 × 10^2^	56.6 ± 0.1	2.3 × 10^2^	59.5 ± 0.1	3.2 : 1.0	−2.9 ± 0.1
*R*_p_-Zn5a	−122	4.4 × 10^2^	57.9 ± 0.1	9.8 × 10^2^	55.9 ± 0.1	1.0 : 2.2	+2.0 ± 0.1
*S*_p_-Zn5a	+136	1.0 × 10^3^	55.8 ± 0.1	4.2 × 10^2^	58.0 ± 0.1	2.5 : 1.0	−2.3 ± 0.2
*R*_p_-Zn5b	−103	3.5 × 10^2^	58.5 ± 0.1	6.8 × 10^2^	56.8 ± 0.1	1.0 : 2.0	+1.7 ± 0.2
*S*_p_-Zn5b	+113	6.5 × 10^2^	57.0 ± 0.1	3.3 × 10^2^	58.7 ± 0.1	2.0 : 1.0	−1.7 ± 0.2
*R*_p_-Zn6a	−58	5.6 × 10^2^	57.3 ± 0.1	1.0 × 10^3^	55.8 ± 0.1	1.0 : 1.8	+1.5 ± 0.1
*S*_p_-Zn6a	+48	1.0 × 10^3^	55.8 ± 0.1	5.6 × 10^2^	57.3 ± 0.1	1.8 : 1.0	−1.5 ± 0.2
*R*_p_-Zn6b	−8 → +9^d^	2.4 × 10^2^	59.4 ± 0.1	3.6 × 10^2^	58.4 ± 0.1	1.0 : 1.5	+1.0 ± 0.1
*S*_p_-Zn6b	+6 → −10^d^	3.5 × 10^2^	58.5 ± 0.1	2.4 × 10^2^	59.4 ± 0.1	1.5 : 1.0	−0.9 ± 0.2

a Estimated error 10%.

b Values taken from ref. 3*b*.

c By definition.

d Couplet as a result of exciton coupling.

Table 1 also allows one to compare the threading rates of two hosts with the same planar chirality but opposite enantiomeric substituents onto the same chiral guest, *e.g. R*_p_-H_2_3a and *R*_p_-H_2_3b onto (*R*,*R*)-7. The measured range of *k*_on(1)_ : *k*_on(2)_ ratios for the different combinations of one enantiomeric guest and two hosts with opposite enantiomeric substituents spans a range of *k*_on(1)_ : *k*_on(2)_ = 1.3–2.9, which is rather similar to the range for one particular chiral host and two enantiomeric guests (*k*_on(1)_ : *k*_on(2)_ = 1.0–3.3, see Table 1).

The results presented in Table 1 clearly illustrate that the planar chirality of the chiral porphyrin cages, except for Zn2, dictates the sign of the kinetic enantio-recognition (ΔΔ*G*^‡^_on_) of the guest, while the substituent determines the magnitude of the selectivity. In order to see whether the stereoselectivity of the threading process could be correlated to the chiral environment of the porphyrin macrocyclic hosts, ECD measurements were carried out. The molar circular dichroism (Δ*ε*) associated with the Soret band of the porphyrin (*λ*_max_ ≈ 420 nm) was assumed to be a measure of the extent by which the substituent attached to the xylylene sidewall induces a chiral effect. It was already shown before that the nitro-functionalized porphyrin cages H_2_2 and Zn2 possess nearly identical ECD spectra, with the sign and magnitude of their Cotton effects being very similar.^6*c*^ Hence, the presence or absence of the zinc(ii) metal center does not influence the chiral induction caused by the substituent attached to the xylylene side wall, and thus it is valid to compare the free base and zinc(ii) cages amongst each other. All planar chiral porphyrin macrocyclic hosts, except the enantiomers of helicene-functionalized host Zn6b displayed ordinary Cotton effects for their Soret bands. All chiral hosts with the *R*_p_-configuration displayed a negative Cotton effect, and those with the *S*_p_-configuration had a positive Cotton effect of similar magnitude. In contrast, the enantiomers of Zn6b displayed couplets for the Soret band, presumably because of exciton coupling with the appended helicene moieties.^9^ In Fig. 2A the kinetic enantio-recognition (ΔΔ*G*^‡^_on_) of each investigated host is plotted as a function of the magnitude of the Cotton effect of the Soret band at 420 nm in the CD spectrum. A clear trend is observed between the sign and magnitude of the Cotton effect and the degree of enantio-recognition. Strong negative Cotton effects, such as observed for *R*_p_-Zn4a/b, *R*_p_-Zn5a/b, and to a lower extent *R*_p_-Zn6a, are associated with a significant enantio-recognition of (*S*,*S*)-7 over (*R*,*R*)-7. The hosts that displayed weak negative Cotton effects, such as *R*_p_-H_2_3a/b, displayed poor enantio-recognition of either enantiomer of guest 7. Similar but opposite relationships were observed for the hosts that displayed positive Cotton effects. The enantiomers of Zn6b are not included in Fig. 2A, since they displayed couplets for their Soret bands. Nonetheless, their ability to recognize the enantiomers of 7 could still be correlated to their planar chirality, *i.e. R*_p_-Zn6b preferred (*S*,*S*)-7 over (*R*,*R*)-7.

**Fig. 2:**
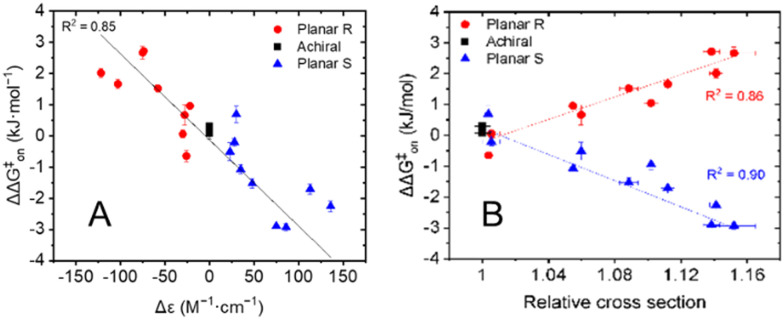
(A) Plot displaying the chiral selectivity (kinetic enantio-recognition) in the threading processes of various achiral and planar chiral hosts onto guests (*R*,*R*)-7 and (*S*,*S*)-7 as a function of the sign and magnitude of the Soret band at *λ*_max_ ≈ 420 nm. (B) Kinetic enantio-recognition for the threading of various achiral and planar-chiral hosts onto the enantiomers of guest 7 as a function of the relative collisional cross section (CCS) of the [M^+^] ion.

The enantio-recognition of the different hosts was also correlated to the size of the substituent attached to the xylylene sidewalls. Traditionally, the steric parameters applied in linear-free energy relationships, such as the Hammett equation^10*a*^ or the Taft equation^10*b*,*c*^ are used for this. Unfortunately, these steric parameters are not available for the large substituents attached to the porphyrin cage compounds. To overcome this, we used ion mobility-mass spectrometry (IMS-MS) to parametrize the size of the substituents.^5^ As far as we know, such an approach has not been reported before in linear-free energy relationships. Solutions of the achiral and chiral hosts in CH_2_Cl_2_/CH_3_CN (1 : 20, v/v) were subjected to ionization. Since the free-base and Zn(ii) porphyrin cage complexes are neutral in solution, the detected ions correspond to radical cations [M]^+^˙ or sodium adducts [M + Na]^+^. These ions were subjected to IMS to yield the collisional cross-section of each ion (CCS), see Table S3 in ESI.† The presented values are the average collisional cross sections, representing the sizes of the ions, and the full-width-at-half-maximum (FWHM), representing the distribution of the sizes. The relative sizes of the substituted porphyrin cages 2–6 were obtained using the following correction factors: the CCS values of the free base porphyrin cages H_2_1–H_2_3 were normalized against H_2_1, and the CCS values of the zinc(ii) porphyrin cages Zn1–Zn6 were normalized against Zn1. The results in Table S3 (ESI†) indicate that the relative sizes of the substituted porphyrin cages with respect to the non-substituted porphyrin cages are nearly identical for both the [M]^+^˙ and [M + Na]^+^ ions, ruling out the possibility that a specific type of ionization distorts the cage structure. Note that the IMS-MS studies were only carried out on the *S*_p_-enantiomers of the chiral porphyrin cages. By definition, the values for their *R*_p_-enantiomers are identical. The CCS values of the planar chiral porphyrin cages relative to the parent achiral porphyrin cages were used to measure the size of the substituents attached to the xylylene sidewalls. In Fig. 2B the kinetic enantio-recognition (ΔΔ*G*^‡^_on_) for the threading of achiral and chiral hosts 1–6 onto the enantiomers of guest 7 are plotted as a function of the relative collisional cross-section of their radical cations/molecular ions [M]^+^˙ relative to that of the collisional cross-section of the non-substituted hosts H_2_1 and Zn1 (the free base and zinc(ii) porphyrins are combined in one plot). The same plot was generated for the corresponding sodium adducts [M + Na]^+^ of the hosts (not shown). Fig. 2B clearly illustrate that the planar chirality of the chiral porphyrin cage compounds dictates the chiral preference, *i.e.*, the sign of the kinetic enantio-recognition, while the size of the substituent determines the extent to which the chiral porphyrin cages can discriminate between the enantiomers of guest 7, *i.e.* the magnitude of the kinetic enantio-recognition effect. The data points in Fig. 2B were fitted according to a linear function. However, It should be noted that the correlation between the kinetic enantio-recognition and the substituent size is not necessarily linear. Nevertheless, this function was used to show the trends.

In a previous study, we demonstrated that a viologen guest with attached tails can enter the porphyrin macrocyclic host in two ways: either directly or by first binding to the outside of the host. After this initial outside binding, the tail end loops back into the cavity through one opening (known as the entron effect) and then threads further through the other opening.^4*b*^ This second mechanism is preferred for guests with tails containing 8–22 carbon atoms. We hypothesize that chiral guest 7, which has two tails consisting of approximately 20 atoms each, enters the planar chiral porphyrin macrocyclic hosts using the second mechanism. The observed chiral discrimination could occur at one or more stages of the threading process: (i) during the pre-association with the chiral host, (ii) when the chiral tail end enters the chiral cavity (from either the substituted or non-substituted side), or (iii) when the tail end threads further through the second opening. Further studies are necessary to distinguish between these different possibilities.

In conclusion, the results of this study demonstrate that planar chiral porphyrin cage compounds can distinguish between the enantiomers of a viologen guest equipped with chiral chain termini, with kinetic enantio-recognition values of up to 3 kJ mol^−1^, similar to those reported for a different chiral porphyrin cage in a previous study.^3*b*^ The planar chirality in the porphyrin cage compounds dictates which enantiomer of the chiral guest is preferentially threaded and the size of the substituent determines the magnitude of the enantio-recognition effect. These results may pave the way for directionally threading a polymer chain through a chiral porphyrin cage, and future studies may explore the potential of larger substituents both on the host and on the guest to achieve even higher selectivities than those reported in this work.

This work was funded by the European Research Council (ERC Advanced Grant No. 740925 to R. J. M. N.), the Dutch Research Council (VI.C.192.044 and OCENW.KLEIN.348 to J. R.), and by the Dutch Ministry of Education, Culture, and Science (Gravitation program 024.001.035).

## Conflicts of interest

There are no conflicts to declare.

## Supplementary Material

CC-059-D3CC04934E-s001

## References

[cit1] Bonner W. A. (1995). Origins Life Evol. Biospheres.

[cit2] Kirby A. J. (1996). Angew. Chem., Int. Ed. Engl..

[cit3] Wang B.-Y., Stojanović S., Turner D. A., Young T. L., Hadad C. M., Badjić J. D. (2013). Chem. – Eur. J..

[cit4] Thordarson P., Bijsterveld E. J. A., Rowan A. E., Nolte R. J. M. (2003). Nature.

[cit5] Pu Y., Ridgeway M. E., Glaskin R. S., Park M. A., Costello C. E., Cheng Lin C. (2016). Anal. Chem..

[cit6] Elemans J. A. A. W., Claase M. B., Aarts P. P. M., Rowan A. E., Schenning A. P. H. J., Nolte R. J. M. (1999). J. Org. Chem..

[cit7] Coumans R. G. E., Elemans J. A. A. W., Nolte R. J. M., Rowan A. E. (2006). Proc. Natl. Acad. Sci. U. S. A..

[cit8] Hidalgo Ramos P., Coumans R. G. E., Deutman A. B. C., Smits J. M. M., De Gelder R., Elemans J. A. A. W., Nolte R. J. M., Rowan A. E. (2007). J. Am. Chem. Soc..

[cit9] Ouyang J. , Gilissen P. J., Dhbaibi K., Vanthuyne N., Chentouf S., Rutjes F. P. J. T., Naubron J.-V., Crassous J., Elemans J. A. A. W. and Nolte R. J. M., Manuscript in preparation

[cit10] Hammett L. P. (1937). J. Am. Chem. Soc..

