# Supplementary material for: Kinetic enantio-recognition of chiral viologen guests by planar-chiral porphyrin cages
Source: Chem Commun (Camb). 2023 Nov 2;59(94):13974–7. doi: 10.1039/d3cc04934e (PMC10667586; doi:10.1039/d3cc04934e)
Supplement: CC-059-D3CC04934E-s001 [file CC-059-D3CC04934E-s001.pdf]

Electronic Supplementary Material (ESI) for ChemComm.
This journal is © The Royal Society of Chemistry 2023

Supporting Information

Effects of substituents on the kinetic enantio-recognition of chiral viologen guests by planar-chiral porphyrin cages

Pieter J. Gilissen, Quentin Duez, Guilherme L. Tripodi, Magda M.J. Dekker, Jiangkun Ouyang, Kais Dhbaibi, Nicolas Vanthuyne, Jeanne Crassous, Jana Roithová, Johannes A.A.W. Elemans, and Roeland J.M. Nolte

Contents

1. Synthesis........ 2
1.1. Synthesis of compound **Zn5**........2
1.2. Synthesis of compound **Zn6**........10
2. Threading........13
2.1 Threading procedure........13
2.2 Typical threading experiments........13
2.3 Tables with kinetic threading data 13
3. Ion mobility experiments........16
3.1 Ion mobility procedure........16
3.2 Table of collisional cross sections........ .17
4. Spectra........18
4.1. CD spectra........18
4.2 Fluorescence threading spectra........23
4.3 NMR spectra of synthesized compounds........64
4.4 NMR spectra of host-guest complexes........88
5. References........100

1. Synthesis

1.1. Synthesis of compound Zn5

Compound **Zn5** was prepared according to the route shown in Scheme S1.

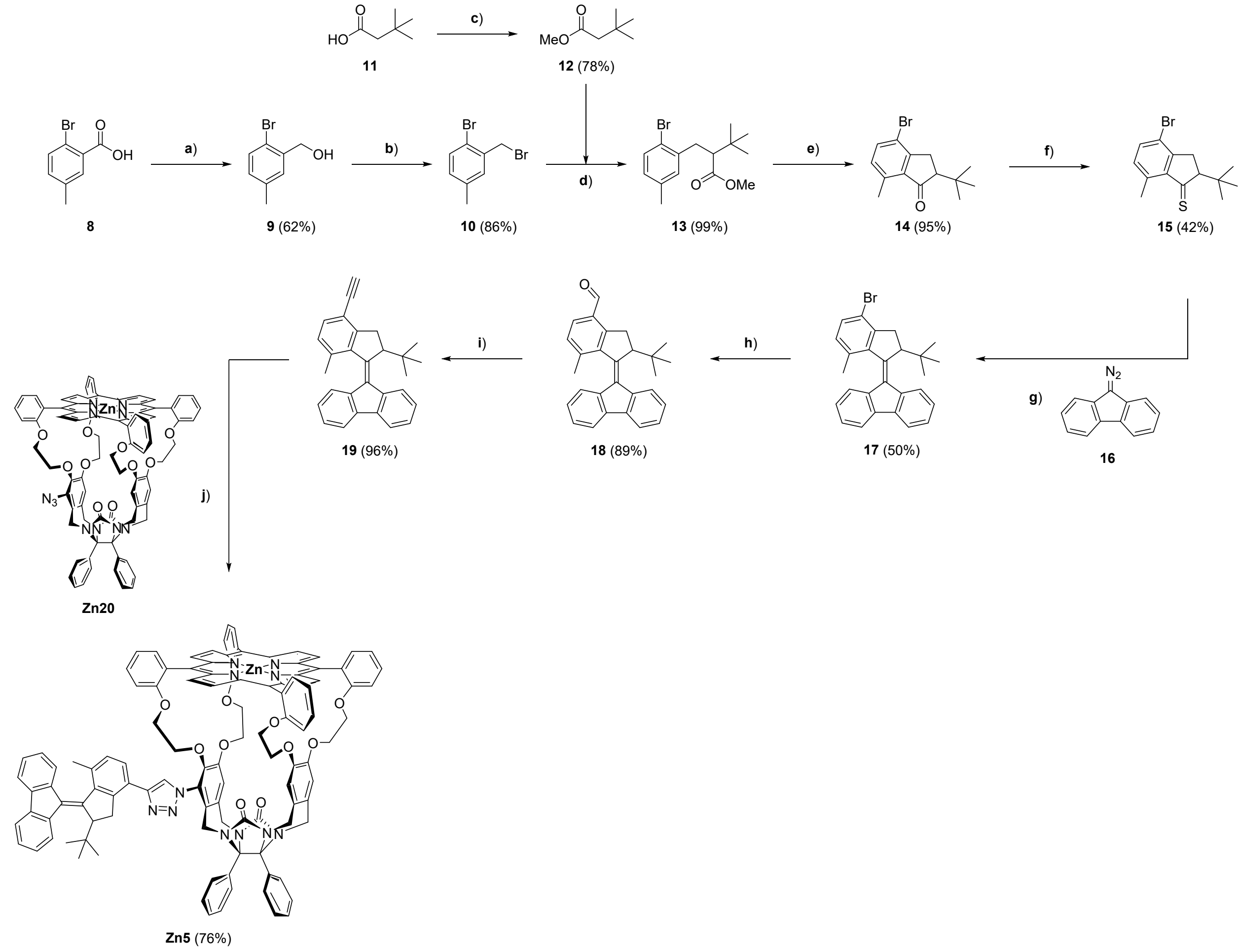

Scheme S1. Synthesis route for the preparation of compound **Zn5.** Reagents and conditions: a) $LiAlH_4$, THF, 0 → 20 °C; b) PBr_3, Et_2O, 0 °C; c) MeI, K_2CO_3, DMF, 20 °C; d) LDA, THF, −78 → 20 °C; e) H_2SO_4, 20 °C; f) P_2S_5 (as the dimer P_4S_{10}), toluene, reflux; g) toluene, reflux; then PPh_3, toluene, reflux; h) ^{n}BuLi, THF, −78 °C; then DMF, THF, −78 → 20 °C; i) dimethyl-1-diazo-2-oxopropylphosphonate, K_2CO_3, MeOH/THF, 0 → 20 °C; j) CuI, DIPEA, sodium ascorbate, DMSO/H_2O, 20 °C.

*2-Bromo-5-methylbenzyl alcohol (**9**)*

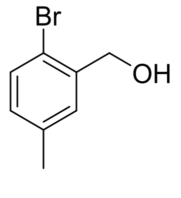

9

Compound **9** was synthesized according to a modified literature procedure.[S1] $LiAlH_4$ (4.7 g, 130 mmol, 2.5 equiv.) was suspended in dry THF (250 mL) under an argon atmosphere, and the mixture was cooled to 0 °C. Then, a solution of 2-bromo-5-methylbenzoic acid **8** (11 g, 50 mmol, 1.0 equiv.) in dry THF (50 mL) was added dropwise over 10 minutes. After stirring the suspension for 1 hour at 0 °C and subsequently for 2 hours at 20 °C, the reaction was quenched by the careful addition of $Na_2SO_4 \cdot 10H_2O$ (40 g). Once the suspension turned white and the effervescence had ceased, the suspension was filtered to remove the insoluble salts, and the solvent was removed in vacuo. The residue was partitioned between 1M aqueous HCl (250 mL) and CH_2Cl_2 (250 mL). The organic layer was separated, dried over Na_2SO_4 and the solvent was removed in vacuo. The crude product was purified by 60A silica gel column chromatography (eluent: CH_2Cl_2/*n*-heptane, 1:1, v/v) to

afford alcohol **9** (6.3 g, 62%) as a white solid. TLC (eluent: CH_2Cl_2) R_f 0.35. ^{1}H NMR (400 MHz, $CDCl_3$) δ 7.41 (d, *J* = 8.1 Hz, 1H), 7.28 (d, *J* = 2.0 Hz, 1H), 6.97 (dd, *J* = 8.1, 2.2 Hz, 1H), 4.71 (d, *J* = 6.0 Hz, 2H), 2.32 (s, 3H), 2.01 (t, *J* = 6.2 Hz, 1H). ^{13}C NMR (101 MHz, $CDCl_3$) δ 139.42, 137.79, 132.47, 130.04, 129.94, 119.34, 65.26, 21.07. Analytical data were in agreement with literature values.[S1]

*2-Bromo-5-methylbenzyl bromide (**10**)*

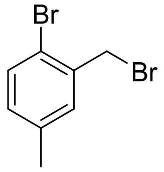

10

Compound **10** was synthesized according to a literature procedure.[S2] Phosphorus tribromide (3.1 mL, 33 mmol, 1.1 equiv.) was added dropwise over 5 minutes to a solution of alcohol **9** (6.0 g, 30 mmol, 1.0 equiv.) in dry Et_2O (140 mL) under an argon atmosphere. The resulting mixture was stirred at 0 °C for 2.5 hours. Upon completion (indicated by TLC, eluent: CH_2Cl_2/*n*-heptane, 1:1, v/v), the reaction was quenched by the successive addition of MeOH (15 mL) and water (150 mL). The organic layer was separated, and the aqueous layer was extracted with Et_2O (50 mL). The combined organic extracts were washed with 0.6M aqueous $NaHCO_3$ (200 mL), then dried over Na_2SO_4 and the solvent was removed in vacuo. The crude product was purified by 60A silica gel column chromatography (eluent: *n*-heptane) to afford bromide **10** (6.8 g, 86%) as a white solid. TLC (eluent: CH_2Cl_2/*n*-heptane, 1:1, v/v) R_f 0.72. ^{1}H NMR (400 MHz, $CDCl_3$) δ 7.44 (d, *J* = 8.1 Hz, 1H), 7.26 (d, *J* = 2.2 Hz, 1H), 6.97 (dd, *J* = 8.1, 2.2 Hz, 1H), 4.57 (s, 2H), 2.30 (s, 3H). **^{13}C NMR** (101 MHz, $CDCl_3$) δ 138.12, 136.78, 133.21, 132.09, 131.18, 121.20, 33.67, 20.92. Analytical data were in agreement with literature values.[S1,S2]

*Methyl 3,3-dimethylbutanoate (**12**)*

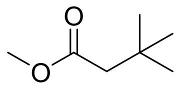

12

Compound **12** was synthesized according to a literature procedure.[S3] A mixture of 3,3-dimethylbutanoic acid **11** (27 mL, 0.21 mol, 1.0 equiv.), iodomethane (20 mL, 0.32 mol, 1.5 equiv.), and K_2CO_3 (25 g, 0.18 mol, 0.85 equiv.) in DMF (150 mL) was stirred at 20 °C for 22 hours. Et_2O (100 mL) was added and the organic layer was washed with water (6 × 200 mL), then dried over Na_2SO_4, and the solvent was removed under reduced pressure (T_{bath} = 40 °C, $p > 200$ mbar). The crude product was distilled at atmospheric pressure to afford ester **12** (21 g, 78%) as a colorless liquid. B.p. 125–130 °C (lit. 124–128 °C).[S4] ^{1}H NMR (400 MHz, $CDCl_3$) δ 3.65 (s, 3H), 2.20 (s, 2H), 1.03 (s, 9H). ^{13}C NMR (101 MHz, $CDCl_3$) δ 172.84, 51.12, 47.87, 30.74, 29.68. Analytical data were in agreement with literature values.[S3]

*Methyl 2-(2-bromo-5-methylbenzyl)-3,3-dimethylbutanoate (**13**)*

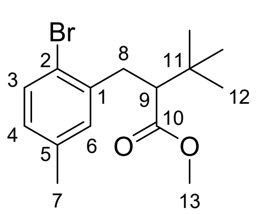

13

^{n}BuLi (32 mL, 51 mmol, 3.0 equiv., 1.6M in hexanes) was added dropwise at 0 °C to a solution of diisopropylamine (7.3 mL, 51 mmol, 3.0 equiv.) in dry THF (150 mL) under an argon atmosphere. The resulting mixture was stirred at 0 °C for 60 minutes and subsequently cooled to −78 °C. Then, methyl 3,3-dimethylbutanoate **12** (7.4 mL, 51 mmol, 3.0 equiv.) was added and the clear solution was stirred at −78 °C for 60 minutes. Then, a solution of 1-bromo-2-(bromomethyl)-4-methylbenzene **10** (4.5 g, 17 mmol, 1.0 equiv.) in dry THF (50 mL) was added dropwise, the resulting solution was warmed to 20 °C, and it was stirred at 20 °C for 3 hours. Upon complete conversion of the alkyl bromide (indicated by TLC, eluent: CH_2Cl_2/*n*-heptane, 1:1, v/v), the reaction was quenched with aqueous 2M NH_4Cl (200 mL). The organic layer was separated and the aqueous layer was extracted with EtOAc (100 mL). The combined organic layers were dried over Na_2SO_4 and the solvent was removed in vacuo. The crude product was purified by 60A silica gel column chromatography (eluent: CH_2Cl_2/*n*-heptane, 1:3, v/v) to afford ester **13** (5.3 g, 99%) as a colorless transparent oil that crystallized upon standing into white needles. TLC (eluent: CH_2Cl_2/*n*-heptane, 1:1, v/v) R_f 0.50. m.p. 64–66 °C. ^{1}H NMR (400 MHz, $CDCl_3$) δ 7.37 (d, *J* = 8.1 Hz, 1H, H-3), 6.96 (d, *J* = 2.2 Hz, 1H, H-6), 6.86 (dd, *J* = 8.1, 2.2 Hz, 1H, H-4), 3.48 (s, 3H, H-13), 3.03 (dd, *J* = 13.1, 2.9 Hz,

1H, H-8a), 2.87 (dd, *J* = 13.1, 12.1 Hz, 1H, H-8b), 2.60 (dd, *J* = 12.1, 2.8 Hz, 1H, H-9), 2.24 (s, 3H, H-7), 1.07 (s, 9H, H-12). ^{13}C NMR (101 MHz, $CDCl_3$) δ 174.87 (C-10), 138.96 (C-1), 137.12 (C-5), 132.57 (C-3), 131.71 (C-6), 128.83 (C-4), 121.08 (C-2), 56.05 (C-9), 50.75 (C-13), 34.37 (C-8), 33.48 (C-11), 27.76 (C-12), 20.79 (C-7). HRMS (EI) *m/z* calcd. for $[C_{15}H_{21}{}^{79}BrO_2]^+$ 312.07249, found 312.06986.

*4-Bromo-2-(tert-butyl)-7-methyl-2,3-dihydro-1H-inden-1-one (**14**)*

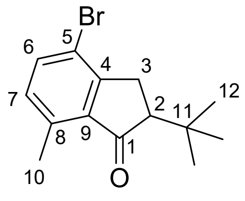

14

Ester **13** (5.0 g, 16 mmol, 1.0 equiv.) was added to concentrated H_2SO_4 (80 mL) and the resulting mixture was vigorously stirred at 20 °C for 4 hours. Upon completion (indicated by TLC, eluent: EtOAc/*n*-heptane, 1:9, v/v), the mixture was poured into ice water (500 mL) and the resulting suspension was stirred vigorously and then filtered. The precipitate was washed with water (500 mL) and dried under high vacuum to afford ketone **14** (4.3 g, 95%) as a white solid. TLC (eluent: EtOAc/*n*-heptane, 1:9, v/v) R_f 0.56. m.p. 52–53 °C. ^{1}H NMR (400 MHz, $CDCl_3$) δ 7.56 (d, *J* = 7.9 Hz, 1H, H-6), 6.98 (dq, *J* = 8.0, 0.9 Hz, 1H, H-7), 3.08 (dd, *J* = 17.9, 8.2 Hz, 1H, H-3a), 2.84 (dd, *J* = 17.9, 4.5 Hz, 1H, H-3b), 2.57 (d, *J* = 0.7 Hz, 3H, H-10), 2.47 (dd, *J* = 8.2, 4.4 Hz, 1H, H-2), 1.06 (s, 9H, H-12). ^{13}C NMR (101 MHz, $CDCl_3$) δ 208.19 (C-1), 153.26 (C-4), 137.69 (C-8), 137.04 (C-9), 136.29 (C-6), 131.02 (C-7), 118.64 (C-5), 56.90 (C-2), 33.88 (C-11), 30.73 (C-3), 27.56 (C-12), 17.92 (C-10). HRMS (EI) *m/z* calcd. for $[C_{14}H_{17}{}^{79}BrO]^+$ 280.04628, found 280.04747.

*4-Bromo-2-(tert-butyl)-7-methyl-2,3-dihydro-1H-inden-1-thione (**15**)*

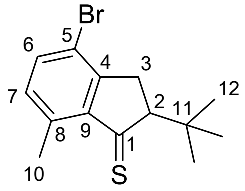

15

Phosphorus decasulfide (68 g, 0.15 mol, 11 equiv.) was added to a solution of ketone **14** (3.9 g, 14 mmol, 1.0 equiv.) in dry toluene (300 mL) and the resulting mixture was refluxed for 47 hours under an argon atmosphere. After cooling, the mixture was filtered through a plug of silica. The filter cake was washed with toluene until colorless washings were obtained. The combined filtrate was evaporated to dryness and quickly purified by 60A silica gel column chromatography (eluent: toluene/*n*-heptane, 1:10, v/v) to afford unstable thioketone **15** (1.7 g, 42%) as a dark blue oil. *Note: compound **15** rapidly decomposes, in the solid state, in solution, and on silica. Therefore, it should be used immediately in the next reaction*. TLC (eluent: toluene/*n*-heptane, 1:7, v/v) R_f 0.59. ^{1}H NMR (400 MHz, $CDCl_3$) δ 7.53 (d, *J* = 8.0 Hz, 1H, H-6), 6.99 (dq, *J* = 8.0, 0.9 Hz, 1H, H-7), 3.15 (dd, *J* = 17.9, 3.2 Hz, 1H, H-3a), 3.11 (dd, *J* = 17.9, 5.4 Hz, 1H, H-3b), 3.03 (dd, *J* = 5.4, 3.2 Hz, 1H, H-2), 2.64 (d, *J* = 0.8 Hz, 3H, H-10), 0.95 (s, 9H, H-12). ^{13}C NMR (101 MHz, $CDCl_3$) δ 249.07 (C-1), 153.11 (C-4), 145.81 (C-9), 138.06 (C-8), 135.13 (C-6), 132.12 (C-7), 117.82 (C-5), 70.93 (C-2), 35.47 (C-3), 35.18 (C-11), 27.63 (C-12), 21.29 (C-10). HRMS (EI) *m/z* calcd. for $[C_{14}H_{17}{}^{79}BrS]^+$ 296.02343, found 296.01854.

*9-(4-Bromo-2-(tert-butyl)-7-methyl-2,3-dihydro-1H-inden-1-ylidene)-9H-fluorene (**17**)*

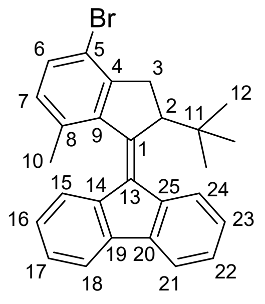

17

A solution of thioketone **15** (1.3 g, 4.4 mmol, 1.0 equiv.) and diazo compound **16**[S6] (1.8 g, 9.4 mmol, 2.1 equiv.) in dry toluene (250 mL) was refluxed in the dark for 20 hours under an argon atmosphere. Then, triphenylphosphine (4.5 g, 17 mmol, 3.9 equiv.) was added and refluxing was continued for 2 hours. After cooling, the solvent was removed in vacuo and the crude product was purified by 60H silica gel column chromatography (eluent: *n*-heptane). The fractions containing the desired product were evaporated to dryness and recrystallized from a minimal amount of EtOH to afford motor **17** (0.94 g, 50%) as a yellow solid. TLC (eluent: *n*-heptane) R_f 0.33. M.p. 97–98 °C. ^{1}H NMR (500 MHz, $CDCl_3$) δ 8.18 (d, *J* = 7.4 Hz, 1H, H-24), 7.80–7.77 (m, 1H, H-21), 7.72 (dt, *J* = 7.6, 0.9 Hz, 1H, H-18), 7.38 (d, *J* = 8.4 Hz, 1H, H-15), 7.37 (d, *J* = 7.9 Hz, 1H, H-6), 7.38–7.34 (m, 1H,

H-22), 7.34 (td, *J* = 7.4, 1.5 Hz, 1H, H-23), 7.28 (td, *J* = 7.4, 1.0 Hz, 1H, H-17), 7.08 (ddd, *J* = 8.3, 7.4, 1.2 Hz, 1H, H-16), 6.98 (d, *J* = 8.1 Hz, H-7), 4.17 (d, *J* = 5.8 Hz, 1H, H-2), 3.08 (dd, *J* = 15.4, 5.8 Hz, 1H, H-3a), 2.94 (d, *J* = 15.3 Hz, 1H, H-3b), 2.25 (s, 3H, H-10), 0.87 (s, 9H, H-12). ^{13}C NMR (126 MHz, $CDCl_3$) δ 149.78 (C-1), 147.14 (C-4), 144.87 (C-9), 140.81 (C-20), 139.70 (C-19), 139.28 (C-25), 137.94 (C-14), 134.85 (C-8), 132.28 (C-13), 131.81 (C-6), 130.11 (C-7), 127.79 (C-17), 127.55 (C-22), 126.82 (C-16), 126.55 (C-23), 125.18 (C-24), 123.93 (C-15), 119.77 (C-21), 119.21 (C-18), 116.99 (C-5), 58.36 (C-2), 38.25 (C-3), 35.76 (C-11), 29.03 (C-12), 20.65 (C-10). UV-Vis (CH_2Cl_2) λ/nm (log(ε/M^{-1}·cm^{-1})) 352 (4.28). MS (MALDI-TOF) *m/z* calcd. for $[C_{27}H_{25}{}^{79}Br - C_4H_9]^+$ 371.0, found 371.2. HRMS (EI) *m/z* calcd. for $[C_{27}H_{25}{}^{79}Br]^+$ 428.11396, found 428.11164.

*2-(tert-Butyl)-1-(9H-fluoren-9-ylidene)-7-methyl-2,3-dihydro-1H-indene-4-carbaldehyde (**18**)*

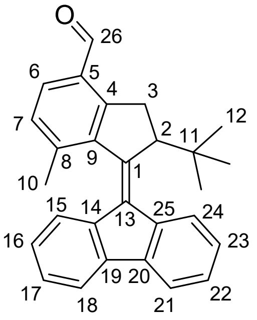

18

^{n}BuLi (2.3 mL, 3.7 mmol, 2.2 equiv., 1.6M in hexanes) was added at −78 °C to a pale yellow solution of motor **17** (0.72 g, 1.7 mmol, 1.0 equiv.) in dry THF (30 mL) under an argon atmosphere. The resulting dark orange solution was stirred at −78 °C for 50 minutes and then dry DMF (1.4 mL, 18 mmol, 11 equiv.) was added. The resulting pale orange solution was slowly warmed to 20 °C and stirring was continued for 4 hours. The reaction was quenched by the addition of 3M aqueous NH_4Cl (40 mL) and the product was extracted with EtOAc (3 × 60 mL). The combined organic extracts were washed with water (90 mL) and brine (90 mL), then dried over Na_2SO_4, and the solvent was removed in vacuo. The crude product was purified by 60A silica gel column chromatography (eluent: CH_2Cl_2/*n*-heptane, 1:1, v/v) to afford aldehyde **18** (0.57 g, 89%) as a yellow solid. TLC (eluent: CH_2Cl_2/*n*-heptane, 1:1, v/v) R_f 0.39. M.p. 92–93 °C. ^{1}H NMR (500 MHz, $CDCl_3$) δ 10.18 (s, 1H, H-26), 8.24–8.18 (m, 1H, H-24), 7.82–7.76 (m, 1H, H-21), 7.75–7.70 (m, 1H, H-18), 7.72 (d, *J* = 7.8 Hz, 1H, H-6), 7.41–7.35 (m, 1H, H-22), 7.38–7.32 (m, 1H, H-23), 7.32–7.27 (m, 1H, H-17), 7.29 (d, *J* = 7.8 Hz, 1H, H-7), 7.28 (d, *J* = 7.6 Hz, 1H, H-15), 7.07 (ddd, *J* = 8.2, 7.2, 1.2 Hz, 1H, H-16), 4.26 (d, *J* = 5.8 Hz, 1H, H-2), 3.52 (d, *J* = 16.3 Hz, 1H, H-3a), 3.31 (dd, *J* = 16.4, 5.8 Hz, 1H, H-3b), 2.39 (s, 3H, H-10), 0.86 (s, 9H, H-12). ^{13}C NMR (126 MHz, $CDCl_3$) δ 191.97 (C-26), 150.18 (C-4), 148.51 (C-1), 144.65 (C-9), 142.10 (C-8), 140.83 (C-20), 139.85 (C-19), 139.28 (C-25), 137.97 (C-14), 132.51 (C-13), 131.74 (C-6), 130.14 (C-5), 128.93 (C-7), 127.92 (C-17), 127.67 (C-22), 126.78 (C-16), 126.63 (C-23), 125.27 (C-24), 123.68 (C-15), 119.80 (C-21), 119.33 (C-18), 58.68 (C-2), 36.02 (C-3), 35.85 (C-11), 29.08 (C-12), 21.58 (C-10). UV-Vis (CH_2Cl_2) λ/nm (log(ε/M^{-1}·cm^{-1})) 340 (4.21), 360 (4.24). MS (MALDI-TOF) *m/z* calcd. for $[C_{28}H_{26}O - C_4H_9]^+$ 321.1, found 321.1. HRMS (EI) *m/z* calcd. for $[C_{28}H_{26}O]^+$ 378.19836, found 378.19773.

*9-(2-(tert-Butyl)-4-ethynyl-7-methyl-2,3-dihydro-1H-inden-1-ylidene)-9H-fluorene (**19**)*

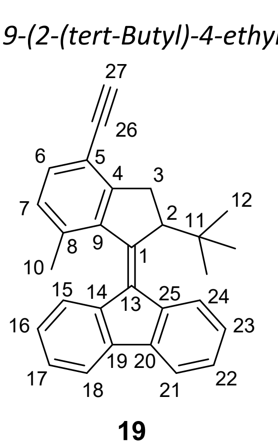

19

K_2CO_3 (69 mg, 0.50 mmol, 2.0 equiv.) was added at 0 °C to a solution of aldehyde **18** (95 mg, 0.25 mmol, 1.0 equiv.) and dimethyl (1-diazo-2-oxopropyl)phosphonate (56 µL, 0.38 mmol, 1.5 equiv.) in dry MeOH/dry THF (6 mL, 1:1, v/v) under an argon atmosphere. The resulting suspension was vigorously stirred at 20 °C for 7 hours. Then, additional dimethyl (1-diazo-2-oxopropyl)phosphonate (39 µL, 0.25 mmol, 1.0 equiv.) and K_2CO_3 (69 mg, 0.50 mmol, 2.0 equiv.) were added and stirring was continued for 15 hours. Upon completion of the reaction (indicated by TLC, eluent: CH_2Cl_2/*n*-heptane, 1:1, v/v), the solvent was removed in vacuo. Water (30 mL) was added to the residue, and the product was extracted with CH_2Cl_2 (2 × 20 mL). The combined organic extracts were dried over Na_2SO_4 and the solvent was removed in vacuo. The crude product was purified by 60A silica gel column chromatography (eluent: CH_2Cl_2/*n*-pentane, 1:5, v/v) to afford alkyne **19** (90 mg, 96%) as an off-white solid. TLC (eluent: CH_2Cl_2/*n*-heptane, 1:1, v/v) R_f 0.65. M.p. 184–185 °C. ^{1}H NMR (500 MHz, $CDCl_3$) δ 8.23–8.18 (m, 1H, H-24), 7.81–

7.77 (m, 1H, H-21), 7.72 (dt, J = 7.5, 0.9 Hz, 1H, H-18), 7.37 (d, J = 7.9 Hz, 1H, H-6), 7.39–7.34 (m, 2H, H-15 + H-22), 7.33 (td, J = 7.4, 1.5 Hz, 1H, H-23), 7.28 (td, J = 7.5, 1.1 Hz, 1H, H-17), 7.08 (ddd, J = 8.3, 7.4, 1.2 Hz, 1H, H-16), 7.08 (d, J = 7.9 Hz, H-7), 4.21 (d, J = 5.6 Hz, 1H, H-2), 3.24 (s, 1H, H-27), 3.14 (dd, J = 15.5, 5.6 Hz, 1H, H-3a), 3.06 (d, J = 15.5 Hz, 1H, H-3b), 2.30 (s, 3H, H-10), 0.87 (s, 9H, H-12). ^{13}C NMR (126 MHz, $CDCl_3$) δ 150.79 (C-4), 149.81 (C-1), 143.18 (C-9), 140.76 (C-20), 139.63 (C-19), 139.41 (C-25), 138.10 (C-14), 136.73 (C-8), 132.24 (C-6), 131.85 (C-13), 128.47 (C-7), 127.66 (C-17), 127.42 (C-22), 126.78 (C-16), 126.50 (C-23), 125.18 (C-24), 123.92 (C-15), 119.75 (C-21), 119.18 (C-18), 115.93 (C-5), 81.84 (C-26), 80.01 (C-27), 58.71 (C-2), 36.63 (C-3), 35.76 (C-11), 29.07 (C-12), 21.15 (C-10). UV-Vis (CH_2Cl_2) λ/nm (log(ε/M^{-1}·cm^{-1})) 355 (4.34). **MS** (MALDI-TOF) *m/z* calcd. for $[C_{29}H_{26} - C_4H_9]^+$ 317.1, found 317.1. HRMS (EI) *m/z* calcd. for $[C_{29}H_{26}]^+$ 374.20345, found 374.19942.

*Molecular motor-functionalized porphyrin cages (**Zn5a** and **Zn5b**)*

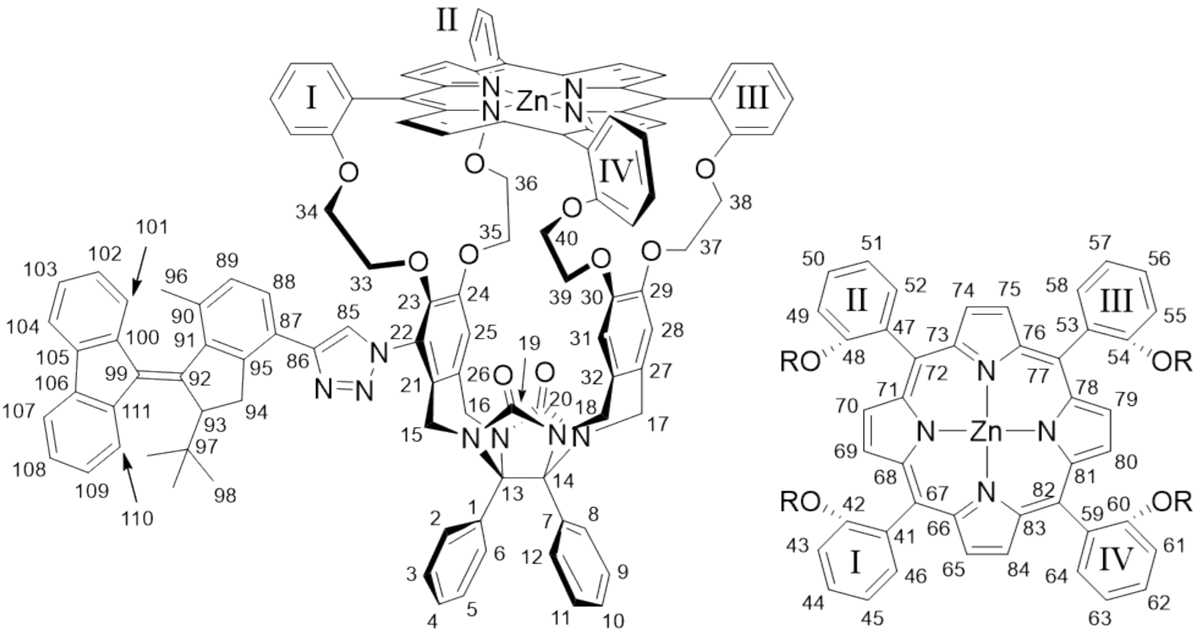

Sodium ascorbate (0.39 g, 2.0 mmol, 10 equiv.), copper(I) iodide (75 mg, 0.39 mmol, 2.0 equiv.) and DIPEA (0.34 mL, 2.0 mmol, 10 equiv.) were successively added to a solution of zinc(II) azido porphyrin cage compound **Zn20**[S5] (0.28 g, 0.20 mmol, 1.0 equiv) and alkyne motor **19** (74 mg, 0.20 mol, 1.0 equiv.) in a deoxygenated mixture of DMSO (80 mL) and water (5 mL). The resulting purple mixture was stirred at 20 °C for 2 days under an argon atmosphere. Then, the reaction mixture was diluted with $CHCl_3$ (200 mL), washed with water (3 × 200 mL) and brine (200 mL), dried over Na_2SO_4, and the solvent was removed in vacuo. The crude product was purified by 60H silica gel column chromatography (eluent: $CHCl_3$/CH_3CN, 15:1, v/v). The first purple fraction was collected and unreacted zinc(II) azido porphyrin cage compound **Zn20** (34 mg, 12%) was recovered as a purple solid. The second purple fraction contained a 1:1 mixture of (±)-**Zn5a** and (±)-**Zn5b**. The purified material was dissolved in a minimal amount of CH_2Cl_2 (10 mL) and precipitated by the addition of *n*-heptane (20 mL). Most CH_2Cl_2 was removed under reduced pressure and the resulting suspension was centrifuged. The supernatant was removed and the precipitate was washed with *n*-pentane (3 × 10 mL) and dried under high vacuum to afford molecular motor-functionalized porphyrin cage compound **Zn5** (0.27 g, 76%) as a purple solid. The isomeric mixture of **Zn5** (0.23 g) was dissolved in EtOH/CH_2Cl_2 (7:13, v/v, 28 mL) and the stereoisomers were resolved by preparative chiral HPLC (Chiralpak ID column, 250 × 10 mm, eluent: EtOH/CH_2Cl_2, 1:1, v/v, flow rate 5 mL/min, UV detection at 254 nm)

using 140 stacked injections affording (−)-**Zn5a** (57 mg, first eluted isomer, e.e. > 99.5%), (−)-**Zn5b** (53 mg, second eluted isomer, e.e. > 99.5%), (+)-**Zn5b** (53 mg, third eluted isomer, e.e. > 99.5%), and (+)-**Zn5a** (57 mg, fourth eluted isomer, e.e. > 99.5%), all as purple solids. The enantiomeric purity was determined by chiral HPLC (Chiralpak ID column, eluent: EtOH/CH_2Cl_2, 1:1, v/v, flow rate 1 mL/min, UV detection at 420 nm, retention times of (−)-**Zn5a**, (−)-**Zn5b**, (+)-**Zn5b**, and (+)-**Zn5a** were 5.6, 6.5, 7.6, and 9.3 *min, respectively).*

*Analytical data for the first eluted isomer ((−)-**Zn5a**)*
TLC (eluent: $CHCl_3$/CH_3CN, 9:1, v/v) R_f 0.63. M.p. >300 °C. ^{1}H NMR (500 MHz, $CDCl_3$) δ 8.92 (d, *J* = 4.7 Hz, 1H, H-75), 8.92–8.87 (m, 2H, H-65 + H-74), 8.83 (d, *J* = 4.7 Hz, 1H, H-84), 8.79 (d, *J* = 4.7 Hz, 1H, H-79/80)*, 8.77 (d, *J* = 4.6 Hz, 1H, H-79/80)*, 8.71 (s, 2H, H-69 + H-70), 8.29–8.23 (m, 2H, H-46 + H-110), 8.13 (dd, *J* = 7.3, 1.7 Hz, 1H, H-58), 8.08 (dd, *J* = 7.3, 1.8 Hz, 1H, H-64), 8.04 (dd, *J* = 7.4, 1.7 Hz, 1H, H-52), 7.89–7.82 (m, 1H, H-107), 7.82 (s, 1H, H-85), 7.81–7.73 (m, 4H, H-50 + H-56 + H-62 + H-104), 7.69 (d, *J* = 7.7 Hz, 1H, H-88), 7.58 (td, *J* = 7.9, 1.7 Hz, 1H, H-44), 7.49 (d, *J* = 8.1 Hz, 1H, H-101), 7.45–7.31 (m, 10H, H-45 + H-49 + H-51 + H-55 + H-57 + H-61 + H-63 + H-103 + H-108 + H-109), 7.20–7.13 (m, 2H, H-89 + H-102), 7.03–6.96 (m, 1H, H-5), 6.94 (d, *J* = 8.2 Hz, 1H, H-43), 6.94–6.87 (m, 4H, H-4 + H-9 + H-10 + H-11), 6.87–6.80 (m, 1H, H-3), 6.75–6.68 (m, 2H, H-6 + H-12), 6.63–6.57 (m, 2H, H-2 + H-8), 6.23 (s, 1H, H-25), 6.17 (s, 1H, H-28), 6.13 (s, 1H, H-31), 4.26–4.20 (m, 2H, H-38a + H-40a), 4.18–4.14 (m, 1H, H-40b), 4.14 (d, *J* = 5.7 Hz, 1H, H-93), 4.14–4.01 (m, 4H, H-16a/17a + H-18a + H-36a + H-38b)*, 3.99–3.89 (m, 1H, H-16a/17a)*, 3.88–3.77 (m, 3H, H-15a + H-34a + H-36b), 3.75–3.67 (m, 2H, H-16b/17b + H-35a)*, 3.66–3.58 (m, 2H, H-16b/17b + H-18b + H-39a)*, 3.54 (d, *J* = 16.5 Hz, 1H, H-15b), 3.49–3.36 (m, 3H, H-34b + H-37a + H-39b), 3.33 (ddd, *J* = 10.9, 7.2, 4.2 Hz, 1H, H-37b), 3.27–3.17 (m, 3H, H-33a + H-35b + H-94a), 3.04–2.98 (m, 1H, H-33b), 2.98 (d, *J* = 15.2 Hz, 1H, H-94b), 2.38 (s, 3H, H-96), 0.79 (s, 9H, H-98). *Note: proton signals marked with an asterisk (*) could not be assigned unambiguously.* ^{13}C NMR (126 MHz, $CDCl_3$) δ 159.24 (C-42), 159.18 (C-48), 159.05 (C-60), 159.01 (C-54), 157.03 (C-20), 156.64 (C-19), 151.39 (C-92), 150.80 (C-66/68/71/73/76/78/81/83)*, 150.49 (C-66/68/71/73/76/78/81/83)*, 150.45 (C-66/68/71/73/76/78/81/83)*, 150.40 (C-66/68/71/73/76/78/81/83)*, 150.25 (C-66/68/71/73/76/78/81/83)*, 150.08 (C-24), 149.90 (C-66/68/71/73/76/78/81/83)*, 149.85 (C-66/68/71/73/76/78/81/83)*, 149.81 (C-66/68/71/73/76/78/81/83)*, 147.10 (C-30), 146.46 (C-29), 145.43 (C-86), 144.63 (C-95), 143.53 (C-91), 142.33 (C-23), 140.70 (C-106), 139.59 (C-111), 139.50 (C-105), 138.30 (C-100), 135.89 (C-64), 135.82 (C-52), 135.68 (C-58), 135.34 (C-90), 134.89 (C-46), 133.25 (C-1/7)*, 133.24 (C-1/7)*, 132.96 (C-47/53/59)*, 132.83 (C-47/53/59)*, 132.60 (C-47/53/59)*, 132.52 (C-26), 132.22 (C-41), 131.68 (C-69/70/79/80/84)*, 131.64 (C-69/70/79/80/84)*, 131.60 (C-69/70/79/80/84)*, 131.47 (2× C-69/70/79/80/84)*, 131.32 (C-65), 130.99 (C-99), 130.92 (C-74 + C-75), 130.70 (C-32), 130.02 (C-22), 129.65 (C-27), 129.59 (C-44 + C-50 + C-56 + C-62), 128.90 (C-3/4/5/9/10/11)*, 128.77 (C-3/4/5/9/10/11)*, 128.75 (C-3/4/5/9/10/11)*, 128.73 (C-3/4/5/9/10/11)*, 128.65 (C-3/4/5/9/10/11)*, 128.60 (C-89), 128.55 (C-3/4/5/9/10/11)*, 128.38 (C-2), 128.24 (C-8), 127.92 (C-12 + C-88), 127.70 (C-6), 127.44 (C-103), 127.16 (C-108), 126.94 (C-21), 126.84 (C-102), 126.49 (C-109), 125.15 (C-110), 125.13 (C-85), 124.46 (C-87), 124.16 (C-101), 120.37 (C-51), 120.16 (C-57), 120.07 (C-45), 119.99 (C-63), 119.74 (C-107), 119.11 (C-104), 116.85 (C-28), 116.42 (C-77), 116.28 (C-67 + C-82), 116.20 (C-72), 115.76 (C-31), 115.33 (C-25), 113.27 (C-49), 112.54 (C-55), 112.40 (C-43), 111.99 (C-61), 85.02 (C-14), 84.60 (C-13), 71.38 (C-33), 68.10 (C-37), 67.96 (C-36), 67.91 (C-39), 67.51 (C-35), 67.43 (C-40), 67.20 (C-38), 67.16 (C-34), 58.26 (C-93), 44.68 (C-16/17)*, 44.58 (C-16/17)*, 44.20 (C-18), 38.95 (C-15), 37.33 (C-94), 35.74 (C-97), 29.25 (C-98), 20.94 (C-96). *Note: carbon signals marked with an asterisk (*) could not be assigned unambiguously.* UV-Vis (CH_2Cl_2) λ/nm (log(ε/$M^{-1}\cdot cm^{-1}$)) 354 (4.56), 420 (5.66), 548 (4.31), 580 (3.44). ECD (CH_2Cl_2) λ/nm (Δε/$M^{-1}\cdot cm^{-1}$) 354 (+44), 420 (−122). Fluorescence ($CHCl_3$/CH_3CN, 1:1, v/v, λ_{ex} = 557 nm) λ_{em}/nm 607, 658. MS (MALDI-TOF) *m/z* calcd. for $[C_{113}H_{87}N_{11}O_{10}Zn]^+$ 1821.6,

found 1821.5. HRMS (ESI) *m/z* calcd. for [$C_{113}H_{87}N_{11}O_{10}Zn$ + H]$^+$ 1822.60071, found 1822.60619; calcd. for [$C_{113}H_{87}N_{11}O_{10}Zn$ + Na]$^+$ 1844.58265, found 1844.58629.

*Analytical data for the second eluted isomer ((–)-**Zn5b**)*

TLC (eluent: $CHCl_3/CH_3CN$, 9:1, v/v) R_f 0.63. M.p. >300 °C. ^{1}H NMR (500 MHz, $CDCl_3$) δ 8.91 (d, *J* = 4.6 Hz, 1H, H-75), 8.85 (d, *J* = 4.6 Hz, 1H, H-74), 8.75 (d, *J* = 4.7 Hz, 1H, H-79/80)*, 8.74 (d, *J* = 4.6 Hz, 1H, H-79/80)*, 8.66 (s, 2H, H-69 + H-70), 8.54 (d, *J* = 4.6 Hz, 1H, H-65/84)*, 8.53 (d, *J* = 4.7 Hz, 1H, H-65/84)*, 8.28 (d, *J* = 7.8 Hz, 1H, H-110), 8.12 (d, *J* = 7.3 Hz, 1H, H-58), 8.04 (dd, *J* = 7.3, 1.7 Hz, 1H, H-46), 8.02–7.97 (m, 1H, H-52), 7.95–7.88 (m, 2H, H-64 + H-107), 7.82 (dd, *J* = 7.5, 1.2 Hz, 1H, H-104), 7.80–7.70 (m, 4H, H-50 + H-56 + H-62 + H-88), 7.58 (s, 1H, H-85), 7.46 (d, *J* = 8.1 Hz, 1H, H-101), 7.43 (td, *J* = 7.6, 0.9 Hz, 1H, H-108), 7.42–7.27 (m, 8H, H-44 + H-49 + H-51 + H-55 + H-57 + H-63 + H-103 + H-109), 7.26 (d, *J* = 8.2 Hz, 1H, H-61), 7.22 (ddd, *J* = 8.2, 7.3, 1.2 Hz, 1H, H-102), 7.16 (d, *J* = 7.9 Hz, 1H, H-89), 7.08 (t, *J* = 7.5 Hz, 1H, H-45), 7.00–6.82 (m, 6H, H-3 + H-4 + H-5 + H-9 + H-10 + H-11), 6.79 (d, *J* = 8.2 Hz, 1H, H-43), 6.74–6.52 (m, 4H, H-2 + H-6 + H-8 + H-12), 6.17 (s, 1H, H-25), 6.09 (s, 1H, H-28), 6.07 (s, 1H, H-31), 4.21–4.05 (m, 6H, H-16a/17a + H-18a + H-36a + H-38a + H-40a + H-93)*, 4.01 (dt, *J* = 10.1, 4.8 Hz, 1H, H-38b), 3.97–3.88 (m, 3H, H-15a + H-16a/17a + H-40b)*, 3.87–3.81 (m, 1H, H-36b), 3.77–3.71 (m, 2H, H-34a + H-35a), 3.70–3.53 (m, 4H, H-16b + H-17b + H-18b + H-39a), 3.53 (d, *J* = 16.6 Hz, 1H, H-15b), 3.41 (dt, *J* = 10.0, 4.9 Hz, 1H, H-37a), 3.36–3.24 (m, 3H, H-34b + H-37b + H-39b), 3.23–3.13 (m, 2H, H-33a + H-35b), 3.08 (d, *J* = 15.2 Hz, 1H, H-94a), 2.98 (ddd, *J* = 10.0, 7.7, 5.8 Hz, 1H, H-33b), 2.87 (dd, *J* = 15.2, 5.6 Hz, 1H, H-94b), 2.37 (s, 3H, H-96), 0.86 (s, 9H, H-98). *Note: proton signals marked with an asterisk (*) could not be assigned unambiguously.* ^{13}C NMR (126 MHz, $CDCl_3$) δ 159.16 (C-48), 159.05 (C-42/54)*, 159.01 (C-42/54)*, 158.86 (C-60), 156.91 (C-20), 156.45 (C-19), 151.16 (C-92), 150.71 (C-68/71)*, 150.48 (C-66/83)*, 150.33 (C-66/83)*, 150.28 (C-78 + C-81), 149.97 (C-24), 149.82 (C-68/71)*, 149.72 (C-73 + C-76), 147.24 (C-30), 146.23 (C-29), 145.33 (C-86), 144.66 (C-95), 143.41 (C-91), 142.37 (C-23), 140.88 (C-106), 139.62 (C-111), 139.51 (C-105), 138.43 (C-100), 135.79 (C-64), 135.70 (C-52), 135.52 (C-58), 135.24 (C-90), 134.75 (C-46), 133.43 (C-1/7)*, 133.36 (C-1/7)*, 132.88 (C-47/53)*, 132.80 (C-47/53)*, 132.63 (C-59), 132.55 (C-26), 131.91 (C-41), 131.59 (C-79 + C-80), 131.56 (C-69/70)*, 131.38 (C-65/84)*, 131.35 (C-65/84)*, 131.14 (C-99), 131.05 (C-69/70)*, 130.83 (C-74 + C-75), 130.75 (C-32), 129.98 (C-22), 129.63 (C-62), 129.57 (C-44 + C-50 + C-56), 129.38 (C-27), 128.87 (C-3/4/5/9/10/11)*, 128.78 (2× C-3/4/5/9/10/11)*, 128.72 (2× C-3/4/5/9/10/11)*, 128.64 (C-89), 128.59 (C-3/4/5/9/10/11)*, 128.30 (C-2/6/8/12)*, 128.23 (C-2/6/8/12)*, 127.98 (C-2/6/8/12)*, 127.82 (C-2/6/8/12)*, 127.54 (C-88), 127.35 (C-103), 127.22 (C-108), 127.04 (C-102), 126.95 (C-21), 126.44 (C-109), 125.54 (C-85), 125.08 (C-110), 124.30 (C-87), 124.24 (C-101), 120.21 (C-51), 120.17 (C-57), 120.01 (C-45), 119.94 (C-63), 119.85 (C-107), 119.13 (C-104), 116.96 (C-28), 116.30 (C-67/72/77/82)*, 116.28 (C-67/72/77/82)*, 116.18 (C-67/72/77/82)*, 116.12 (C-67/72/77/82)*, 115.15 (C-25), 114.52 (C-31), 112.72 (C-49/55)*, 112.66 (C-49/55)*, 112.25 (C-61), 112.14 (C-43), 84.88 (C-14), 84.48 (C-13), 71.63 (C-33), 68.24 (C-37), 67.74 (C-36), 67.49 (C-34), 67.33 (C-40), 67.25 (C-39), 67.23 (C-38), 67.18 (C-35), 58.66 (C-93), 44.65 (C-16/17)*, 44.34 (C-16/17 + C-18)*, 38.69 (C-15), 37.13 (C-94), 35.84 (C-97), 29.23 (C-98), 20.93 (C-96). *Note: carbon signals marked with an asterisk (*) could not be assigned unambiguously.* UV-Vis (CH_2Cl_2) λ/nm (log(ε/$M^{-1}\cdot cm^{-1}$)) 354 (4.56), 421 (5.67), 548 (4.32), 582 (3.51). ECD (CH_2Cl_2) λ/nm (Δε/$M^{-1}\cdot cm^{-1}$) 354 (−25), 420 (−103). Fluorescence ($CHCl_3/CH_3CN$, 1:1, v/v, λ_{ex} = 557 nm) λ_{em}/nm 607, 658. MS (MALDI-TOF) *m/z* calcd. for [$C_{113}H_{87}N_{11}O_{10}Zn$]$^+$ 1821.6, found 1821.7. HRMS (ESI) *m/z* calcd. for [$C_{113}H_{87}N_{11}O_{10}Zn$ + H]$^+$ 1822.60071, found 1822.60524; calcd. for [$C_{113}H_{87}N_{11}O_{10}Zn$ + Na]$^+$ 1844.58265, found 1844.59622.

*Analytical data for the third eluted isomer ((+)-**Zn5b**)*

ECD (CH_2Cl_2) λ/nm (Δε/$M^{-1}\cdot cm^{-1}$) 353 (+25), 421 (+113). Other analytical data were in agreement with those obtained for (−)-**Zn5b**.

*Analytical data for the fourth eluted isomer ((+)-**Zn5a**)*
ECD (CH_2Cl_2) λ/nm (Δε/$M^{-1}\cdot cm^{-1}$) 354 (−43), 420 (+136). Other analytical data were in agreement with those obtained for (−)-**Zn5a**.

1.2 Synthesis of compound Zn6

The helicene-functionalized porphyrin cages **Zn6a** and **Zn6b** were prepared by the CuAAC reaction of mono-azide (Zn(II) porphyrin cage **Zn20**[S6] and enantiopure ethynyl-appended helicenes (*P*)-**21** and (*M*)-**21**, obtained by chiral HPLC on Chiralpak IB according to reported procedures [S7] (Scheme S2).

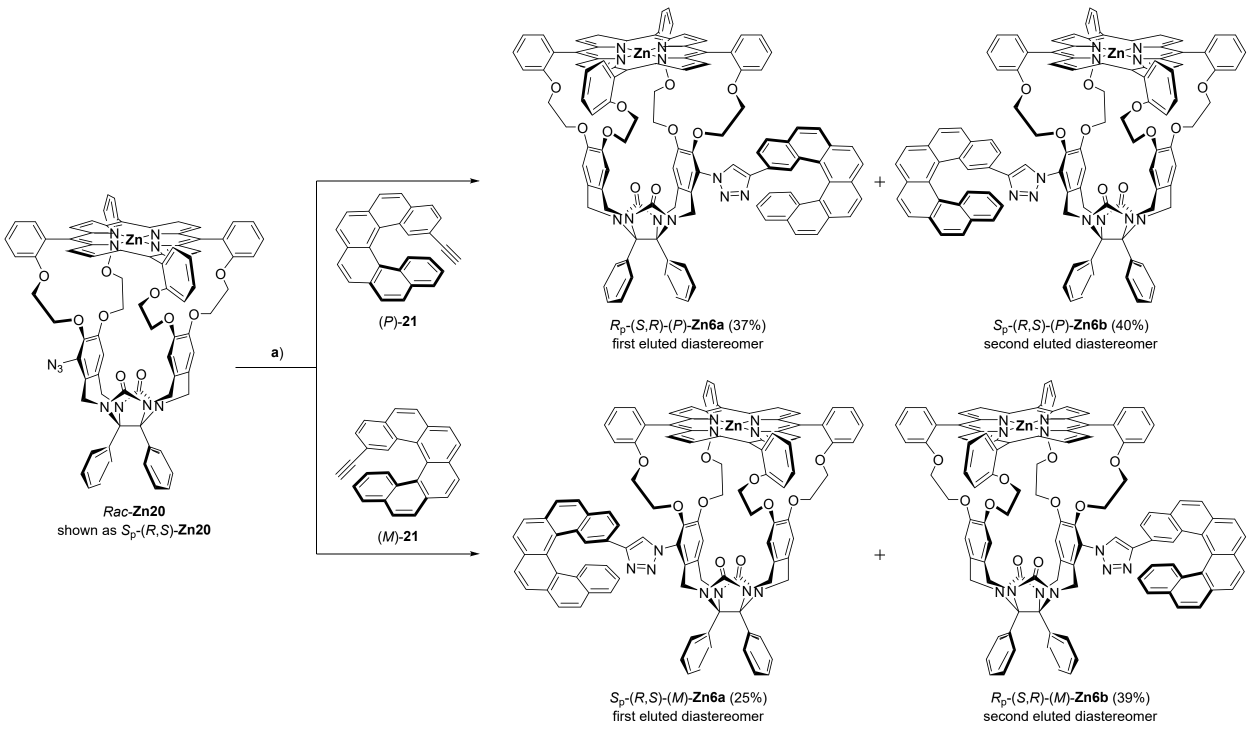

Scheme S2. Synthesis of mono-[6]-helicene functionalized porphyrin cages **Zn6a** and **Zn6b**. Reagents and conditions; a) CuI, DIPEA, sodium ascorbate, DMSO/CH_2Cl_2/H_2O, 20 °C. The descriptors *R*p and *S*p indicate the planar chiralities of the porphyrin cages. The descriptors (*R*) and (*S*) denote the central chiralities of the quaternary carbon atoms of the porphyrin cages. The descriptors (*P*) and (*M*) refer to the helical chirality of the [6]-helicene moieties.

*R_p-(S,R)-(P)-**Zn6a** and S_p-(R,S)-(P)-**Zn6b***

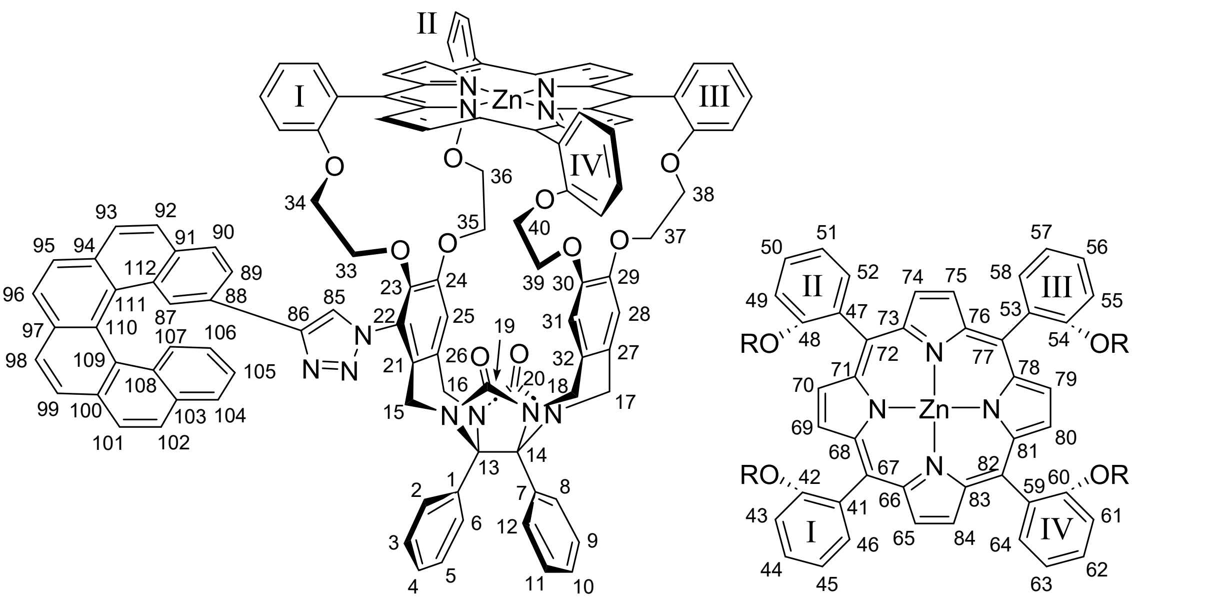

Zn6a (diastereomer 1): R_p-(*S*,*R*)-(*P*)-**Zn6a** or S_p-(*R*,*S*)-(*M*)-**Zn6a**
Zn6b (diastereomer 2): S_p-(*R*,*S*)-(*P*)-**Zn6b** or R_p-(*S*,*R*)-(*M*)-**Zn6b**

Sodium ascorbate (0.11 g, 0.57 mmol, 10 equiv.), copper(I) iodide (22 mg, 0.11 mmol, 2.0 equiv.) and DIPEA (99 µL, 0.57 mmol, 10 equiv.) were successively added to a solution of azide cage *Rac*-**Zn20** (82 mg, 57 µmol, 1.0 equiv.) and helicene alkyne (*P*)-**21** (26 mg, 74 µmol, 1.3 equiv.) in a deoxygenated solvent mixture of DMSO/CH_2Cl_2/water (25 mL, 16:4:1, v/v/v). The resulting purple suspension was stirred at 20 °C for 70 hours under an argon atmosphere. Water (50 mL) was added and the product was extracted with $CHCl_3$ (3 × 50 mL). The combined organic extracts were washed with water (2 × 50 mL) and brine (50 mL), dried over Na_2SO_4, and the solvent was removed in vacuo. The residual purple solid was purified by 60H silica gel column chromatography (eluent: $CHCl_3$/CH_3CN, 12:1, v/v). The first purple fraction contained trace amounts of unreacted azide cage *Rac*-**Zn20** and was discarded. The second purple fraction was evaporated to dryness, dissolved in a minimal amount of CH_2Cl_2 (5 mL) and precipitated by the addition of *n*-heptane (10 mL). Most CH_2Cl_2 was removed under reduced pressure and the resulting purple suspension was centrifuged. The supernatant was removed, and the precipitate was washed with *n*-pentane (3 × 10 mL) and then dried under high vacuum to afford R_p-(*S*,*R*)-(*P*)-**Zn6a** (first eluted diastereomer, 38.5 mg, 37%) as a purple solid. Accordingly, the third purple fraction was precipitated from CH_2Cl_2/*n*-heptane to afford S_p-(*R*,*S*)-(*P*)-**Zn6b** (second eluted diastereomer, 41.6 mg, 40%) as a purple solid.

*S_p-(R,S)-(M)-**Zn6a** and R_p-(S,R)-(M)-**Zn6b***

According to the procedure for R_p-(*S*,*R*)-(*P*)-**Zn6a** and S_p-(*R*,*S*)-(*P*)-**Zn6b**, the reaction of azide cage *Rac*-Zn20 (133 mg, 92 µmol, 1.0 equiv.), helicene alkyne (*M*)-**21** (32 mg, 91 µmol, 1.0 equiv.), sodium ascorbate (0.18 g, 0.91 mmol, 10 equiv.), copper(I) iodide (35 mg, 0.18 mmol, 2.0 equiv.) and DIPEA (0.16 mL, 0.91 mmol, 10 equiv.) in deoxygenated DMSO/CH_2Cl_2/water (42 mL, 16:4:1, v/v/v) afforded S_p-(*R*,*S*)-(*M*)-**Zn6a** (first eluted diastereomer, 41.7 mg, 25%) and R_p-(*S*,*R*)-(*M*)-**Zn6b** (second eluted diastereomer, 63.7 mg, 39%), both as purple solids. Analytical data for R_p-(*S*,*R*)-(*P*)-**Zn6a**:

TLC (eluent: $CHCl_3$/CH_3CN, 9:1, v/v) R_f 0.62. M.p. >300 °C. ^1H NMR (500 MHz, c = 10^{-3} M in $CDCl_3$) δ 9.32 (d, *J* = 4.7 Hz, 1H, H-65), 8.95 (d, *J* = 4.7 Hz, 2H, H-75 + H-84), 8.91 (d, *J* = 4.7 Hz, 1H, H-74), 8.84

(d, *J* = 4.6 Hz, 1H, H-79/80)*, 8.82 (d, *J* = 4.6 Hz, 1H, H-79/80)*, 8.80 (d, *J* = 4.5 Hz, 1H, H-69), 8.76 (d, *J* = 4.5 Hz, 1H, H-70), 8.53 (dd, *J* = 7.1, 1.8 Hz, 1H, H-46), 8.19 (dd, *J* = 7.3, 1.6 Hz, 1H, H-58), 8.11 (dd, *J* = 7.4, 1.6 Hz, 1H, H-64), 8.07 (dd, *J* = 7.5, 1.3 Hz, 1H, H-52), 8.05 (d, *J* = 8.4 Hz, 1H, H-96), 8.02 (d, *J* = 8.4 Hz, 1H, H-95), 8.02 (d, *J* = 1.6 Hz, 1H, H-87), 7.97 (d, *J* = 8.6 Hz, 1H, H-93), 7.95 (d, *J* = 8.0 Hz, 1H, H-98), 7.94 (d, *J* = 8.6 Hz, 1H, H-92), 7.81 (d, *J* = 8.1 Hz, 1H, H-90), 7.82–7.75 (m, 2H, H-50 + H-56), 7.74–7.68 (m, 1H, H-62), 7.70 (d, *J* = 8.3 Hz, 1H, H-107), 7.68–7.64 (m, 2H, H-99 + H-104), 7.61 (td, *J* = 7.8, 2.0 Hz, 1H, H-44), 7.60–7.54 (m, 1H, H-45), 7.50 (dd, *J* = 8.0, 1.6 Hz, 1H, H-89), 7.46 (d, *J* = 8.7 Hz, 1H, H-101), 7.46–7.41 (m, 1H, H-57), 7.42–7.34 (m, 4H, H-49 + H-51 + H-55 + H-63), 7.27 (d, *J* = 8.7 Hz, 1H, H-102), 7.27–7.22 (m, 2H, H-61 + H-105), 7.05–7.00 (m, 1H, H-5), 7.00–6.96 (m, 1H, H-11), 6.95 (s, 1H, H-85), 6.96–6.91 (m, 2H, H-4 + H-10), 6.91–6.86 (m, 1H, H-9), 6.85–6.79 (m, 4H, H-3 + H-6 + H-12 + H-106), 6.78 (d, *J* = 8.2 Hz, 1H, H-43), 6.70 (d, *J* = 8.0 Hz, 1H, H-8), 6.56 (d, *J* = 8.1 Hz, 1H, H-2), 6.31 (s, 1H, H-28), 6.28 (s, 1H, H-25), 6.16 (s, 1H, H-31), 4.30 (ddd, *J* = 10.8, 6.6, 4.1 Hz, 1H, H-38a), 4.29 (d, *J* = 15.1 Hz, 1H, H-16a), 4.23 (d, *J* = 15.9 Hz, 1H, H-18a), 4.21–4.15 (m, 1H, H-40a), 4.15 (d, *J* = 15.5 Hz, 1H, H-17a), 4.15–4.08 (m, 2H, H-36a + H-38b), 4.05–3.99 (m, 1H, H-36b), 3.95–3.89 (m, 1H, H-40b), 3.76 (d, *J* = 15.9 Hz, 1H, H-18b), 3.75 (d, *J* = 15.4 Hz, 1H, H-16b), 3.75–3.69 (m, 2H, H-34a + H-35a), 3.72 (d, *J* = 15.5 Hz, 1H, H-17b), 3.65–3.50 (m, 3H, H-37a + H-37b + H-39a), 3.60 (d, *J* = 16.8 Hz, 1H, H-15a), 3.50 (d, *J* = 16.7 Hz, 1H, H-15b), 3.33 (ddd, *J* = 10.8, 8.4, 2.8 Hz, 1H, H-35b), 3.28 (ddd, *J* = 10.4, 8.4, 2.3 Hz, 1H, H-39b), 3.26–3.18 (m, 2H, H-33a + H-34b), 2.75–2.69 (m, 1H, H-33b). Note: proton signals marked with an asterisk (*) could not be assigned unambiguously. ^{13}C NMR (126 MHz, c = 10^{-2} M in $CDCl_3$) δ 159.16 (C-42/48/54/60)*, 159.14 (C-42/48/54/60)*, 158.98 (C-42/48/54/60)*, 158.82 (C-42/48/54/60)*, 156.97 (C-20), 156.33 (C-19), 150.91 (C-68/71)*, 150.46 (C-78/81)*, 150.43 (C-78/81)*, 150.27 (C-66/73/76/83)*, 150.13 (C-66/73/76/83)*, 150.03 (C-68/71)*, 150.00 (C-24), 149.94 (C-66/73/76/83)*, 149.88 (C-66/73/76/83)*, 147.01 (C-30), 146.69 (C-86), 145.96 (C-29), 141.68 (C-23), 135.71 (C-52 + C-58), 135.66 (C-64), 134.40 (C-46), 133.15 (C-1/7)*, 133.09 (C-1/7)*, 133.07 (C-97), 133.01 (C-47/59)*, 132.87 (C-47/59)*, 132.50 (C-26), 132.39 (C-41), 132.28 (C-103), 132.23 (C-53), 131.76 (C-69/79/80)*, 131.69 (C-91), 131.67 (C-69/79/80)*, 131.65 (C-69/79/80)*, 131.50 (C-70 + C-84), 131.41 (C-94), 131.24 (C-65), 131.11 (C-100), 131.07 (C-74), 130.97 (C-75), 130.61 (C-32), 129.98 (C-44), 129.93 (C-112), 129.85 (C-108), 129.76 (C-22), 129.66 (C-50/56)*, 129.63 (C-50/56)*, 129.56 (C-62), 129.31 (C-27), 128.87 (C-3/4/9/10)*, 128.79 (2× C-3/4/9/10)*, 128.73 (C-3/4/9/10)*, 128.65 (C-5/11)*, 128.52 (C-5/11)*, 128.38 (C-2), 128.26 (C-8), 128.24 (C-111), 128.17 (C-99/104)*, 128.09 (C-90), 127.92 (C-109), 127.87 (C-6/12)*, 127.77 (C-92), 127.59 (C-6/12 + C-99/104)*, 127.41 (C-107), 127.30 (C-21 + C-88 + C-95), 127.16 (C-102), 127.01 (C-96), 126.62 (C-93/98)*, 126.56 (C-101), 126.40 (C-93/98)*, 125.57 (C-105), 125.40 (C-87), 124.65 (C-106), 124.44 (C-89), 124.13 (C-110), 123.36 (C-85), 120.33 (C-51/57)*, 120.18 (C-45), 120.13 (C-51/57)*, 119.92 (C-63), 116.60 (C-28), 116.46 (C-72/77/82)*, 116.31 (C-72/77/82)*, 116.21 (C-72/77/82)*, 116.05 (C-67), 115.36 (C-25), 114.18 (C-31), 113.03 (C-49), 112.43 (C-55), 112.30 (C-43), 112.11 (C-61), 84.93 (C-14), 84.44 (C-13), 71.02 (C-33), 67.94 (C-37), 67.86 (C-36), 67.54 (C-35), 67.17 (C-40), 67.11 (C-39), 66.92 (C-38), 65.82 (C-34), 44.58 (C-16/17)*, 44.45 (C-16/17)*, 44.32 (C-18), 38.82 (C-15). Note: carbon signals marked with an asterisk (*) could not be assigned unambiguously. UV-Vis (CH_2Cl_2) λ_{max}/nm (log(ε/M^{-1}·cm^{-1})) 272 (4.85), 320 (4.55), 420 (5.64), 548 (4.26). ECD (CH_2Cl_2) λ_{max}/nm (Δε/M^{-1}·cm^{-1}) 264 (−150), 335 (+215), 421 (−58). Fluorescence ($CHCl_3$/CH_3CN, 1:1, v/v, λ_{ex} = 420 nm) λ_{em}/nm 608, 658. HRMS (ESI) *m/z* calcd. for $[C_{112}H_{77}N_{11}O_{10}Zn + H]^+$ 1800.5224, found 1800.5208; calcd. for $[C_{112}H_{77}N_{11}O_{10}Zn + Na]^+$ 1822.5044, found 1822.5053.

Analytical data for S_p*-(R,S)-(M)-**Zn6a***

ECD (CH_2Cl_2) λ_{max}/nm (Δε/M^{-1}·cm^{-1}) 264 (+153), 335 (−221), 421 (+48). Other analytical data were in agreement with those obtained for R_p-(*S*,*R*)-(*P*)-**Zn6a**.

*Analytical data for S_p-(R,S)-(P)-**Zn6b***

TLC (eluent: $CHCl_3/CH_3CN$, 9:1, v/v) R_f 0.57. M.p. >300 °C. ^{1}H NMR (500 MHz, c = 10^{-3} M in $CDCl_3$) δ 8.94 (d, *J* = 4.6 Hz, 1H, H-75), 8.92 (d, *J* = 4.6 Hz, 1H, H-74), 8.83 (d, *J* = 4.6 Hz, 1H, H-79/80)*, 8.82 (d, *J* = 4.6 Hz, 1H, H-79/80)*, 8.80 (d, *J* = 4.6 Hz, 1H, H-65), 8.78 (d, *J* = 4.5 Hz, 1H, H-69/70)*, 8.76 (d, *J* = 4.6 Hz, 1H, H-69/70)*, 8.61 (d, *J* = 4.6 Hz, 1H, H-84), 8.31 (dd, *J* = 7.3, 1.6 Hz, 1H, H-46), 8.13 (dd, *J* = 7.5, 1.6 Hz, 1H, H-58), 8.10 (dd, *J* = 7.6, 1.6 Hz, 1H, H-52), 8.02 (dd, *J* = 7.3, 1.7 Hz, 1H, H-64), 8.01 (d, *J* = 8.3 Hz, 1H, H-95), 7.98 (d, *J* = 8.2 Hz, 2H, H-96 + H-98), 7.92 (s, 2H, H-92 + H-93), 7.90 (d, *J* = 8.2 Hz, 1H, H-99), 7.89–7.85 (m, 2H, H-89 + H-90), 7.85–7.83 (m, 1H, H-87), 7.82–7.75 (m, 2H, H-50 + H-56), 7.70–7.63 (m, 2H, H-44 + H-62), 7.55 (d, *J* = 8.6 Hz, 1H, H-107), 7.50 (d, *J* = 8.6 Hz, 1H, H-101), 7.45–7.38 (m, 5H, H-45 + H-49 + H-51 + H-55 + H-57), 7.33 (d, *J* = 8.3 Hz, 1H, H-61), 7.31–7.26 (m, 1H, H-63), 7.11 (s, 1H, H-85), 7.08 (d, *J* = 8.3 Hz, 1H, H-43), 7.02 (d, *J* = 7.6 Hz, 1H, H-104), 7.02–6.97 (m, 1H, H-5), 6.92–6.83 (m, 4H, H-4 + H-9 + H-10 + H-11), 6.80 (d, *J* = 7.9 Hz, 1H, H-6), 6.77–6.64 (m, 5H, H-3 + H-8 + H-12 + H-102 + H-105), 6.57 (ddd, *J* = 8.3, 6.7, 1.4 Hz, 1H, H-106), 6.46 (d, *J* = 8.3 Hz, 1H, H-2), 6.37 (s, 1H, H-31), 6.35 (s, 1H, H-28), 6.27 (s, 1H, H-25), 4.40–4.29 (m, 3H, H-38a + H-40a + H-40b), 4.27 (d, *J* = 15.8 Hz, 1H, H-16a), 4.17 (d, *J* = 15.8 Hz, 1H, H-18a), 4.17–4.12 (m, 2H, H-17a + H-38b), 4.00 (ddd, *J* = 10.9, 7.9, 2.8 Hz, 1H, H-36a), 3.94 (ddd, *J* = 9.7, 5.5, 2.6 Hz, 1H, H-39a), 3.90–3.83 (m, 2H, H-34a + H-36b), 3.76–3.65 (m, 5H, H-16b + H-17b + H-18b + H-35a + H-37a), 3.62 (ddd, *J* = 9.7, 6.6, 2.7 Hz, 1H, H-39b), 3.53 (ddd, *J* = 10.9, 7.4, 3.8 Hz, 1H, H-37b), 3.48–3.41 (m, 1H, H-34b), 3.37 (d, *J* = 16.5 Hz, 1H, H-15a), 3.30 (d, *J* = 16.7 Hz, 1H, H-15b), 3.31–3.25 (m, 1H, H-35b), 3.22 (ddd, *J* = 9.4, 7.3, 4.3 Hz, 1H, H-33a), 2.93–2.86 (m, 1H, H-33b). Note: proton signals marked with an asterisk (*) could not be assigned unambiguously. ^{13}C NMR (126 MHz, c = 10^{-2} M in $CDCl_3$) δ 159.30 (C-42/48/54/60)*, 159.03 (C-42/48/54/60)*, 158.94 (C-42/48/54/60)*, 158.90 (C-42/48/54/60)*, 156.97 (C-20), 156.29 (C-19), 150.93 (C-68/71)*, 150.55 (C-78/81)*, 150.48 (C-78/81)*, 150.36 (C-24/66/83)*, 150.26 (C-24/66/83)*, 150.20 (C-24/66/83)*, 149.95 (C-73/76)*, 149.90 (C-68/71)*, 149.81 (C-73/76)*, 146.90 (C-30), 146.45 (C-29), 146.36 (C-86), 142.26 (C-23), 136.03 (C-64), 135.93 (C-58), 135.80 (C-52), 134.79 (C-46), 133.25 (C-1/7)*, 133.19 (C-1/7)*, 133.12 (C-47/53)*, 132.89 (C-97), 132.84 (C-47/53)*, 132.52 (C-59), 132.39 (C-26), 132.24 (C-41), 131.94 (C-103), 131.76 (C-69/70/79/80)*, 131.71 (C-91), 131.68 (C-69/70/79/80)*, 131.65 (C-84), 131.57 (C-69/70/79/80 + C-94)*, 131.49 (C-65), 131.39 (C-69/70/79/80)*, 131.36 (C-100), 130.92 (C-74/75)*, 130.90 (C-74/75)*, 130.77 (C-32), 130.18 (C-22), 130.05 (C-112), 129.79 (C-27 + C-44/50/56/62)*, 129.61 (2× C-44/50/56/62)*, 129.56 (C-44/50/56/62)*, 129.38 (C-108), 128.83 C-4/9/10/11)*, 128.73 (2× C-4/9/10/11)*, 128.69 (C-4/9/10/11)*, 128.61 (C-3), 128.47 (C-5), 128.32 (C-2 + C-102)*, 128.25 (C-8/12)*, 128.10 (C-90 + C-111), 127.85 (C-8/12), 127.76 (C-92/93)*, 127.69 (C-109), 127.55 (C-6), 127.51 (C-104), 127.30 (C-88), 127.20 (C-96/98)*, 127.15 (C-107), 127.07 (C-95), 127.05 (C-21), 126.96 (C-99), 126.75 (C-96/98)*, 126.36 (C-92/93)*, 125.75 (C-105), 125.63 (C-101), 125.19 (C-87), 124.56 (C-106), 124.42 (C-110), 123.77 (C-89), 123.34 (C-85), 120.46 (C-45/51/57/63)*, 120.15 (C-45/51/57/63)*, 120.09 (C-45/51/57/63)*, 120.07 (C-45/51/57/63)*, 116.52 (C-28 + C-67/72/77/82)*, 116.27 (C-67/72/77/82)*, 116.23 (C-67/72/77/82)*, 116.21 (C-67/72/77/82)*, 116.15 (C-31), 115.10 (C-25), 113.64 (C-49/55)*, 112.40 (C-49/55)*, 112.17 (C-61), 111.66 (C-43), 84.91 (C-14), 84.47 (C-13), 70.97 (C-33), 68.48 (C-39), 68.12 (C-36), 68.00 (C-40), 67.80 (C-37), 67.49 (C-35), 67.14 (C-38), 66.18 (C-34), 44.68 (C-17), 44.43 (C-16), 44.01 (C-18), 38.75 (C-15). Note: carbon signals marked with an asterisk (*) could not be assigned unambiguously. UV-Vis (CH_2Cl_2) λ_{max}/nm (log(ε/$M^{-1}\cdot cm^{-1}$)) 274 (4.86), 321 (4.56), 420 (5.65), 548 (4.27). ECD (CH_2Cl_2) λ_{max}/nm (Δε/$M^{-1}\cdot cm^{-1}$) 264 (−167), 335 (+233), 413 (+6), 422 (−9). Fluorescence ($CHCl_3/CH_3CN$, 1:1, v/v, λ_{ex} = 420 nm) λ_{em}/nm 608, 658. HRMS (ESI) *m/z* calcd. for $[C_{112}H_{77}N_{11}O_{10}Zn + H]^+$ 1800.5224, found 1800.5261; calcd. for $[C_{112}H_{77}N_{11}O_{10}Zn + Na]^+$ 1822.5044, found 1822.5032.

*Analytical data for R_p-(S,R)-(M)-**Zn6b***

ECD (CH_2Cl_2) λ_{max}/nm ($\Delta\varepsilon$/$M^{-1}\cdot cm^{-1}$) 264 (+164), 335 (−234), 413 (−7), 422 (+7). Other analytical data were in agreement with those obtained for S_p-(*R*,*S*)-(*P*)-**Zn6b**.

2. Threading

2.1 Threading procedure

A solution of one of the hosts **1**–**6** (2 mL, c = 10^{-5} M in $CHCl_3/CH_3CN$, 1:1, v/v, 298 K) was added to a 1 cm quartz cuvette and the cuvette was placed inside the fluorescence spectrometer. The fluorescence intensity of the host (λ_{ex} = 512 nm, λ_{em} = 647 nm for the free base porphyrin cages; λ_{ex} = 555 nm, λ_{em} = 657 nm for the zinc(II) porphyrin cages) was monitored as a function of time (10 data points per second). At t ≈ 40s, the cuvette was removed from the spectrometer, and at t ≈ 50s a solution of guest 7 (10 µL, c = 2 × 10^{-3} M in $CHCl_3/CH_3CN$, 1:1, v/v, 298 K, 1 equiv.) was added to the solution containing the host. The contents of the cuvette were shaken vigorously for 5 seconds. At t ≈ 60 s, the cuvette was placed back inside the spectrometer. At t = 600 s, the experiment was stopped (for examples see Figure S1A). The fluorescence intensity of the host was normalized to the average fluorescence intensity during the initial 40 seconds. The inverse concentration of the host during the initial part (up to 50%) of the threading process was plotted as a function of time. By fitting the resulting data points to a linear function, the slope (= k_{on}) was determined (for examples see Figure S1B). The experiment was repeated two more times and the averages of the rate constants are presented in Table 1.3. Details of the individual experiments, such as non-averaged rate constants and fitting parameters, are provided in section 1.4, Table S1 (free base porphyrin cages) and Table S2 (zinc(II) porphyrin cages) of the Supporting Information.

2.2. Typical threading experiments

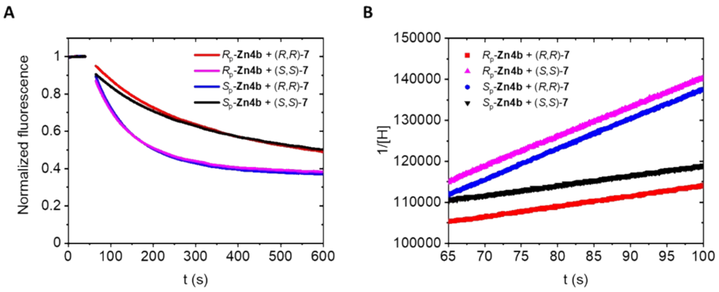

Figure S1. Typical threading experiments employing all combinations of enantiomeric hosts **Zn4b** and enantiomeric guests **7**. (A) Normalized fluorescence intensity of the host as a function of time after the addition (t ≈ 50 s) of 1 equivalent of the guest (c = 10^{-5} M in $CHCl_3/CH_3CN$, 1:1, v/v, 298 K, λ_{ex} = 555 nm, λ_{em} = 657 nm). (B) Corresponding second-order kinetics plots.

2.3. Tables with kinetic threading data

Table S1. Kinetic data for the threading of free base porphyrin cages onto the enantiomers of guest **7**. Data were acquired by fluorescence spectroscopy at 298 K (c = 10^{-5} M in $CHCl_3/CH_3CN$, 1:1, v/v).

**Host**	**Guest**	k_{on} **(× 10^3 $M^{-1}·s^{-1}$)**	**R^2**
R_p-**$H_2$2**	(*R*,*R*)-**7**	3.77	0.999
R_p-**$H_2$2**	(*R*,*R*)-**7**	3.78	0.999
R_p-**$H_2$2**	(*R*,*R*)-**7**	3.72	0.999
R_p-**$H_2$2**	(*S*,*S*)-**7**	3.64	0.999
R_p-**$H_2$2**	(*S*,*S*)-**7**	3.92	0.999
R_p-**$H_2$2**	(*S*,*S*)-**7**	3.97	0.999
S_p-**$H_2$2**	(*R*,*R*)-**7**	3.90	0.999
S_p-**$H_2$2**	(*R*,*R*)-**7**	3.67	0.999
S_p-**$H_2$2**	(*R*,*R*)-**7**	3.61	0.999
S_p-**$H_2$2**	(*S*,*S*)-**7**	3.47	0.999
S_p-**$H_2$2**	(*S*,*S*)-**7**	3.43	0.999
S_p-**$H_2$2**	(*S*,*S*)-**7**	3.38	0.999
R_p-**$H_2$3a**	(*R*,*R*)-**7**	2.00	0.998
R_p-**$H_2$3a**	(*R*,*R*)-**7**	2.09	0.999
R_p-**$H_2$3a**	(*R*,*R*)-**7**	2.03	0.998
R_p-**$H_2$3a**	(*S*,*S*)-**7**	2.97	0.997
R_p-**$H_2$3a**	(*S*,*S*)-**7**	3.02	0.998
R_p-**$H_2$3a**	(*S*,*S*)-**7**	3.01	0.998
S_p-**$H_2$3a**	(*R*,*R*)-**7**	3.50	0.999
S_p-**$H_2$3a**	(*R*,*R*)-**7**	3.65	0.998
S_p-**$H_2$3a**	(*R*,*R*)-**7**	3.68	0.998
S_p-**$H_2$3a**	(*S*,*S*)-**7**	2.40	0.999
S_p-**$H_2$3a**	(*S*,*S*)-**7**	2.36	0.999
S_p-**$H_2$3a**	(*S*,*S*)-**7**	2.25	0.999
R_p-**$H_2$3b**	(*R*,*R*)-**7**	1.29	0.999
R_p-**$H_2$3b**	(*R*,*R*)-**7**	1.57	0.999
R_p-**$H_2$3b**	(*R*,*R*)-**7**	1.47	0.999
R_p-**$H_2$3b**	(*S*,*S*)-**7**	1.87	0.999
R_p-**$H_2$3b**	(*S*,*S*)-**7**	1.96	0.999
R_p-**$H_2$3b**	(*S*,*S*)-**7**	1.83	0.999
S_p-**$H_2$3b**	(*R*,*R*)-**7**	1.96	0.999
S_p-**$H_2$3b**	(*R*,*R*)-**7**	1.86	0.999
S_p-**$H_2$3b**	(*R*,*R*)-**7**	1.84	0.999
S_p-**$H_2$3b**	(*S*,*S*)-**7**	1.38	0.998
S_p-**$H_2$3b**	(*S*,*S*)-**7**	1.60	0.998
S_p-**$H_2$3b**	(*S*,*S*)-**7**	1.63	0.999

Table S2. Kinetic data for the threading of zinc(II) porphyrin cages onto the enantiomers of guest **7**. Data were acquired by fluorescence spectroscopy at 298 K (c = 10^{-5} M in $CHCl_3/CH_3CN$, 1:1, v/v).

**Host**	**Guest**	k_{on} **(× 10^2 $M^{-1}·s^{-1}$)**	**R^2**
**Zn1**	(*R*,*R*)-**7**	3.88	0.999
**Zn1**	(*R*,*R*)-**7**	4.11	0.999
**Zn1**	(*R*,*R*)-**7**	4.19	0.999
**Zn1**	(*S*,*S*)-**7**	4.12	0.999
**Zn1**	(*S*,*S*)-**7**	4.09	0.999

**Zn1**	(*S*,*S*)-**7**	4.37	0.999
R_p-**Zn2**	(*R*,*R*)-**7**	19.3	0.999
R_p-**Zn2**	(*R*,*R*)-**7**	19.0	0.999
R_p-**Zn2**	(*R*,*R*)-**7**	19.1	0.999
R_p-**Zn2**	(*S*,*S*)-**7**	14.4	0.999
R_p-**Zn2**	(*S*,*S*)-**7**	13.9	0.999
R_p-**Zn2**	(*S*,*S*)-**7**	15.9	0.999
S_p-**Zn2**	(*R*,*R*)-**7**	13.2	0.999
S_p-**Zn2**	(*R*,*R*)-**7**	13.7	0.999
S_p-**Zn2**	(*R*,*R*)-**7**	14.1	0.999
S_p-**Zn2**	(*S*,*S*)-**7**	18.3	0.999
S_p-**Zn2**	(*S*,*S*)-**7**	16.6	0.999
S_p-**Zn2**	(*S*,*S*)-**7**	19.3	0.999
R_p-**Zn4a**	(*R*,*R*)-**7**	3.53	0.999
R_p-**Zn4a**	(*R*,*R*)-**7**	3.75	0.999
R_p-**Zn4a**	(*R*,*R*)-**7**	3.44	0.999
R_p-**Zn4a**	(*S*,*S*)-**7**	10.6	0.999
R_p-**Zn4a**	(*S*,*S*)-**7**	10.8	0.999
R_p-**Zn4a**	(*S*,*S*)-**7**	10.0	0.999
S_p-**Zn4a**	(*R*,*R*)-**7**	10.8	0.999
S_p-**Zn4a**	(*R*,*R*)-**7**	10.3	0.999
S_p-**Zn4a**	(*R*,*R*)-**7**	10.4	0.999
S_p-**Zn4a**	(*S*,*S*)-**7**	3.31	0.999
S_p-**Zn4a**	(*S*,*S*)-**7**	3.16	0.999
S_p-**Zn4a**	(*S*,*S*)-**7**	3.18	0.999
R_p-**Zn4b**	(*R*,*R*)-**7**	2.44	0.999
R_p-**Zn4b**	(*R*,*R*)-**7**	2.38	0.998
R_p-**Zn4b**	(*R*,*R*)-**7**	2.42	0.998
R_p-**Zn4b**	(*S*,*S*)-**7**	7.38	0.999
R_p-**Zn4b**	(*S*,*S*)-**7**	7.26	0.998
R_p-**Zn4b**	(*S*,*S*)-**7**	7.00	0.998
S_p-**Zn4b**	(*R*,*R*)-**7**	7.47	0.999
S_p-**Zn4b**	(*R*,*R*)-**7**	7.33	0.999
S_p-**Zn4b**	(*R*,*R*)-**7**	7.36	0.998
S_p-**Zn4b**	(*S*,*S*)-**7**	2.32	0.998
S_p-**Zn4b**	(*S*,*S*)-**7**	2.31	0.998
S_p-**Zn4b**	(*S*,*S*)-**7**	2.26	0.997
R_p-**Zn5a**	(*R*,*R*)-**7**	4.44	0.999
R_p-**Zn5a**	(*R*,*R*)-**7**	4.40	0.999
R_p-**Zn5a**	(*R*,*R*)-**7**	4.23	0.999
R_p-**Zn5a**	(*S*,*S*)-**7**	9.94	0.998
R_p-**Zn5a**	(*S*,*S*)-**7**	10.1	0.999
R_p-**Zn5a**	(*S*,*S*)-**7**	9.41	0.998
S_p-**Zn5a**	(*R*,*R*)-**7**	10.9	0.999
S_p-**Zn5a**	(*R*,*R*)-**7**	10.3	0.999
S_p-**Zn5a**	(*R*,*R*)-**7**	10.1	0.999
S_p-**Zn5a**	(*S*,*S*)-**7**	4.19	0.999
S_p-**Zn5a**	(*S*,*S*)-**7**	4.33	0.999
S_p-**Zn5a**	(*S*,*S*)-**7**	4.07	0.998
R_p-**Zn5b**	(*R*,*R*)-**7**	3.67	0.999
R_p-**Zn5b**	(*R*,*R*)-**7**	3.36	0.999
R_p-**Zn5b**	(*R*,*R*)-**7**	3.47	0.999
R_p-**Zn5b**	(*S*,*S*)-**7**	6.78	0.999
R_p-**Zn5b**	(*S*,*S*)-**7**	6.99	0.999
R_p-**Zn5b**	(*S*,*S*)-**7**	6.75	0.999
S_p-**Zn5b**	(*R*,*R*)-**7**	6.54	0.998

S_p-**Zn5b**	(*R*,*R*)-**7**	6.46	0.999
S_p-**Zn5b**	(*R*,*R*)-**7**	6.47	0.999
S_p-**Zn5b**	(*S*,*S*)-**7**	3.50	0.998
S_p-**Zn5b**	(*S*,*S*)-**7**	3.19	0.998
S_p-**Zn5b**	(*S*,*S*)-**7**	3.09	0.998
R_p-**Zn6a**	(*R*,*R*)-**7**	5.51	0.999
R_p-**Zn6a**	(*R*,*R*)-**7**	5.81	0.999
R_p-**Zn6a**	(*R*,*R*)-**7**	5.59	0.999
R_p-**Zn6a**	(*S*,*S*)-**7**	10.2	0.999
R_p-**Zn6a**	(*S*,*S*)-**7**	10.4	0.999
R_p-**Zn6a**	(*S*,*S*)-**7**	10.6	0.999
S_p-**Zn6a**	(*R*,*R*)-**7**	10.3	0.999
S_p-**Zn6a**	(*R*,*R*)-**7**	10.4	0.999
S_p-**Zn6a**	(*R*,*R*)-**7**	10.4	0.999
S_p-**Zn6a**	(*S*,*S*)-**7**	5.25	0.999
S_p-**Zn6a**	(*S*,*S*)-**7**	5.90	0.999
S_p-**Zn6a**	(*S*,*S*)-**7**	5.65	0.999
R_p-**Zn6b**	(*R*,*R*)-**7**	2.38	0.999
R_p-**Zn6b**	(*R*,*R*)-**7**	2.37	0.999
R_p-**Zn6b**	(*R*,*R*)-**7**	2.41	0.999
R_p-**Zn6b**	(*S*,*S*)-**7**	3.70	0.999
R_p-**Zn6b**	(*S*,*S*)-**7**	3.49	0.999
R_p-**Zn6b**	(*S*,*S*)-**7**	3.73	0.999
S_p-**Zn6b**	(*R*,*R*)-**7**	3.36	0.999
S_p-**Zn6b**	(*R*,*R*)-**7**	3.62	0.999
S_p-**Zn6b**	(*R*,*R*)-**7**	3.66	0.999
S_p-**Zn6b**	(*S*,*S*)-**7**	2.43	0.999
S_p-**Zn6b**	(*S*,*S*)-**7**	2.35	0.999
S_p-**Zn6b**	(*S*,*S*)-**7**	2.49	0.999

3. Ion mobility experiments

3.1. Ion mobility procedure

Ion mobility-mass spectrometry experiments were performed in the positive mode with a trapped ion mobility-quadrupole time-of-flight mass spectrometer (timsTOF, Bruker Daltonics, Bremen, Germany). A detailed description of the instrument can be found in the literature.[S8,S9] Ions were electro-sprayed with a source voltage of +4.5 kV, with a sprayer pressure of 0.03 MPa and a drying gas flow of 3.5 L·min^{-1}. Typical ion transfer voltages were: quadrupole ion energy = 3 eV and collision energy = 5 eV. TIMS experiments were performed in N_2 using the imeX Detect mode, by scanning ion mobility from 0.8 Vs cm^{-2} to 2.2 Vs cm^{-2}, with the accumulation time set at 0.5 ms. The MS and TIMS dimensions were calibrated quadratically using eight selected ions from the Agilent ESI LC/MS tuning mix [(622.0290, 0.9848 Vs cm^{-2}), (922.0098, 1.1896 Vs cm^{-2}) (1221.9906, 1.3820 Vs cm^{-2}), (1521.9715, 1.5558 Vs cm^{-2}), (1821.9523, 1.7286 Vs cm^{-2}), (2121.9331, 1.8842 Vs cm^{-2}), (2421.9140, 2.0298 Vs cm^{-2}), (2721.8948, 2.1682 Vs cm^{-2})]. The measured inverse mobilities $1/K_0$ were converted to CCS_{N2} according to the Mason-Schamp equation, by considering a temperature of 305K in the TIMS cell.[S10]

Table S4. Values of the collisional cross section (CCS) of the radical cations/molecular ions $[M]^+$ and of their sodium adducts $[M + Na]^+$ for different porphyrin cages as determined by IMS-MS. CCS values of the free base porphyrin cages **$H_2$1**–**$H_2$3** were normalized against **$H_2$1** and CCS values of the zinc(II) porphyrin cages **Zn1**–**Zn6** were normalized against **Zn1**.

	Radical cation/Molecular ion $[M]^+$			Sodium adduct $[M + Na]^+$		
Host	CCS (Å^2)	FWHM (Å^2)	relative CCS	CCS (Å^2)	FWHM (Å^2)	relative CCS
**$H_2$1**	370.2	3.6	**1.000** ± 0.005	367.0	2.4	**1.000** ± 0.003
S_p-**$H_2$2**	372.2	3.8	1.005 ± 0.005	368.1	2.7	1.003 ± 0.004
S_p-**$H_2$3a**	390.5	1.3	1.055 ± 0.002	382.6	1.3	1.043 ± 0.002
S_p-**$H_2$3b**	392.3	1.4	1.060 ± 0.002	383.6	1.4	1.045 ± 0.002
**Zn1**	370.2	3.4	**1.000** ± 0.005	368.8	3.9	**1.000** ± 0.005
S_p-**Zn2**	371.6	1.5	1.004 ± 0.002	368.5	2.9	0.999 ± 0.004
S_p-**Zn4a**	424.1/428.9[a]	5.2/3.0[a]	1.152 ± 0.013[a]	424.0	4.1	1.150 ± 0.006
S_p-**Zn4b**	421.5	3.6	1.138 ± 0.005	424.4	3.6	1.151 ± 0.005
S_p-**Zn5a**	422.5	2.9	1.141 ± 0.004	420.4	3.2	1.140 ± 0.004
S_p-**Zn5b**	411.7	3.1	1.112 ± 0.004	413.0	3.3	1.120 ± 0.005
S_p-**Zn6a**	403.0	4.1	1.088 ± 0.006	400.7	2.9	1.087 ± 0.004
S_p-**Zn6b**	407.9	3.4	1.102 ± 0.005	407.4	3.3	1.105 ± 0.004

[a]For the radical cation/molecular ion $[M]^+$ of compound S_p-**Zn4a** two conformers with different CCS values were detected. The reported relative CCS of S_p-**Zn4a** is the average value for these two conformers and the associated error covers the CCS values of both conformers including the corresponding FWHM values.

3.2. Table of collisional cross-sections

4. Spectra

4.1. CD Spectra

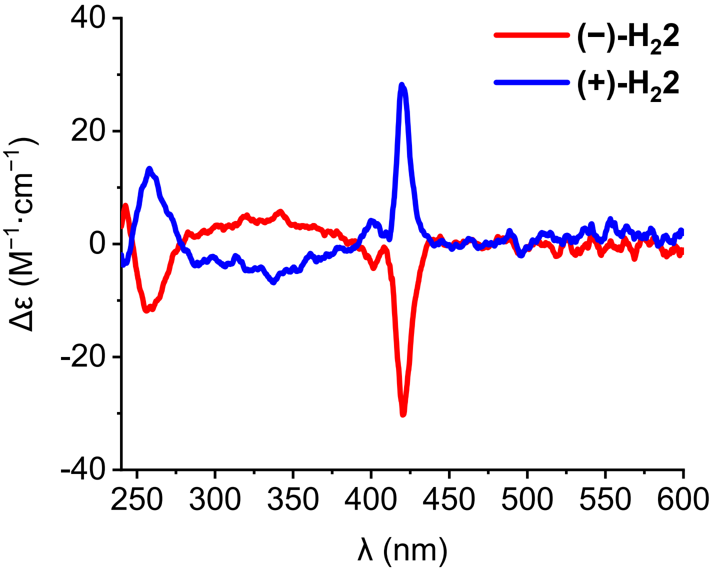

Figure S2. ECD spectra (CH_2Cl_2, 298 K) of the enantiomers of **H$_2$2**.

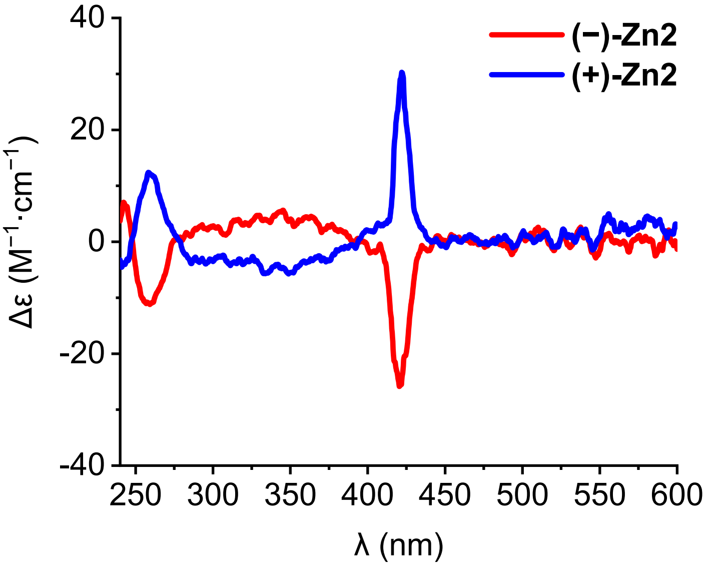

Figure S3. ECD spectra (CH_2Cl_2, 298 K) of the enantiomers of **Zn2**.

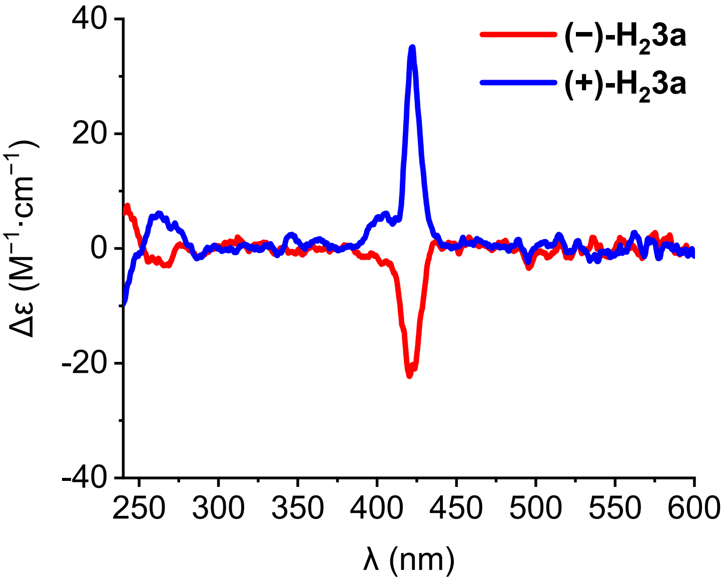

Figure S4. ECD spectra (CH_2Cl_2, 298 K) of the enantiomers of **$H_2$3a**.

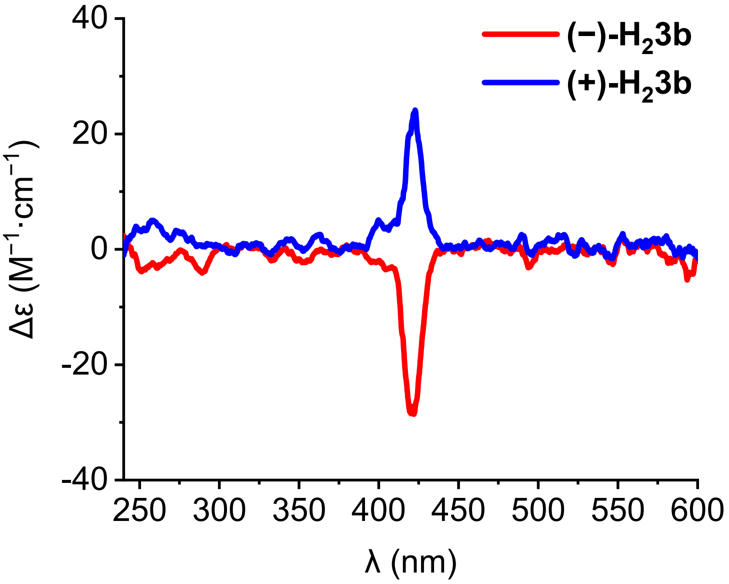

Figure **S5**. ECD spectra (CH_2Cl_2, 298 K) of the enantiomers of **$H_2$3b**.

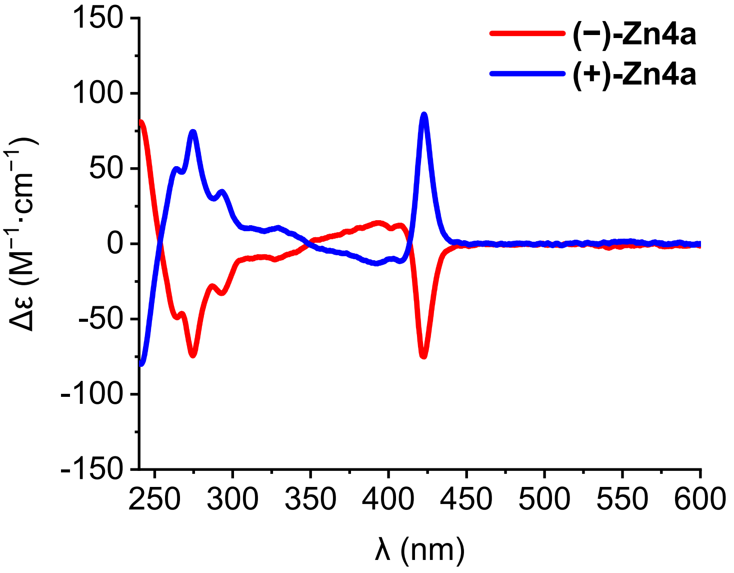

Figure S6. ECD spectra (CH_2Cl_2, 298 K) of the enantiomers of **Zn4a**.

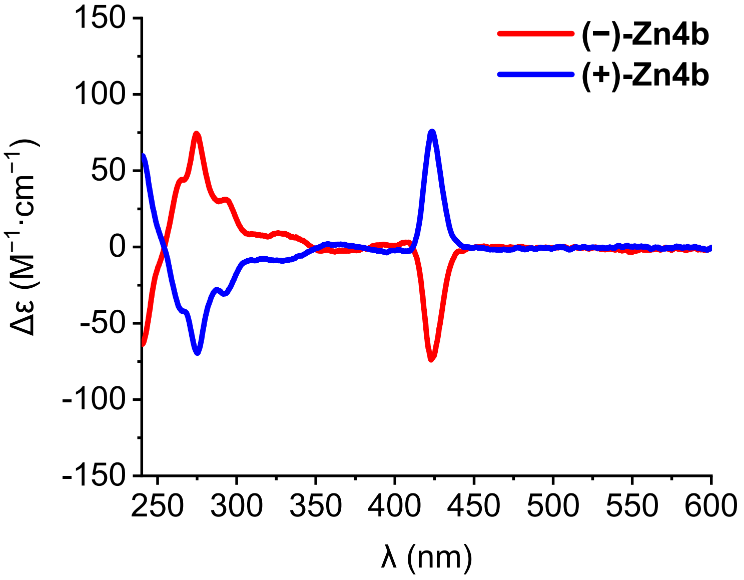

Figure S7. ECD spectra (CH_2Cl_2, 298 K) of the enantiomers of **Zn4b**.

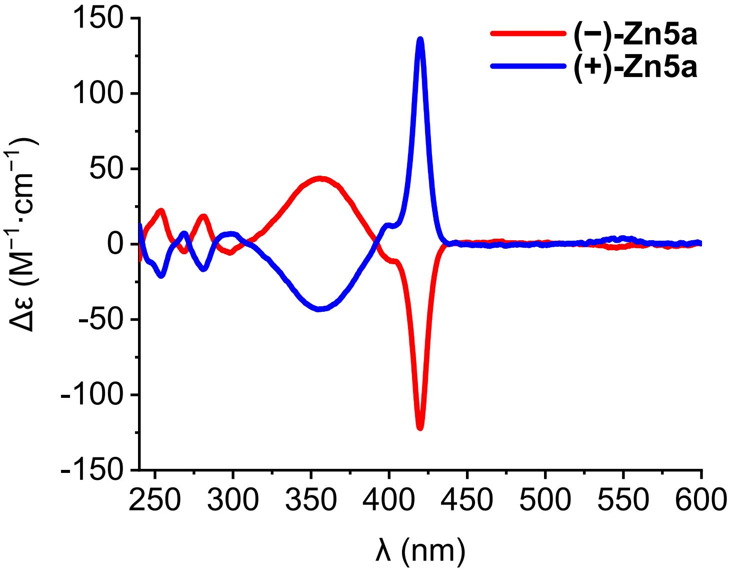

Figure S8. ECD spectra (CH_2Cl_2, 298 K) of the enantiomers of **Zn5a**.

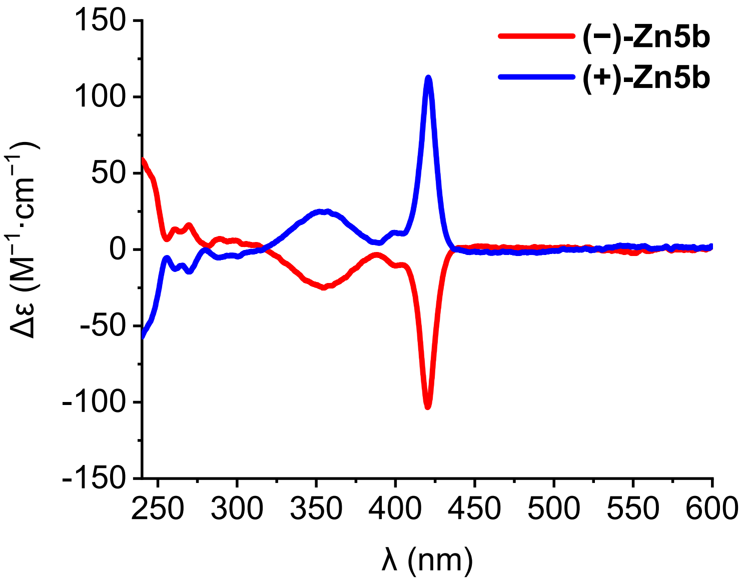

Figure S9. ECD spectra (CH_2Cl_2, 298 K) of the enantiomers of **Zn5b**.

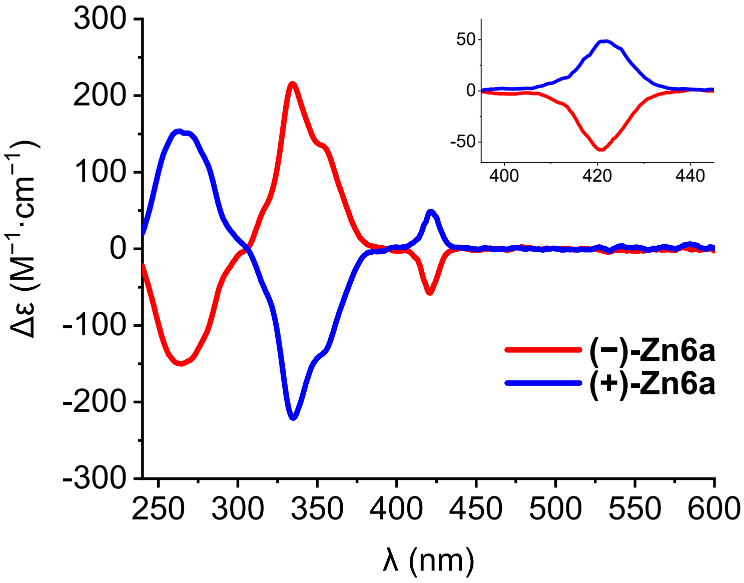

Figure S10. ECD spectra (CH_2Cl_2, 298 K) of the enantiomers of **Zn6a**.

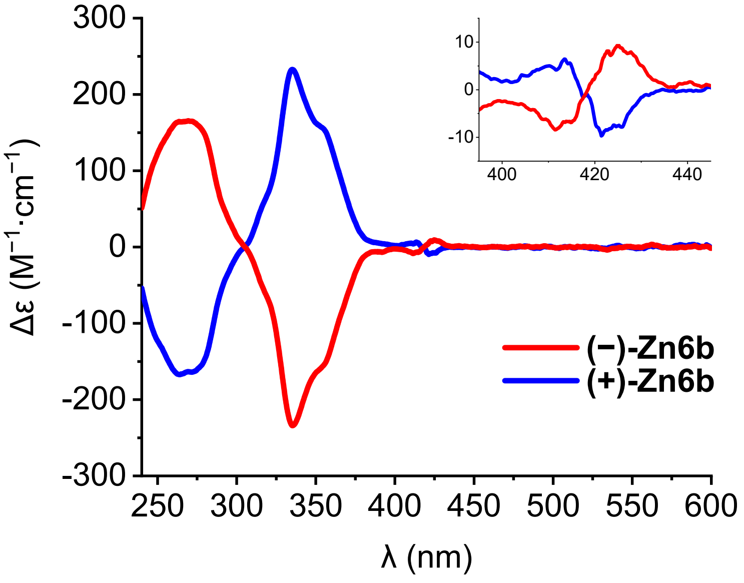

Figure S11. ECD spectra (CH_2Cl_2, 298 K) of the enantiomers of **Zn6b.**

4.2. Fluorescence threading spectra

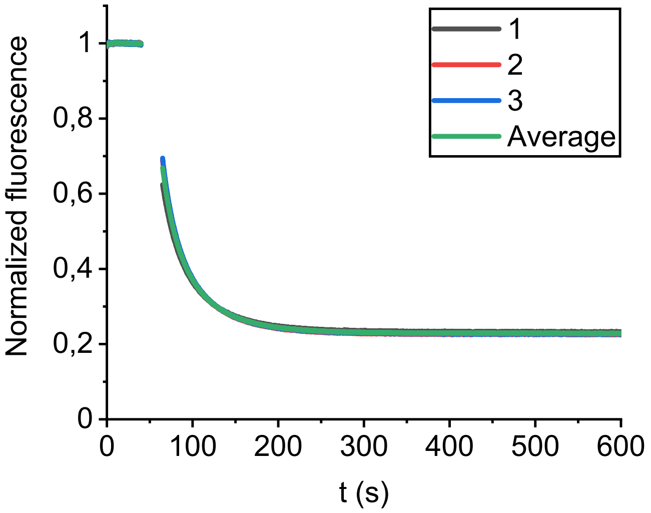

Figure S12. Normalized fluorescence intensity of host (−)-**$H_2$2** as a function of time after the addition (t = 50 s) of 1 equivalent of guest (*S,S*)-**7** (c = 10^{-5} M in $CHCl_3/CH_3CN$, 1:1, v/v, 298 K). The experiment was performed in triplicate.

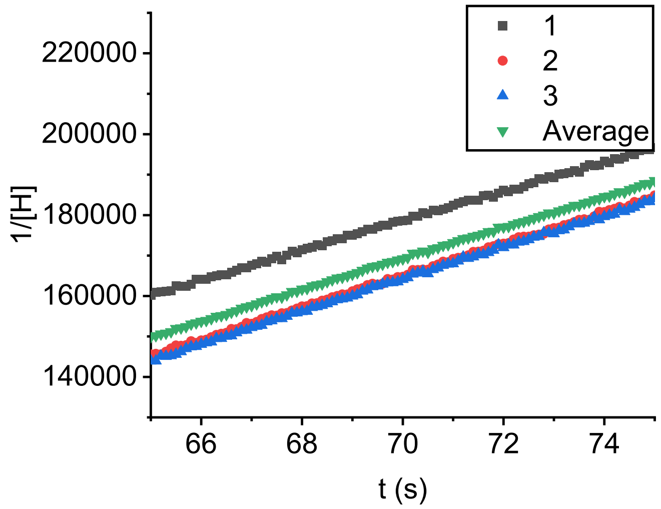

Figure S13. Second-order kinetics plots for the complexation of host **(−)-$H_2$2** to guest (*S,S*)-**7** (c = 10^{-5} M in $CHCl_3/CH_3CN$, 1:1, v/v, 298 K).

Table S4. Kinetic data for the threading of host (−)-**$H_2$2** onto guest (*S,S*)-**7**.

**Entry**	**Host**	**Guest**	**k_{on} (× 10^3 $M^{-1}\cdot s^{-1}$)**	**R^2**
1	**$H_2$2 (negative Soret)**	(*S,S*)-**7**	3.64	0.999
2	**$H_2$2 (negative Soret)**	(*S,S*)-**7**	3.92	0.999
3	**$H_2$2 (negative Soret)**	(*S,S*)-**7**	3.97	0.999
**AVERAGE**	**$H_2$2 (negative Soret)**	(*S,S*)-**7**	3.85	0.999

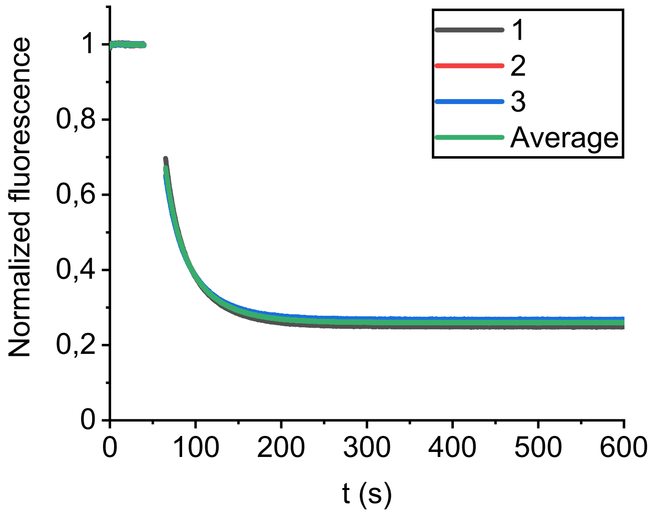

Figure S14. Normalized fluorescence intensity of host (+)-$\mathbf{H_22}$ as a function of time after the addition (t = 50 s) of 1 equivalent of guest (*R,R*)-**7** (c = 10^{-5} M in $CHCl_3/CH_3CN$, 1:1, v/v, 298 K). The experiment was performed in triplicate.

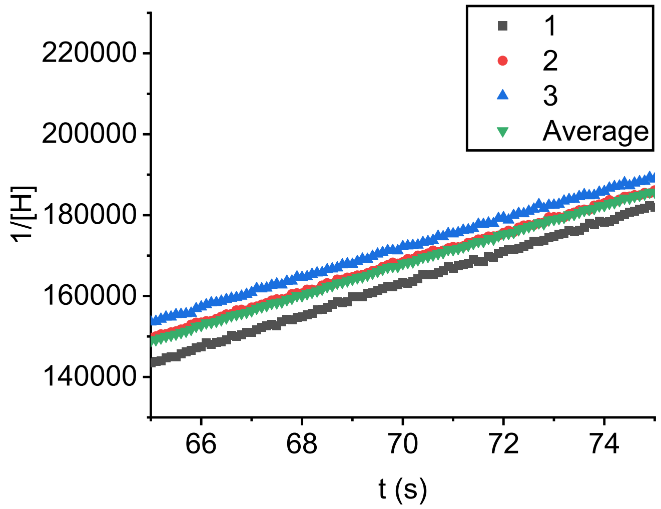

Figure S15. Second-order kinetics plots for the complexation of host (+)-$\mathbf{H_22}$ to guest (*R*,*R*)-**7** (c = 10^{-5} M in $CHCl_3/CH_3CN$, 1:1, v/v, 298 K).

Table S5. Kinetic data for the threading of host (+)-$\mathbf{H_22}$ onto guest (*R*,*R*)-**7**.

**Entry**	**Host**	**Guest**	**k_{on} (× 10^3 M^{-1}·s^{-1})**	**R^2**
1	**H_22 (positive Soret)**	(*R*,*R*)-**7**	3.90	0.999
2	**H_22 (positive Soret)**	(*R*,*R*)-**7**	3.67	0.999
3	**H_22 (positive Soret)**	(*R*,*R*)-**7**	3.61	0.999
**AVERAGE**	**H_22 (positive Soret)**	(*R*,*R*)-**7**	3.72	0.999

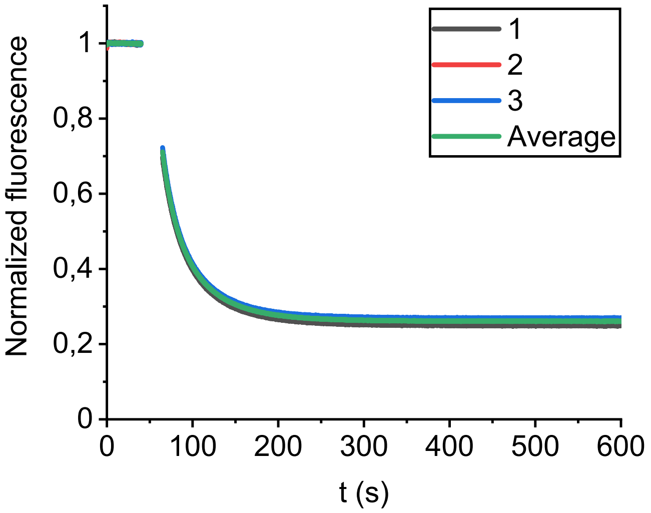

Figure S16. Normalized fluorescence intensity of host (+)-$\mathbf{H_22}$ as a function of time after the addition (t = 50 s) of 1 equivalent of guest (*S,S*)-**7** (c = 10^{-5} M in $CHCl_3/CH_3CN$, 1:1, v/v, 298 K). The experiment was performed in triplicate.

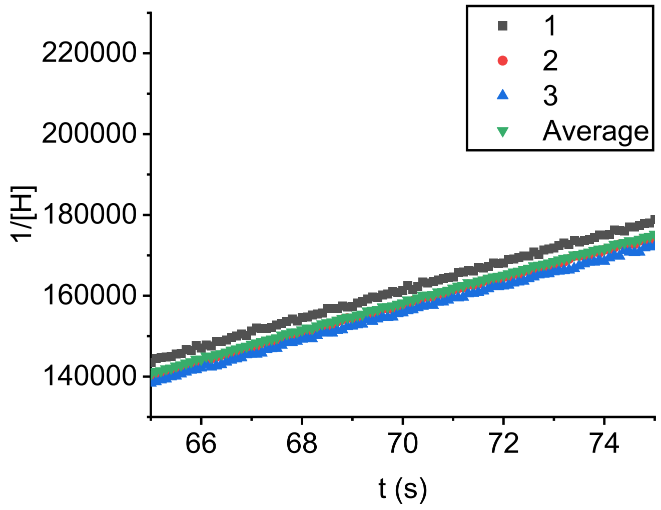

Figure S17. Second-order kinetics plots for the complexation of host (+)-$\mathbf{H_22}$ to guest (*S,S*)-**7** (c = 10^{-5} M in $CHCl_3/CH_3CN$, 1:1, v/v, 298 K).

Table S6. Kinetic data for the threading of host (+)-$\mathbf{H_22}$ onto guest (*S,S*)-**7**.

**Entry**	**Host**	**Guest**	**k_{on} (× 10^3 M^{-1}·s^{-1})**	**R^2**
1	**$H_2$2 (positive Soret)**	(*S,S*)-**7**	3.47	0.999
2	**$H_2$2 (positive Soret)**	(*S,S*)-**7**	3.43	0.999
3	**$H_2$2 (positive Soret)**	(*S,S*)-**7**	3.38	0.999
**AVERAGE**	**$H_2$2 (positive Soret)**	(*S,S*)-**7**	3.43	0.999

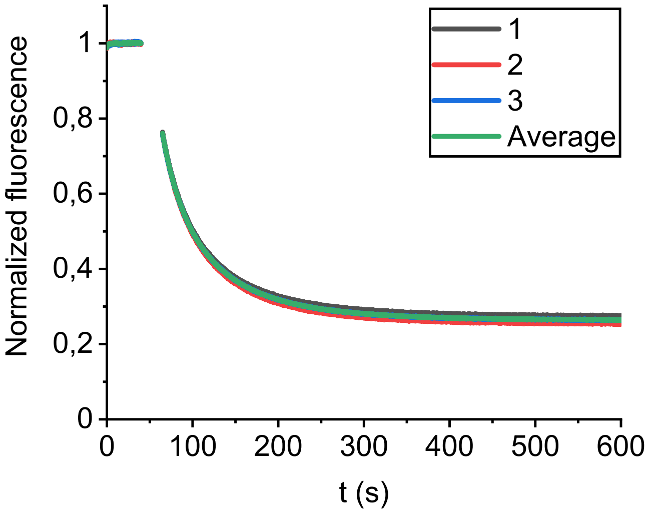

Figure S18. Normalized fluorescence intensity of host (−)-**$H_2$3a** as a function of time after the addition (t = 50 s) of 1 equivalent of guest (*R,R*)-**7** (c = 10^{-5} M in $CHCl_3/CH_3CN$, 1:1, v/v, 298 K). The experiment was performed in triplicate.

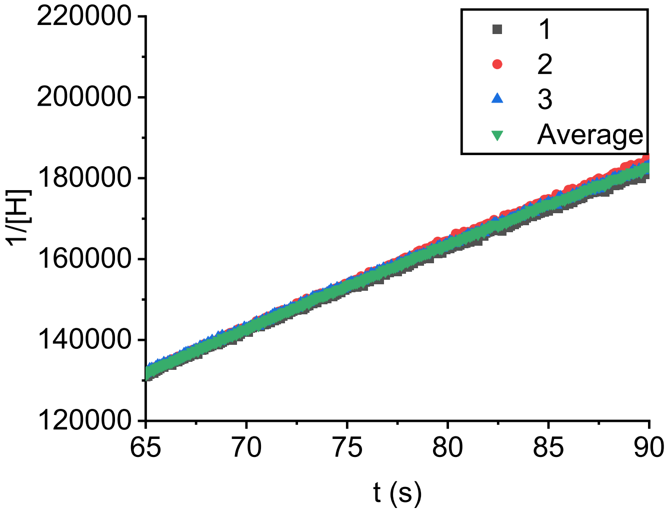

Figure S19. Second-order kinetics plots for the complexation of host (−)-**$H_2$3a** to guest (*R,R*)-**7** (c = 10^{-5} M in $CHCl_3/CH_3CN$, 1:1, v/v, 298 K).

Table S7. Kinetic data for the threading of host (−)-**$H_2$3a** onto guest (*R,R*)-**7**.

**Entry**	**Host**	**Guest**	**k_{on} (× 10^3 M^{-1}·s^{-1})**	**R^2**
1	**$H_2$3a (negative Soret)**	(*R*,*R*)-**7**	2.00	0.998
2	**$H_2$3a (negative Soret)**	(*R*,*R*)-**7**	2.09	0.999
3	**$H_2$3a (negative Soret)**	(*R*,*R*)-**7**	2.03	0.998
**AVERAGE**	**$H_2$3a (negative Soret)**	(*R*,*R*)-**7**	2.04	0.999

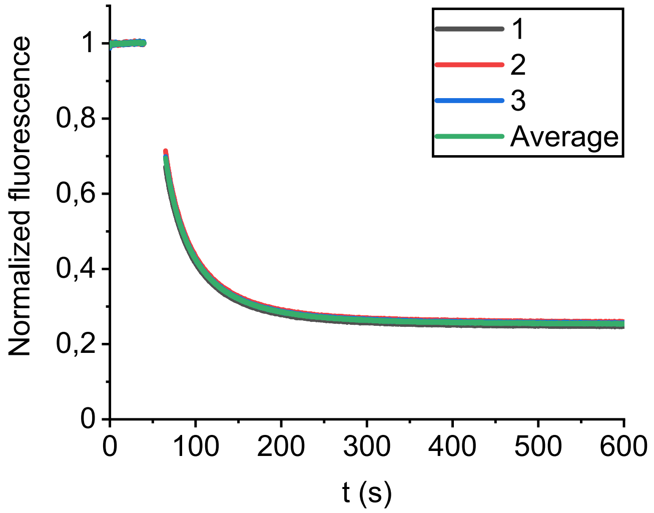

Figure S20. Normalized fluorescence intensity of host (−)-**$H_2$3a** as a function of time after the addition (t = 50 s) of 1 equivalent of guest (S,S)-**7** (c = 10^{-5} M in $CHCl_3/CH_3CN$, 1:1, v/v, 298 K). The experiment was performed in triplicate.

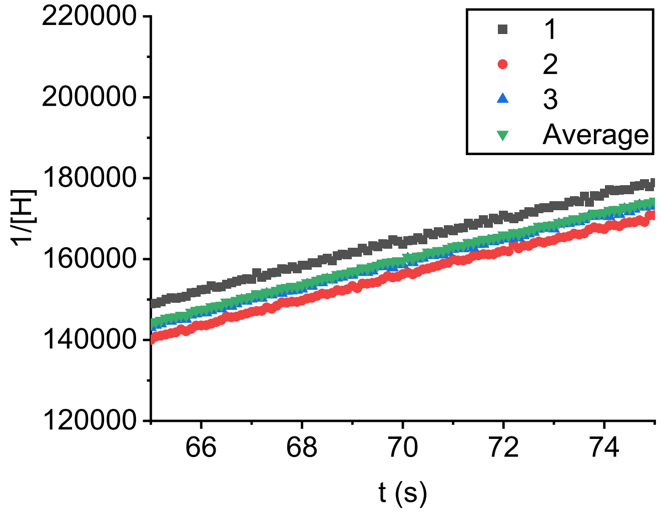

Figure S21. Second-order kinetics plots for the complexation of host (−)-**$H_2$3a** to guest (*S,S*)-**7** (c = 10^{-5} M in $CHCl_3/CH_3CN$, 1:1, v/v, 298 K).

Table S8. Kinetic data for the threading of host (−)-**$H_2$3a** onto guest (*S,S*)-**7**.

Entry	Host	Guest	k_{on} (× 10^3 M^{-1}·s^{-1})	R^2
1	**$H_2$3a (negative Soret)**	(*S,S*)-**7**	2.97	0.997
2	**$H_2$3a (negative Soret)**	(*S,S*)-**7**	3.02	0.998
3	**$H_2$3a (negative Soret)**	(*S,S*)-**7**	3.01	0.998
**AVERAGE**	**$H_2$3a (negative Soret)**	(*S,S*)-**7**	3.00	0.998

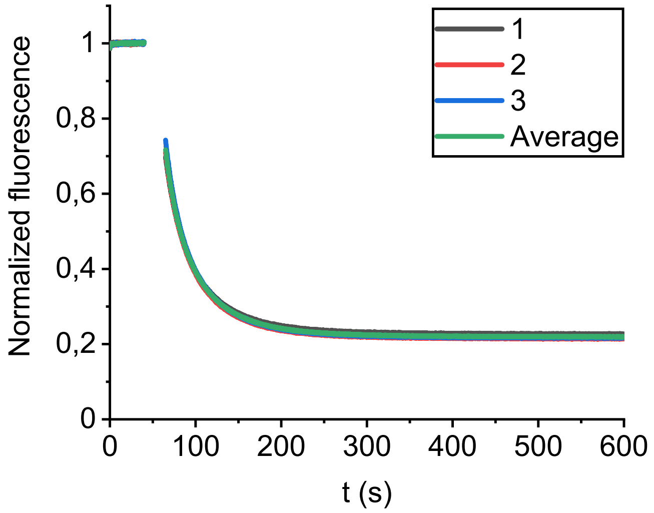

FigureS22. Normalized fluorescence intensity of host (+)-**$H_2$3a** as a function of time after the addition (t = 50 s) of 1 equivalent of guest (*R,R*)-**7** (c = 10^{-5} M in $CHCl_3$/CH_3CN, 1:1, v/v, 298 K). The experiment was performed in triplicate.

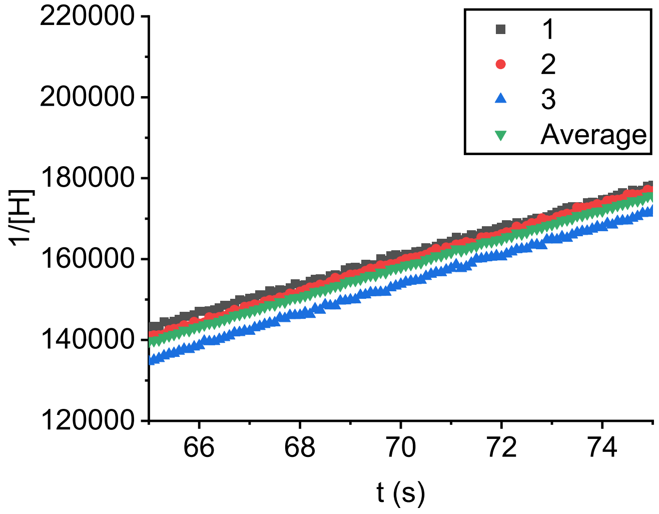

Figure S23. Second-order kinetics plots for the complexation of host (+)-**$H_2$3a** to guest (*R,R*)-**7** (c = 10^{-5} M in $CHCl_3$/CH_3CN, 1:1, v/v, 298 K).

Table S9. Kinetic data for the threading of host (+)-**$H_2$3a** onto guest (*R,R*)-**7**.

**Entry**	**Host**	**Guest**	**k_{on} (× 10^3 M^{-1}·s^{-1})**	**R^2**
1	**$H_2$3a (positive Soret)**	(*R,R*)-**7**	3.50	0.999
2	**$H_2$3a (positive Soret)**	(*R,R*)-**7**	3.65	0.998
3	**$H_2$3a (positive Soret)**	(*R,R*)-**7**	3.68	0.998
**AVERAGE**	**$H_2$3a (positive Soret)**	(*R,R*)-**7**	3.61	0.999

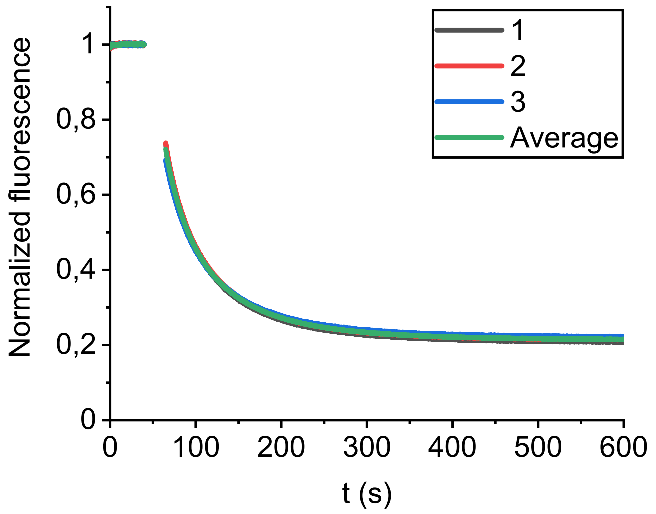

Figure S24. Normalized fluorescence intensity of host (+)-$\mathbf{H_23a}$ as a function of time after the addition (t = 50 s) of 1 equivalent of guest (*S,S*)-**7** (c = 10^{-5} M in $CHCl_3/CH_3CN$, 1:1, v/v, 298 K). The experiment was performed in triplicate.

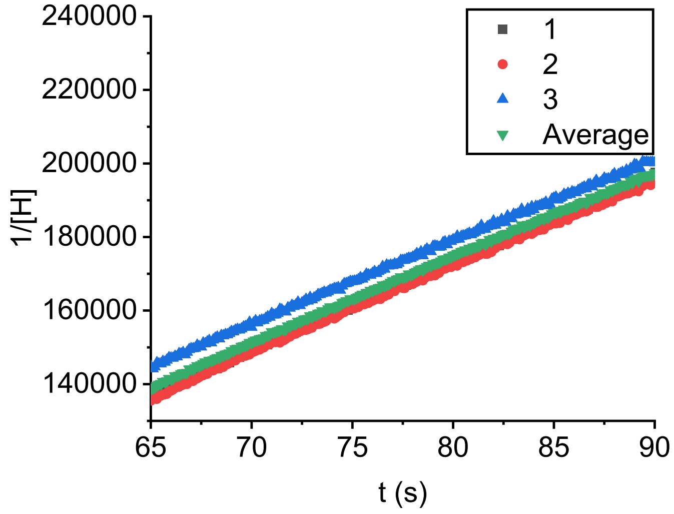

Figure S25. Second-order kinetics plots for the complexation of host (+)-$\mathbf{H_23a}$ to guest (*S,S*)-**7** (c = 10^{-5} M in $CHCl_3/CH_3CN$, 1:1, v/v, 298 K).

Table S10. Kinetic data for the threading of host (+)-$\mathbf{H_23a}$ onto guest (*S,S*)-**7**.

**Entry**	**Host**	**Guest**	k_{on} **(× 10^3 $M^{-1}·s^{-1}$)**	R^2
1	**H_23a (positive Soret)**	(*S,S*)-**7**	2.40	0.999
2	**H_23a (positive Soret)**	(*S,S*)-**7**	2.36	0.999
3	**H_23a (positive Soret)**	(*S,S*)-**7**	2.25	0.999
**AVERAGE**	**H_23a (positive Soret)**	(*S,S*)-**7**	2.33	0.999

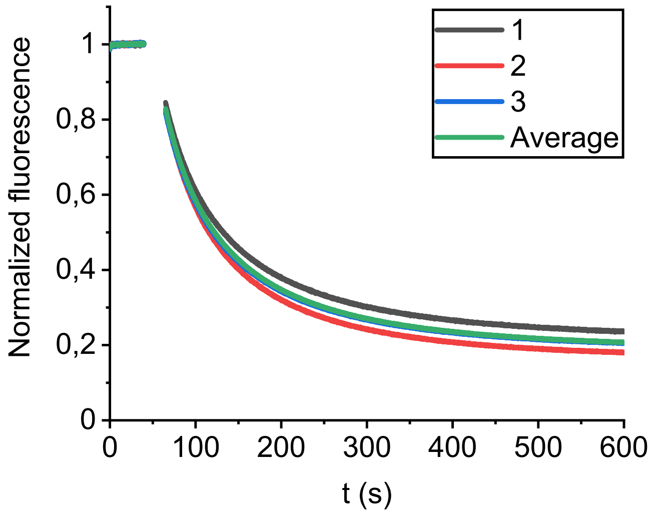

Figure S26. Normalized fluorescence intensity of host (−)-$\mathbf{H_23b}$ as a function of time after the addition (t = 50 s) of 1 equivalent of guest (*R,R*)-**7** (c = 10^{-5} M in $CHCl_3/CH_3CN$, 1:1, v/v, 298 K). The experiment was performed in triplicate.

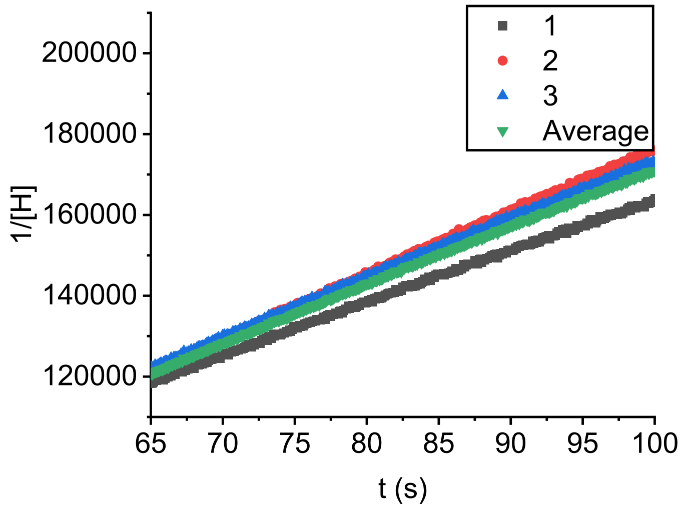

Figure S27. Second-order kinetics plots for the complexation of host (−)-$\mathbf{H_23b}$ to guest (*R,R*)-**7** (c = 10^{-5} M in $CHCl_3/CH_3CN$, 1:1, v/v, 298 K).

Table S11. Kinetic data for the threading of host (−)-$\mathbf{H_23b}$ onto guest (*R,R*)-**7**.

**Entry**	**Host**	**Guest**	k_{on} **(× 10^3 $M^{-1}\cdot s^{-1}$)**	R^2
1	**$H_2$3b (negative Soret)**	(*R*,*R*)-**7**	1.29	0.999
2	**$H_2$3b (negative Soret)**	(*R*,*R*)-**7**	1.57	0.999
3	**$H_2$3b (negative Soret)**	(*R*,*R*)-**7**	1.47	0.999
**AVERAGE**	**$H_2$3b (negative Soret)**	(*R*,*R*)-**7**	1.44	0.999

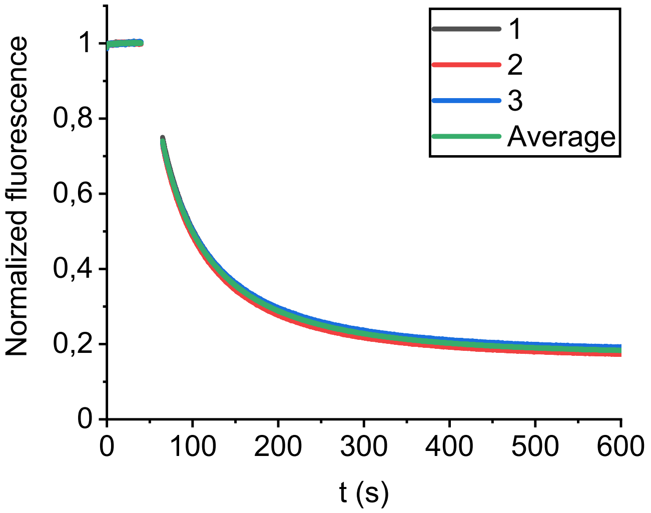

Figure S28. Normalized fluorescence intensity of host (−)-**$H_2$3b** as a function of time after the addition (t = 50 s) of 1 equivalent of guest (*S,S*)-**7** (c = 10^{-5} M in $CHCl_3/CH_3CN$, 1:1, v/v, 298 K). The experiment was performed in triplicate.

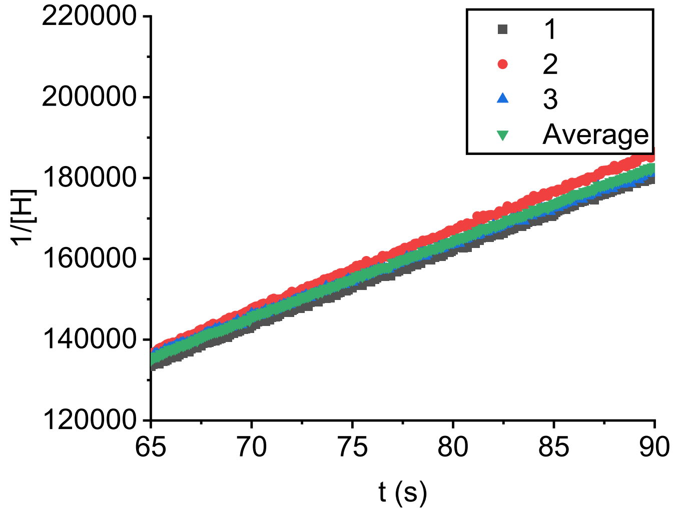

Figure S29. Second-order kinetics plots for the complexation of host (−)-**$H_2$3b** to guest (*S,S*)-**7** (c = 10^{-5} M in $CHCl_3/CH_3CN$, 1:1, v/v, 298 K).

Table S12. Kinetic data for the threading of host (−)-**$H_2$3b** onto guest (*S,S*)-**7**.

**Entry**	**Host**	**Guest**	**k_{on} (× 10^3 M^{-1}·s^{-1})**	**R^2**
1	**$H_2$3b (negative Soret)**	(*S,S*)-**7**	1.87	0.999
2	**$H_2$3b (negative Soret)**	(*S,S*)-**7**	1.96	0.999
3	**$H_2$3b (negative Soret)**	(*S,S*)-**7**	1.83	0.999
**AVERAGE**	**$H_2$3b (negative Soret)**	(*S,S*)-**7**	1.89	0.999

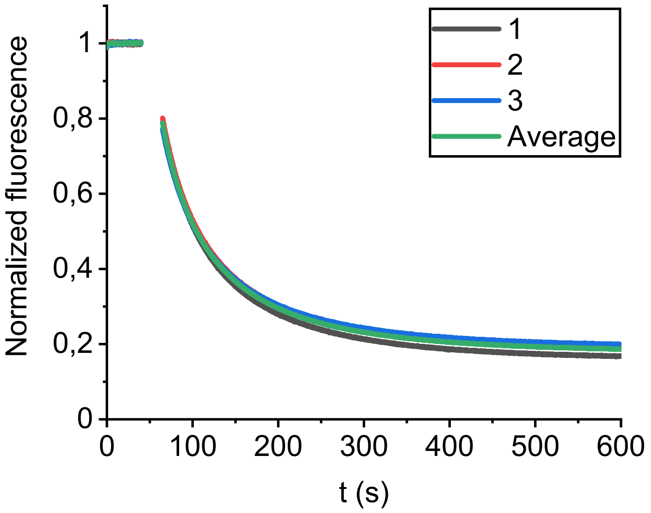

Figure S30. Normalized fluorescence intensity of host (+)-$\mathbf{H_23b}$ as a function of time after the addition (t = 50 s) of 1 equivalent of guest (*R,R*)-**7** (c = 10^{-5} M in $CHCl_3/CH_3CN$, 1:1, v/v, 298 K). The experiment was performed in triplicate.

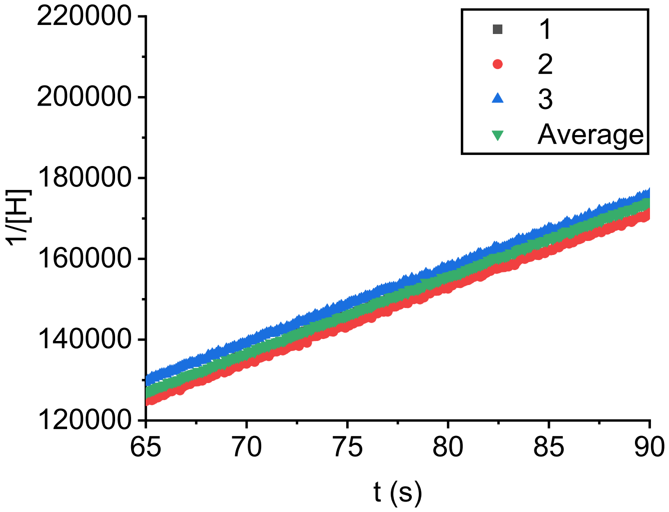

Figure S31. Second-order kinetics plots for the complexation of host (+)-$\mathbf{H_23b}$ to guest (*R,R*)-**7** (c = 10^{-5} M in $CHCl_3/CH_3CN$, 1:1, v/v, 298 K).

Table S13. Kinetic data for the threading of host (+)-$\mathbf{H_23b}$ onto guest (*R,R*)-**7**.

Entry	Host	Guest	k_{on} (× 10^3 $M^{-1}\cdot s^{-1}$)	R^2
1	**H_23b (positive Soret)**	(*R*,*R*)-**7**	1.96	0.999
2	**H_23b (positive Soret)**	(*R*,*R*)-**7**	1.86	0.999
3	**H_23b (positive Soret)**	(*R*,*R*)-**7**	1.84	0.999
**AVERAGE**	**H_23b (positive Soret)**	(*R*,*R*)-**7**	1.89	0.999

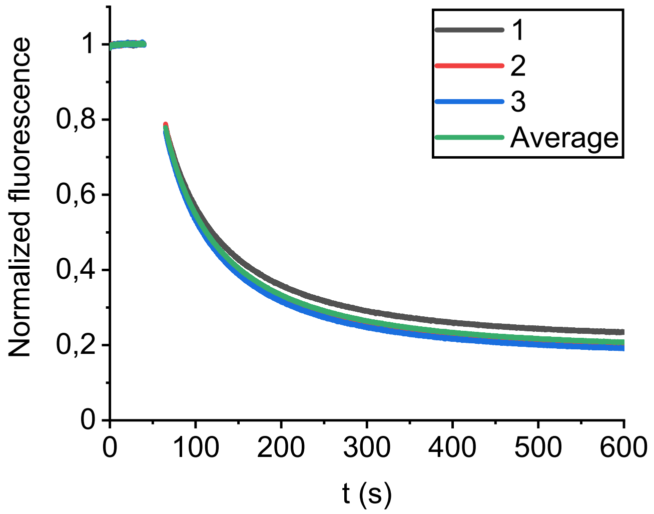

Figure S32. Normalized fluorescence intensity of host (+)-$\mathbf{H_2 3b}$ as a function of time after the addition (t = 50 s) of 1 equivalent of guest (*S,S*)-**7** (c = 10^{-5} M in $CHCl_3/CH_3CN$, 1:1, v/v, 298 K). The experiment was performed in triplicate.

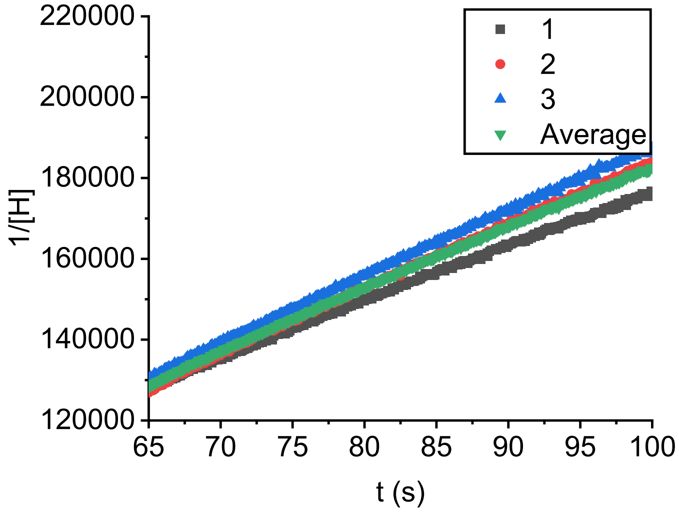

Figure S33. Second-order kinetics plots for the complexation of host (+)-$\mathbf{H_2 3b}$ to guest (*S,S*)-**7** (c = 10^{-5} M in $CHCl_3/CH_3CN$, 1:1, v/v, 298 K).

Table S14. Kinetic data for the threading of host (+)-$\mathbf{H_2 3b}$ onto guest (*S,S*)-**7**.

**Entry**	**Host**	**Guest**	k_{on} **(× 10^3 $M^{-1}\cdot s^{-1}$)**	R^2
1	**$H_2$3b (positive Soret)**	(*S,S*)-**7**	1.38	0.998
2	**$H_2$3b (positive Soret)**	(*S,S*)-**7**	1.60	0.998
3	**$H_2$3b (positive Soret)**	(*S,S*)-**7**	1.63	0.999
**AVERAGE**	**$H_2$3b (positive Soret)**	(*S,S*)-**7**	1.54	0.999

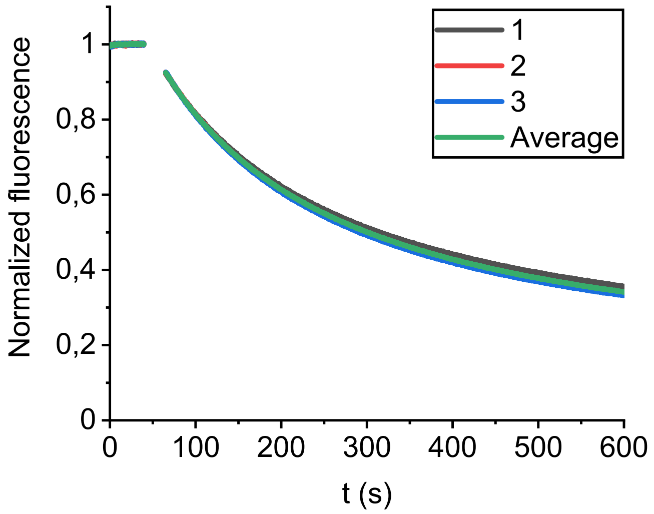

Figure S34. Normalized fluorescence intensity of host **Zn1** as a function of time after the addition (t = 50 s) of 1 equivalent of guest (*R,R*)-**7** (c = 10^{-5} M in $CHCl_3/CH_3CN$, 1:1, v/v, 298 K). The experiment was performed in triplicate.

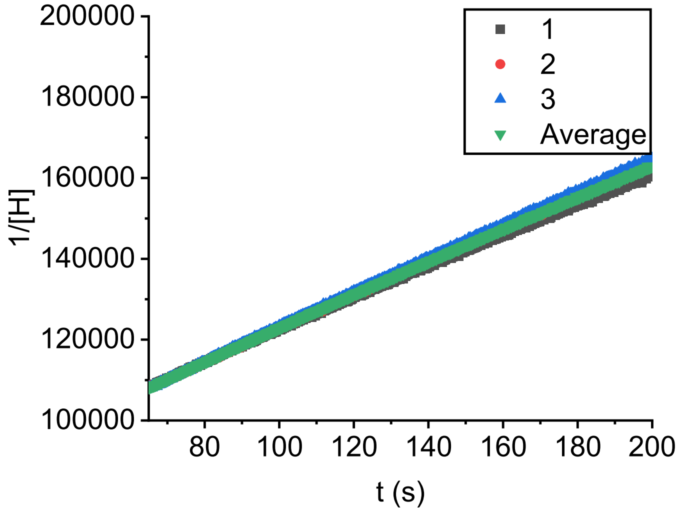

Figure S35. Second-order kinetics plots for the complexation of host **Zn1** to guest (*R,R*)-**7** (c = 10^{-5} M in $CHCl_3/CH_3CN$, 1:1, v/v, 298 K).

Table S15. Kinetic data for the threading of host **Zn1** onto guest (*R,R*)-**7**.

**Entry**	**Host**	**Guest**	k_{on} **(× 10^2 $M^{-1}\cdot s^{-1}$)**	R^2
1	**Zn1**	(*R,R*)-**7**	3.88	0.999
2	**Zn1**	(*R,R*)-**7**	4.11	0.999
3	**Zn1**	(*R,R*)-**7**	4.19	0.999
**AVERAGE**	**Zn1**	(*R,R*)-**7**	4.06	0.999

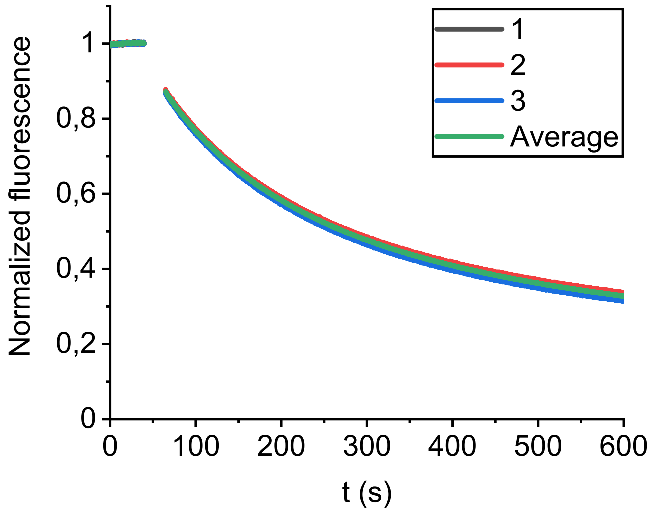

Figure S36. Normalized fluorescence intensity of host **Zn1** as a function of time after the addition (t = 50 s) of 1 equivalent of guest (*S,S*)-**7** (c = 10^{-5} M in $CHCl_3/CH_3CN$, 1:1, v/v, 298 K). The experiment was performed in triplicate.

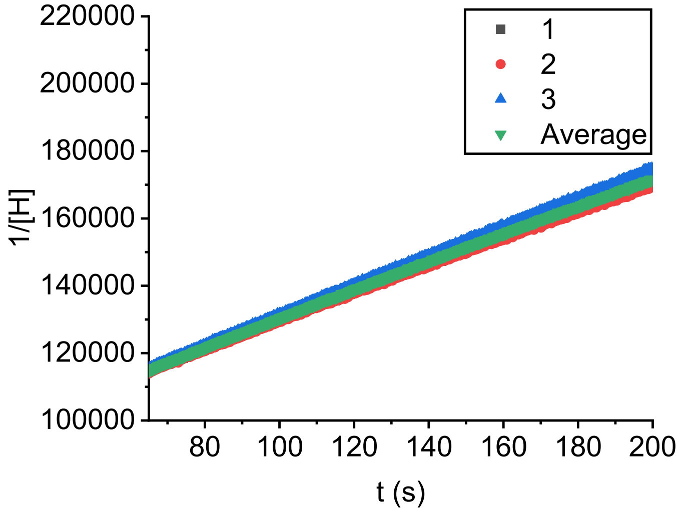

Figure S37. Second-order kinetics plots for the complexation of host **Zn1** to guest (*S,S*)-**7** (c = 10^{-5} M in $CHCl_3/CH_3CN$, 1:1, v/v, 298 K).

Table S16. Kinetic data for the threading of host **Zn1** onto guest (*S,S*)-**7**.

**Entry**	**Host**	**Guest**	**k_{on} (× 10^2 $M^{-1}\cdot s^{-1}$)**	**R^2**
1	**Zn1**	(*S,S*)-**7**	4.12	0.999
2	**Zn1**	(*S,S*)-**7**	4.09	0.999
3	**Zn1**	(*S,S*)-**7**	4.37	0.999
**AVERAGE**	**Zn1**	(*S,S*)-**7**	4.19	0.999

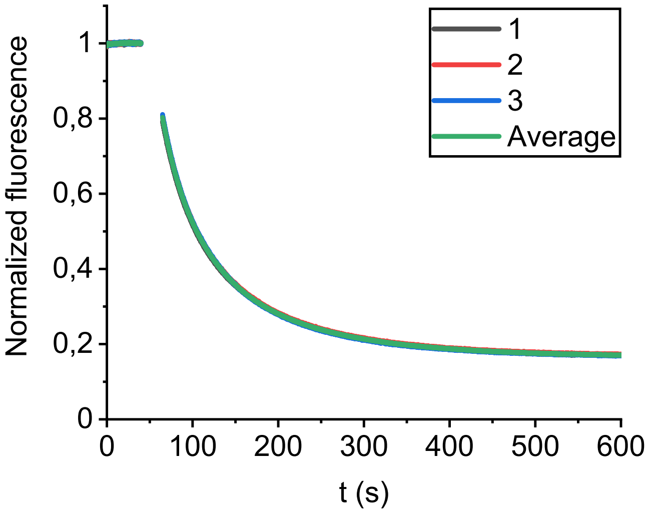

Figure S38. Normalized fluorescence intensity of host (−)-**Zn2** as a function of time after the addition (t = 50 s) of 1 equivalent of guest (*R,R*)-**7** (c = 10^{-5} M in $CHCl_3/CH_3CN$, 1:1, v/v, 298 K). The experiment was performed in triplicate.

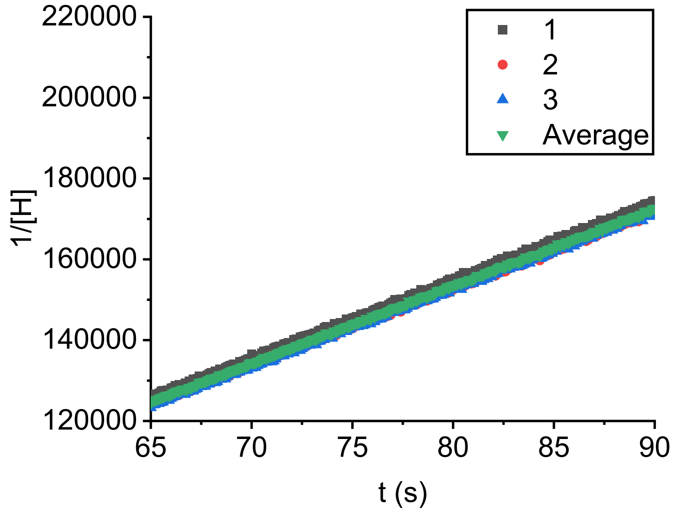

Figure S39. Second-order kinetics plots for the complexation of host (−)-**Zn2** to guest (*R*,*R*)-**7** (c = 10^{-5} M in $CHCl_3/CH_3CN$, 1:1, v/v, 298 K).

Table S17. Kinetic data for the threading of host (−)-**Zn2** onto guest (*R,R*)-**7**.

**Entry**	**Host**	**Guest**	k_{on} **(× 10^3 $M^{-1}\cdot s^{-1}$)**	R^2
1	(−)-**Zn2 (negative Soret)**	(*R*,*R*)-**7**	1.93	0.999
2	(−)-**Zn2 (negative Soret)**	(*R*,*R*)-**7**	1.90	0.999
3	(−)-**Zn2 (negative Soret)**	(*R*,*R*)-**7**	1.91	0.999
**AVERAGE**	(−)-**Zn2 (negative Soret)**	(*R*,*R*)-**7**	1.91	0.999

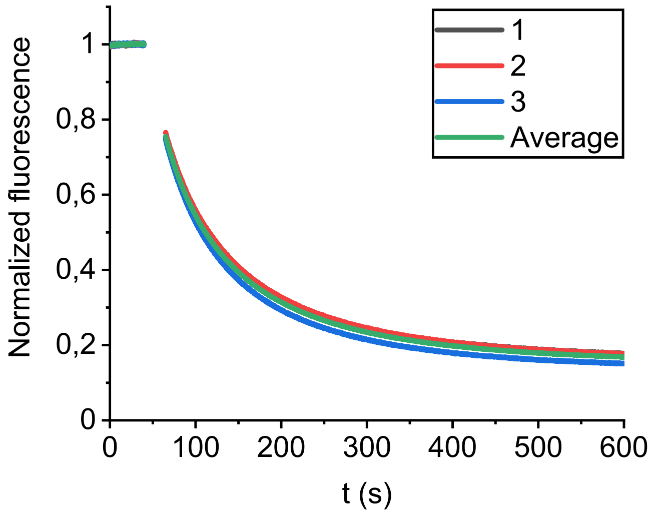

Figure S40. Normalized fluorescence intensity of host (−)-**Zn2** as a function of time after the addition (t = 50 s) of 1 equivalent of guest (*S,S*)-**7** (c = 10^{-5} M in $CHCl_3/CH_3CN$, 1:1, v/v, 298 K). The experiment was performed in triplicate.

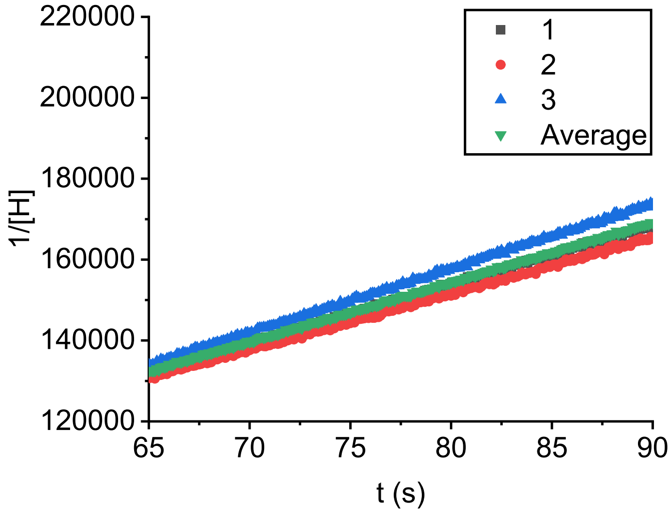

Figure S41. Second-order kinetics plots for the complexation of host (−)-**Zn2** to guest (*S*,*S*)-**7** (c = 10^{-5} M in $CHCl_3/CH_3CN$, 1:1, v/v, 298 K).

Table S18. Kinetic data for the threading of host (−)-**Zn2** onto guest (*S*,*S*)-**7**.

**Entry**	**Host**	**Guest**	**k_{on} (× 10^3 $M^{-1}\cdot s^{-1}$)**	**R^2**
1	(−)-**Zn2 (negative Soret)**	(*S*,*S*)-**7**	1.44	0.999
2	(−)-**Zn2 (negative Soret)**	(*S*,*S*)-**7**	1.39	0.999
3	(−)-**Zn2 (negative Soret)**	(*S*,*S*)-**7**	1.59	0.999
**AVERAGE**	(−)-**Zn2 (negative Soret)**	(*S*,*S*)-**7**	1.47	0.999

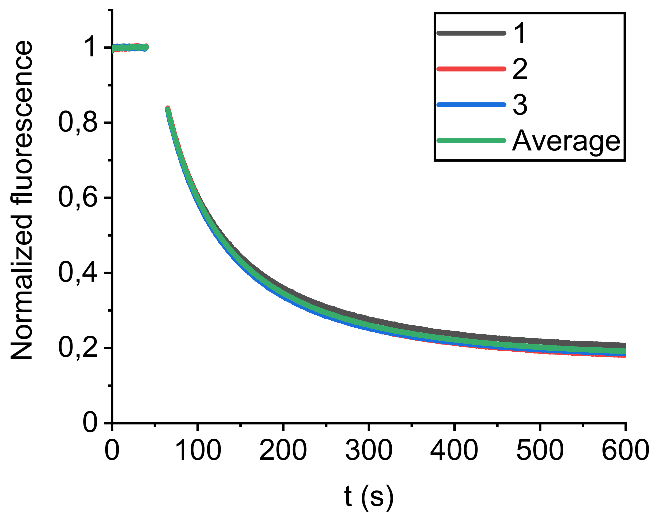

Figure S42. Normalized fluorescence intensity of host (+)-**Zn2** as a function of time after the addition (t = 50 s) of 1 equivalent of guest (*R,R*)-**7** (c = 10^{-5} M in $CHCl_3/CH_3CN$, 1:1, v/v, 298 K). The experiment was performed in triplicate.

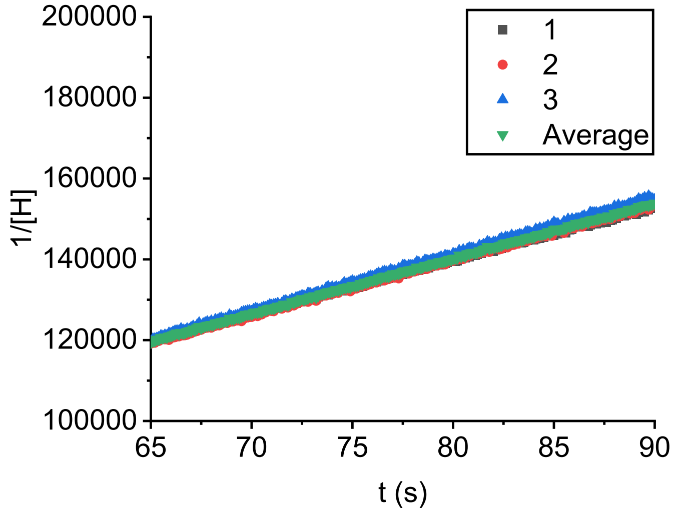

Figure S43. Second-order kinetics plots for the complexation of host (+)-**Zn2** to guest (*R,R*)-**7** (c = 10^{-5} M in $CHCl_3/CH_3CN$, 1:1, v/v, 298 K).

Table S19. Kinetic data for the threading of host (+)-**Zn2** onto guest (*R,R*)-**7**.

**Entry**	**Host**	**Guest**	**k_{on} (× 10^3 M^{-1}·s^{-1})**	**R^2**
1	(+)-**Zn2 (positive Soret)**	(*R*,*R*)-**7**	1.32	0.999
2	(+)-**Zn2 (positive Soret)**	(*R*,*R*)-**7**	1.37	0.999
3	(+)-**Zn2 (positive Soret)**	(*R*,*R*)-**7**	1.41	0.999
**AVERAGE**	(+)-**Zn2 (positive Soret)**	(*R*,*R*)-**7**	1.37	0.999

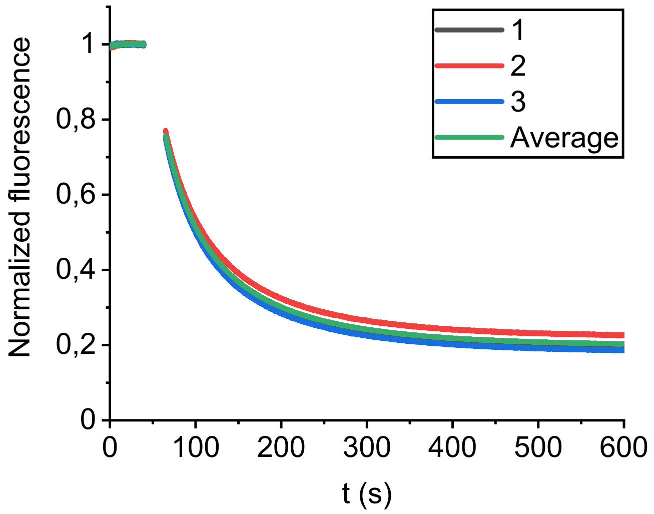

Figure S44. Normalized fluorescence intensity of host (+)-**Zn2** as a function of time after the addition (t = 50 s) of 1 equivalent of guest (*S,S*)-**7** (c = 10^{-5} M in $CHCl_3/CH_3CN$, 1:1, v/v, 298 K). The experiment was performed in triplicate.

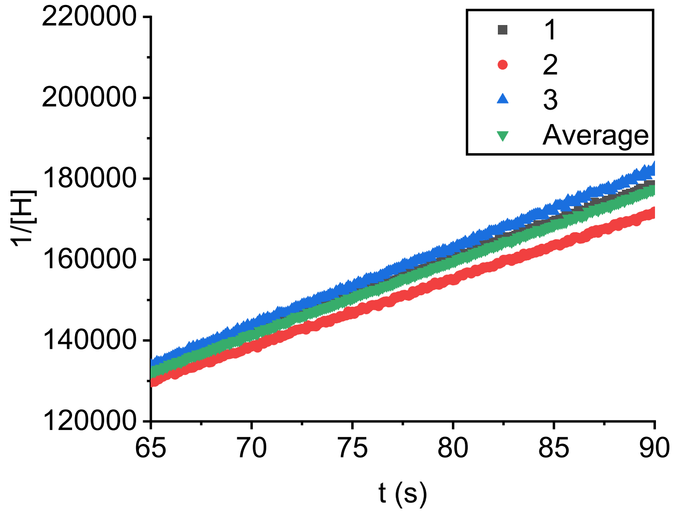

Figure S45. Second-order kinetics plots for the complexation of host (+)-**Zn2** to guest (*S,S*)-**7** (c = 10^{-5} M in $CHCl_3/CH_3CN$, 1:1, v/v, 298 K).

Table S20. Kinetic data for the threading of host (+)-**Zn2** onto guest (*S,S*)-**7**.

**Entry**	**Host**	**Guest**	**k_{on} (× 10^3 M^{-1}·s^{-1})**	**R^2**
1	(+)-**Zn2 (positive Soret)**	(*S,S*)-**7**	1.83	0.999
2	(+)-**Zn2 (positive Soret)**	(*S,S*)-**7**	1.66	0.999
3	(+)-**Zn2 (positive Soret)**	(*S,S*)-**7**	1.93	0.999
**AVERAGE**	(+)-**Zn2 (positive Soret)**	(*S,S*)-**7**	1.81	0.999

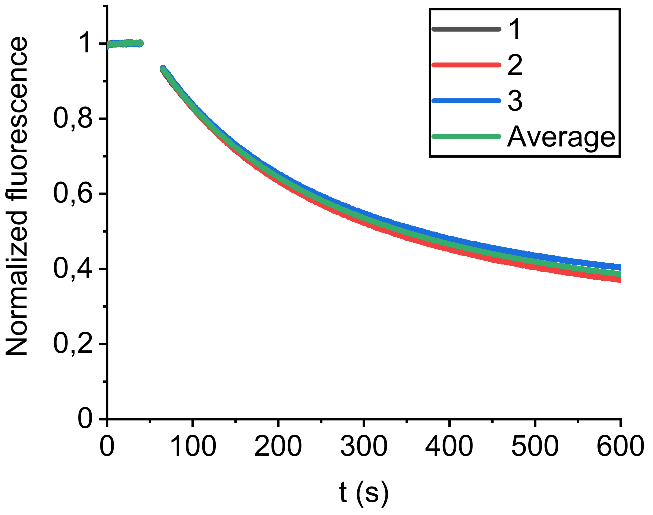

Figure S46. Normalized fluorescence intensity of host (−)-**Zn4a** as a function of time after the addition (t = 50 s) of 1 equivalent of guest (*R,R*)-**7** (c = 10^{-5} M in $CHCl_3/CH_3CN$, 1:1, v/v, 298 K). The experiment was performed in triplicate.

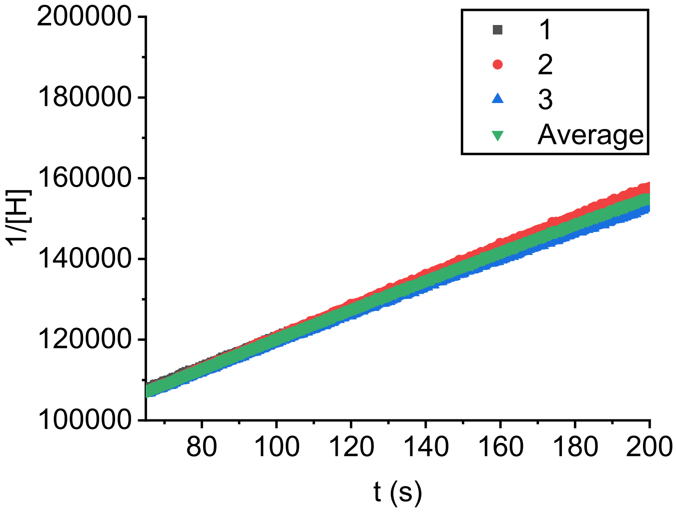

Figure S47. Second-order kinetics plots for the complexation of host (−)-**Zn4a** to guest (*R*,*R*)-**7** (c = 10^{-5} M in $CHCl_3/CH_3CN$, 1:1, v/v, 298 K).

Table S21. Kinetic data for the threading of host (−)-**Zn4a** onto guest (*R*,*R*)-**7**.

**Entry**	**Host**	**Guest**	k_{on} **(× 10^2 M^{-1}·s^{-1})**	R^2
1	(−)-**Zn4a (negative Soret)**	(*R*,*R*)-**7**	3.53	0.999
2	(−)-**Zn4a (negative Soret)**	(*R*,*R*)-**7**	3.75	0.999
3	(−)-**Zn4a (negative Soret)**	(*R*,*R*)-**7**	3.44	0.999
**AVERAGE**	(−)-**Zn4a (negative Soret)**	(*R*,*R*)-**7**	3.57	0.999

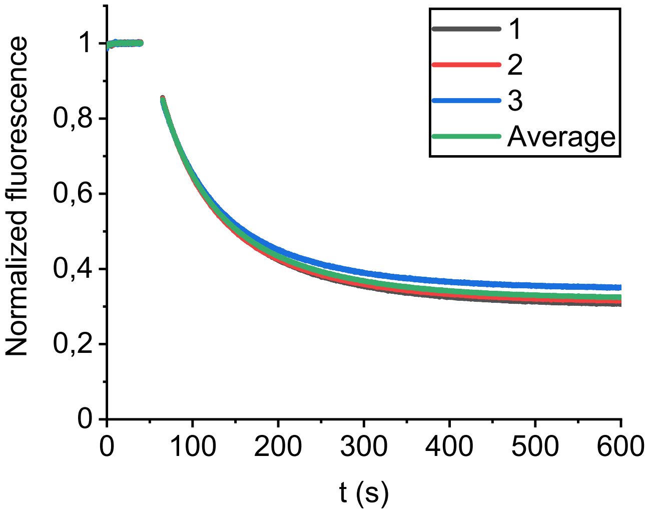

Figure S48. Normalized fluorescence intensity of host (−)-**Zn4a** as a function of time after the addition (t = 50 s) of 1 equivalent of guest (*S,S*)-**7** (c = 10^{-5} M in $CHCl_3/CH_3CN$, 1:1, v/v, 298 K). The experiment was performed in triplicate.

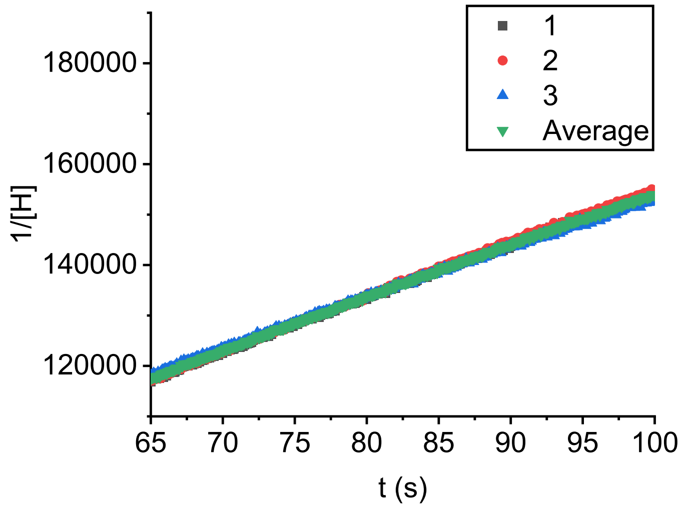

Figure S49. Second-order kinetics plots for the complexation of host (−)-**Zn4a** to guest (*S,S*)-**7** (c = 10^{-5} M in $CHCl_3/CH_3CN$, 1:1, v/v, 298 K).

Table S22. Kinetic data for the threading of host (−)-**Zn4a** onto guest (*S,S*)-**7**.

**Entry**	**Host**	**Guest**	**k_{on} (× 10^3 M^{-1}·s^{-1})**	**R^2**
1	(−)-**Zn4a (negative Soret)**	(*S,S*)-**7**	1.06	0.999
2	(−)-**Zn4a (negative Soret)**	(*S,S*)-**7**	1.08	0.999
3	(−)-**Zn4a (negative Soret)**	(*S,S*)-**7**	1.00	0.999
**AVERAGE**	(−)-**Zn4a (negative Soret)**	(*S,S*)-**7**	1.05	0.999

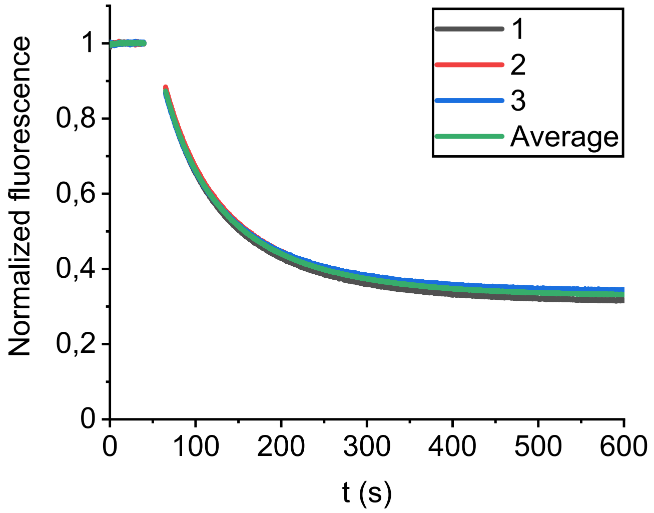

Figure S50. Normalized fluorescence intensity of host (+)-**Zn4a** as a function of time after the addition (t = 50 s) of 1 equivalent of guest (*R,R*)-**7** (c = 10^{-5} M in $CHCl_3/CH_3CN$, 1:1, v/v, 298 K). The experiment was performed in triplicate.

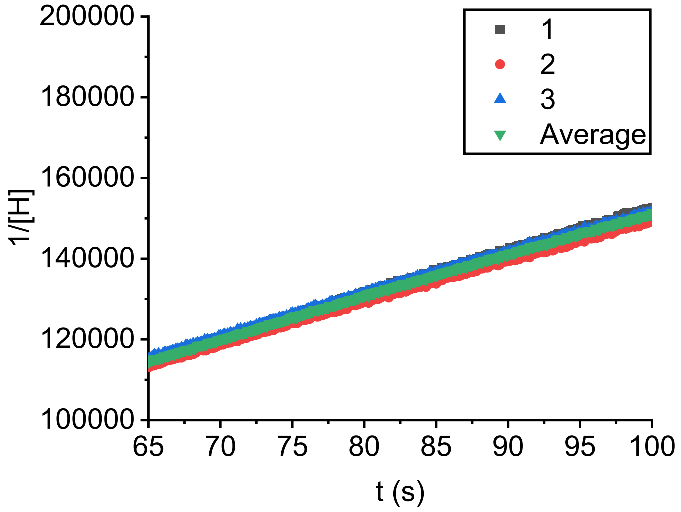

Figure S51. Second-order kinetics plots for the complexation of host (+)-**Zn4a** to guest (*R*,*R*)-**7** (c = 10^{-5} M in $CHCl_3/CH_3CN$, 1:1, v/v, 298 K).

Table S23. Kinetic data for the threading of host (+)-**Zn4a** onto guest (*R*,*R*)-**7**.

**Entry**	**Host**	**Guest**	k_{on} **(× 10³ M⁻¹·s⁻¹)**	R^2
1	(+)-**Zn4a (positive Soret)**	(*R*,*R*)-**7**	1.08	0.999
2	(+)-**Zn4a (positive Soret)**	(*R*,*R*)-**7**	1.03	0.999
3	(+)-**Zn4a (positive Soret)**	(*R*,*R*)-**7**	1.04	0.999
**AVERAGE**	(+)-**Zn4a (positive Soret)**	(*R*,*R*)-**7**	1.05	0.999

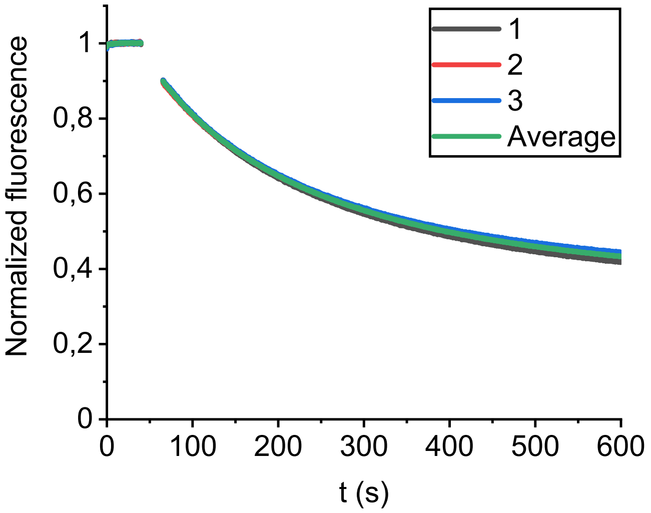

Figure S52. Normalized fluorescence intensity of host (+)-**Zn4a** as a function of time after the addition (t = 50 s) of 1 equivalent of guest (*S,S*)-**7** (c = 10^{-5} M in $CHCl_3/CH_3CN$, 1:1, v/v, 298 K). The experiment was performed in triplicate.

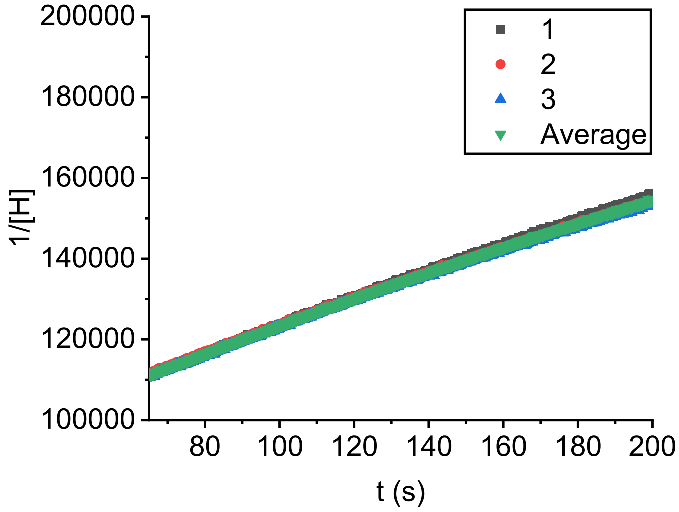

Figure S53. Second-order kinetics plots for the complexation of host (+)-**Zn4a** to guest (*S,S*)-**7** (c = 10^{-5} M in $CHCl_3/CH_3CN$, 1:1, v/v, 298 K).

Table S24. Kinetic data for the threading of host (+)-**Zn4a** onto guest (*S,S*)-**7**.

**Entry**	**Host**	**Guest**	**k_{on} (× 10^2 M^{-1}·s^{-1})**	**R^2**
1	(+)-**Zn4a (positive Soret)**	(*S,S*)-**7**	3.31	0.999
2	(+)-**Zn4a (positive Soret)**	(*S,S*)-**7**	3.16	0.999
3	(+)-**Zn4a (positive Soret)**	(*S,S*)-**7**	3.18	0.999
**AVERAGE**	(+)-**Zn4a (positive Soret)**	(*S,S*)-**7**	3.22	0.999

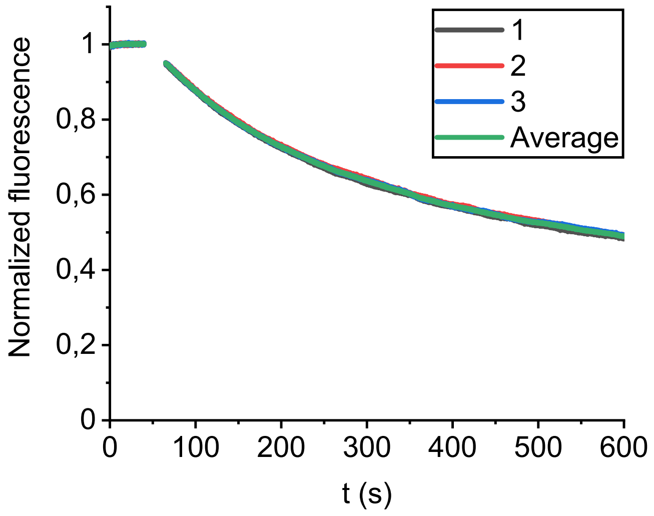

Figure S54. Normalized fluorescence intensity of host (−)-**Zn4b** as a function of time after the addition (t = 50 s) of 1 equivalent of guest (*R,R*)-**7** (c = 10^{-5} M in $CHCl_3/CH_3CN$, 1:1, v/v, 298 K). The experiment was performed in triplicate.

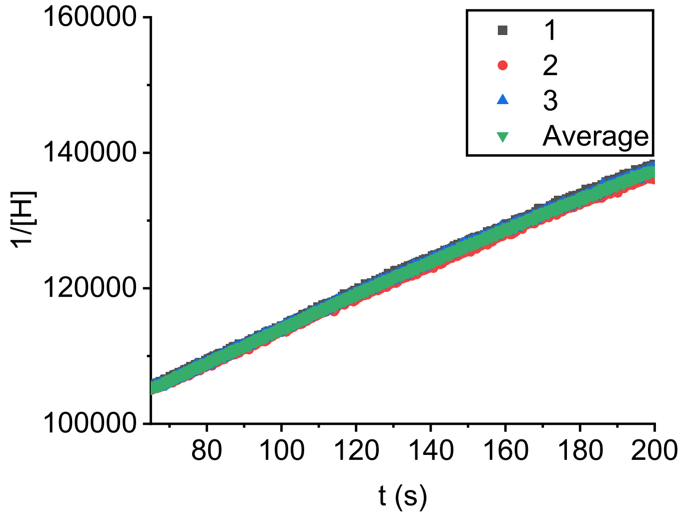

Figure S55. Second-order kinetics plots for the complexation of host (−)-**Zn4b** to guest (*R*,*R*)-**7** (c = 10^{-5} M in $CHCl_3/CH_3CN$, 1:1, v/v, 298 K).

Table S25. Kinetic data for the threading of host (−)-**Zn4b** onto guest (*R*,*R*)-**7**.

**Entry**	**Host**	**Guest**	k_{on} **(× 10^2 $M^{-1}\cdot s^{-1}$)**	R^2
1	(−)-**Zn4b (negative Soret)**	(*R*,*R*)-**7**	2.44	0.999
2	(−)-**Zn4b (negative Soret)**	(*R*,*R*)-**7**	2.38	0.998
3	(−)-**Zn4b (negative Soret)**	(*R*,*R*)-**7**	2.42	0.998
**AVERAGE**	(−)-**Zn4b (negative Soret)**	(*R*,*R*)-**7**	2.41	0.999

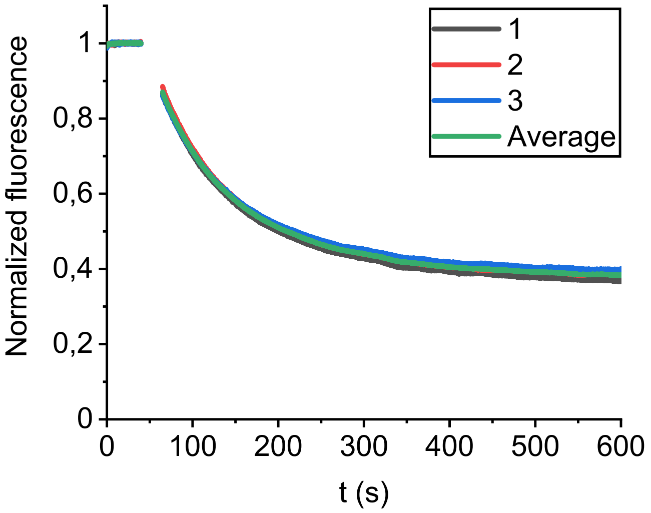

Figure S56. Normalized fluorescence intensity of host (−)-**Zn4b** as a function of time after the addition (t = 50 s) of 1 equivalent of guest (*S,S*)-**7** (c = 10^{-5} M in $CHCl_3/CH_3CN$, 1:1, v/v, 298 K). The experiment was performed in triplicate.

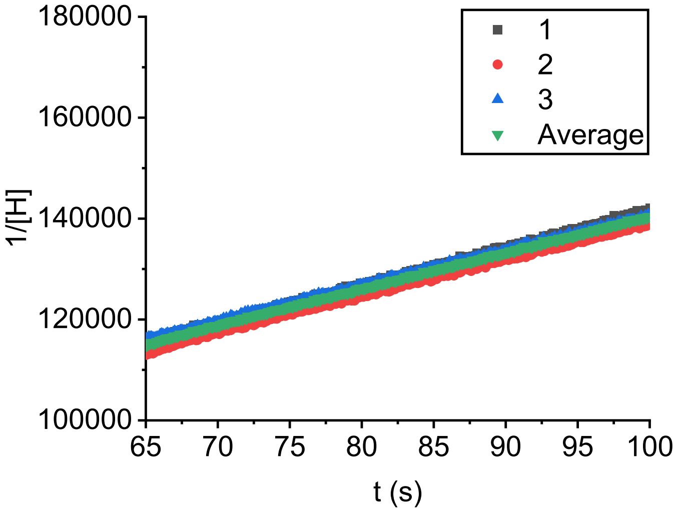

Figure S57. Second-order kinetics plots for the complexation of host (−)-**Zn4b** to guest (*S,S*)-**7** (c = 10^{-5} M in $CHCl_3/CH_3CN$, 1:1, v/v, 298 K).

Table S26. Kinetic data for the threading of host (−)-**Zn4b** onto guest (*S,S*)-**7**.

**Entry**	**Host**	**Guest**	k_{on} **(× 10^2 M^{-1}·s^{-1})**	**R^2**
1	(−)-**Zn4b (negative Soret)**	(*S,S*)-**7**	7.38	0.999
2	(−)-**Zn4b (negative Soret)**	(*S,S*)-**7**	7.26	0.998
3	(−)-**Zn4b (negative Soret)**	(*S,S*)-**7**	7.00	0.998
**AVERAGE**	(−)-**Zn4b (negative Soret)**	(*S,S*)-**7**	7.21	0.999

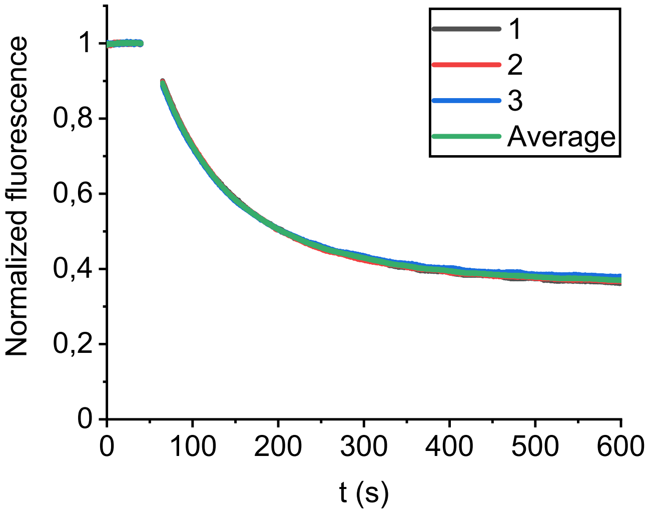

Figure S58. Normalized fluorescence intensity of host (+)-**Zn4b** as a function of time after the addition (t = 50 s) of 1 equivalent of guest (*R,R*)-**7** (c = 10^{-5} M in $CHCl_3/CH_3CN$, 1:1, v/v, 298 K). The experiment was performed in triplicate.

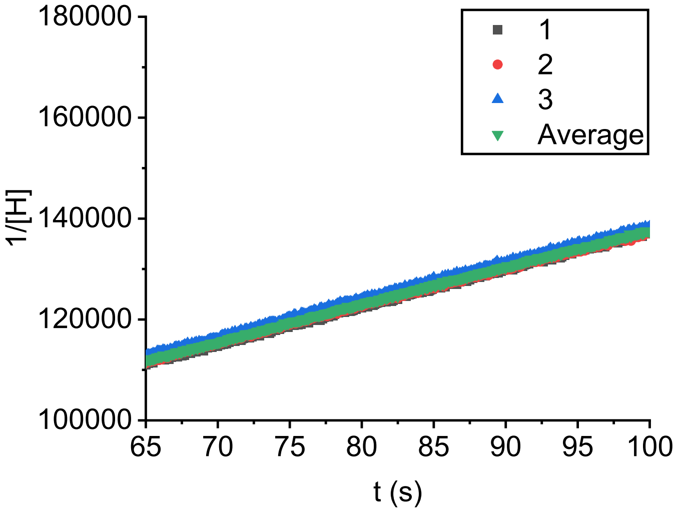

Figure S59. Second-order kinetics plots for the complexation of host (+)-**Zn4b** to guest (*R*,*R*)-**7** (c = 10^{-5} M in $CHCl_3/CH_3CN$, 1:1, v/v, 298 K).

Table S27. Kinetic data for the threading of host (+)-**Zn4b** onto guest (*R,R*)-**7**.

**Entry**	**Host**	**Guest**	**k_{on} (× 10^2 $M^{-1}·s^{-1}$)**	**R^2**
1	(+)-**Zn4b (positive Soret)**	(*R*,*R*)-**7**	7.47	0.999
2	(+)-**Zn4b (positive Soret)**	(*R*,*R*)-**7**	7.33	0.999
3	(+)-**Zn4b (positive Soret)**	(*R*,*R*)-**7**	7.36	0.998
**AVERAGE**	(+)-**Zn4b (positive Soret)**	(*R*,*R*)-**7**	7.39	0.999

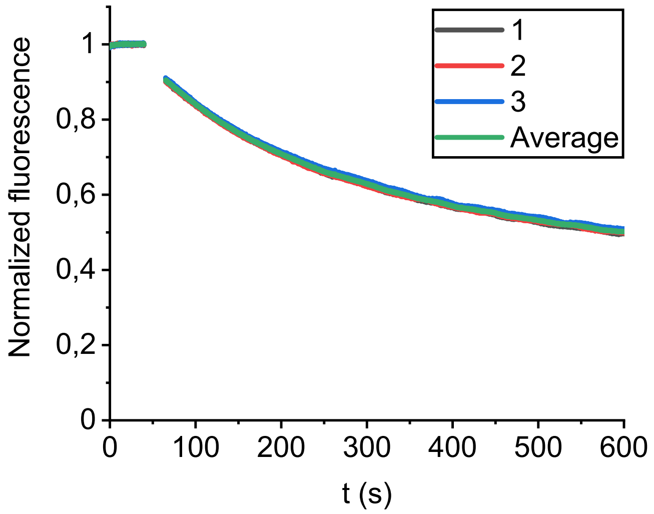

Figure S60. Normalized fluorescence intensity of host (+)-**Zn4b** as a function of time after the addition (t = 50 s) of 1 equivalent of guest (*S,S*)-**7** (c = 10^{-5} M in $CHCl_3/CH_3CN$, 1:1, v/v, 298 K). The experiment was performed in triplicate.

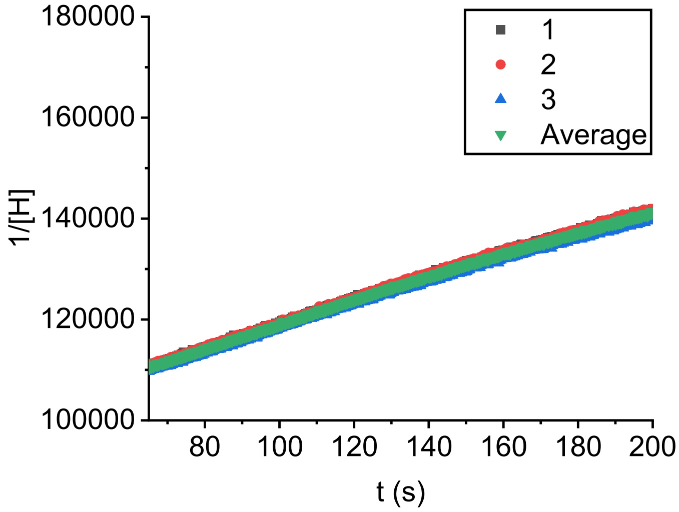

Figure S61. Second-order kinetics plots for the complexation of host (+)-**Zn4b** to guest (*S,S*)-**7** (c = 10^{-5} M in $CHCl_3/CH_3CN$, 1:1, v/v, 298 K).

Table S28. Kinetic data for the threading of host (+)-**Zn4b** onto guest (*S,S*)-**7**.

**Entry**	**Host**	**Guest**	**k_{on} (× 10^2 $M^{-1}·s^{-1}$)**	**R^2**
1	(+)-**Zn4b (positive Soret)**	(*S,S*)-**7**	2.32	0.998
2	(+)-**Zn4b (positive Soret)**	(*S,S*)-**7**	2.31	0.998
3	(+)-**Zn4b (positive Soret)**	(*S,S*)-**7**	2.26	0.997
**AVERAGE**	(+)-**Zn4b (positive Soret)**	(*S,S*)-**7**	2.30	0.998

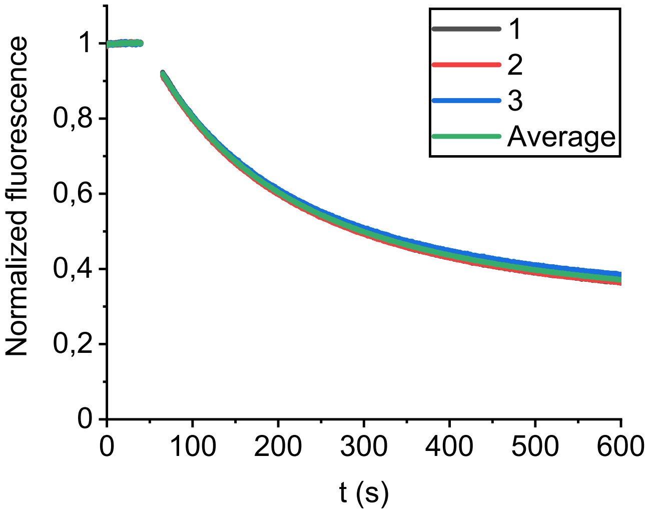

Figure S62. Normalized fluorescence intensity of host (−)-**Zn5a** as a function of time after the addition (t = 50 s) of 1 equivalent of guest (*R,R*)-**7** (c = 10^{-5} M in $CHCl_3/CH_3CN$, 1:1, v/v, 298 K). The experiment was performed in triplicate.

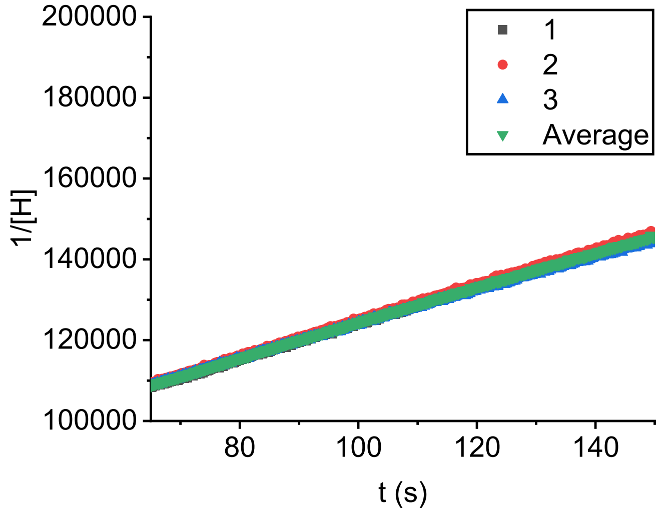

Figure S63. Second-order kinetics plots for the complexation of host (−)-**Zn5a** to guest (*R,R*)-**7** (c = 10^{-5} M in $CHCl_3/CH_3CN$, 1:1, v/v, 298 K).

Table S29. Kinetic data for the threading of host (−)-**Zn5a** onto guest (*R,R*)-**7**.

Entry	Host	Guest	k_{on} (× 10^2 $M^{-1}\cdot s^{-1}$)	R^2
1	(−)-**Zn5a (negative Soret)**	(*R*,*R*)-**7**	4.44	0.999
2	(−)-**Zn5a (negative Soret)**	(*R*,*R*)-**7**	4.40	0.999
3	(−)-**Zn5a (negative Soret)**	(*R*,*R*)-**7**	4.23	0.999
**AVERAGE**	(−)-**Zn5a (negative Soret)**	(*R*,*R*)-**7**	4.36	0.999

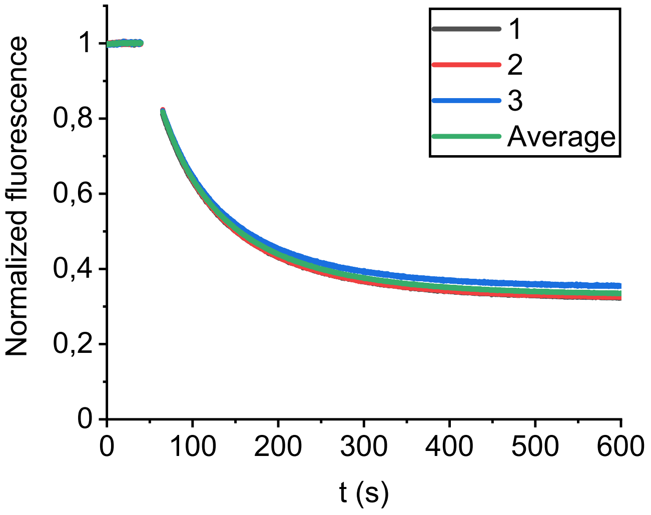

Figure S64. Normalized fluorescence intensity of host (−)-**Zn5a** as a function of time after the addition (t = 50 s) of 1 equivalent of guest (*S,S*)-**7** (c = 10^{-5} M in $CHCl_3/CH_3CN$, 1:1, v/v, 298 K). The experiment was performed in triplicate.

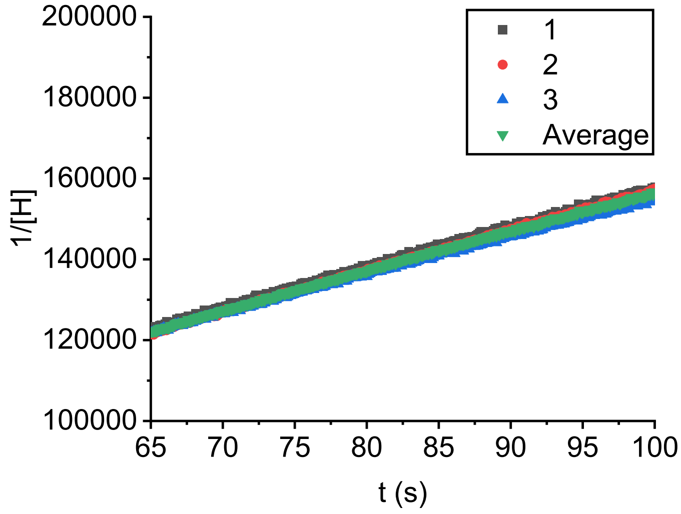

Figure S65 Second-order kinetics plots for the complexation of host (−)-**Zn5a** to guest (*S,S*)-**7** (c = 10^{-5} M in $CHCl_3/CH_3CN$, 1:1, v/v, 298 K).

Table S30. Kinetic data for the threading of host (−)-**Zn5a** onto guest (*S,S*)-**7**.

Entry	Host	Guest	k_{on} (× 10^2 $M^{-1}{\cdot}s^{-1}$)	R^2
1	(−)-**Zn5a (negative Soret)**	(*S,S*)-**7**	9.94	0.998
2	(−)-**Zn5a (negative Soret)**	(*S,S*)-**7**	10.08	0.999
3	(−)-**Zn5a (negative Soret)**	(*S,S*)-**7**	9.41	0.998
**AVERAGE**	(−)-**Zn5a (negative Soret)**	(*S,S*)-**7**	9.81	0.999

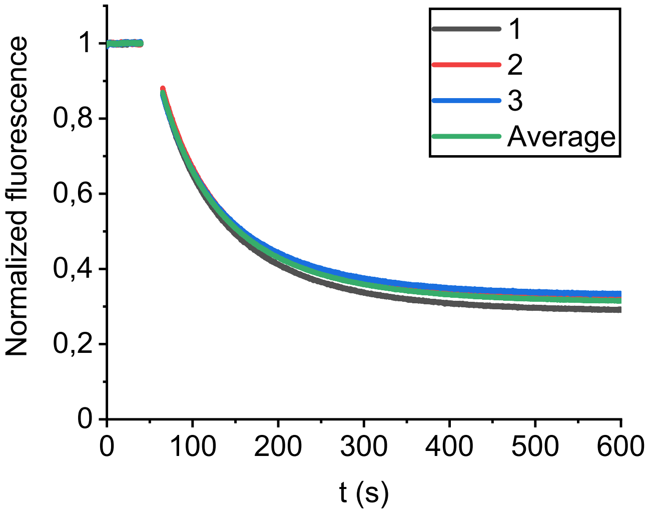

Figure S66. Normalized fluorescence intensity of host (+)-**Zn5a** as a function of time after the addition (t = 50 s) of 1 equivalent of guest (*R,R*)-**7** (c = 10^{-5} M in $CHCl_3/CH_3CN$, 1:1, v/v, 298 K). The experiment was performed in triplicate.

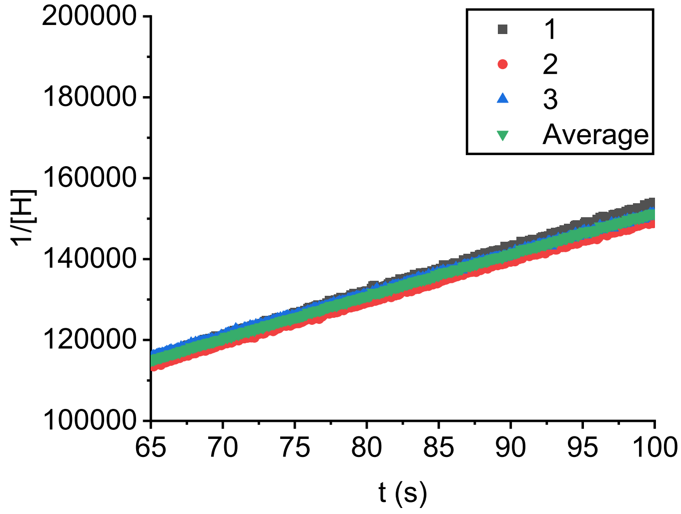

Figure S67. Second-order kinetics plots for the complexation of host (+)-**Zn5a** to guest (*R,R*)-**7** (c = 10^{-5} M in $CHCl_3/CH_3CN$, 1:1, v/v, 298 K).

Table S31. Kinetic data for the threading of host (+)-**Zn5a** onto guest (*R,R*)-**7**.

**Entry**	**Host**	**Guest**	k_{on} **(× 10^3 $M^{-1}\cdot s^{-1}$)**	R^2
1	(+)-**Zn5a (positive Soret)**	(*R*,*R*)-**7**	1.09	0.999
2	(+)-**Zn5a (positive Soret)**	(*R*,*R*)-**7**	1.03	0.999
3	(+)-**Zn5a (positive Soret)**	(*R*,*R*)-**7**	1.01	0.999
**AVERAGE**	(+)-**Zn5a (positive Soret)**	(*R*,*R*)-**7**	1.04	0.999

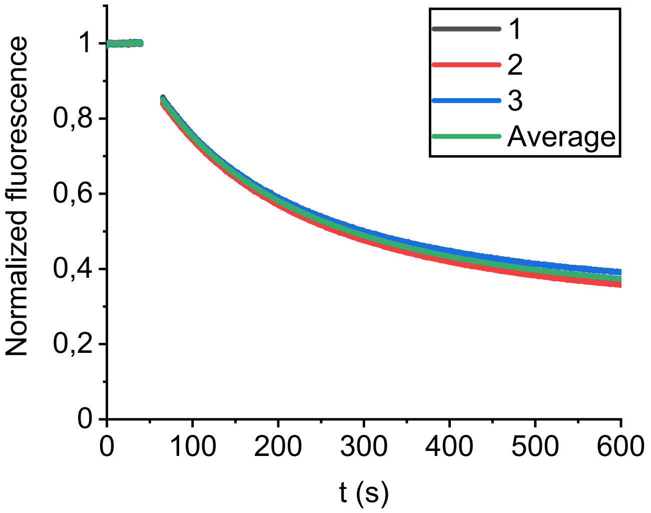

Figure S68. Normalized fluorescence intensity of host (+)-**Zn5a** as a function of time after the addition (t = 50 s) of 1 equivalent of guest (*S,S*)-**7** (c = 10^{-5} M in $CHCl_3/CH_3CN$, 1:1, v/v, 298 K). The experiment was performed in triplicate.

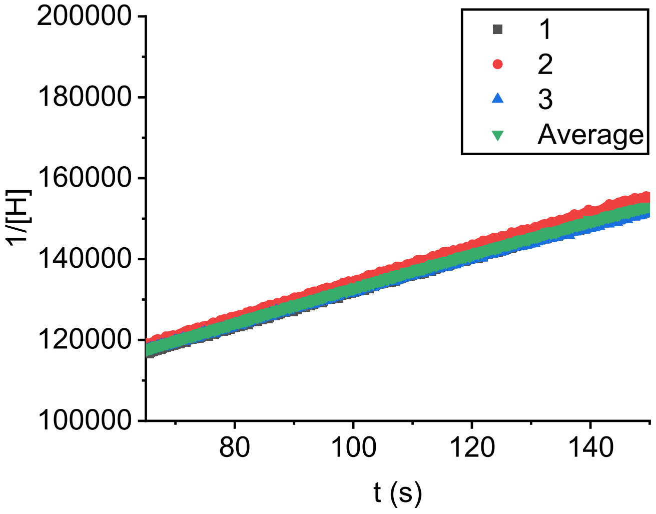

Figure S69. Second-order kinetics plots for the complexation of host (+)-**Zn5a** to guest (*S,S*)-**7** (c = 10^{-5} M in $CHCl_3/CH_3CN$, 1:1, v/v, 298 K).

Table S32. Kinetic data for the threading of host (+)-**Zn5a** onto guest (*S,S*)-**7**.

**Entry**	**Host**	**Guest**	k_{on} **(× 10^2 M^{-1}·s^{-1})**	**R^2**
1	(+)-**Zn5a (positive Soret)**	(*S,S*)-**7**	4.19	0.999
2	(+)-**Zn5a (positive Soret)**	(*S,S*)-**7**	4.33	0.999
3	(+)-**Zn5a (positive Soret)**	(*S,S*)-**7**	4.07	0.998
**AVERAGE**	(+)-**Zn5a (positive Soret)**	(*S,S*)-**7**	4.20	0.999

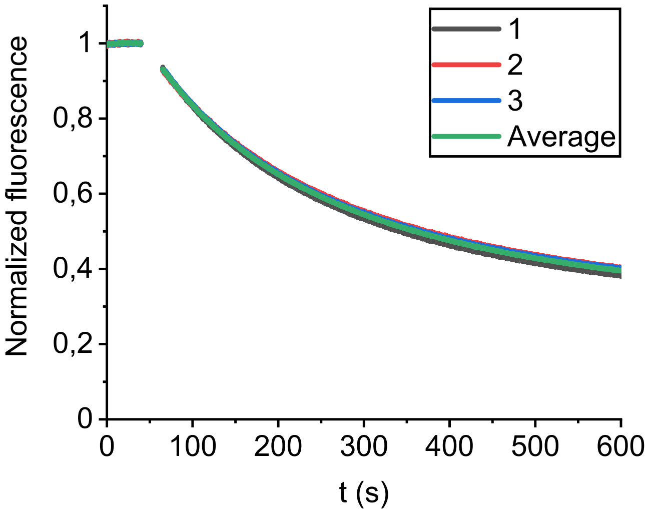

Figure S70. Normalized fluorescence intensity of host (−)-**Zn5b** as a function of time after the addition (t = 50 s) of 1 equivalent of guest (*R,R*)-**7** (c = 10^{-5} M in $CHCl_3/CH_3CN$, 1:1, v/v, 298 K). The experiment was performed in triplicate.

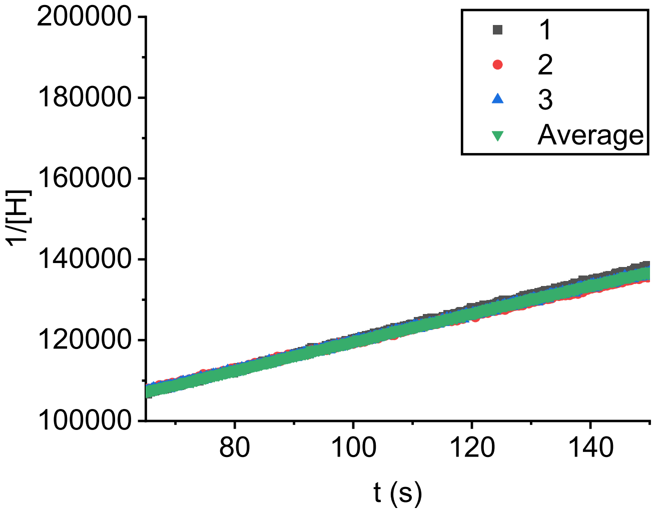

Figure S71. Second-order kinetics plots for the complexation of host (−)-**Zn5b** to guest (*R*,*R*)-**7** (c = 10^{-5} M in $CHCl_3/CH_3CN$, 1:1, v/v, 298 K).

Table S33. Kinetic data for the threading of host (−)-**Zn5b** onto guest (*R*,*R*)-**7**.

**Entry**	**Host**	**Guest**	k_{on} **(× 10^2 M^{-1}·s^{-1})**	**R^2**
1	(−)-**Zn5b (negative Soret)**	(*R*,*R*)-**7**	3.67	0.999
2	(−)-**Zn5b (negative Soret)**	(*R*,*R*)-**7**	3.36	0.999
3	(−)-**Zn5b (negative Soret)**	(*R*,*R*)-**7**	3.47	0.999
**AVERAGE**	(−)-**Zn5b (negative Soret)**	(*R*,*R*)-**7**	3.50	0.999

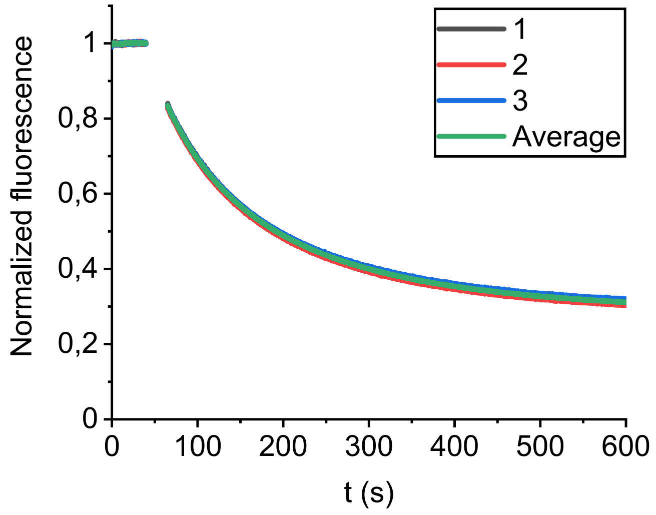

Figure S72. Normalized fluorescence intensity of host (−)-**Zn5b** as a function of time after the addition (t = 50 s) of 1 equivalent of guest (*S,S*)-**7** (c = 10^{-5} M in $CHCl_3/CH_3CN$, 1:1, v/v, 298 K). The experiment was performed in triplicate.

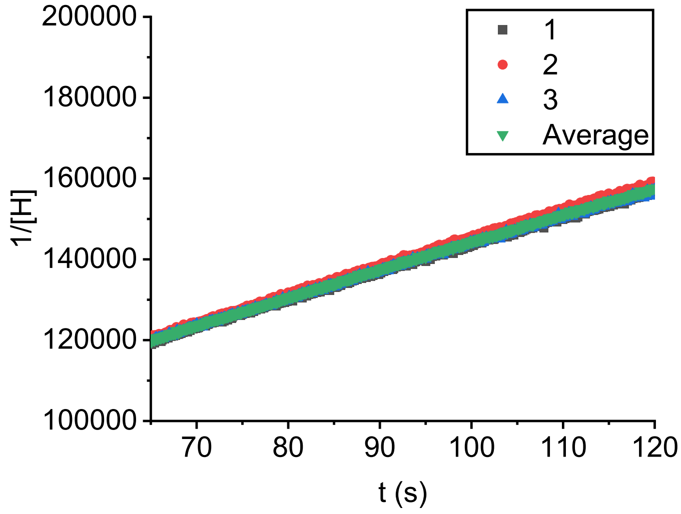

Figure S73. Second-order kinetics plots for the complexation of host (−)-**Zn5b** to guest (*S,S*)-**7** (c = 10^{-5} M in $CHCl_3/CH_3CN$, 1:1, v/v, 298 K).

Table S34. Kinetic data for the threading of host (−)-**Zn5b** onto guest (*S,S*)-**7**.

**Entry**	**Host**	**Guest**	k_{on} **(× 10^2 $M^{-1}\cdot s^{-1}$)**	**R^2**
1	(−)-**Zn5b (negative Soret)**	(*S,S*)-**7**	6.78	0.999
2	(−)-**Zn5b (negative Soret)**	(*S,S*)-**7**	6.99	0.999
3	(−)-**Zn5b (negative Soret)**	(*S,S*)-**7**	6.75	0.999
**AVERAGE**	(−)-**Zn5b (negative Soret)**	(*S,S*)-**7**	6.84	0.999

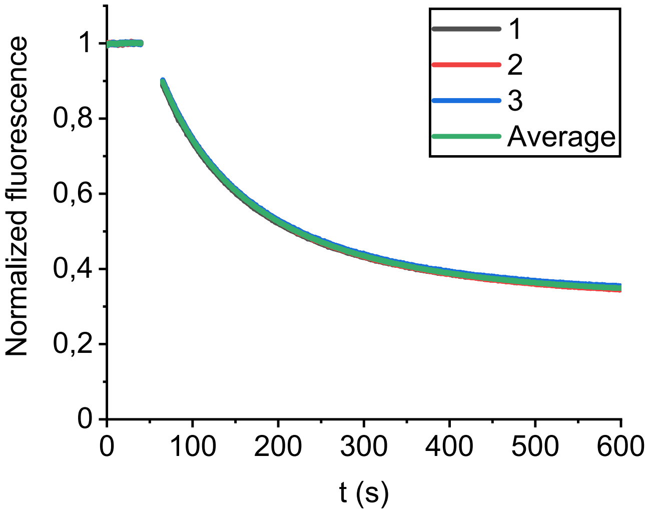

Figure S74. Normalized fluorescence intensity of host (+)-**Zn5b** as a function of time after the addition (t = 50 s) of 1 equivalent of guest (*R,R*)-**7** (c = 10^{-5} M in $CHCl_3/CH_3CN$, 1:1, v/v, 298 K). The experiment was performed in triplicate.

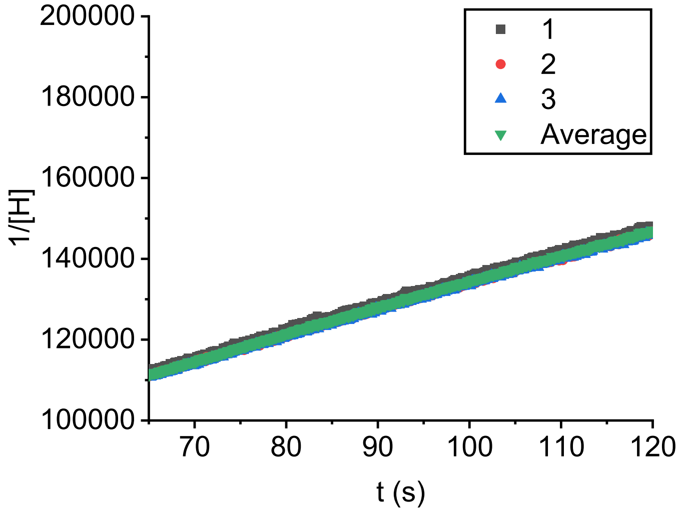

Figure S75. Second-order kinetics plots for the complexation of host (+)-**Zn5b** to guest (*R,R*)-**7** (c = 10^{-5} M in $CHCl_3/CH_3CN$, 1:1, v/v, 298 K).

Table S35. Kinetic data for the threading of host (+)-**Zn5b** onto guest (*R,R*)-**7**.

**Entry**	**Host**	**Guest**	k_{on} **(× 10² M⁻¹·s⁻¹)**	R^2
1	(+)-**Zn5b (positive Soret)**	(*R,R*)-**7**	6.54	0.998
2	(+)-**Zn5b (positive Soret)**	(*R,R*)-**7**	6.46	0.999
3	(+)-**Zn5b (positive Soret)**	(*R,R*)-**7**	6.47	0.999
**AVERAGE**	(+)-**Zn5b (positive Soret)**	(*R,R*)-**7**	6.49	0.999

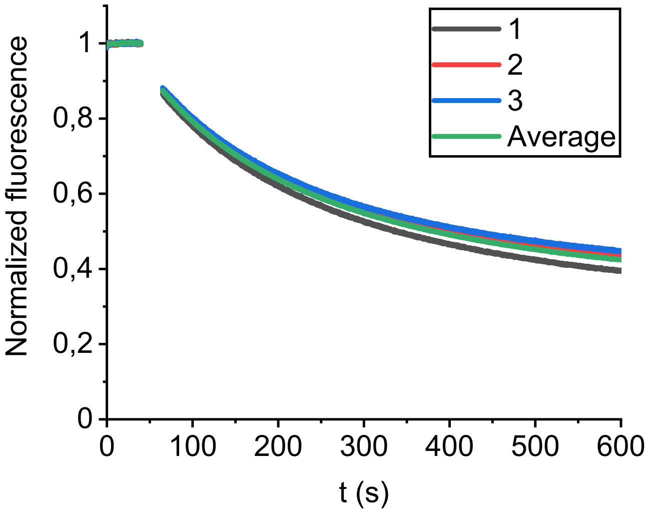

Figure S76. Normalized fluorescence intensity of host (+)-**Zn5b** as a function of time after the addition (t = 50 s) of 1 equivalent of guest (*S,S*)-**7** (c = 10^{-5} M in $CHCl_3/CH_3CN$, 1:1, v/v, 298 K). The experiment was performed in triplicate.

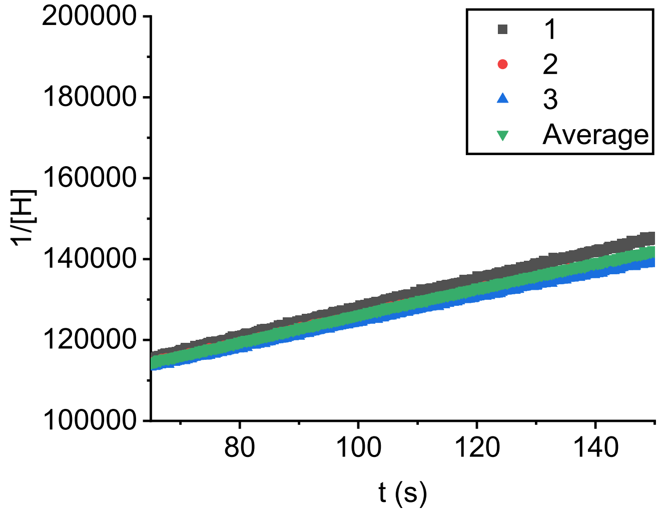

Figure S77. Second-order kinetics plots for the complexation of host (+)-**Zn5b** to guest (*S,S*)-**7** (c = 10^{-5} M in $CHCl_3/CH_3CN$, 1:1, v/v, 298 K).

Table S36. Kinetic data for the threading of host (+)-**Zn5b** onto guest (*S,S*)-**7**.

**Entry**	**Host**	**Guest**	k_{on} **(× 10² M⁻¹·s⁻¹)**	R^2
1	(+)-**Zn5b (positive Soret)**	(*S,S*)-**7**	3.50	0.998
2	(+)-**Zn5b (positive Soret)**	(*S,S*)-**7**	3.19	0.998
3	(+)-**Zn5b (positive Soret)**	(*S,S*)-**7**	3.09	0.998
**AVERAGE**	(+)-**Zn5b (positive Soret)**	(*S,S*)-**7**	3.26	0.999

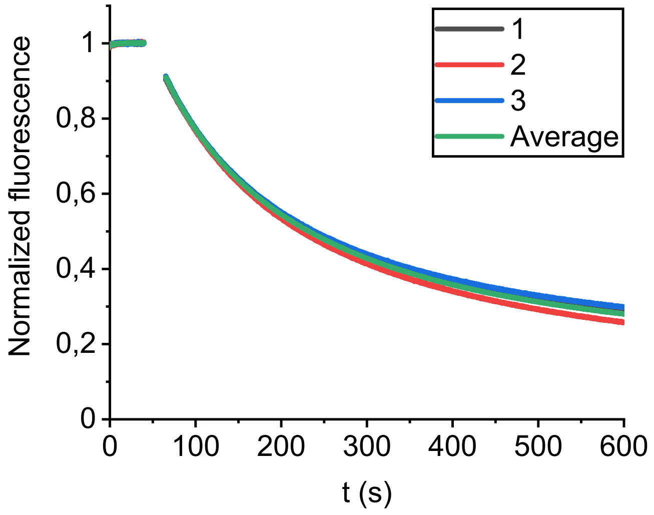

Figure S78. Normalized fluorescence intensity of host (−)-**Zn6a** as a function of time after the addition (t = 50 s) of 1 equivalent of guest (*R,R*)-**7** (c = 10^{-5} M in $CHCl_3/CH_3CN$, 1:1, v/v, 298 K). The experiment was performed in triplicate.

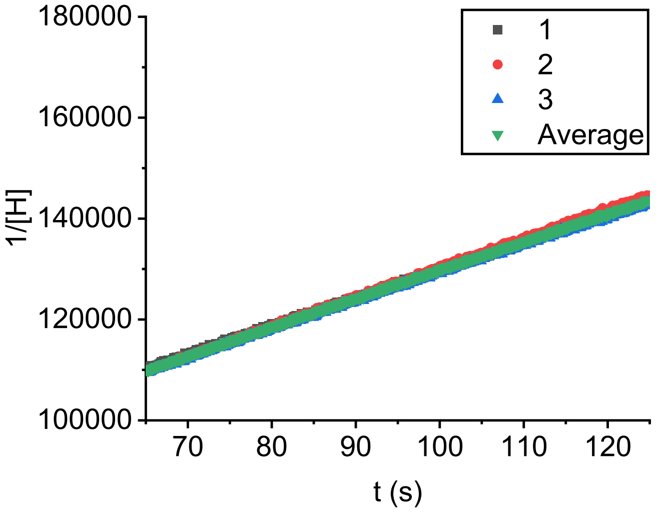

Figure S79 Second-order kinetics plots for the complexation of host (−)-**Zn6a** to guest (*R,R*)-**7** (c = 10^{-5} M in $CHCl_3/CH_3CN$, 1:1, v/v, 298 K).

Table S37. Kinetic data for the threading of host (−)-**Zn6a** onto guest (*R*,*R*)-**7**.

**Entry**	**Host**	**Guest**	k_{on} **(× 10^2 $M^{-1}\cdot s^{-1}$)**	R^2
1	(−)-**Zn6a (negative Soret)**	(*R*,*R*)-**7**	5.51	0.999
2	(−)-**Zn6a (negative Soret)**	(*R*,*R*)-**7**	5.81	0.999
3	(−)-**Zn6a (negative Soret)**	(*R*,*R*)-**7**	5.59	0.999
**AVERAGE**	(−)-**Zn6a (negative Soret)**	(*R*,*R*)-**7**	5.64	0.999

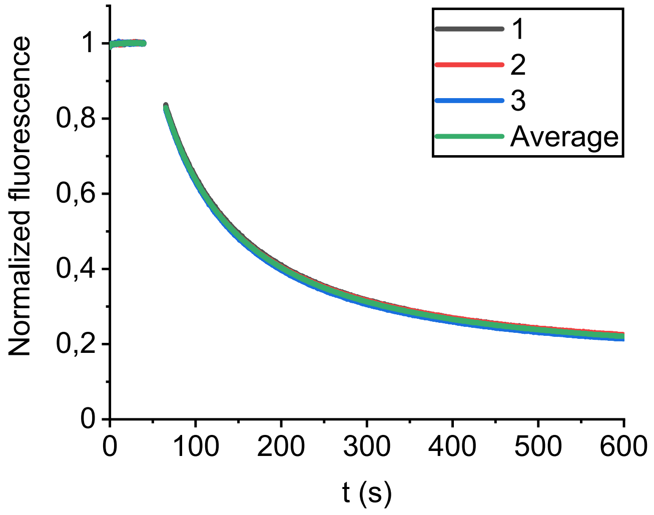

Figure S80. Normalized fluorescence intensity of host (−)-**Zn6a** as a function of time after the addition (t = 50 s) of 1 equivalent of guest (*S,S*)-**7** (c = 10^{-5} M in $CHCl_3/CH_3CN$, 1:1, v/v, 298 K). The experiment was performed in triplicate.

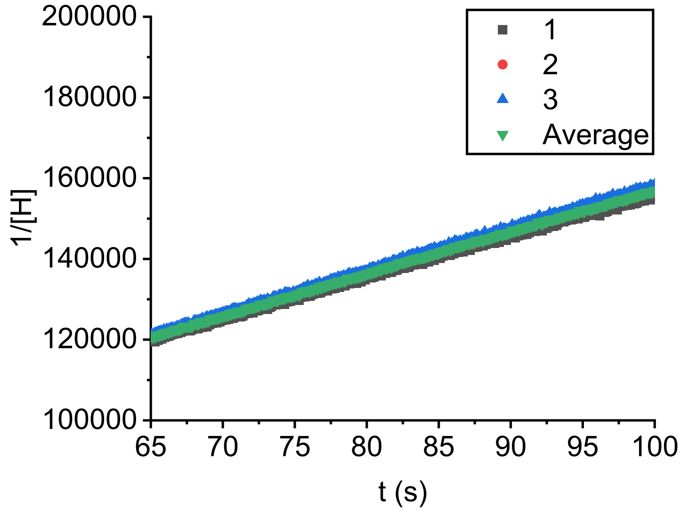

Figure S81. Second-order kinetics plots for the complexation of host (−)-**Zn6a** to guest (*S,S*)-**7** (c = 10^{-5} M in $CHCl_3/CH_3CN$, 1:1, v/v, 298 K).

Table S38. Kinetic data for the threading of host (−)-**Zn6a** onto guest (*S,S*)-**7**.

**Entry**	**Host**	**Guest**	k_{on} **(× 10^3 $M^{-1}\cdot s^{-1}$)**	R^2
1	(−)-**Zn6a (negative Soret)**	(*S,S*)-**7**	1.02	0.999
2	(−)-**Zn6a (negative Soret)**	(*S,S*)-**7**	1.04	0.999
3	(−)-**Zn6a (negative Soret)**	(*S,S*)-**7**	1.06	0.999
**AVERAGE**	(−)-**Zn6a (negative Soret)**	(*S,S*)-**7**	1.04	0.999

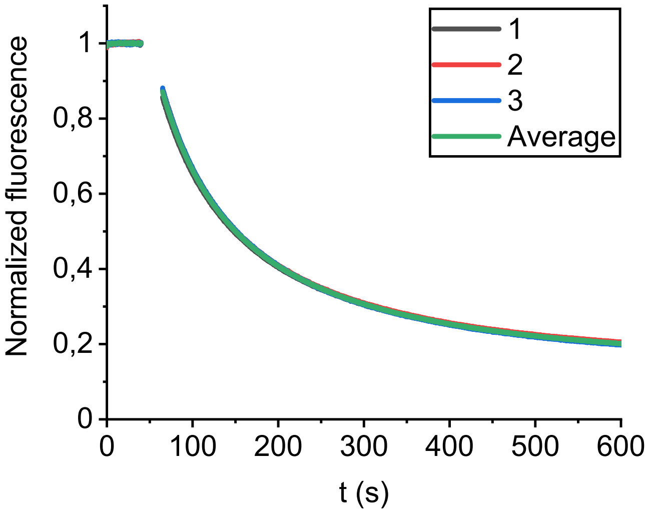

Figure S82. Normalized fluorescence intensity of host (+)-**Zn6a** as a function of time after the addition (t = 50 s) of 1 equivalent of guest (*R,R*)-**7** (c = 10^{-5} M in $CHCl_3/CH_3CN$, 1:1, v/v, 298 K). The experiment was performed in triplicate.

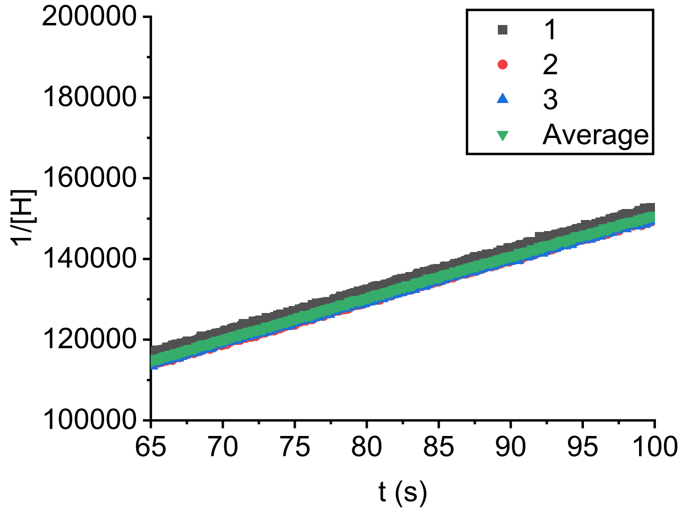

Figure S83. Second-order kinetics plots for the complexation of host (+)-**Zn6a** to guest (*R*,*R*)-**7** (c = 10^{-5} M in $CHCl_3/CH_3CN$, 1:1, v/v, 298 K).

Table S39. Kinetic data for the threading of host (+)-**Zn6a** onto guest (*R*,*R*)-**7**.

Entry	Host	Guest	k_{on} (× 10^3 $M^{-1}{\cdot}s^{-1}$)	R^2
1	(+)-**Zn6a (positive Soret)**	(*R*,*R*)-**7**	1.03	0.999
2	(+)-**Zn6a (positive Soret)**	(*R*,*R*)-**7**	1.04	0.999
3	(+)-**Zn6a (positive Soret)**	(*R*,*R*)-**7**	1.04	0.999
**AVERAGE**	(+)-**Zn6a (positive Soret)**	(*R*,*R*)-**7**	1.03	0.999

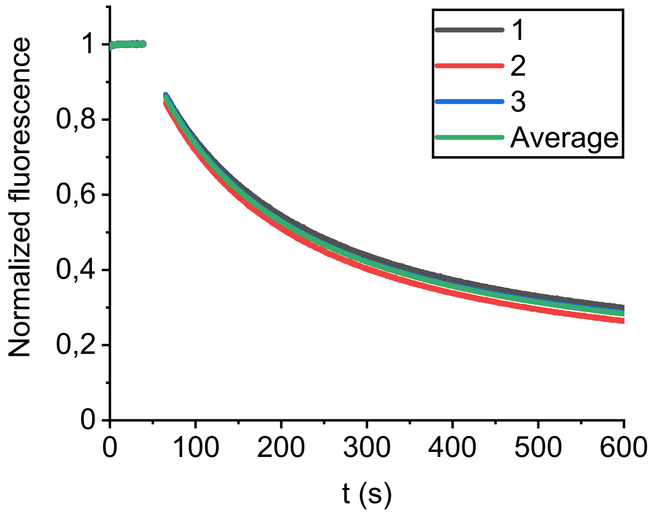

Figure S84. Normalized fluorescence intensity of host (+)-**Zn6a** as a function of time after the addition (t = 50 s) of 1 equivalent of guest (*S,S*)-**7** (c = 10^{-5} M in $CHCl_3/CH_3CN$, 1:1, v/v, 298 K). The experiment was performed in triplicate.

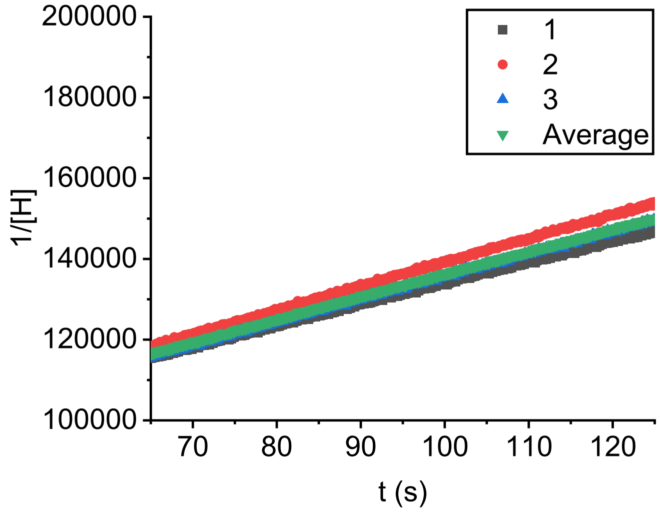

Figure S85. Second-order kinetics plots for the complexation of host (+)-**Zn6a** to guest (*S,S*)-**7** (c = 10^{-5} M in $CHCl_3/CH_3CN$, 1:1, v/v, 298 K).

Table S40. Kinetic data for the threading of host (+)-**Zn6a** onto guest (*S,S*)-**7**.

**Entry**	**Host**	**Guest**	**k_{on} (× 10^2 M^{-1}·s^{-1})**	**R^2**
1	(+)-**Zn6a (positive Soret)**	(*S,S*)-**7**	5.25	0.999
2	(+)-**Zn6a (positive Soret)**	(*S,S*)-**7**	5.90	0.999
3	(+)-**Zn6a (positive Soret)**	(*S,S*)-**7**	5.65	0.999
**AVERAGE**	(+)-**Zn6a (positive Soret)**	(*S,S*)-**7**	5.60	0.999

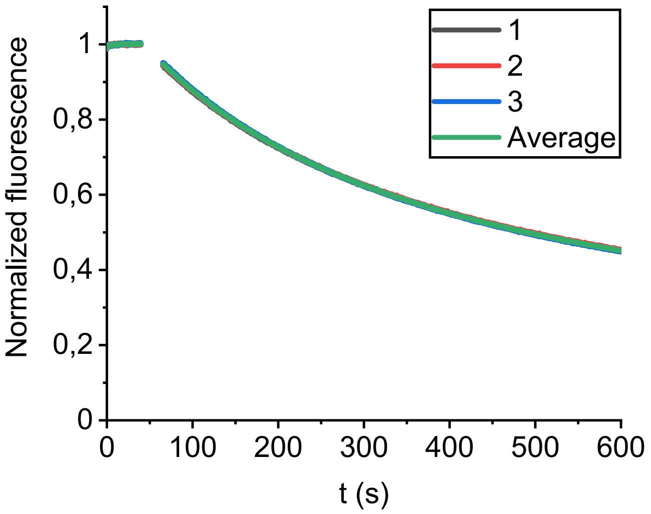

Figure S86. Normalized fluorescence intensity of host (−)-**Zn6b** as a function of time after the addition (t = 50 s) of 1 equivalent of guest (*R,R*)-**7** (c = 10^{-5} M in $CHCl_3/CH_3CN$, 1:1, v/v, 298 K). The experiment was performed in triplicate.

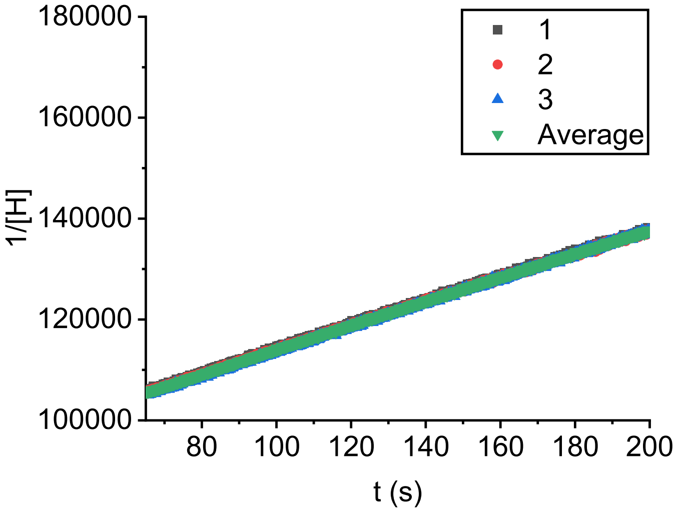

Figure S87. Second-order kinetics plots for the complexation of host (−)-**Zn6b** to guest (*R*,*R*)-**7** (c = 10^{-5} M in $CHCl_3/CH_3CN$, 1:1, v/v, 298 K).

Table S41. Kinetic data for the threading of host (−)-**Zn6b** onto guest (*R*,*R*)-**7**.

**Entry**	**Host**	**Guest**	k_{on} **(× 10^2 M^{-1}·s^{-1})**	**R^2**
1	(−)-**Zn6b (neg to pos Soret)**	(*R*,*R*)-**7**	2.38	0.999
2	(−)-**Zn6b (neg to pos Soret)**	(*R*,*R*)-**7**	2.37	0.999
3	(−)-**Zn6b (neg to pos Soret)**	(*R*,*R*)-**7**	2.41	0.999
**AVERAGE**	(−)-**Zn6b (neg to pos Soret)**	(*R*,*R*)-**7**	2.39	0.999

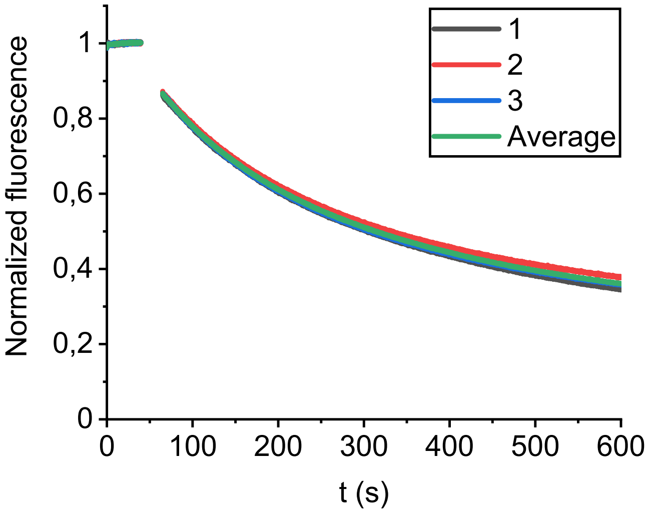

Figure S88. Normalized fluorescence intensity of host (−)-**Zn6b** as a function of time after the addition (t = 50 s) of 1 equivalent of guest (*S,S*)-**7** (c = 10^{-5} M in $CHCl_3/CH_3CN$, 1:1, v/v, 298 K). The experiment was performed in triplicate.

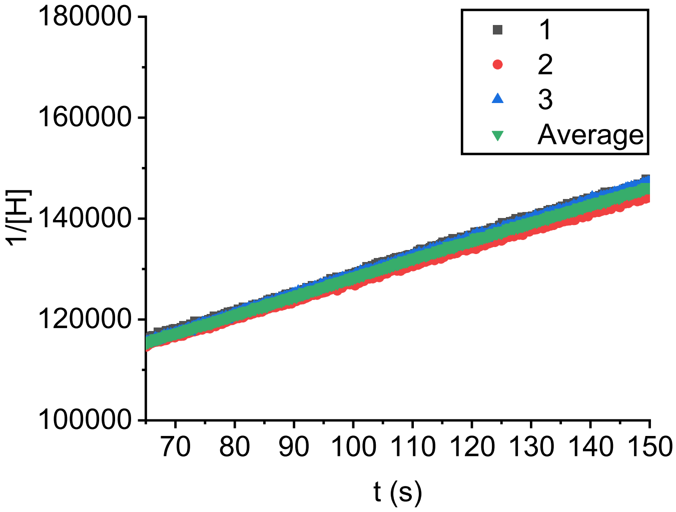

Figure S89. Second-order kinetics plots for the complexation of host (−)-**Zn6b** to guest (*S,S*)-**7** (c = 10^{-5} M in $CHCl_3/CH_3CN$, 1:1, v/v, 298 K).

Table S42. Kinetic data for the threading of host (−)-**Zn6b** onto guest (*R,R*)-**7**.

**Entry**	**Host**	**Guest**	k_{on} **(× 10^2 $M^{-1}·s^{-1}$)**	**R^2**
1	(−)-**Zn6b (neg to pos Soret)**	(*S,S*)-**7**	3.70	0.999
2	(−)-**Zn6b (neg to pos Soret)**	(*S,S*)-**7**	3.49	0.999
3	(−)-**Zn6b (neg to pos Soret)**	(*S,S*)-**7**	3.73	0.999
**AVERAGE**	(−)-**Zn6b (neg to pos Soret)**	(*S,S*)-**7**	3.64	0.999

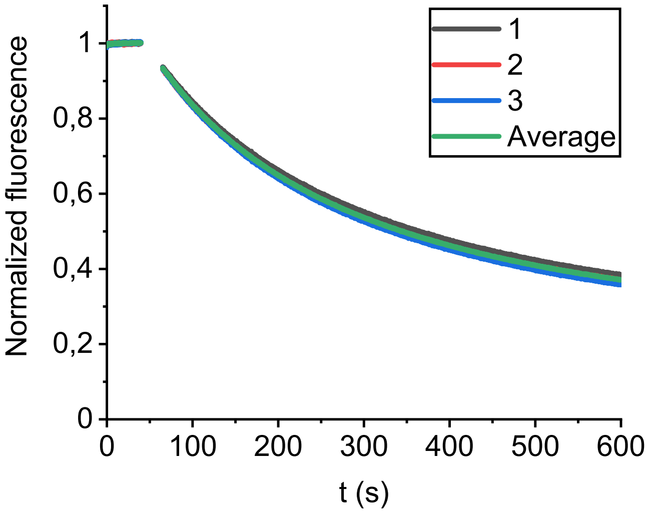

Figure S90. Normalized fluorescence intensity of host (+)-**Zn6b** as a function of time after the addition (t = 50 s) of 1 equivalent of guest (*R,R*)-**7** (c = 10^{-5} M in $CHCl_3/CH_3CN$, 1:1, v/v, 298 K). The experiment was performed in triplicate.

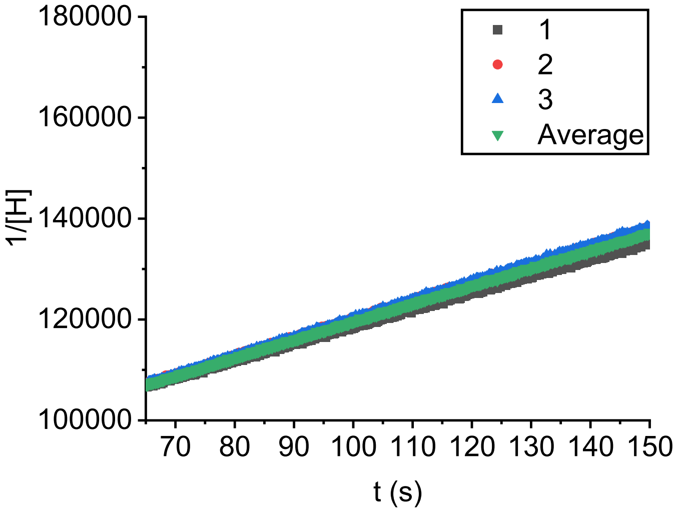

Figure S91. Second-order kinetics plots for the complexation of host (+)-**Zn6b** to guest (*R*,*R*)-**7** (c = 10^{-5} M in $CHCl_3/CH_3CN$, 1:1, v/v, 298 K).

Table S43. Kinetic data for the threading of host (+)-**Zn6b** onto guest (*R*,*R*)-**7**.

**Entry**	**Host**	**Guest**	k_{on} **(× 10^2 $M^{-1}\cdot s^{-1}$)**	**R^2**
1	(+)-**Zn6b (pos to neg Soret)**	(*R*,*R*)-**7**	3.36	0.999
2	(+)-**Zn6b (pos to neg Soret)**	(*R*,*R*)-**7**	3.62	0.999
3	(+)-**Zn6b (pos to neg Soret)**	(*R*,*R*)-**7**	3.66	0.999
**AVERAGE**	(+)-**Zn6b (pos to neg Soret)**	(*R*,*R*)-**7**	3.55	0.999

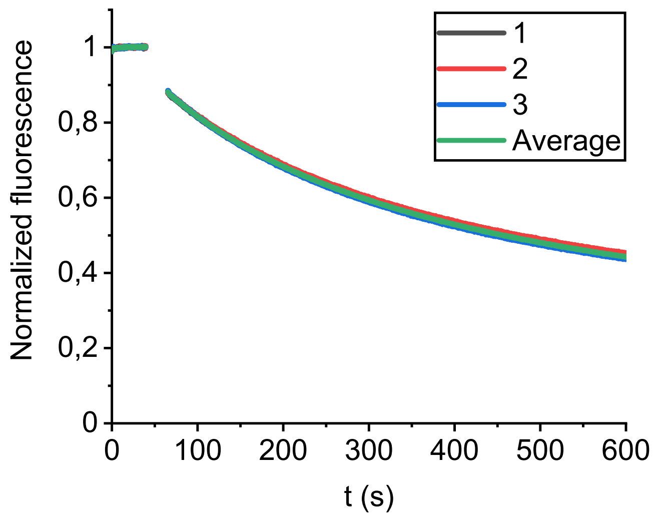

Figure S92. Normalized fluorescence intensity of host (+)-**Zn6b** as a function of time after the addition (t = 50 s) of 1 equivalent of guest (*S,S*)-**7** (c = 10^{-5} M in $CHCl_3/CH_3CN$, 1:1, v/v, 298 K). The experiment was performed in triplicate.

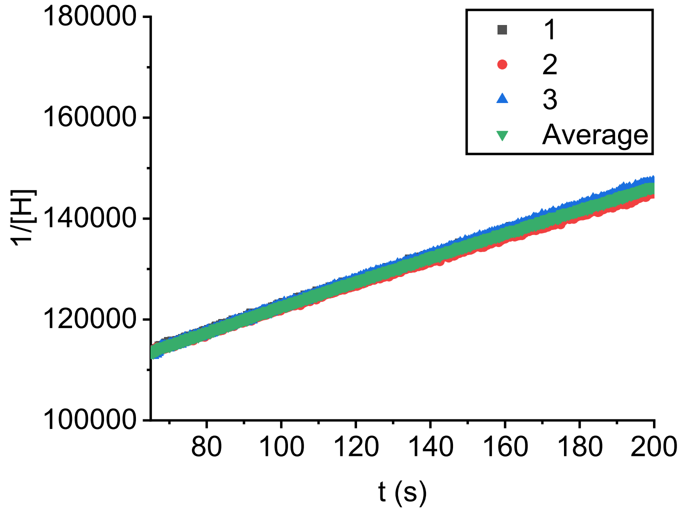

Figure S93. Second-order kinetics plots for the complexation of host (+)-**Zn6b** to guest (*S,S*)-**7** (c = 10^{-5} M in $CHCl_3/CH_3CN$, 1:1, v/v, 298 K).

Table S44. Kinetic data for the threading of host (+)-**Zn6b** onto guest (S,S)-**7**.

Entry	Host	Guest	k_{on} (× 10^2 $M^{-1}{\cdot}s^{-1}$)	R^2
1	(+)-**Zn6b (pos to neg Soret)**	(*S,S*)-**7**	2.43	0.999
2	(+)-**Zn6b (pos to neg Soret)**	(*S,S*)-**7**	2.35	0.999
3	(+)-**Zn6b (pos to neg Soret)**	(*S,S*)-**7**	2.49	0.999
**AVERAGE**	(+)-**Zn6b (pos to neg Soret)**	(*S,S*)-**7**	2.42	0.999

4.3. NMR spectra of synthesized compounds

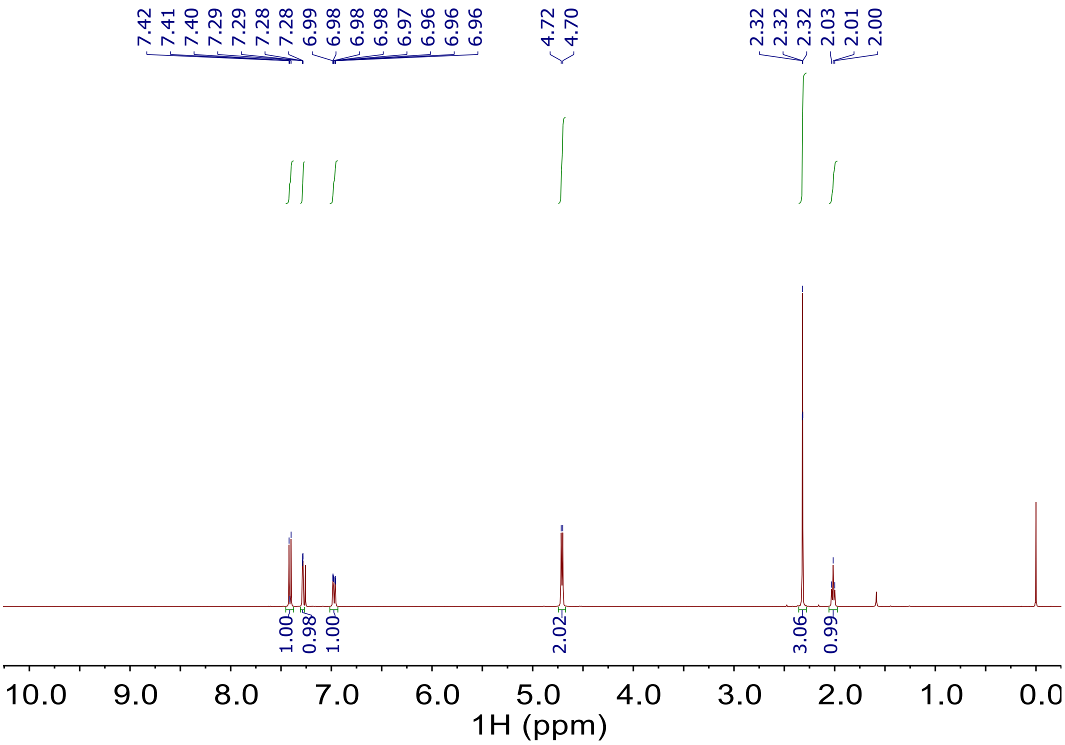

Figure S94. ^{1}H NMR spectrum (400 MHz, $CDCl_3$) of compound **9**.

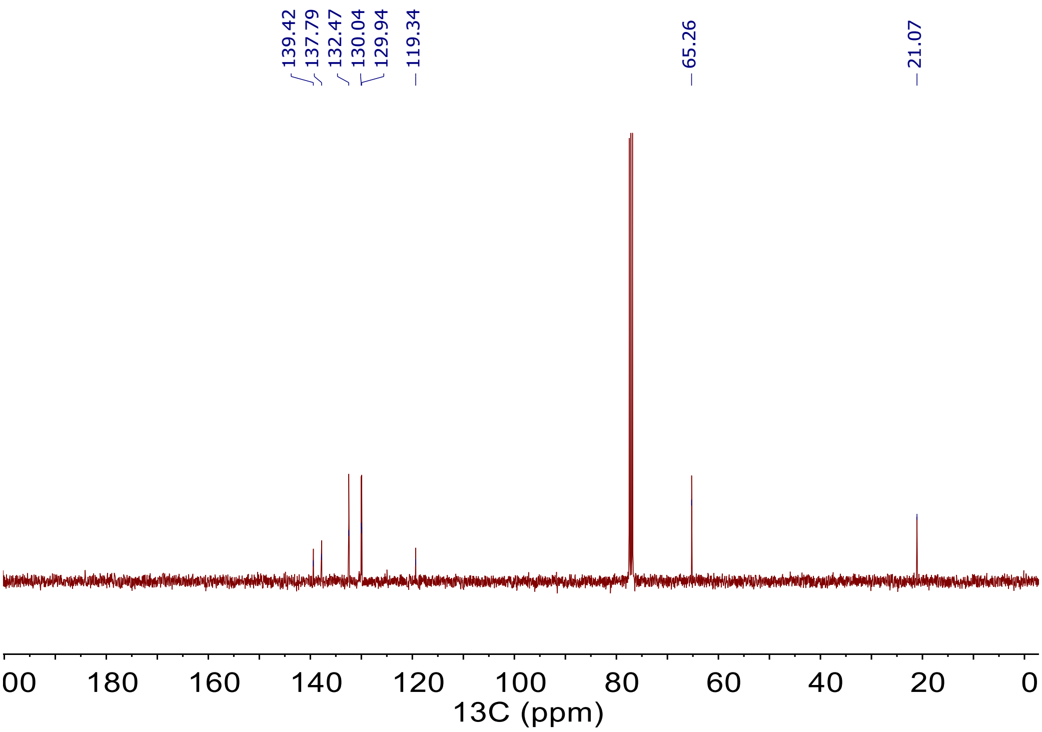

Figure S95. ^{13}C-^{1}H NMR spectrum (101 MHz, $CDCl_3$) of compound **9**.

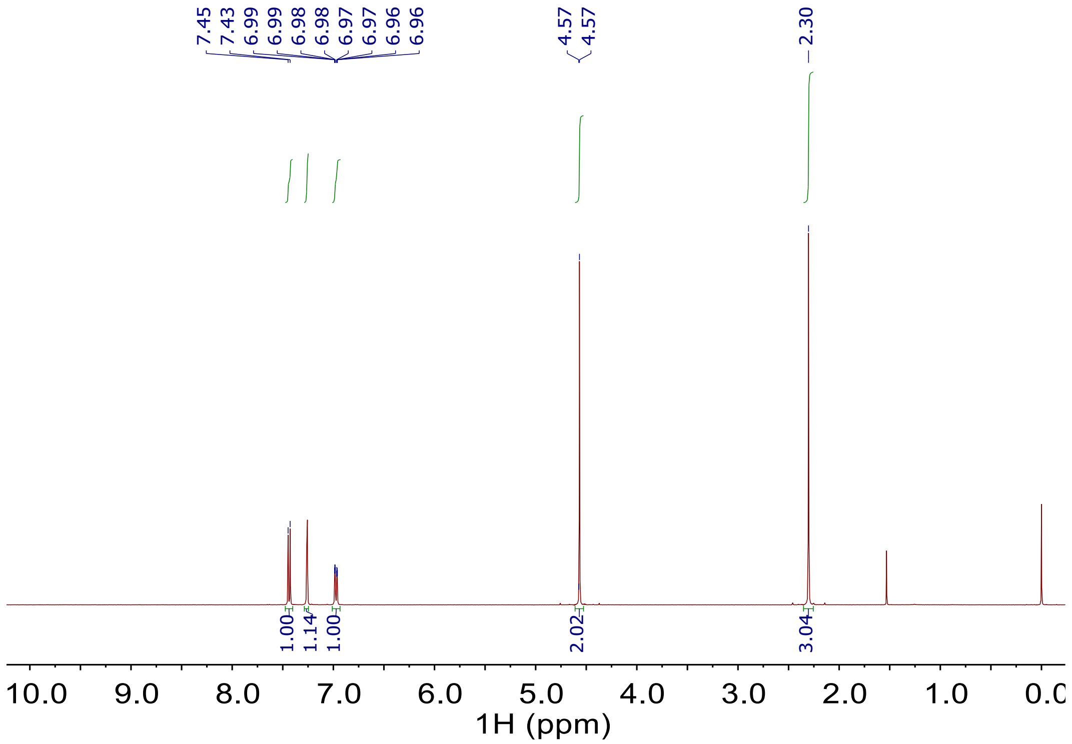

Figure S96. ^{1}H NMR spectrum (400 MHz, $CDCl_3$) of compound **10**.

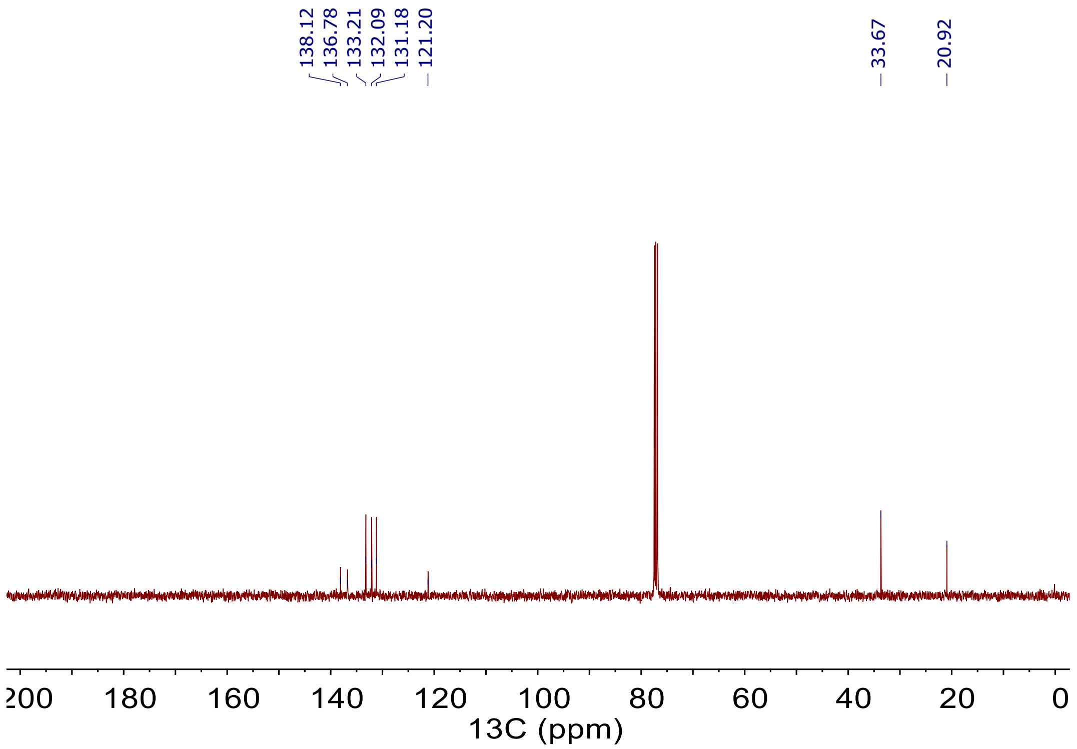

Figure S97. ^{13}C-^{1}H NMR spectrum (101 MHz, $CDCl_3$) of compound **10**.

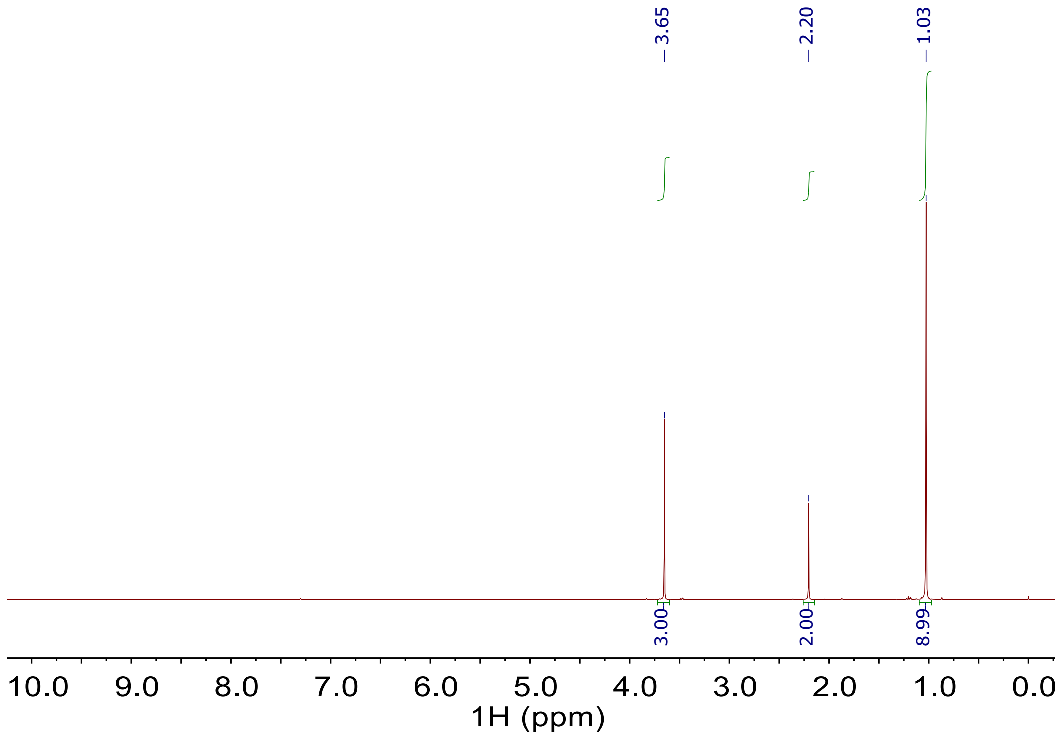

Figure S98. ^{1}H NMR spectrum (400 MHz, $CDCl_3$) of compound **12**.

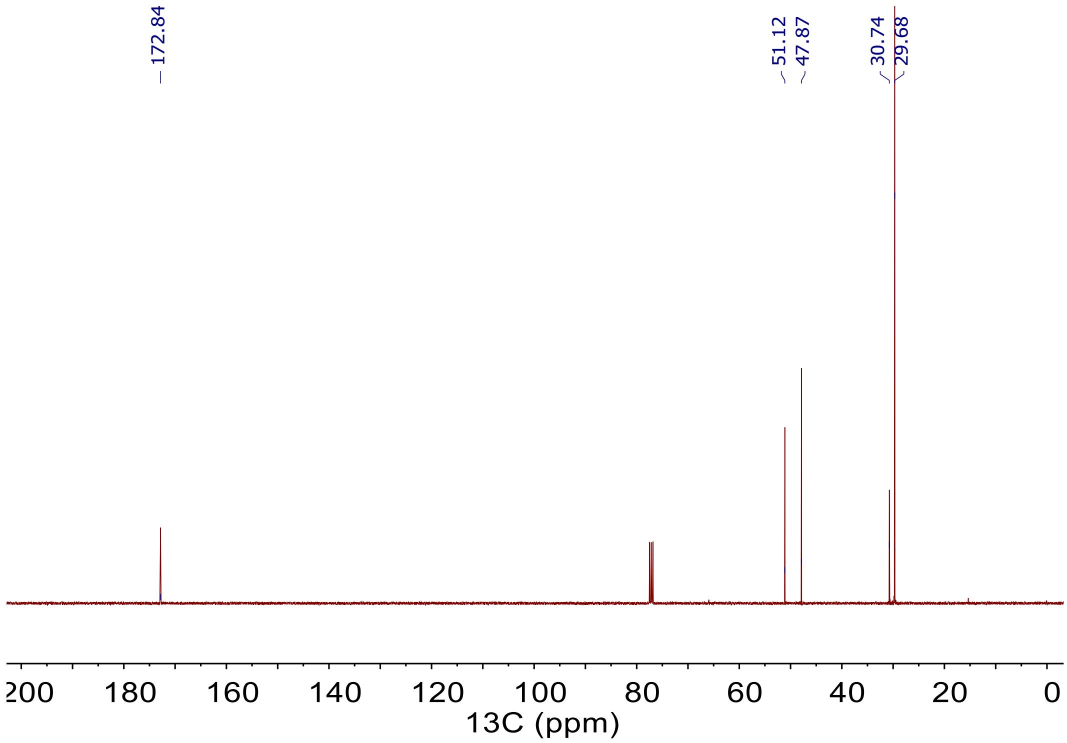

Figure S99. ^{13}C-^{1}H NMR spectrum (101 MHz, $CDCl_3$) of compound **12**.

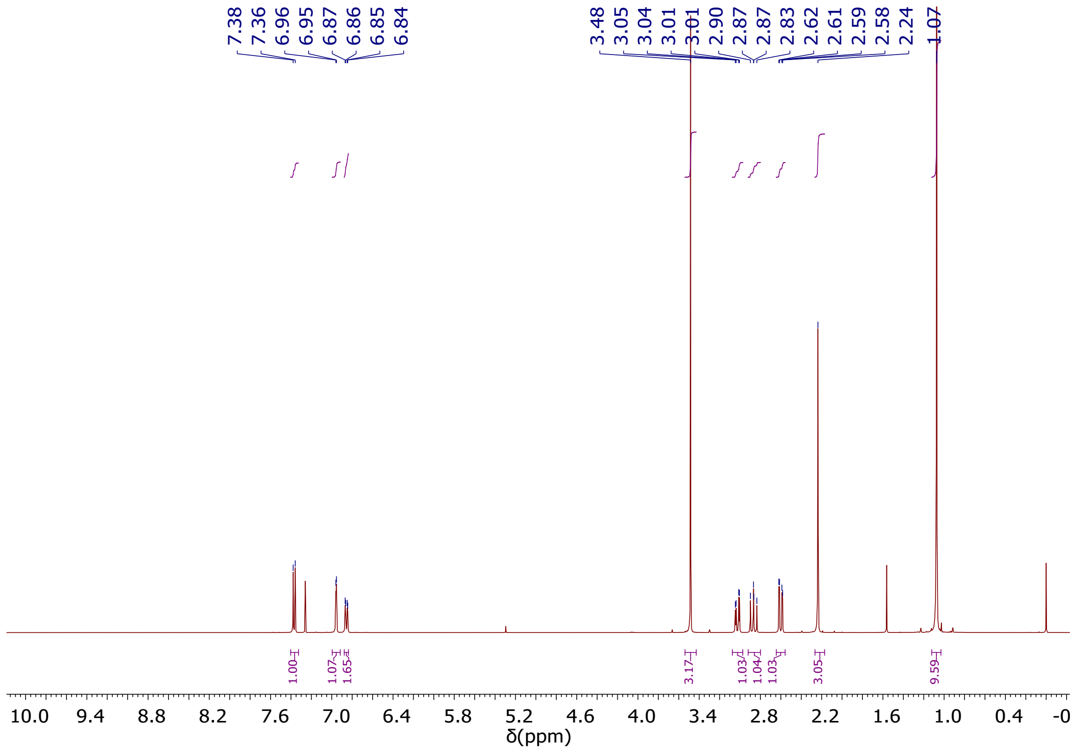

Figure S100. ^{1}H NMR spectrum (400 MHz, $CDCl_3$) of compound **13**.

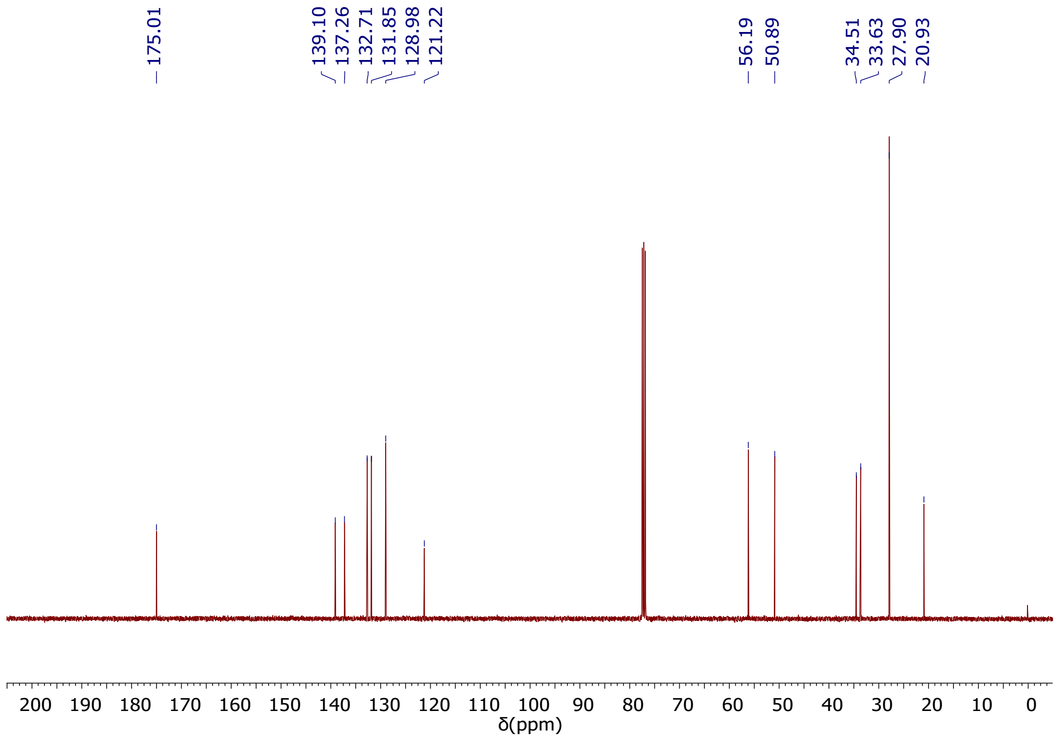

Figure S101. ^{13}C-^{1}H NMR spectrum (101 MHz, $CDCl_3$) of compound **13**.

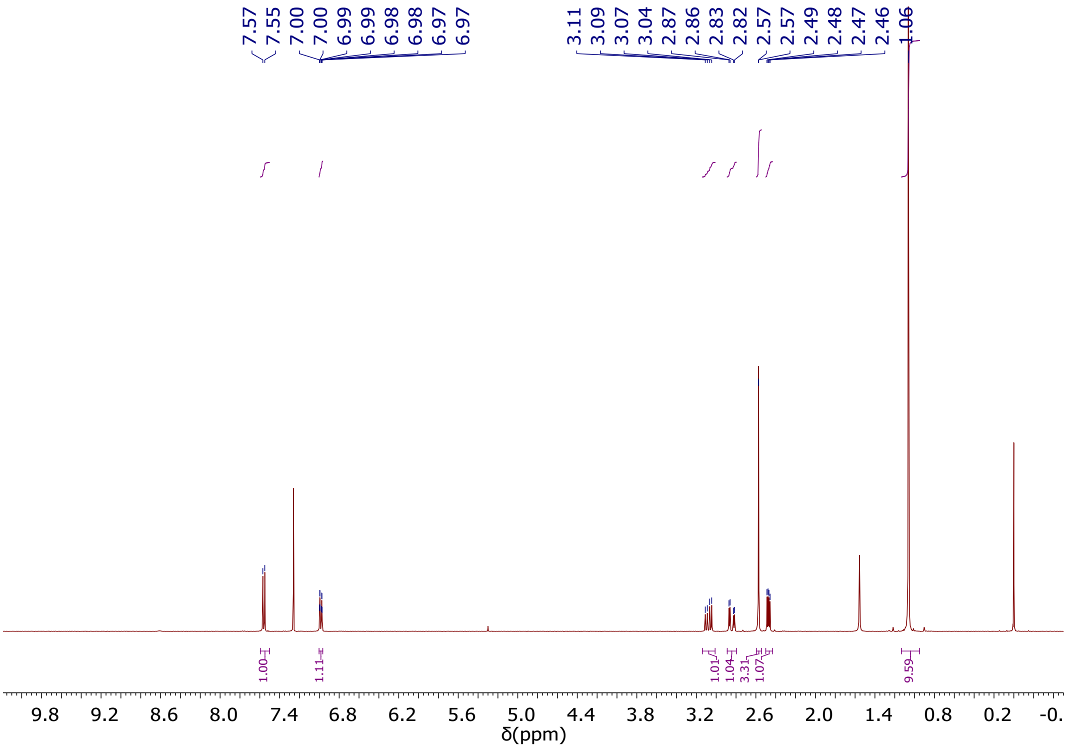

Figure S102. ^{1}H NMR spectrum (400 MHz, $CDCl_3$) of compound **14**.

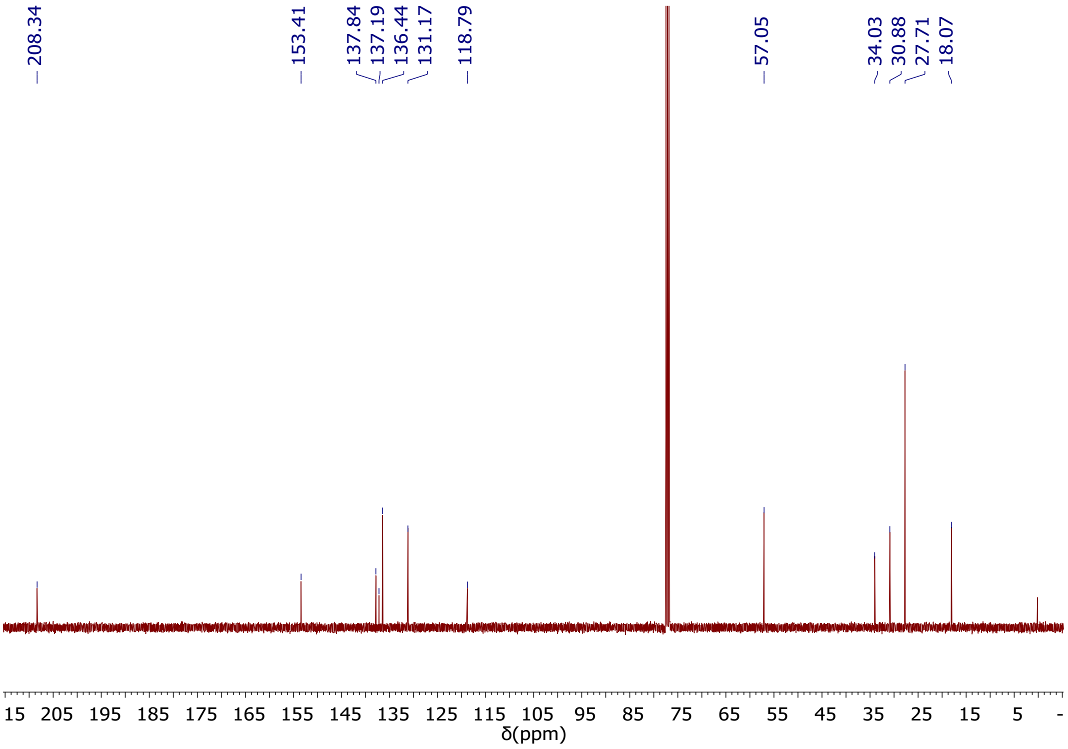

Figure S103. ^{13}C-^{1}H NMR spectrum (101 MHz, $CDCl_3$) of compound **14**.

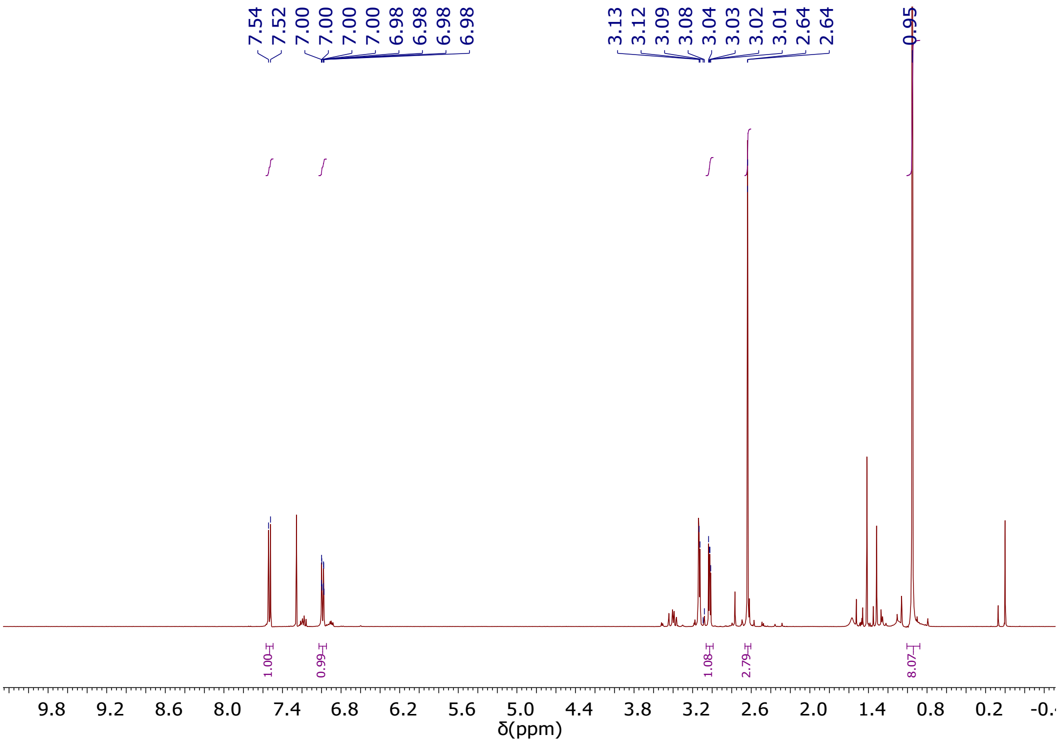

Figure S104. ^{1}H NMR spectrum (400 MHz, $CDCl_3$) of compound **15**.

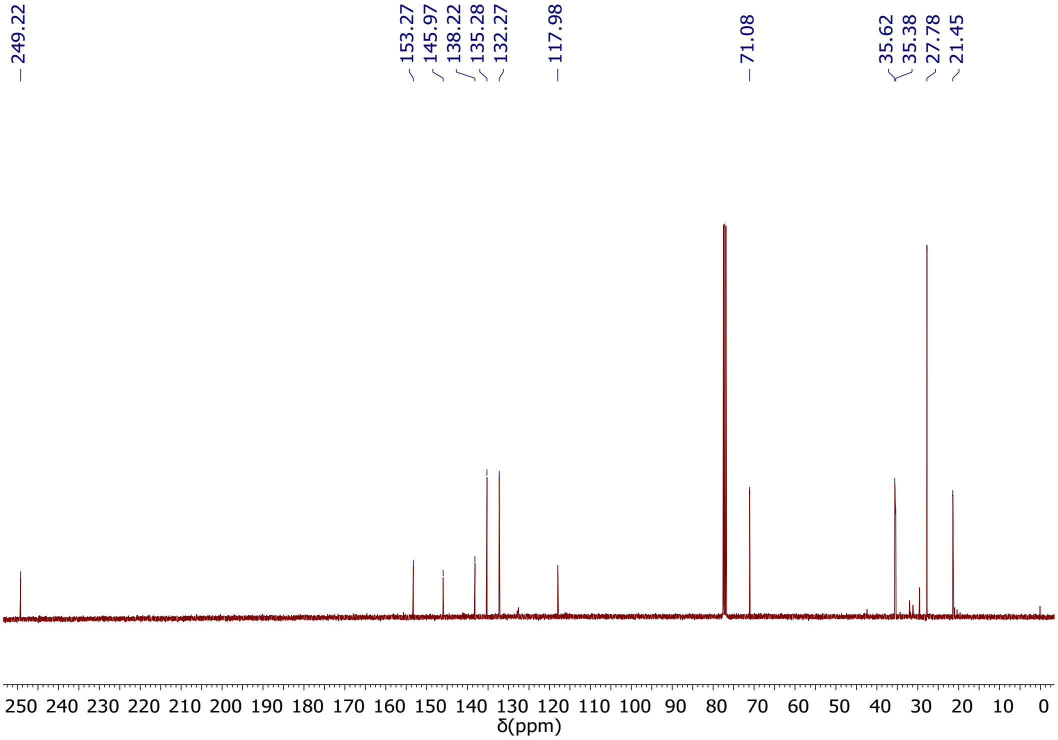

Figure S105. ^{13}C-^{1}H NMR spectrum (101 MHz, $CDCl_3$) of compound **15**.

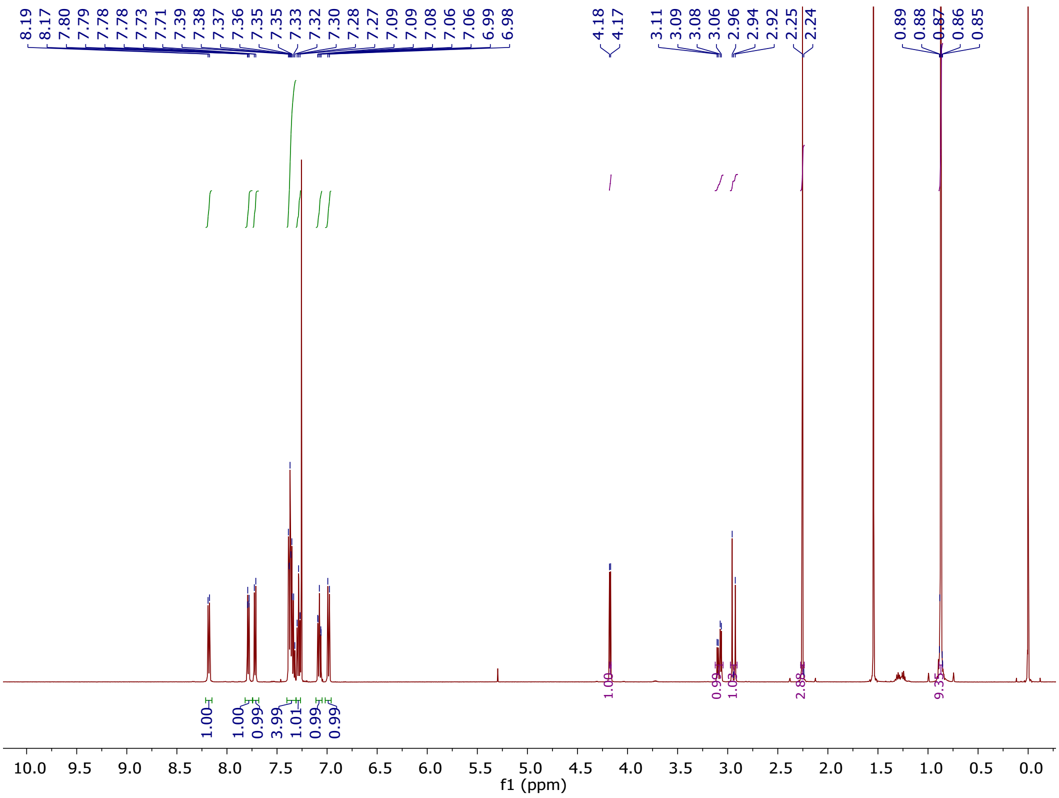

Figure S106. ^{1}H NMR spectrum (500 MHz, $CDCl_3$) of compound **17**.

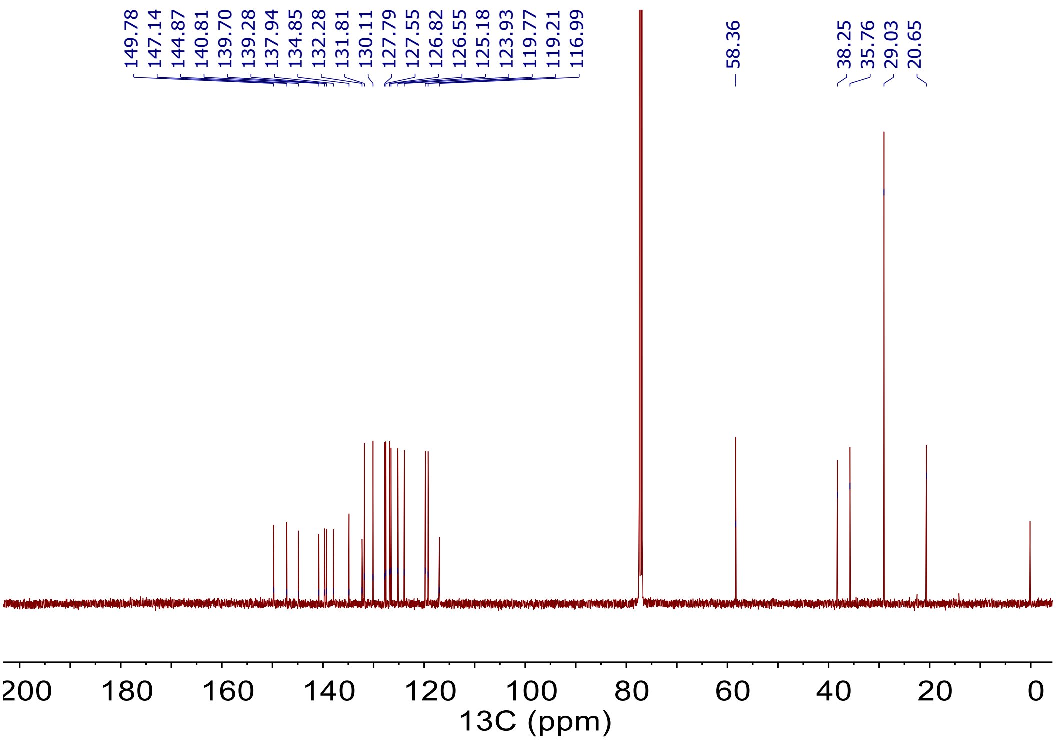

Figure S107. ^{13}C-^{1}H NMR spectrum (126 MHz, $CDCl_3$) of compound **17**.

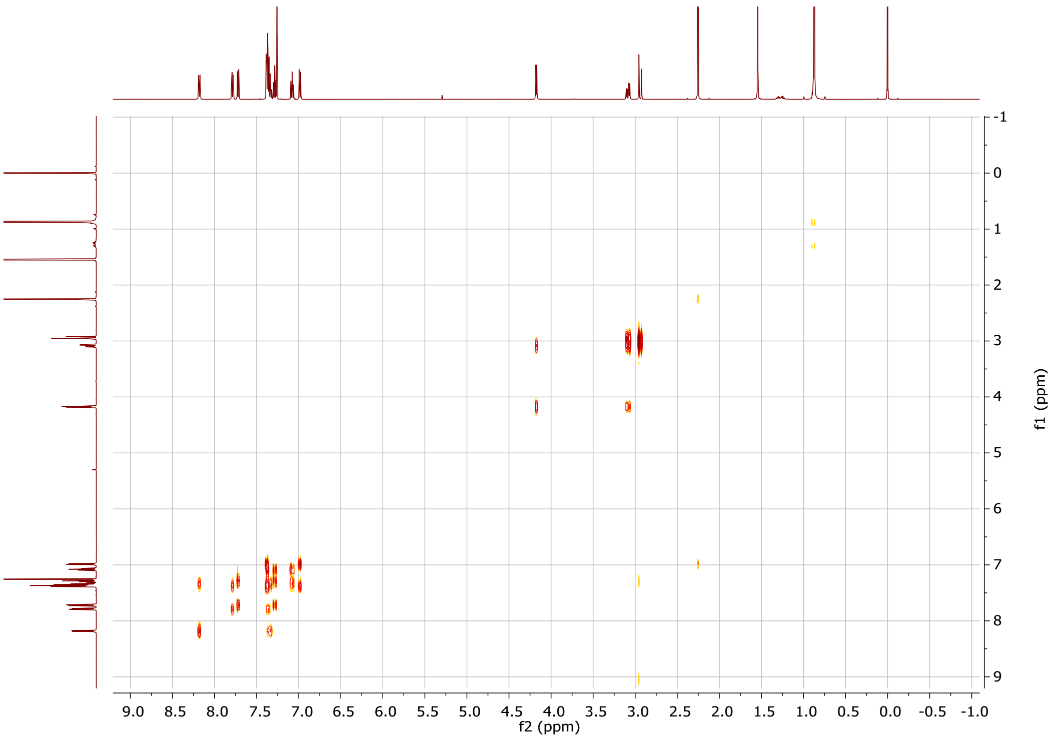

Figure S108. ^{1}H-^{1}H COSY NMR spectrum (500 MHz, $CDCl_3$) of compound **17**.

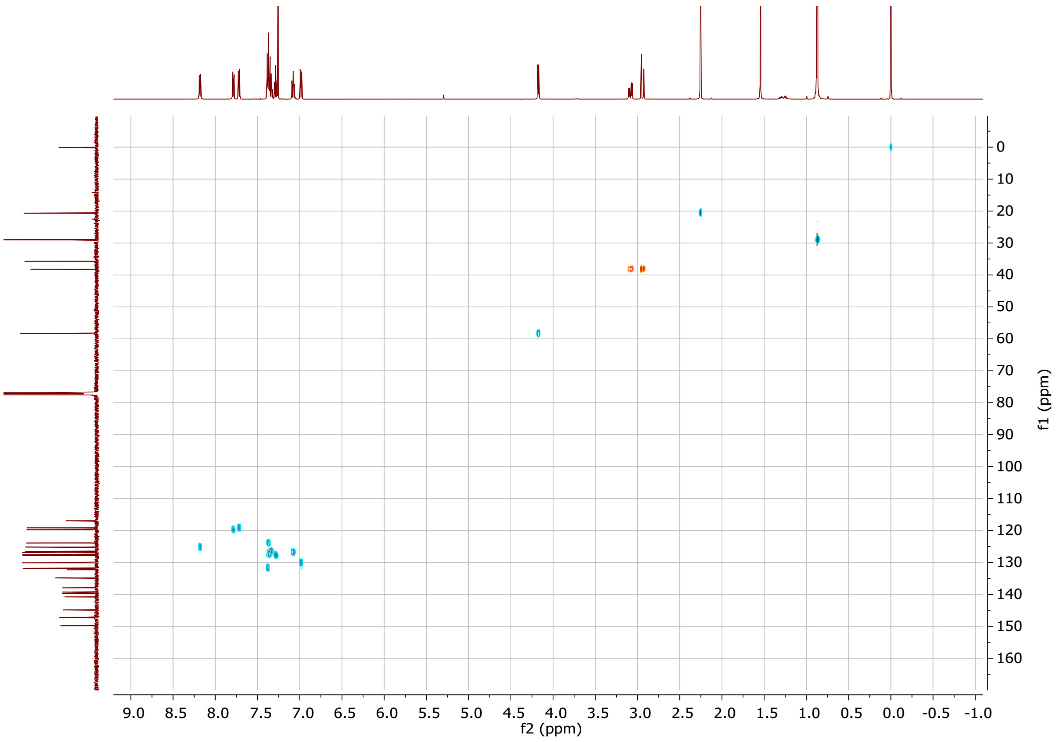

Figure S109. ^{1}H-^{13}C HSQC NMR spectrum (500 MHz, $CDCl_3$) of compound **17**.

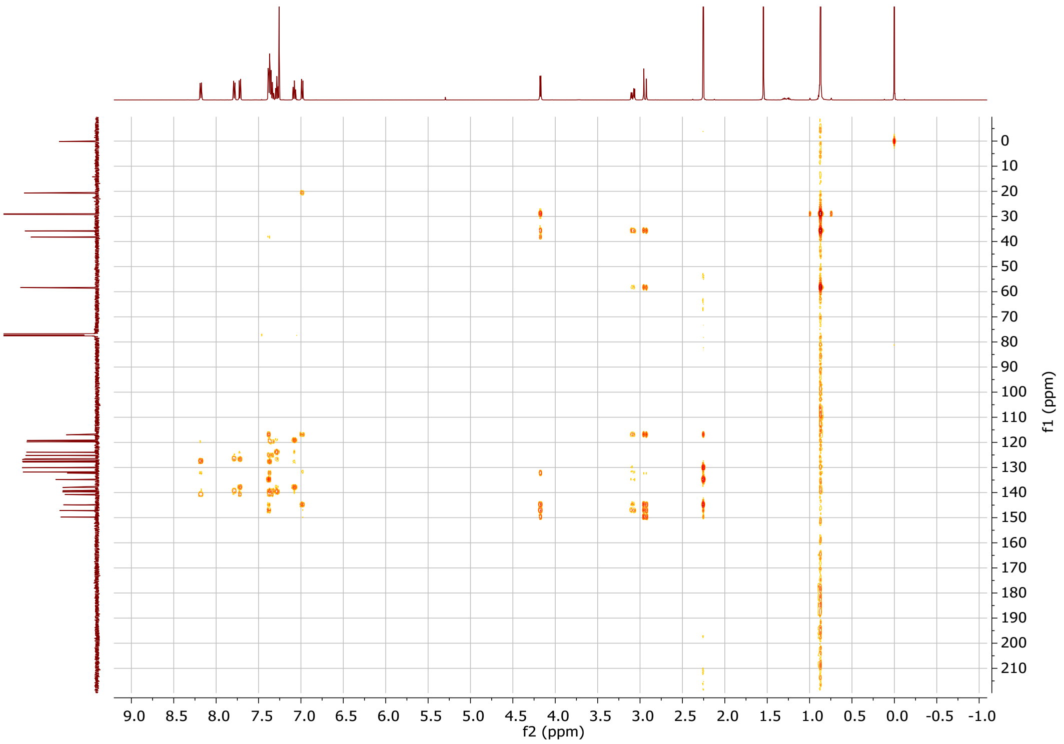

Figure S110. ^{1}H-^{13}C HMBC NMR spectrum (500 MHz, $CDCl_3$) of compound **17**.

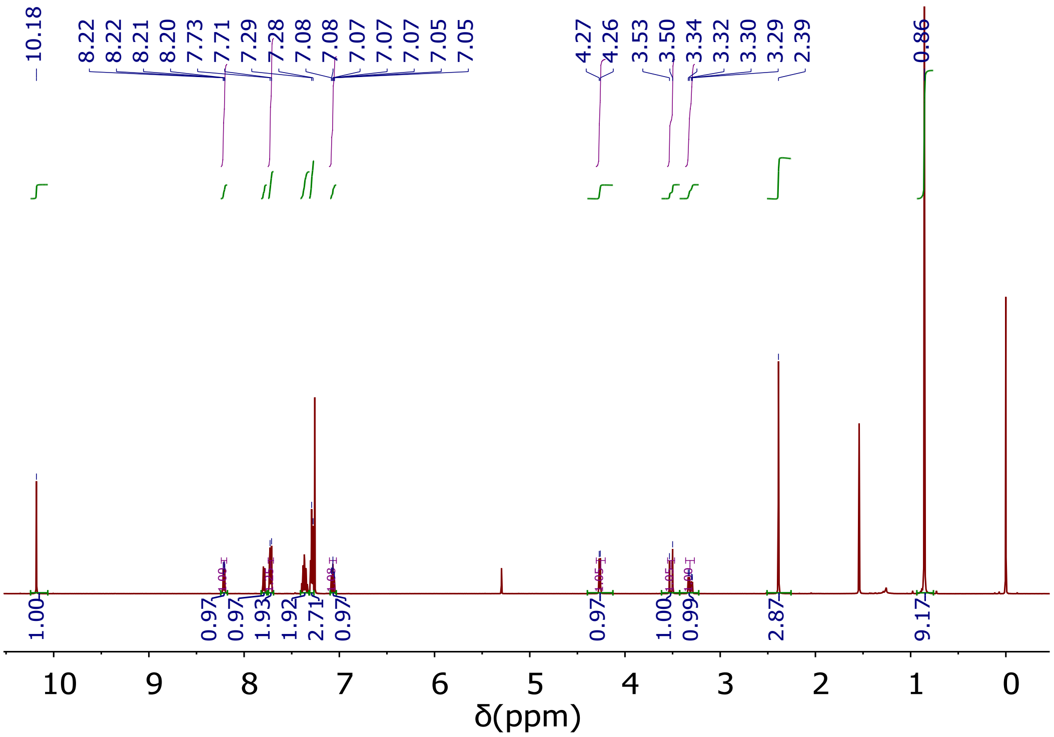

Figure S111. ^{1}H NMR spectrum (500 MHz, $CDCl_3$) of compound **18**.

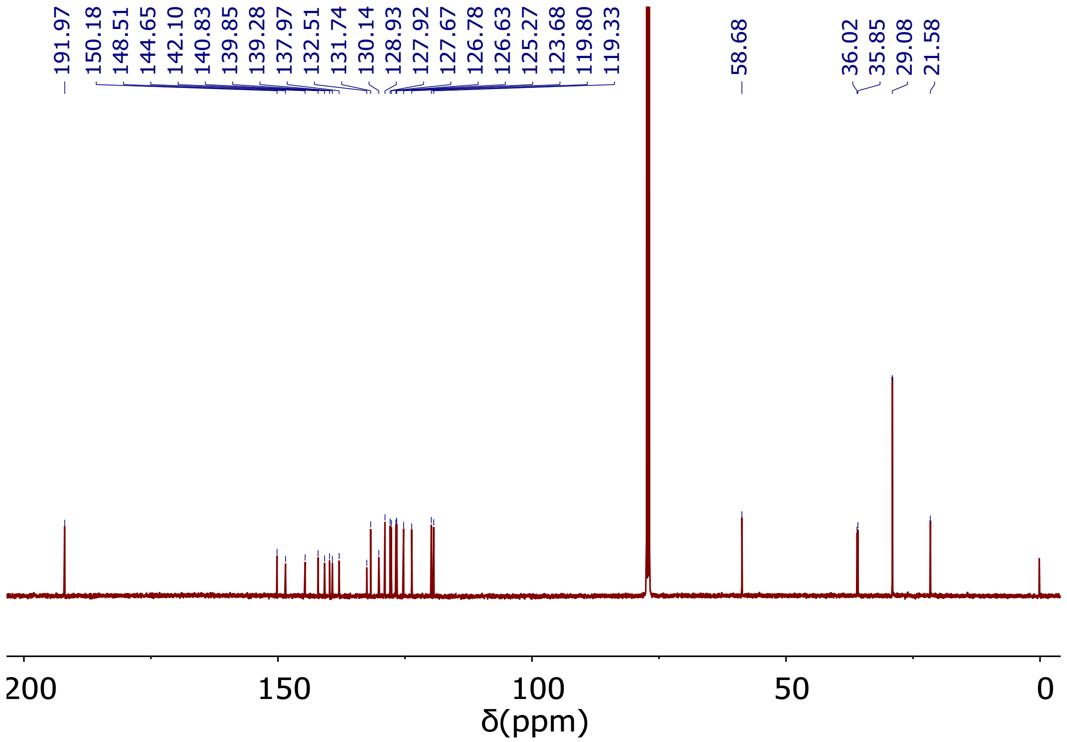

Figure S112. ^{13}C-^{1}H NMR spectrum (126 MHz, $CDCl_3$) of compound **18**.

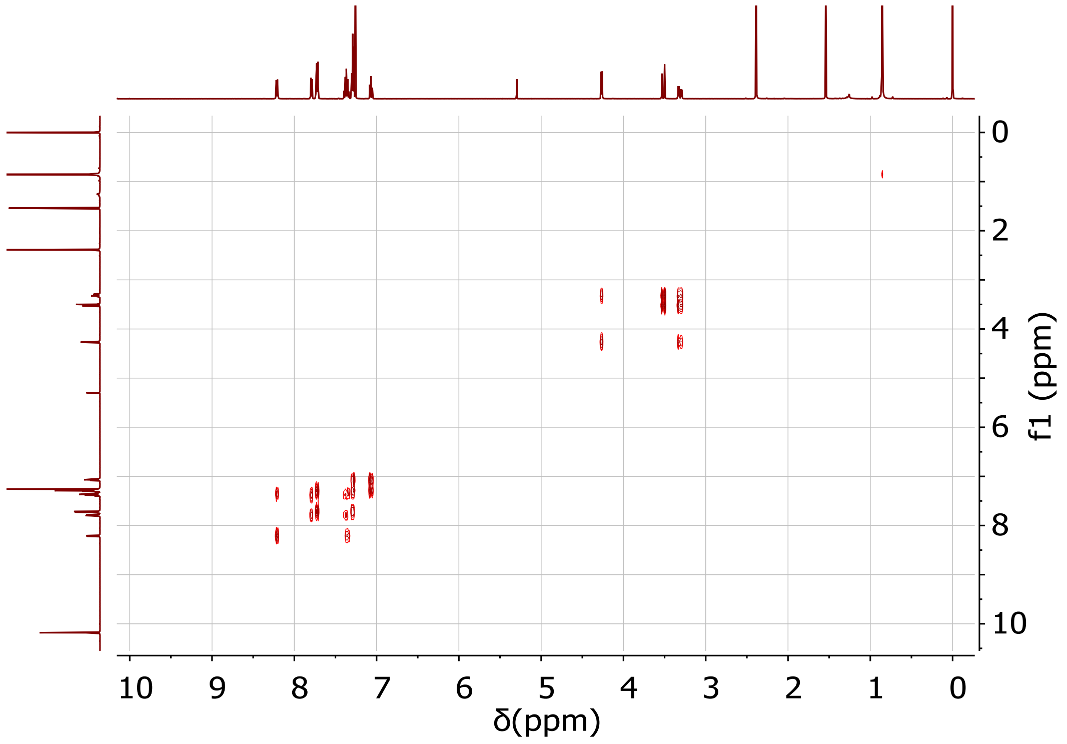

Figure S113. ^{1}H-^{1}H COSY NMR spectrum (500 MHz, $CDCl_3$) of compound **18**.

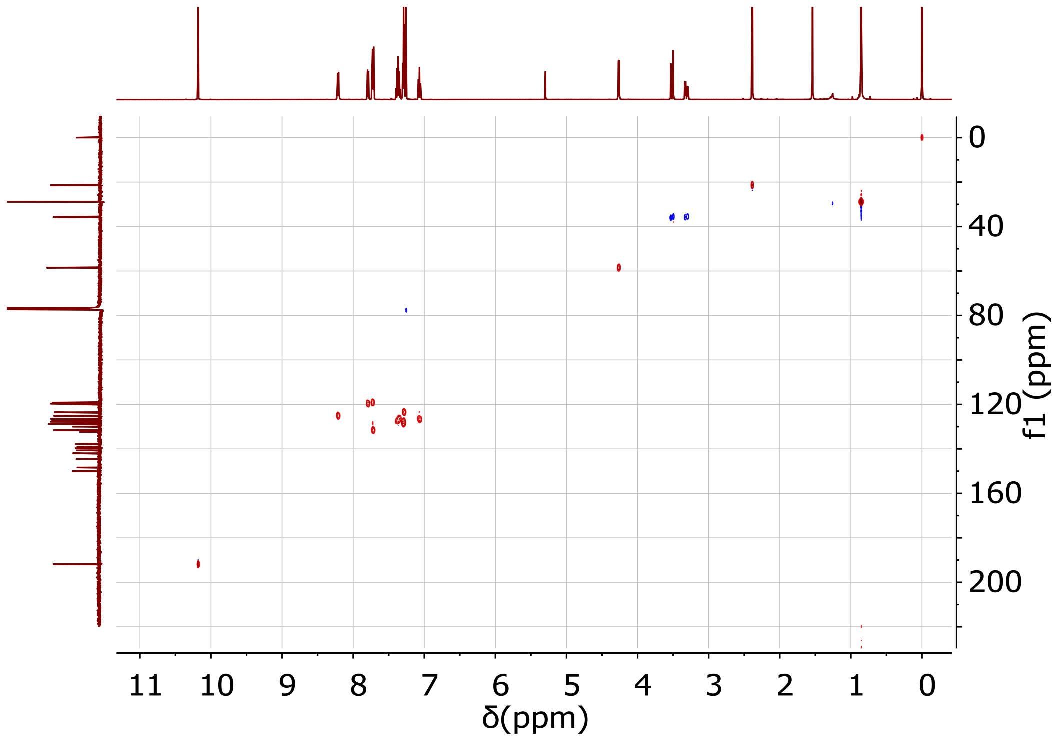

Figure S114. ^{1}H-^{13}C HSQC NMR spectrum (500 MHz, $CDCl_3$) of compound **18**.

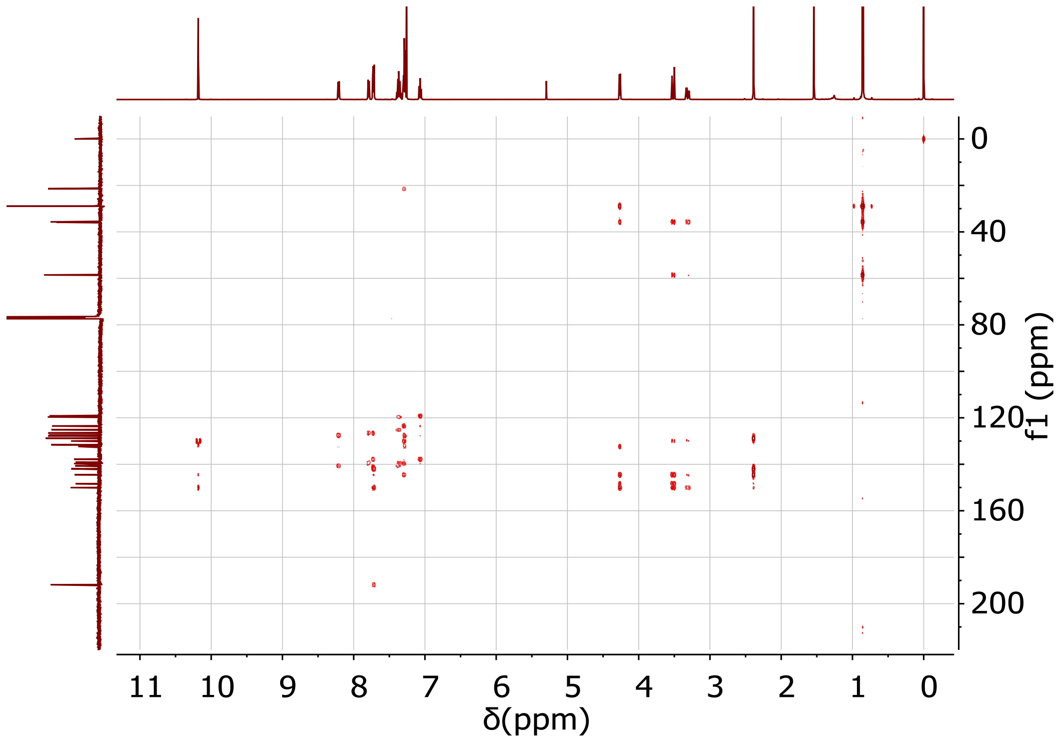

Figure S115. ^{1}H-^{13}C HMBC NMR spectrum (500 MHz, $CDCl_3$) of compound **18**.

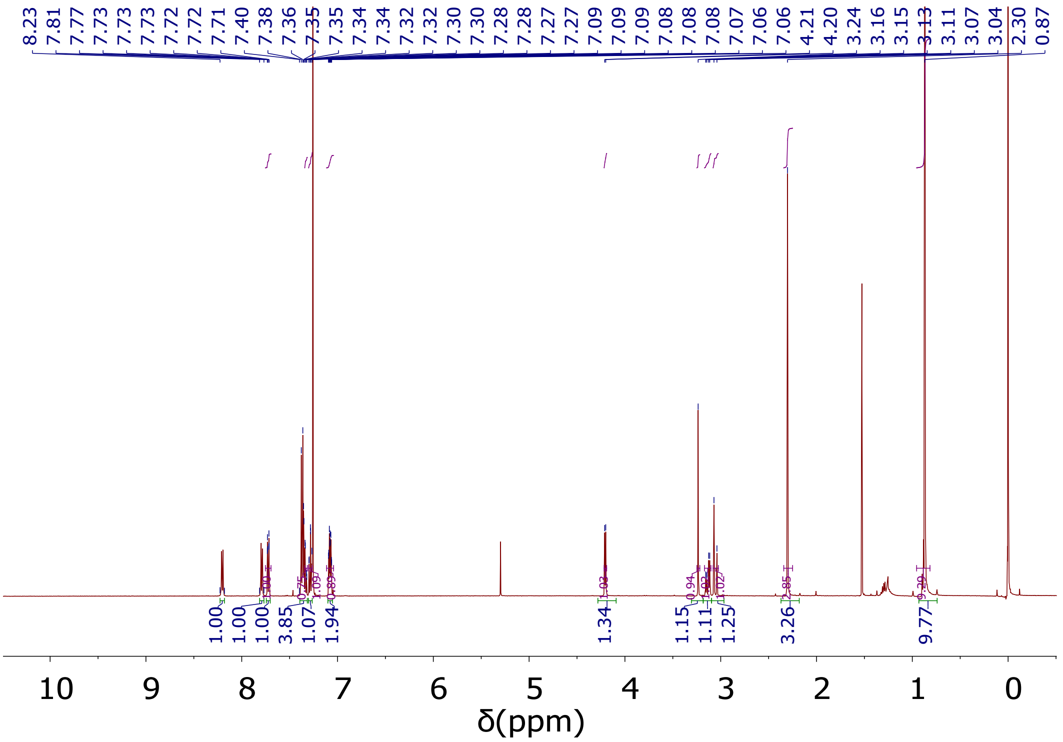

Figure S116. ^{1}H NMR spectrum (500 MHz, $CDCl_3$) of compound **19**.

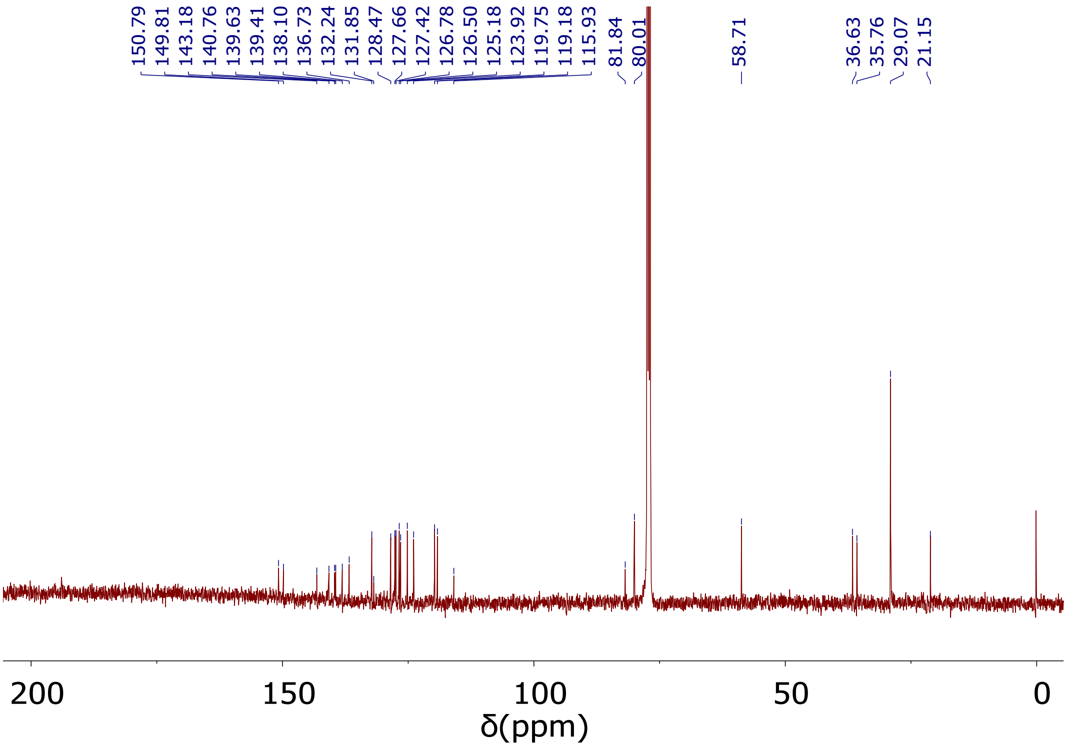

Figure S117. ^{13}C-^{1}H NMR spectrum (126 MHz, $CDCl_3$) of compound **19**.

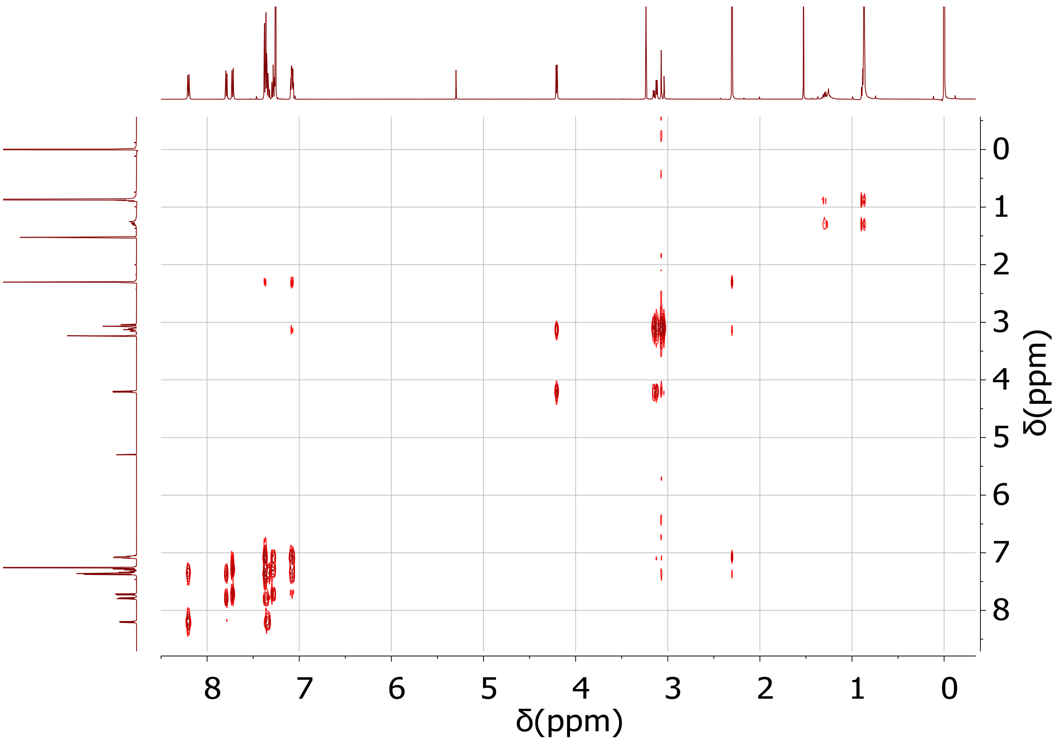

Figure S118. ^1H-^1H COSY NMR spectrum (500 MHz, $CDCl_3$) of compound **19**.

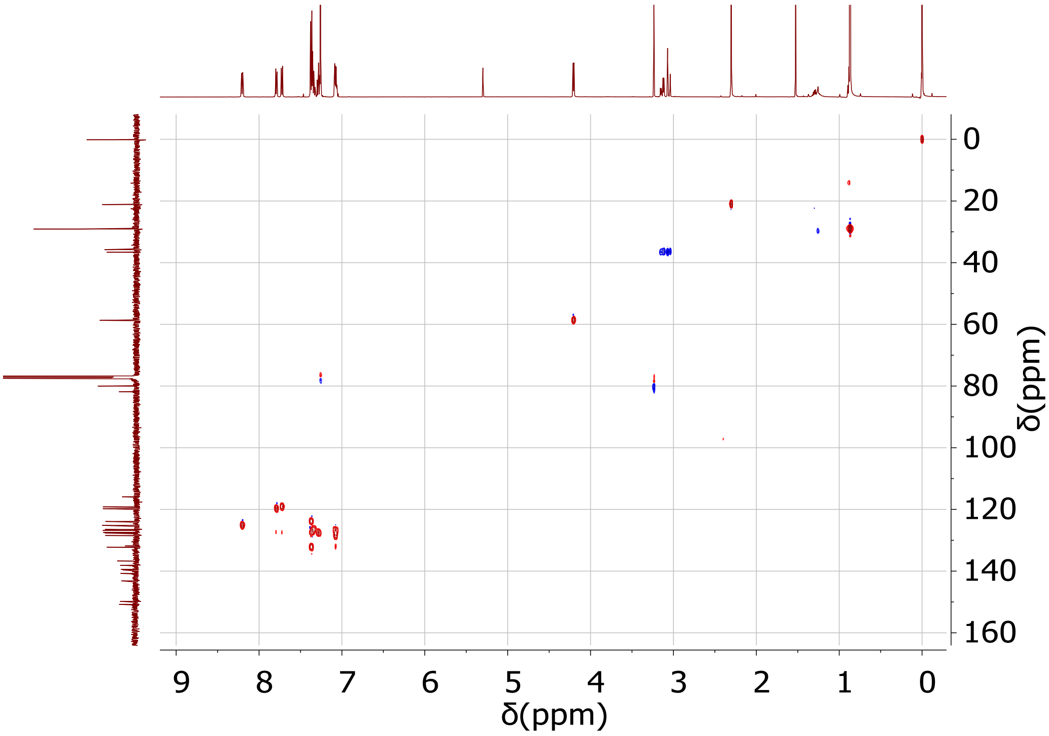

Figure S119. ^1H-^{13}C HSQC NMR spectrum (500 MHz, $CDCl_3$) of compound **19**.

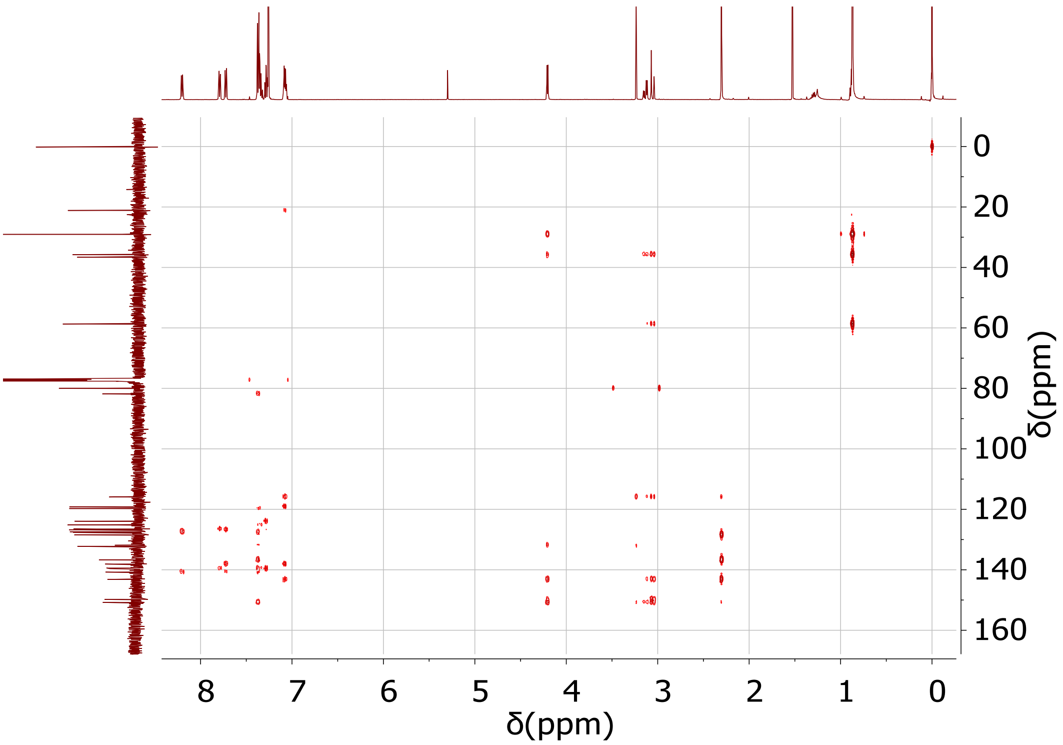

Figure S120. ^{1}H-^{13}C HMBC NMR spectrum (500 MHz, $CDCl_3$) of compound **19**.

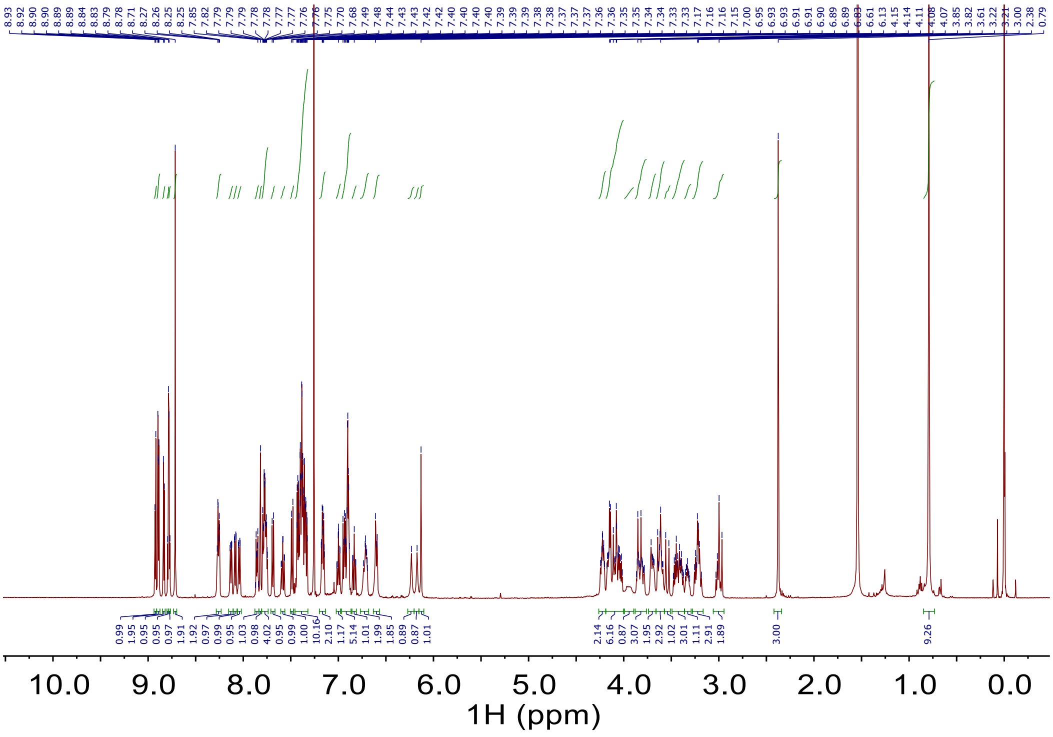

Figure S121. ^{1}H NMR spectrum (500 MHz, $CDCl_3$) of compound (−)-**Zn5a**.

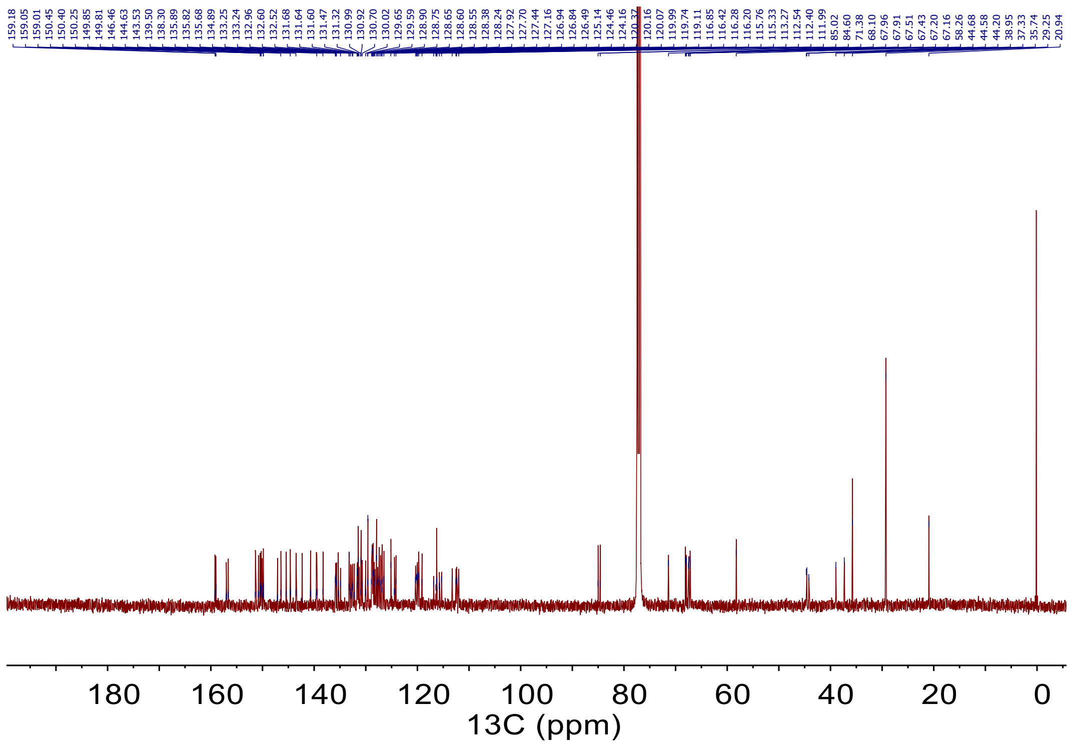

Figure S122. ^{13}C-^{1}H NMR spectrum (126 MHz, $CDCl_3$) of compound (−)-**Zn5a**.

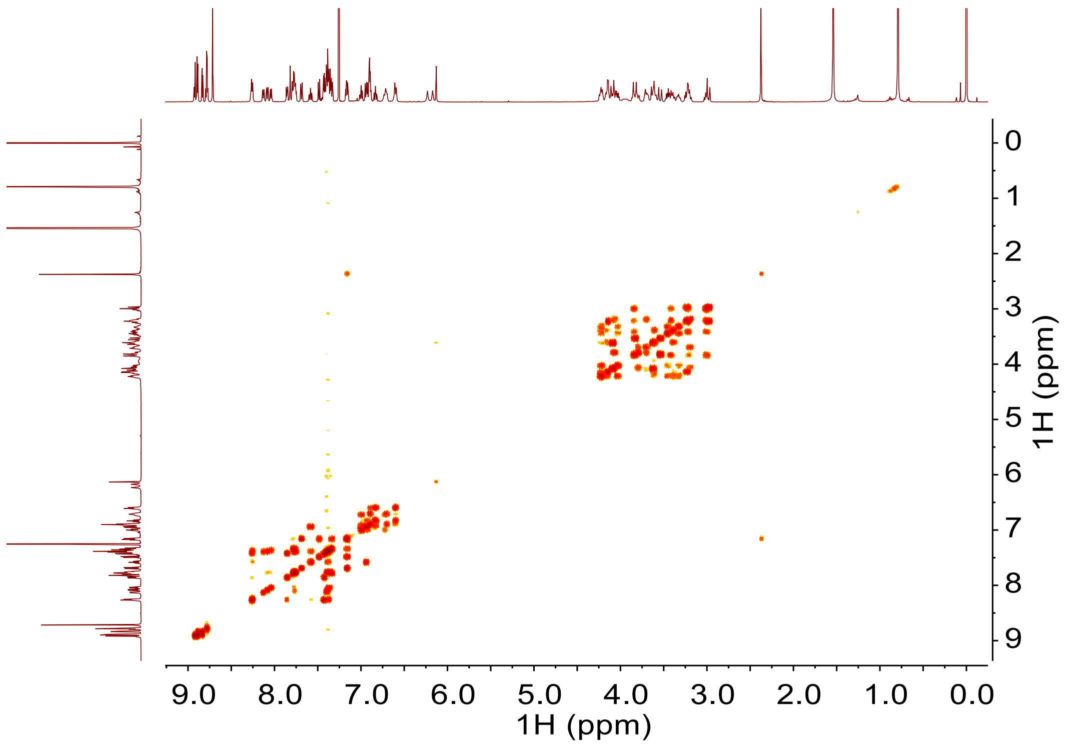

Figure S123. ^{1}H-^{1}H COSY NMR spectrum (500 MHz, $CDCl_3$) of compound (−)-**Zn5a**.

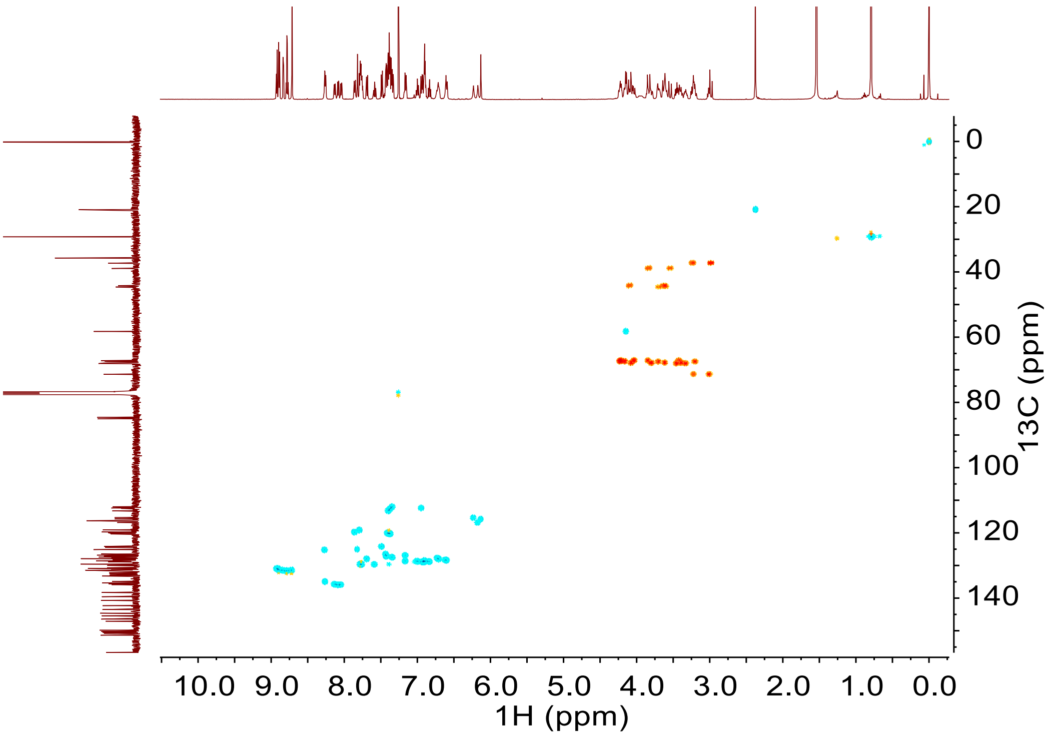

Figure S124. ^{1}H-^{13}C HSQC NMR spectrum (500 MHz, $CDCl_3$) of compound (−)-**Zn5a**.

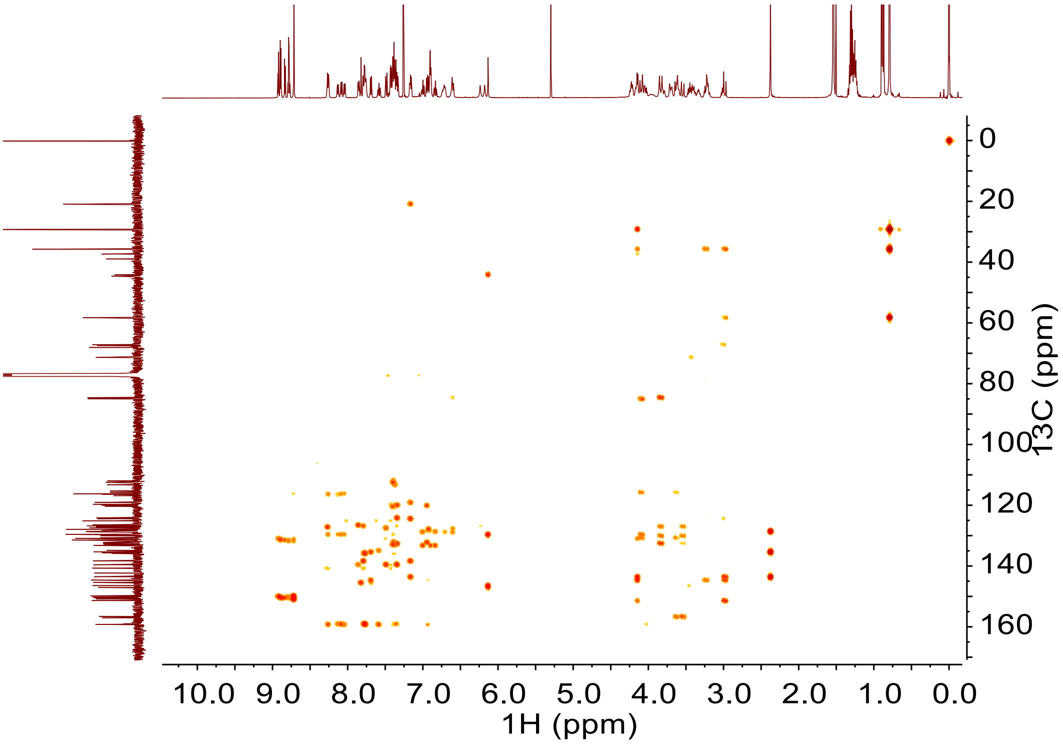

Figure S125. ^{1}H-^{13}C HMBC NMR spectrum (500 MHz, $CDCl_3$) of compound (−)-**Zn5a**.

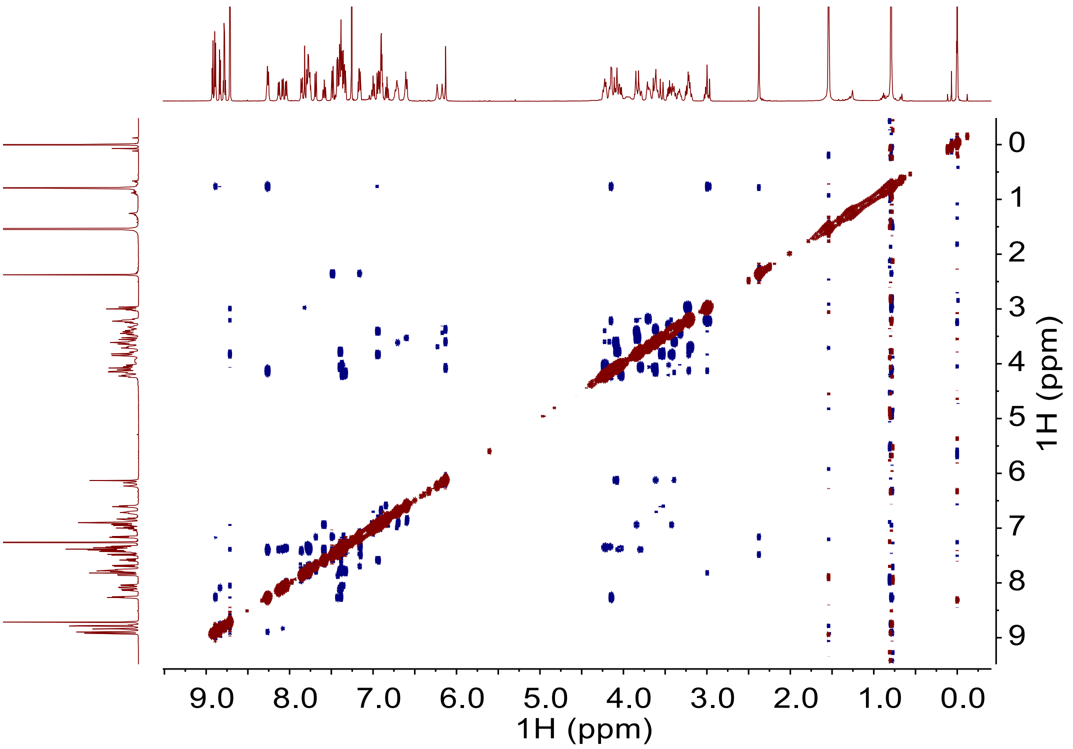

Figure S126. ^{1}H 2D-ROESY NMR spectrum (500 MHz, $CDCl_3$) of compound (−)-**Zn5a**.

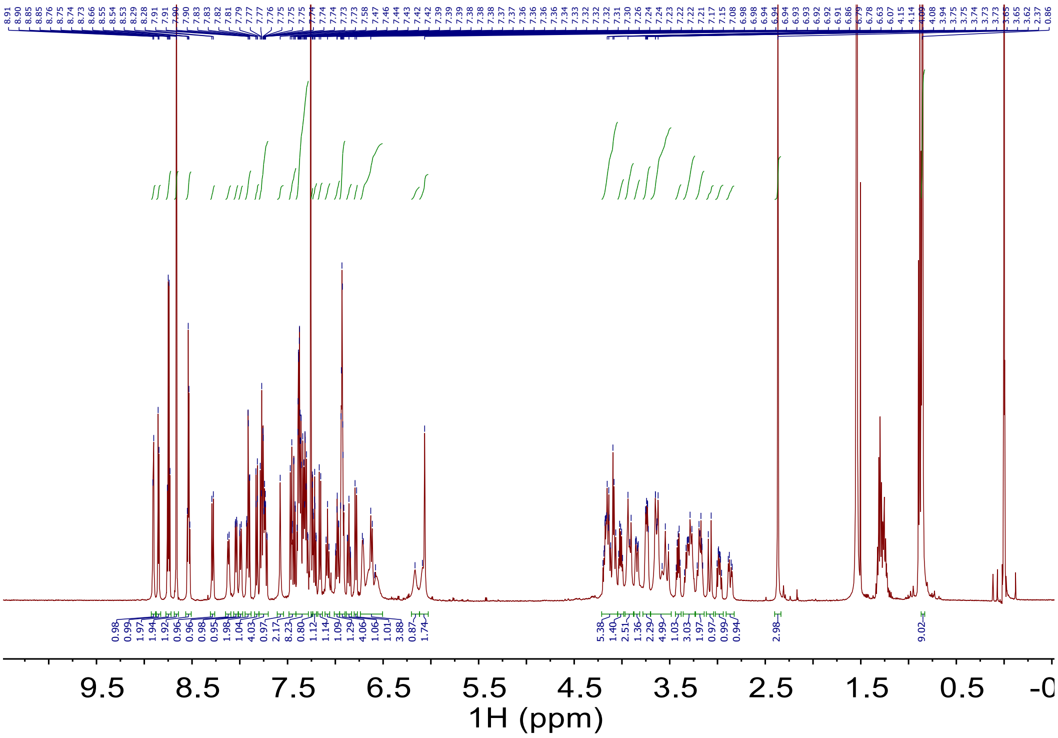

Figure S127. ^{1}H NMR spectrum (500 MHz, $CDCl_3$) of compound (−)-**Zn5b**.

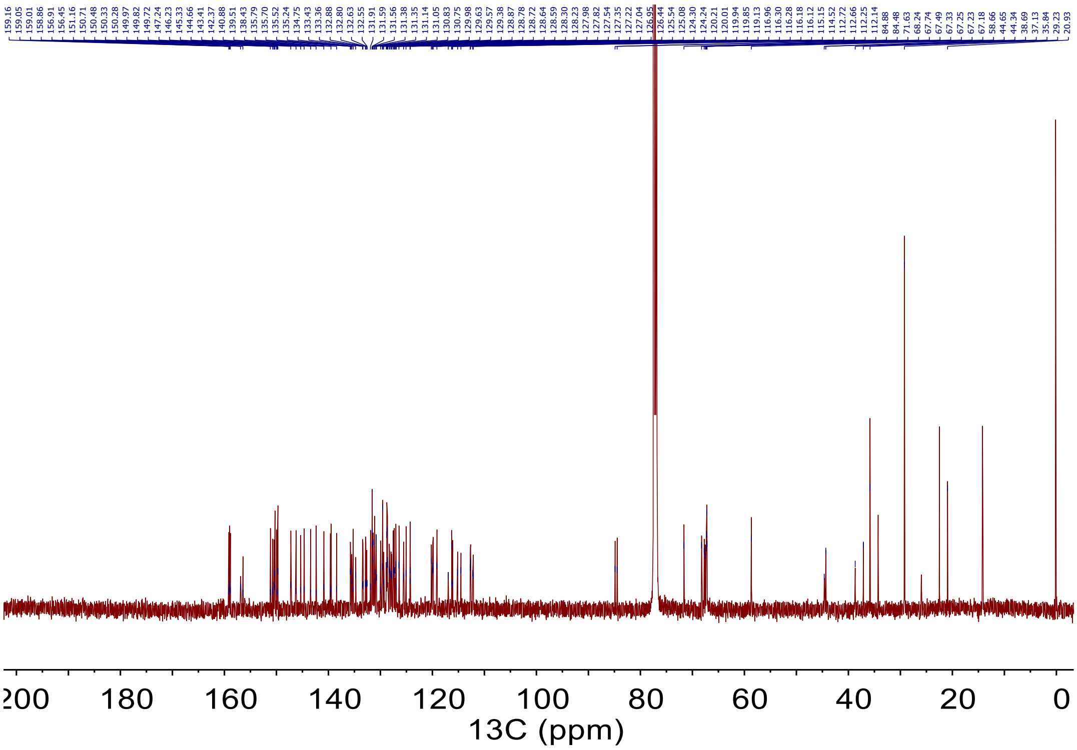

Figure S128. ^{13}C-^{1}H NMR spectrum (126 MHz, $CDCl_3$) of compound (−)-**Zn5b**.

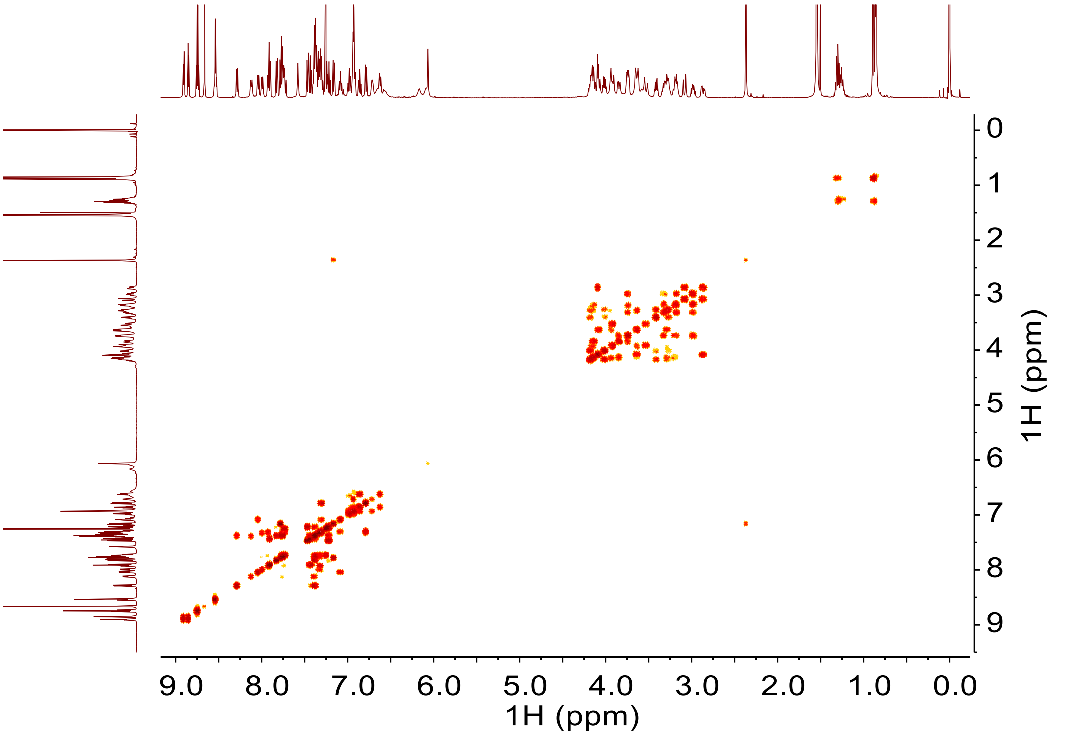

Figure S129. ^{1}H-^{13}C HMBC NMR spectrum (500 MHz, $CDCl_3$) of compound (−)-**Zn5b**.

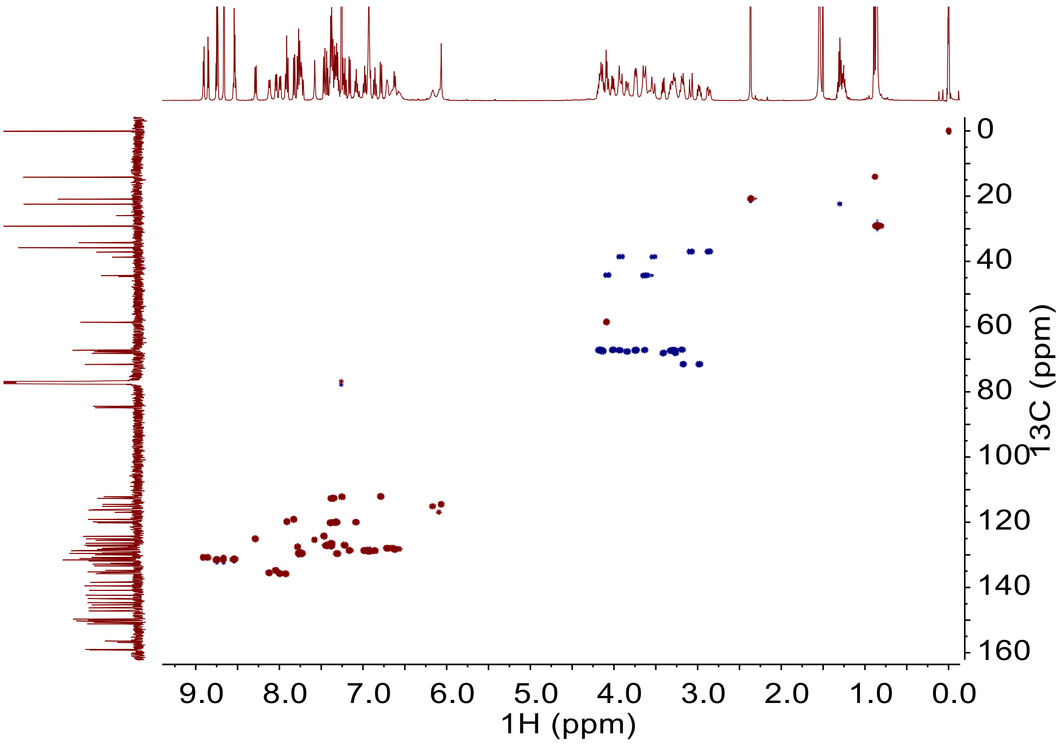

Figure S130. ^{1}H-^{13}C HSQC NMR spectrum (500 MHz, $CDCl_3$) of compound (−)-**Zn5b**.

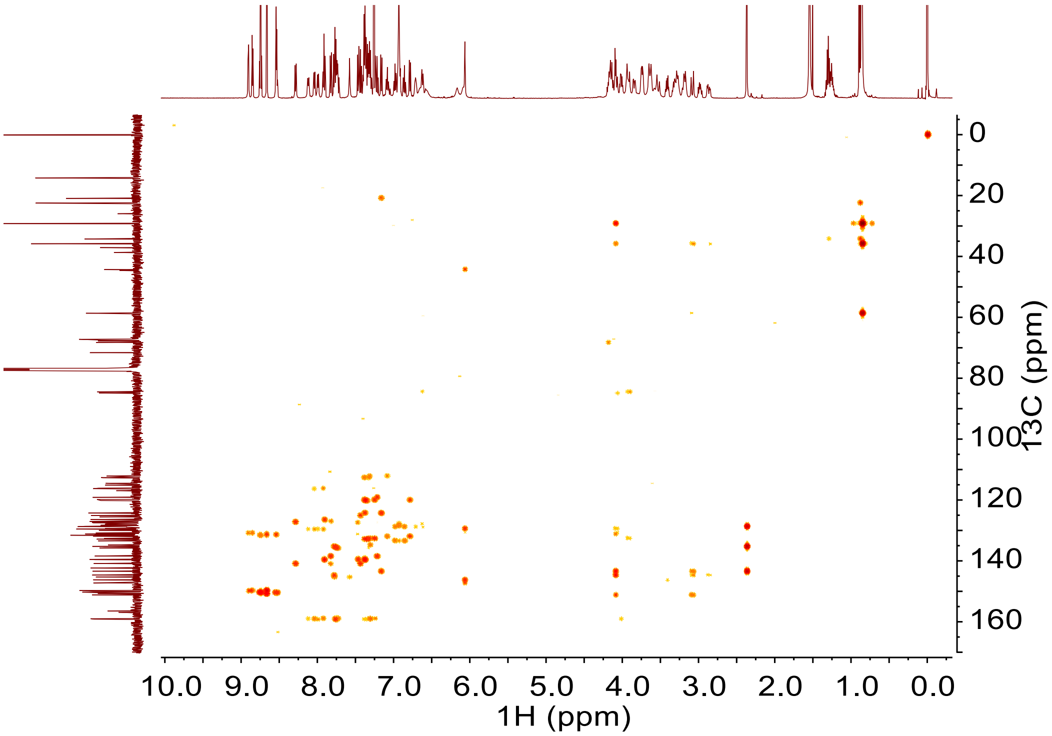

Figure S131. ^{1}H-^{13}C HMBC NMR spectrum (500 MHz, $CDCl_3$) of compound (−)-**Zn5b**.

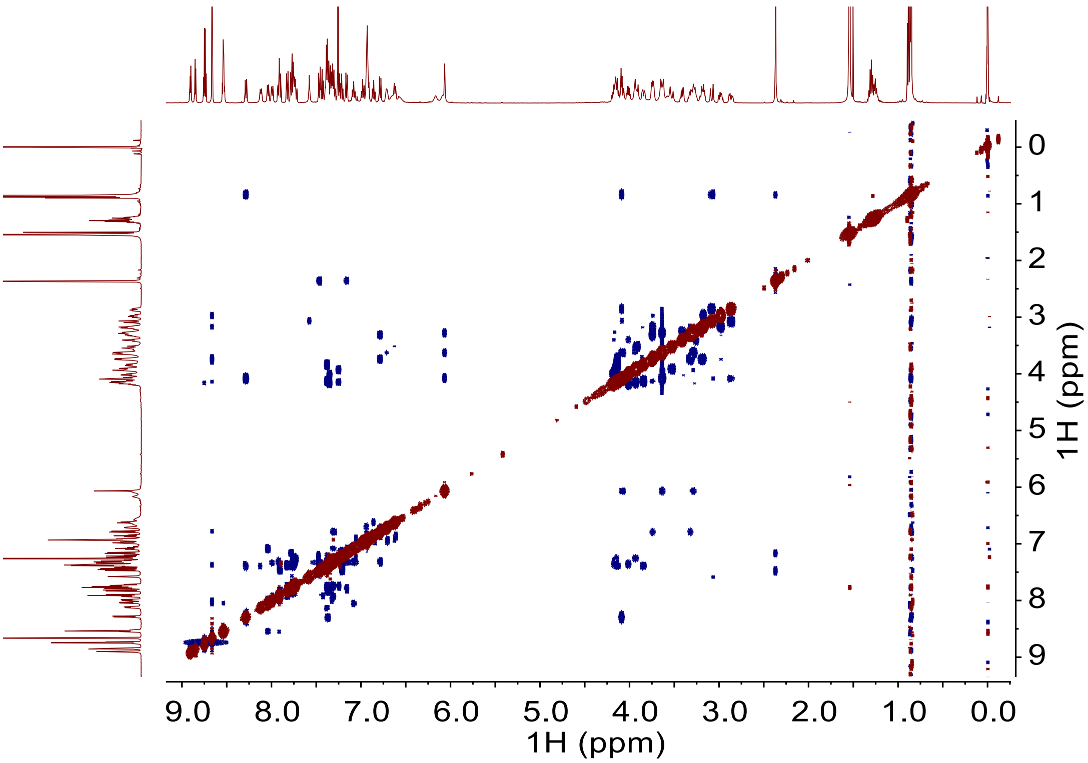

Figure S132. ^{1}H 2D-ROESY NMR spectrum (500 MHz, $CDCl_3$) of compound (−)-**Zn5b**.

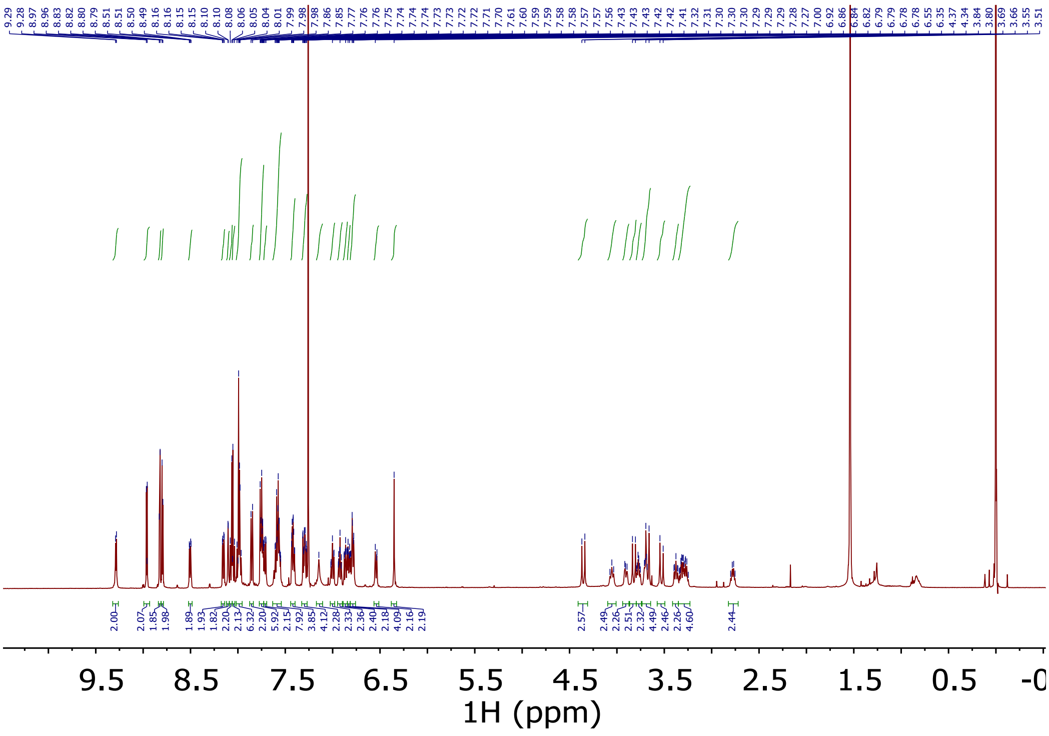

Figure S133. ^{1}H NMR spectrum (500 MHz, $CDCl_3$) of compound *S*$_p$-(*R*,*S*)-(*M*)-**Zn6a**

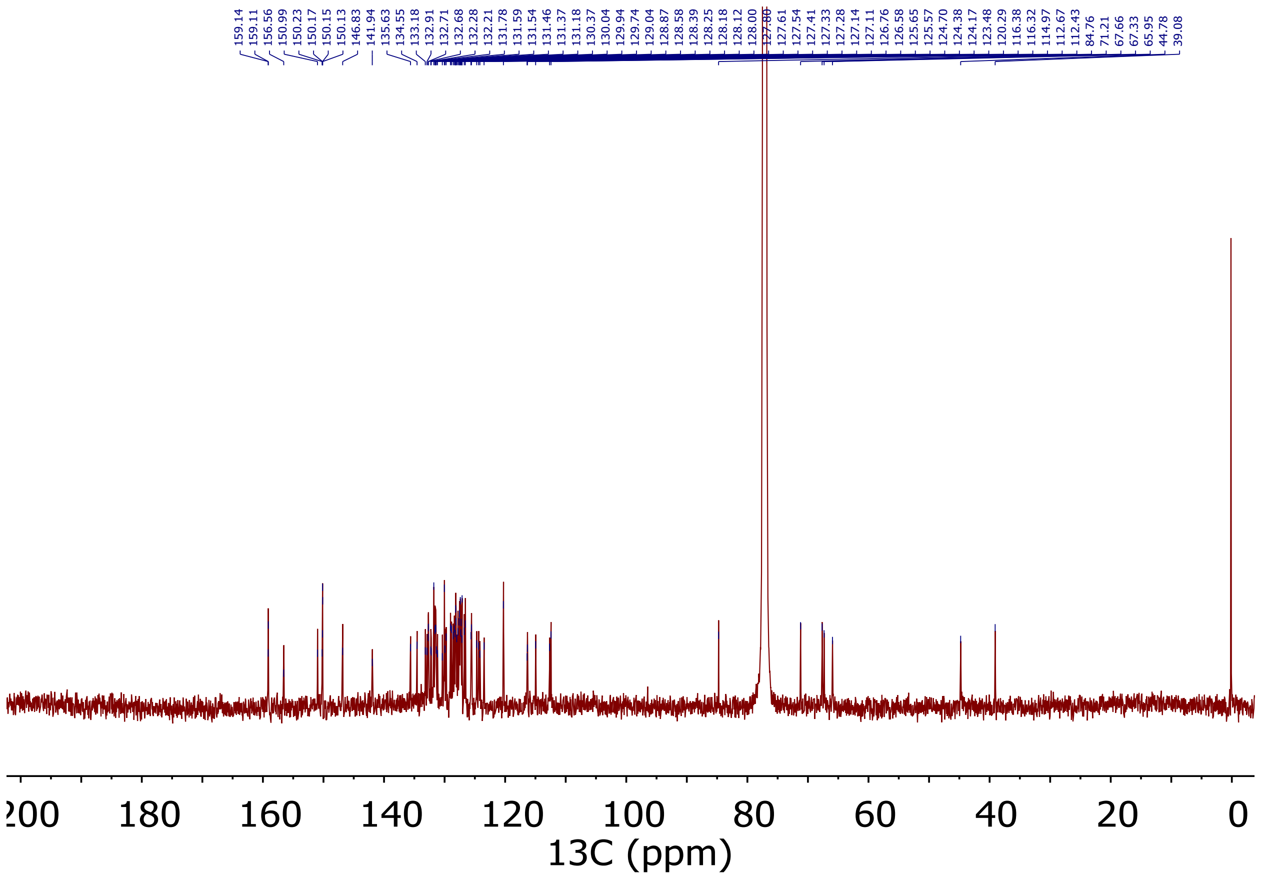

Figure S134. ^{13}C-^{1}H NMR spectrum (126 MHz, $CDCl_3$) of compound S_p-(*R,S*)-(*M*)-**Zn6a**

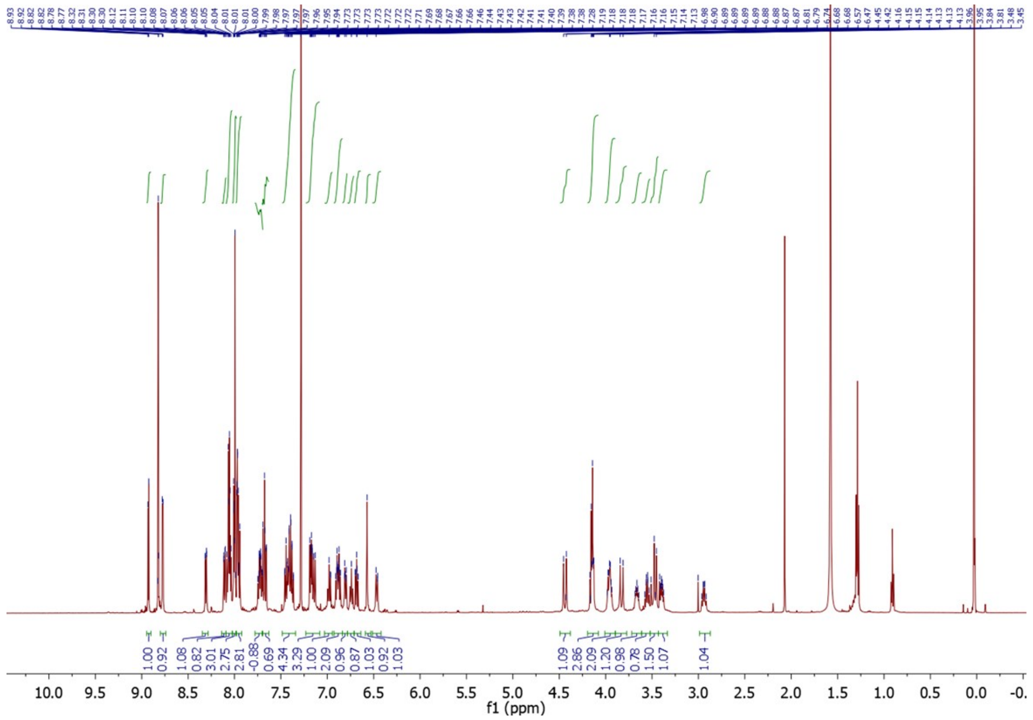

Figure S135. ^{1}H NMR spectrum (500 MHz, $CDCl_3$) of compound S_p-(*R,S*)-(*P*)-**Zn6b**

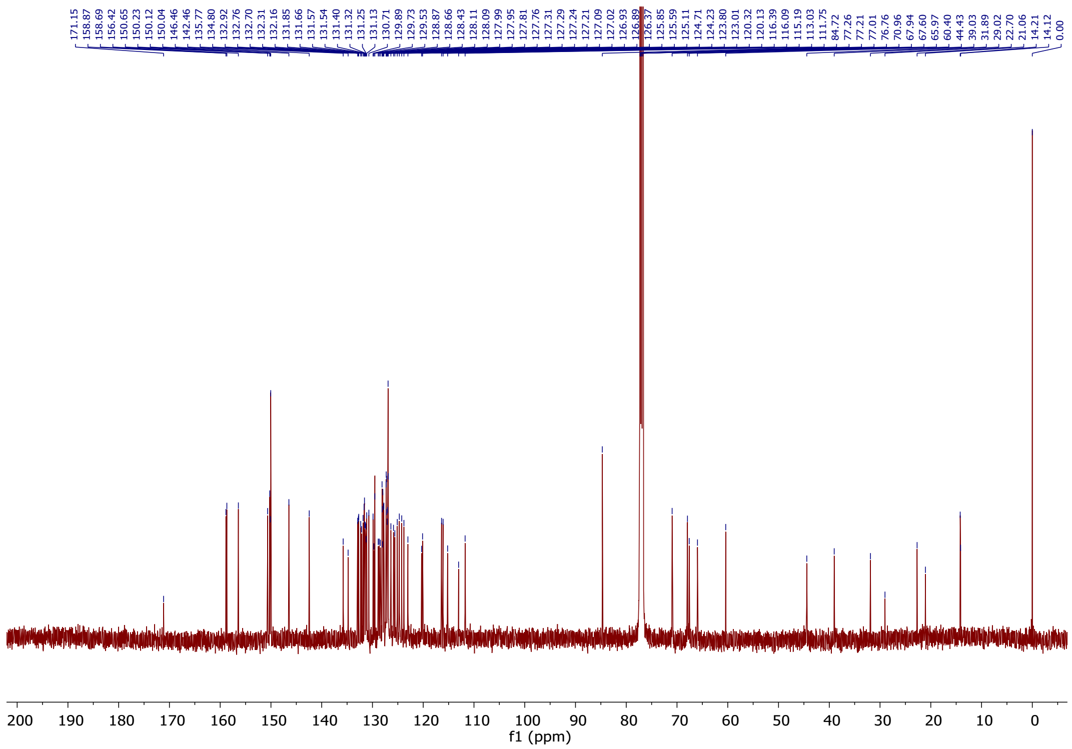

Figure S136. ^{13}C-^{1}H NMR spectrum (126 MHz, $CDCl_3$) of compound S_p-(*R,S*)-(*P*)-**Zn6b**

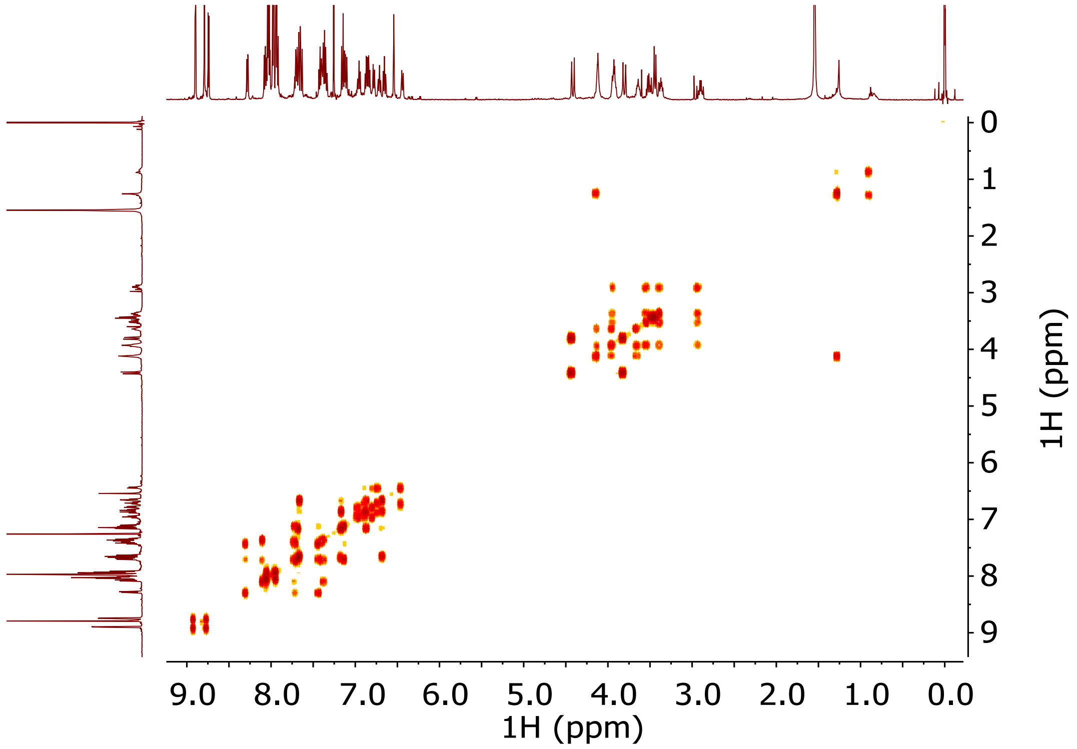

Figure S137. ^{1}H-^{1}H COSY NMR spectrum (500 MHz, $CDCl_3$) of compound S_p-(*R,S*)-(*P*)-**Zn6b**

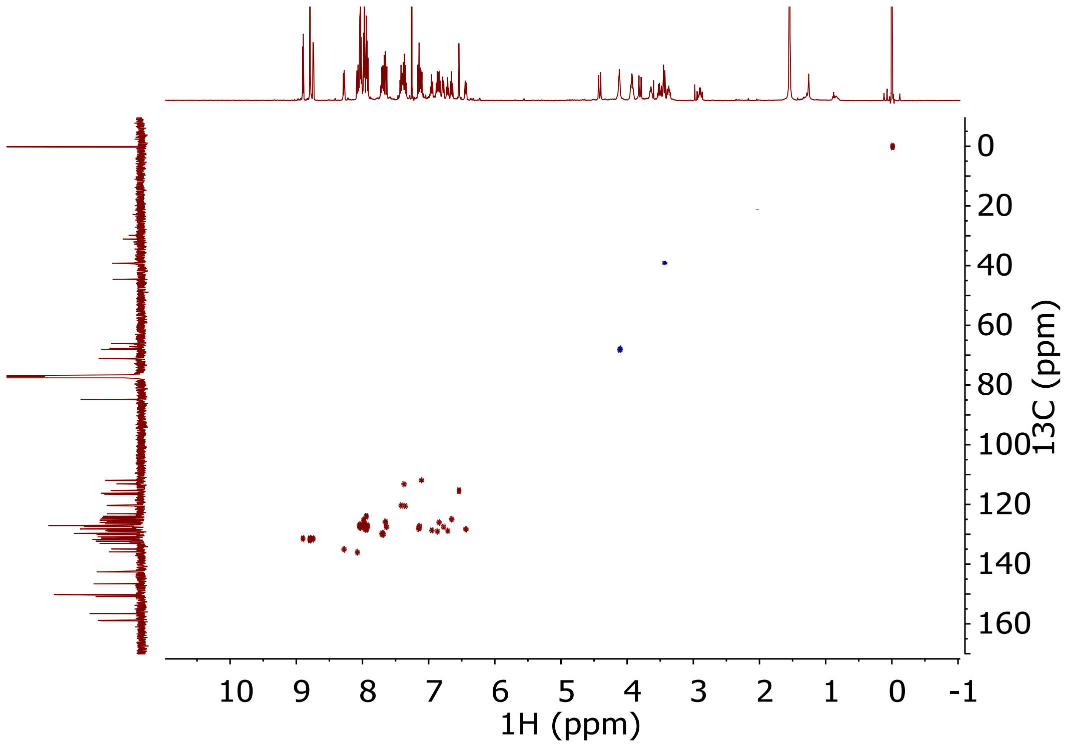

Figure S138. ^{1}H-^{13}C HSQC NMR spectrum (500 MHz, $CDCl_3$) of compound *S*$_p$-(*R,S*)-(*P*)-**Zn6b**

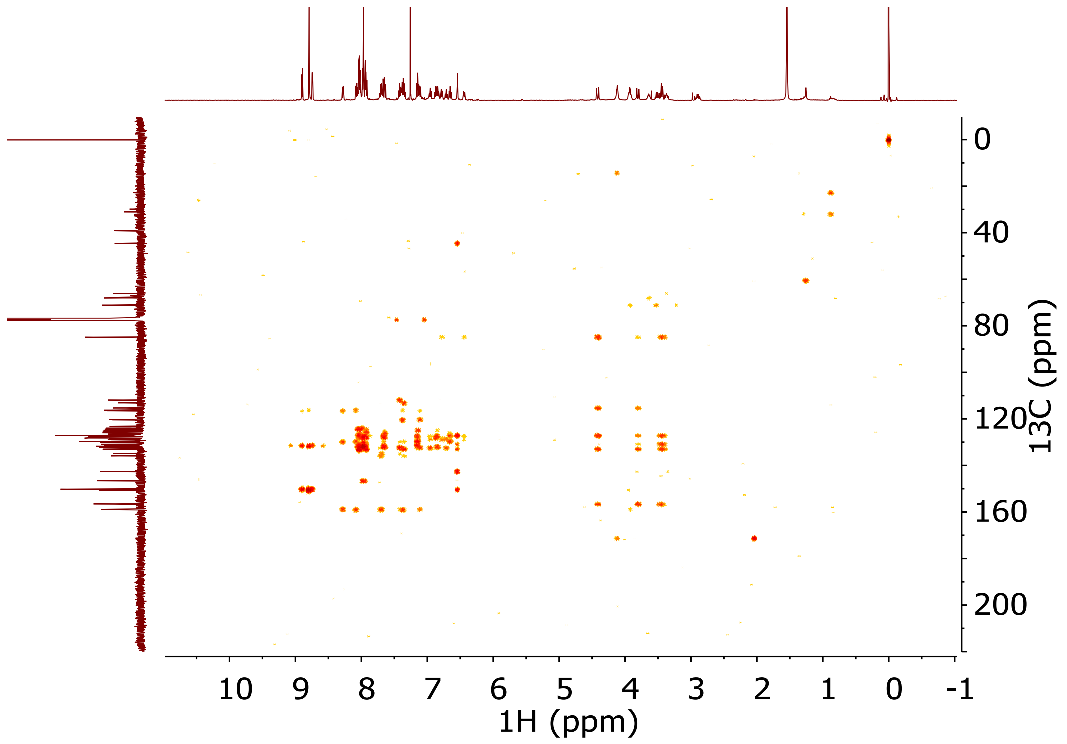

Figure S139. ^{1}H-^{13}C HMBC NMR spectrum (500 MHz, $CDCl_3$) of compound *S*$_p$-(*R,S*)-(*P*)-**Zn6b**

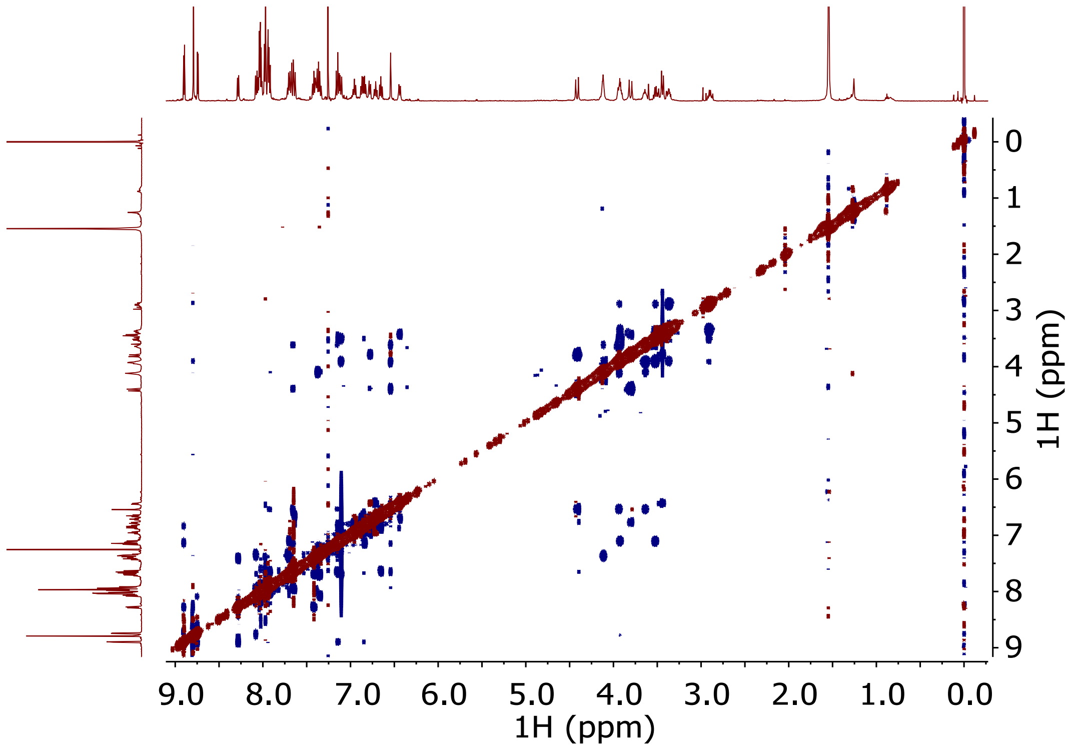

Figure S140. ^{1}H 2D-ROESY NMR spectrum (500 MHz, $CDCl_3$) of compound S_p-(R,S)-(P)-**Zn6b**

4.4. NMR spectra of host-guest complexes

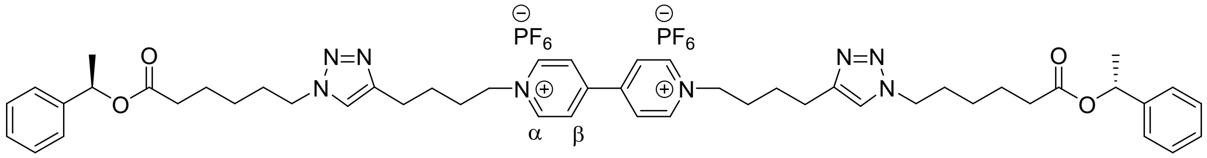

(R,R)-**7**

Table S45. ^{1}H NMR chemical shift values (δ_H) and complexation induced shift values (CIS, $\Delta\delta_H$) for the protons H-α and H-β of guest (R,R)-**7** in the absence (free) and presence (bound) of porphyrin macrocyclic hosts.

	**H-α**	**H-α CIS**	**H-β**	**H-β CIS**
**Host‡**	**δ_H (ppm)**	**$\Delta\delta_H$ (ppm)**	**δ_H (ppm)**	**$\Delta\delta_H$ (ppm)**
**-**	**8.91**	**0**	**8.37**	**0**
**$H_2$1**	6.31	−2.60	4.22	−4.15
(−)-**$H_2$2**	6.59; 6.14	−2.32; −2.77	4.49; 4.13	−3.88; −4.24
(+)-**$H_2$2**	6.60; 6.14	−2.31; −2.77	4.49; 4.12	−3.88; −4.25
(−)-**$H_2$3a**	8.00; 4.39	−0.91; −4.52	5.59; 3.23	−2.78; −5.14
(+)-**$H_2$3a**	8.01; 4.38	−0.90; −4.53	5.58; 3.24	−2.79; −5.13
(−)-**$H_2$3b**	7.77; 4.78	−1.14; −4.13	5.29; 3.32	−3.08; −5.05
(+)-**$H_2$3b**	7.76; 4.80	−1.15; −4.11	5.28; 3.33	−3.09; −5.04
**Zn1**	6.38	−2.53	4.08	−4.29
(−)-**Zn2**	6.48; 6.35	−2.43; −2.56	4.29; 4.22	−4.08; −4.15
(+)-**Zn2**	6.44; 6.38	−2.47; −2.53	4.27; 4.24	−4.10; −4.13
(−)-**Zn4a**	6.77; 6.05	−2.14; −2.86	*	*
(+)-**Zn4a**	6.78; 6.05	−2.13; −2.86	*	*
(−)-**Zn4b**	6.79; 5.97	−2.12; −2.94	*	*
(+)-**Zn4b**	6.80; 5.98	−2.11; −2.93	*	*
(−)-**Zn5a**	6.66; 6.24	−2.25; −2.67	*	*
(+)-**Zn5a**	6.69; 6.23	−2.22; −2.68	*	*
(−)-**Zn5b**	6.64; 6.22	−2.27; −2.69	4.57; 4.32	−3.80; −4.05
(+)-**Zn5b**	6.61; 6.25	−2.30; −2.66	4.54; 4.33	−3.83; −4.04
(−)-**Zn6a**				
(+)-**Zn6a**				
(−)-**Zn6b**				
(+)-**Zn6b**				

‡For all asymmetric hosts, the resulting host-guest complexes contain two sets of non-identical guest proton resonances. *Broad signal of bound guest, resonance could not be assigned unambiguously through 2D NMR experiments.

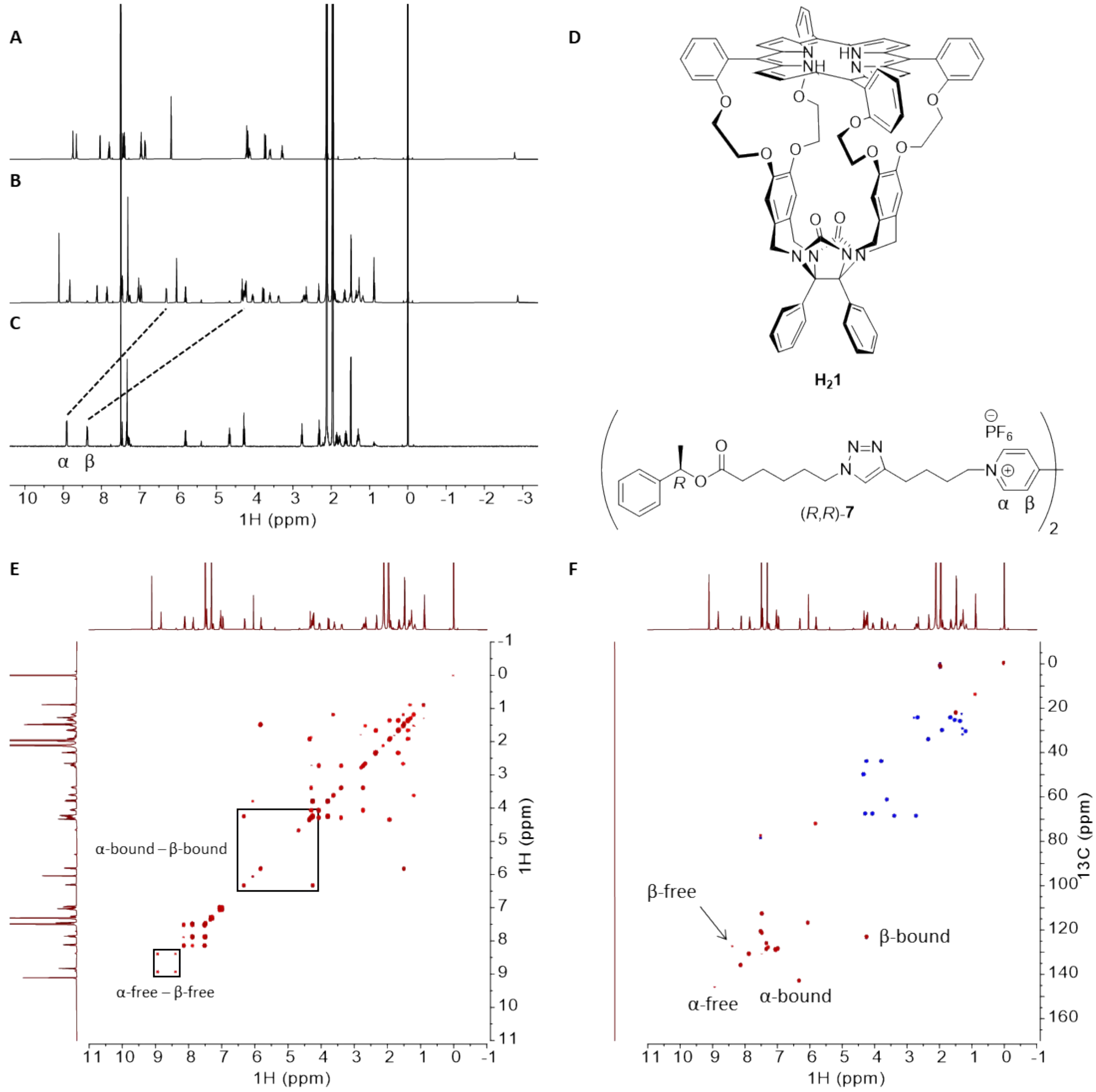

Figure S141. NMR characterization of the host-guest complex **H$_2$1**·(*R,R*)-**7**. ^{1}H NMR spectra (500 MHz, $CDCl_3$/CD_3CN, 1:1, v/v, 298 K) of (A) **H$_2$1**, (B) **H$_2$1**·(*R,R*)-**7**, and (C) (*R,R*)-**7**. (D) Corresponding chemical structures. (E) ^{1}H-^{1}H COSY NMR spectrum of **H$_2$1**·(*R,R*)-**7**. (F) ^{1}H-^{13}C HSQC NMR spectrum of **H$_2$1**·(*R,R*)-**7**.

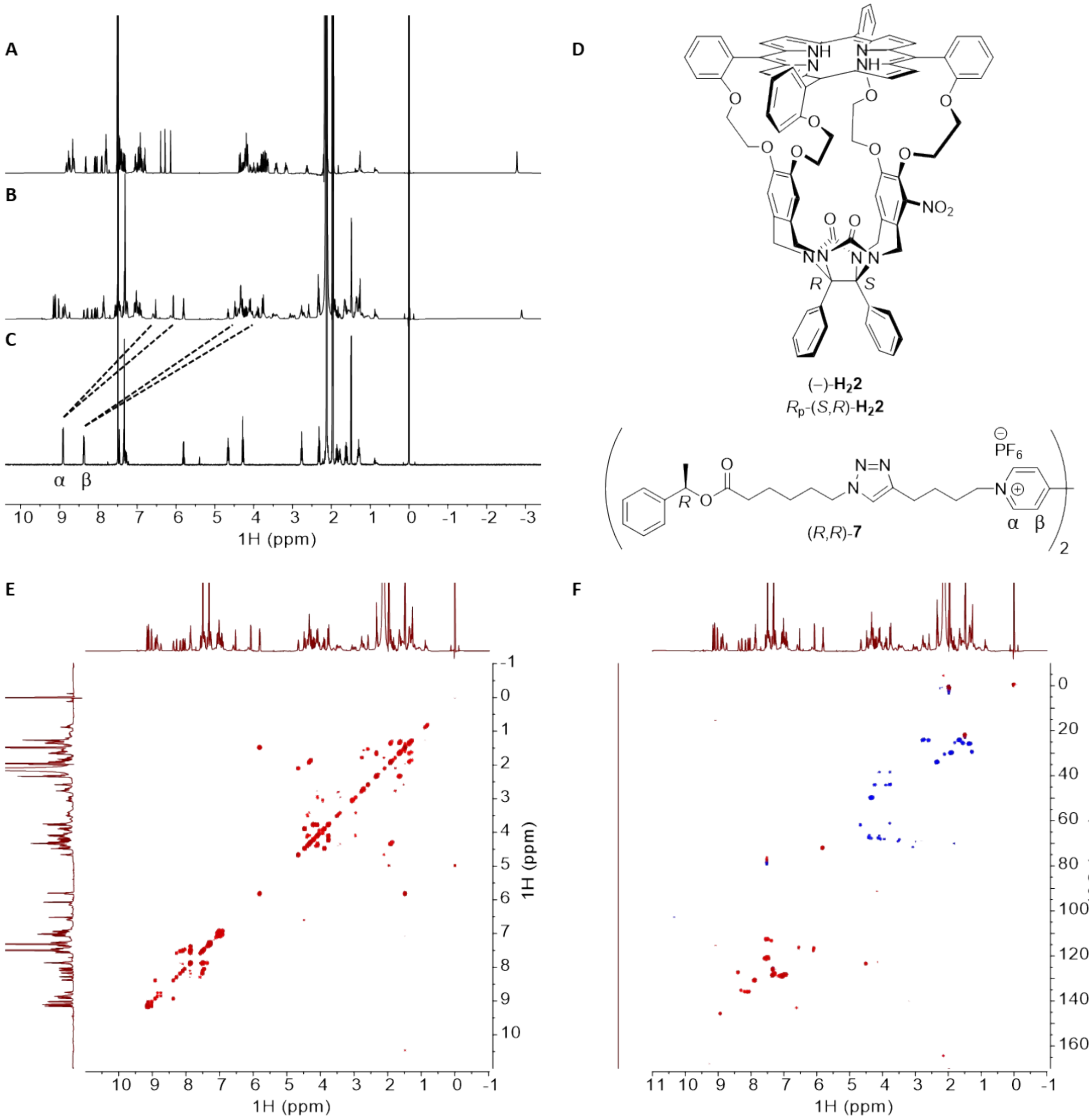

Figure S142. NMR characterization of the host-guest complex (−)-$\mathbf{H_22}$·(*R,R*)-**7**. ^{1}H NMR spectra (500 MHz, $CDCl_3/CD_3CN$, 1:1, v/v, 298 K) of (A) (−)-$\mathbf{H_22}$, (B) (−)-$\mathbf{H_22}$·(*R,R*)-**7**, and (C) (*R,R*)-**7**. (D) Corresponding chemical structures. (E) ^{1}H-^{1}H COSY NMR spectrum of (−)-$\mathbf{H_22}$·(*R,R*)-**7**. (F) ^{1}H-^{13}C HSQC NMR spectrum of (−)-$\mathbf{H_22}$·(*R,R*)-**7**.

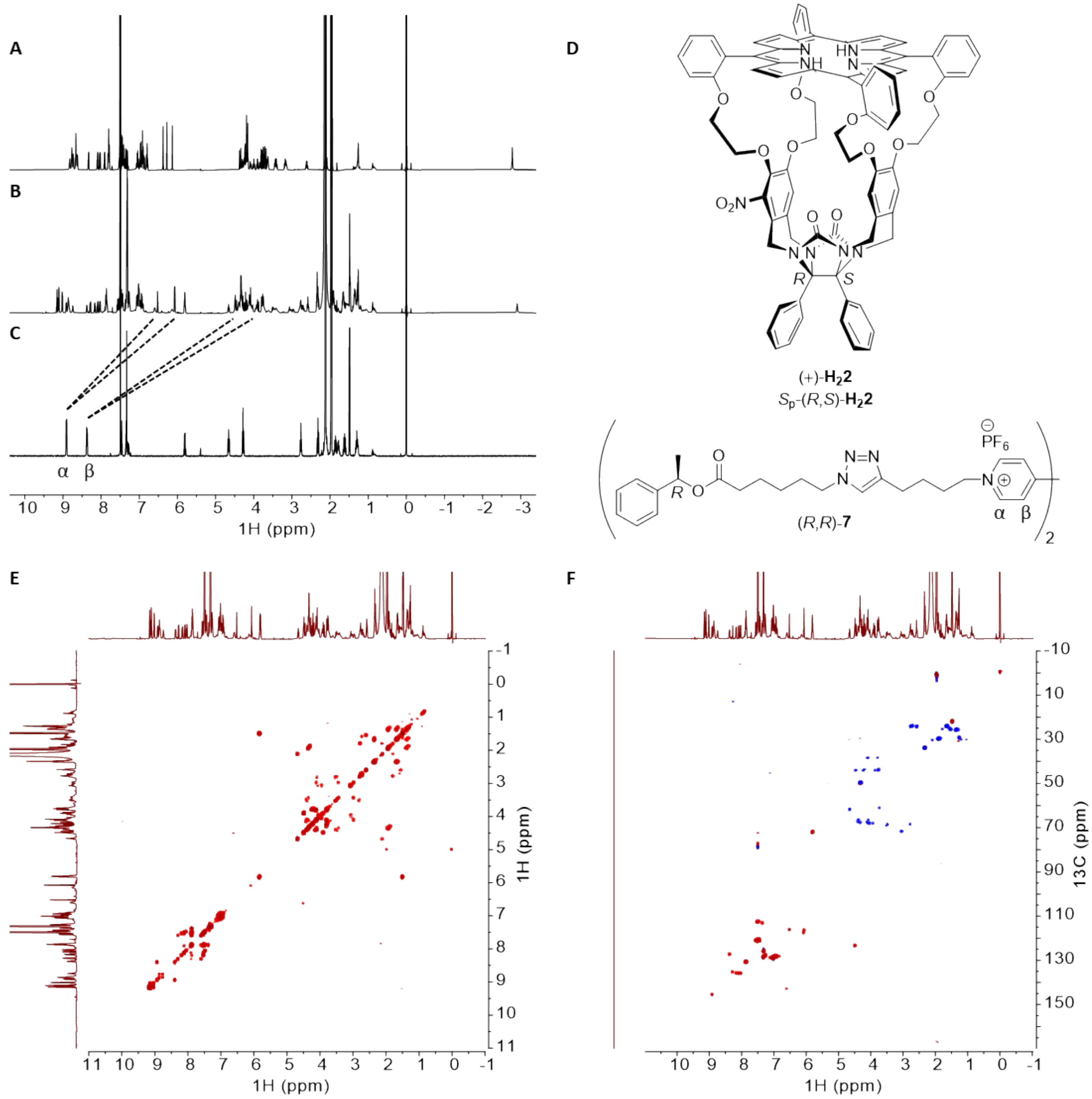

Figure S143. NMR characterization of the host-guest complex (+)-$\mathbf{H_2 2}$·(*R,R*)-**7**. ^{1}H NMR spectra (500 MHz, $CDCl_3/CD_3CN$, 1:1, v/v, 298 K) of (A) (+)-$\mathbf{H_2 2}$, (B) (+)-$\mathbf{H_2 2}$·(*R,R*)-**7**, and (C) (*R,R*)-**7**. (D) Corresponding chemical structures. (E) ^{1}H-^{1}H COSY NMR spectrum of (+)-$\mathbf{H_2 2}$·(*R,R*)-**7**. (F) ^{1}H-^{13}C HSQC NMR spectrum of (+)-$\mathbf{H_2 2}$·(*R,R*)-**7**.

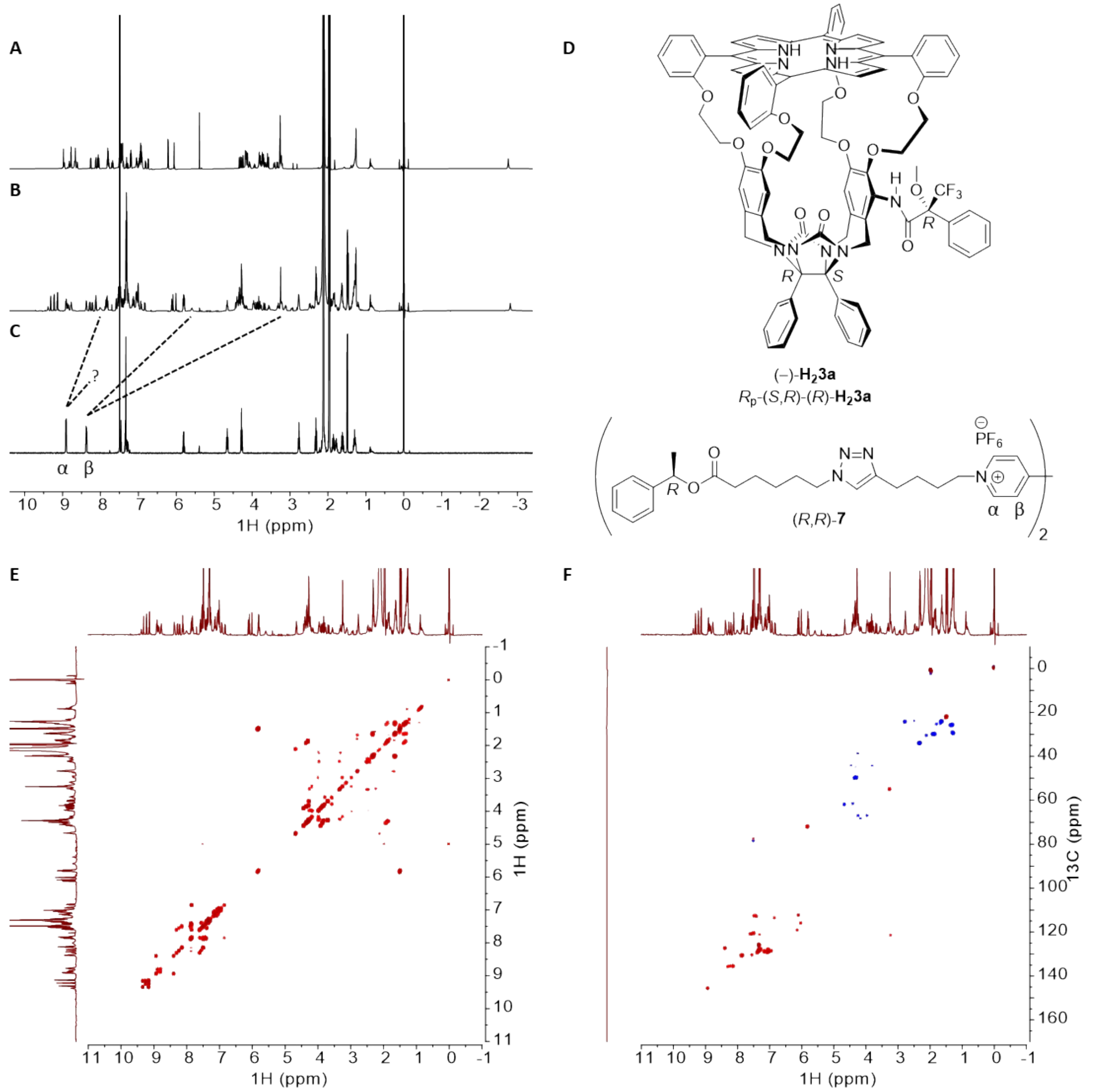

Figure S144. NMR characterization of the host-guest complex (−)-$\mathbf{H_23a}$·(*R,R*)-**7**. ^{1}H NMR spectra (500 MHz, $CDCl_3/CD_3CN$, 1:1, v/v, 298 K) of (A) (−)-$\mathbf{H_23a}$, (B) (−)-$\mathbf{H_23a}$·(*R,R*)-**7**, and (C) (*R,R*)-**7**. (D) Corresponding chemical structures. (E) ^{1}H-^{1}H COSY NMR spectrum of (−)-$\mathbf{H_23a}$·(*R,R*)-**7**. (F) ^{1}H-^{13}C HSQC NMR spectrum of (−)-$\mathbf{H_23a}$·(*R,R*)-**7**.

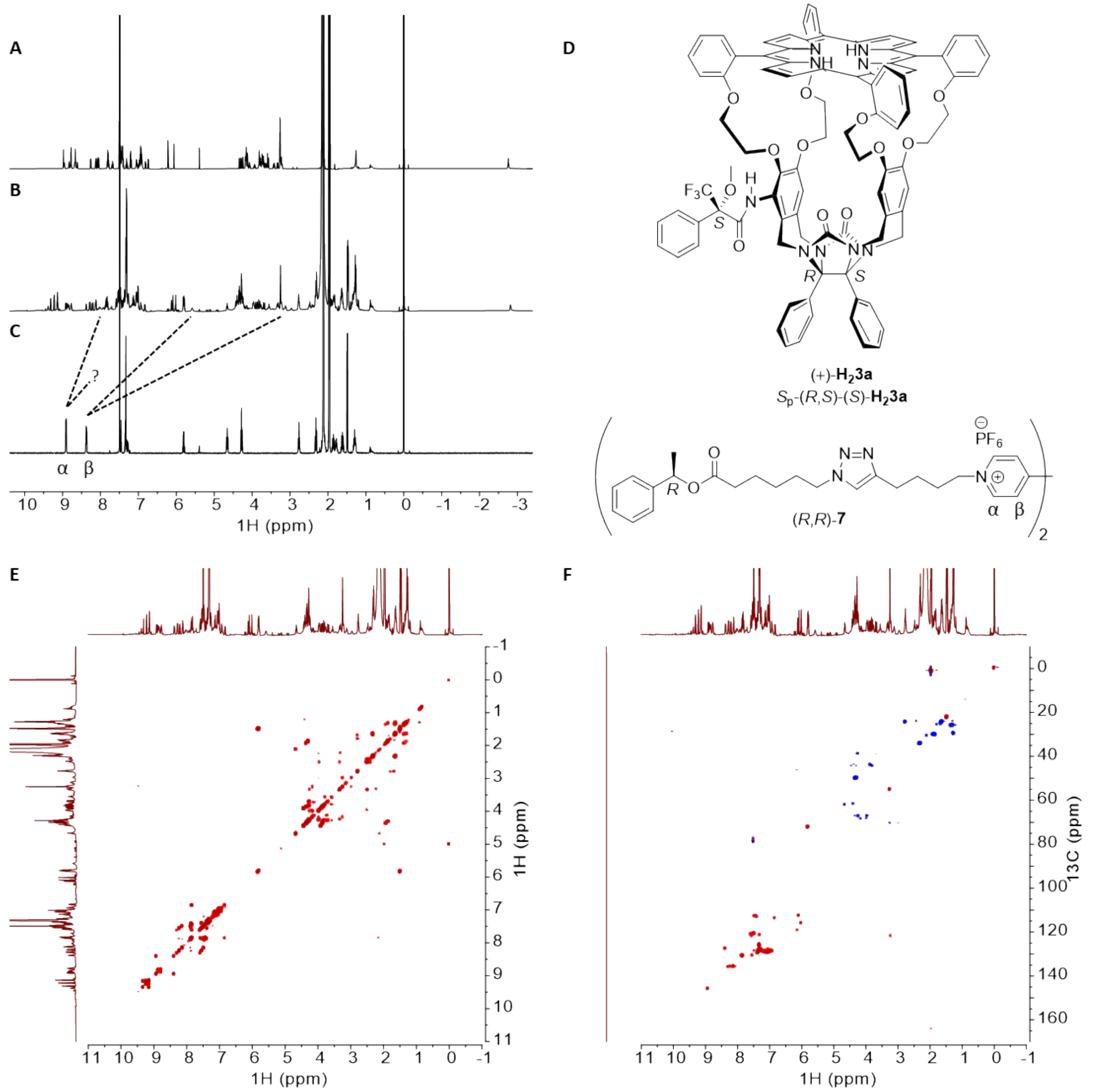

Figure S145. NMR characterization of the host-guest complex (+)-**$H_2$3a·**(*R,R*)-**7**. ^{1}H NMR spectra (500 MHz, $CDCl_3$/CD_3CN, 1:1, v/v, 298 K) of (A) (+)-**$H_2$3a**, (B) (+)-**$H_2$3a·**(*R,R*)-**7**, and (C) (*R,R*)-**7**. (D) Corresponding chemical structures. (E) ^{1}H-^{1}H COSY NMR spectrum of (+)-**$H_2$3a·**(*R,R*)-**7**. (F) ^{1}H-^{13}C HSQC NMR spectrum of (+)-**$H_2$3a**·(*R,R*)-**7**.

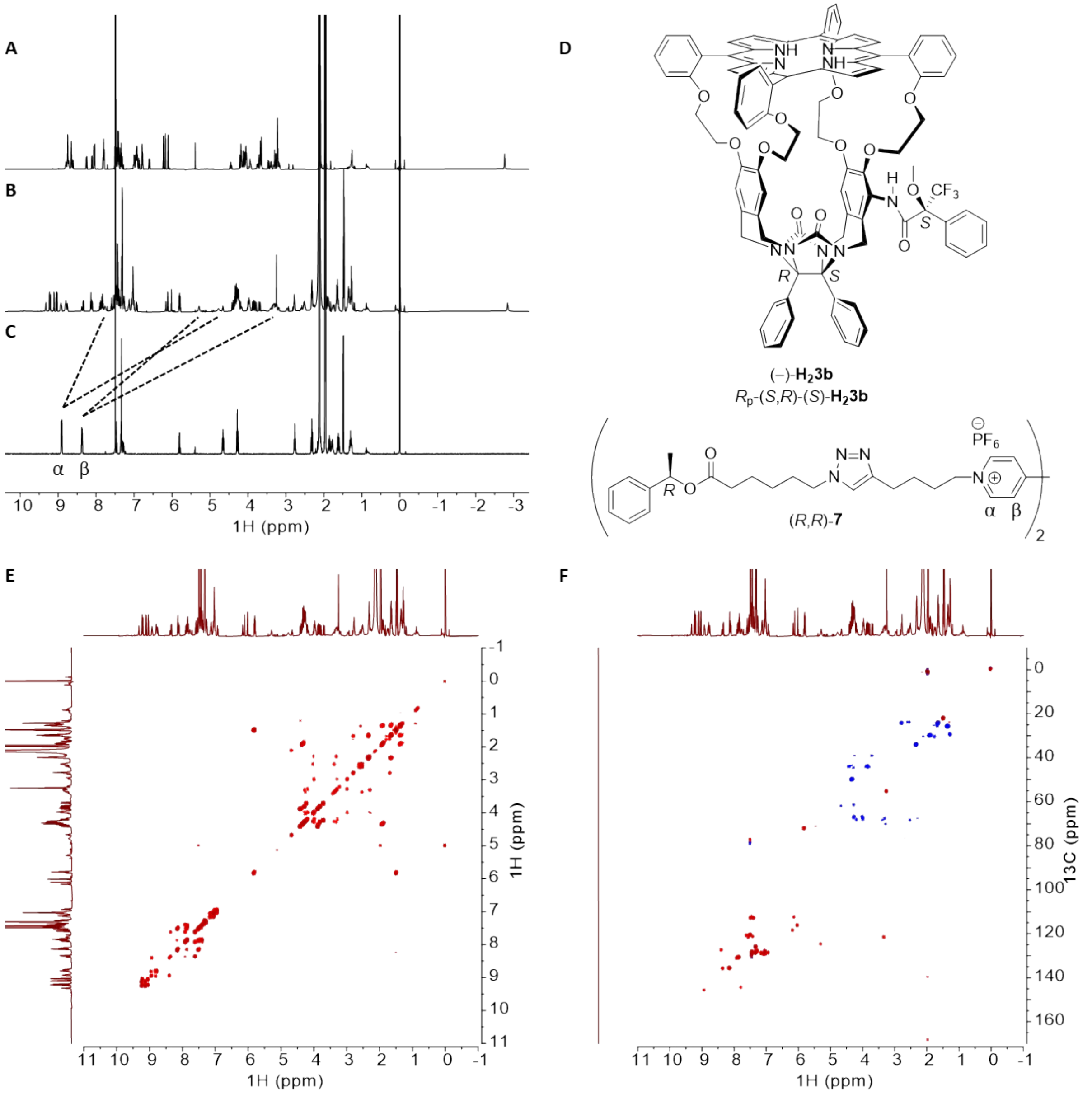

Figure S146. NMR characterization of the host-guest complex (−)-**$H_2$3b·**(*R,R*)-**7**. ^{1}H NMR spectra (500 MHz, $CDCl_3$/CD_3CN, 1:1, v/v, 298 K) of (A) (−)-**$H_2$3b**, (B) (−)-**$H_2$3b·**(*R*,*R*)-**7**, and (C) (*R*,*R*)-**7**. (D) Corresponding chemical structures. (E) ^{1}H-^{1}H COSY NMR spectrum of (−)-**$H_2$3b·**(*R*,*R*)-**7**. (F) ^{1}H-^{13}C HSQC NMR spectrum of (−)-**$H_2$3b·**(*R*,*R*)-**7**.

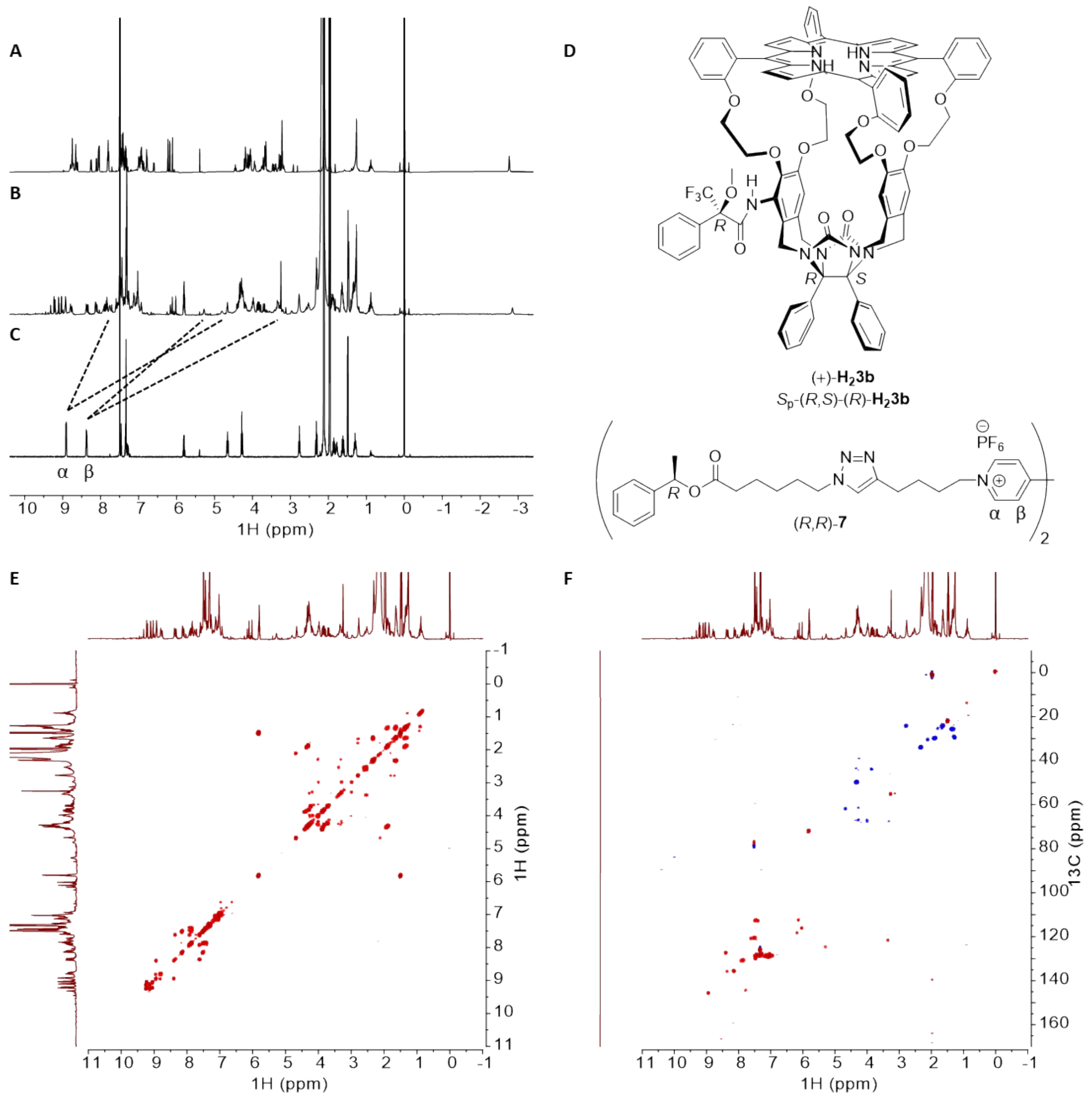

Figure S147. NMR characterization of the host-guest complex (+)-$\mathbf{H_23b}$·(R,R)-**7**. ^{1}H NMR spectra (500 MHz, $CDCl_3/CD_3CN$, 1:1, v/v, 298 K) of (A) (+)-$\mathbf{H_23b}$, (B) (+)-$\mathbf{H_23b}$·(*R,R*)-**7**, and (C) (*R,R*)-**7**. (D) Corresponding chemical structures. (E) ^{1}H-^{1}H COSY NMR spectrum of (+)-$\mathbf{H_23b}$·(*R,R*)-**7**. (F) ^{1}H-^{13}C HSQC NMR spectrum of (+)-$\mathbf{H_23b}$·(*R,R*)-**7**.

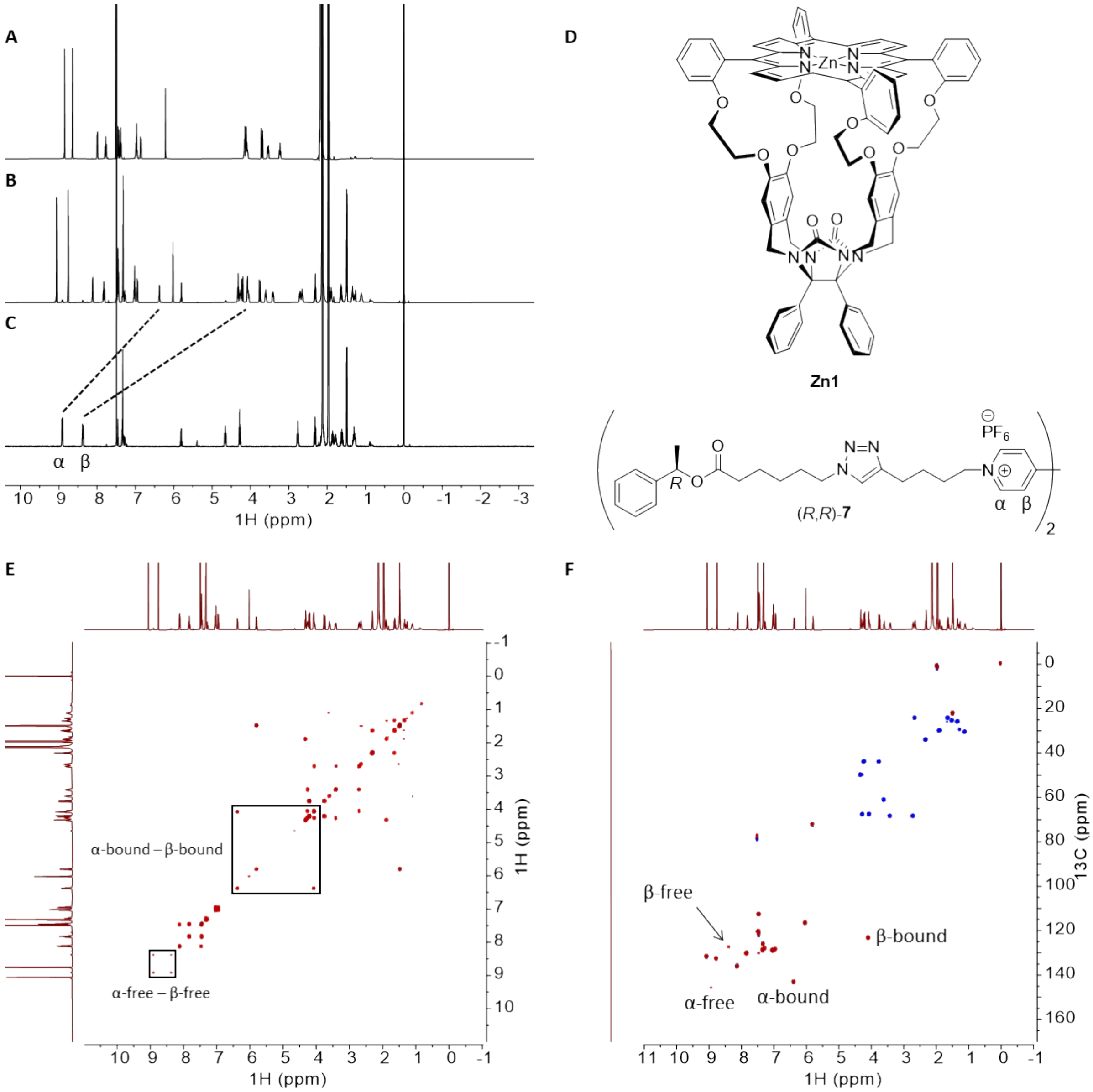

Figure S148. NMR characterization of the host-guest complex **Zn1**·(*R,R*)-**7**. ^{1}H NMR spectra (500 MHz, $CDCl_3$/CD_3CN, 1:1, v/v, 298 K) of (A) **Zn1**, (B) **Zn1**·(*R,R*)-**7**, and (C) (*R,R*)-**7**. (D) Corresponding chemical structures. (E) ^{1}H-^{1}H COSY NMR spectrum of **Zn1·**(*R,R*)-**7**. (F) ^{1}H-^{13}C HSQC NMR spectrum of **Zn1**·(*R,R*)-**7**.

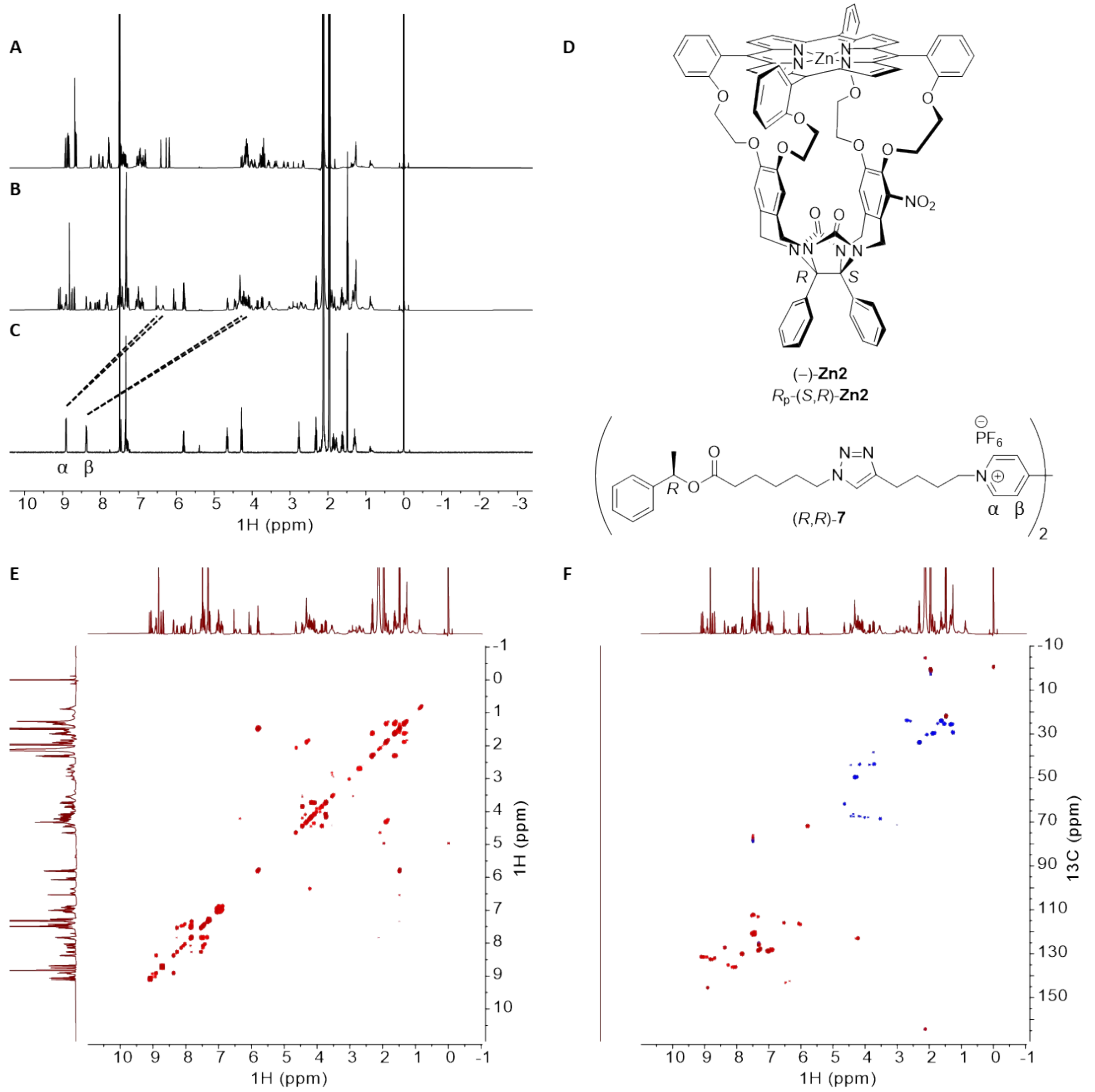

Figure S149. NMR characterization of the host-guest complex (−)-**Zn2**·(*R,R*)-**7**. ^{1}H NMR spectra (500 MHz, $CDCl_3/CD_3CN$, 1:1, v/v, 298 K) of (A) (−)-**Zn2**, (B) (−)-**Zn2**·(R,R)-**7**, and (C) (*R,R*)-**7**. (D) Corresponding chemical structures. (E) ^{1}H-^{1}H COSY NMR spectrum of (−)-**Zn2**·(*R,R*)-**7**. (F) ^{1}H-^{13}C HSQC NMR spectrum of (−)-**Zn2**·(*R,R*)-**7**.

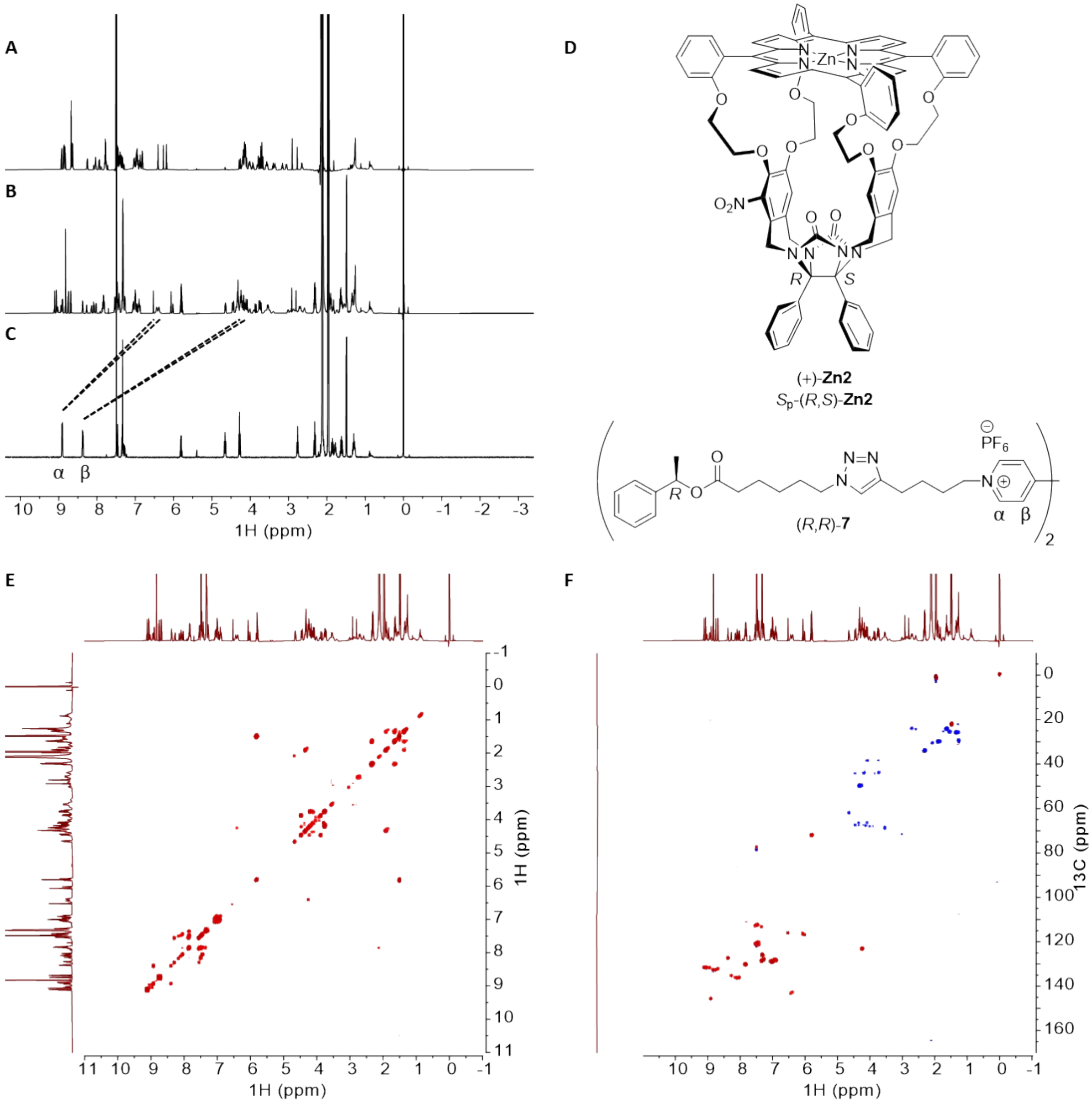

Figure S150. NMR characterization of the host-guest complex (+)-**Zn2·**(*R,R*)-**7**. ^{1}H NMR spectra (500 MHz, $CDCl_3/CD_3CN$, 1:1, v/v, 298 K) of (A) (+)-**Zn2**, (B) (+)-**Zn2·**(*R,R*)-**7**, and (C) (*R,R*)-**7**. (D) Corresponding chemical structures. (E) ^{1}H-^{1}H COSY NMR spectrum of (+)-**Zn2·**(*R,R*)-**7**. (F) ^{1}H-^{13}C HSQC NMR spectrum of (+)-**Zn2·**(*R,R*)-**7**.

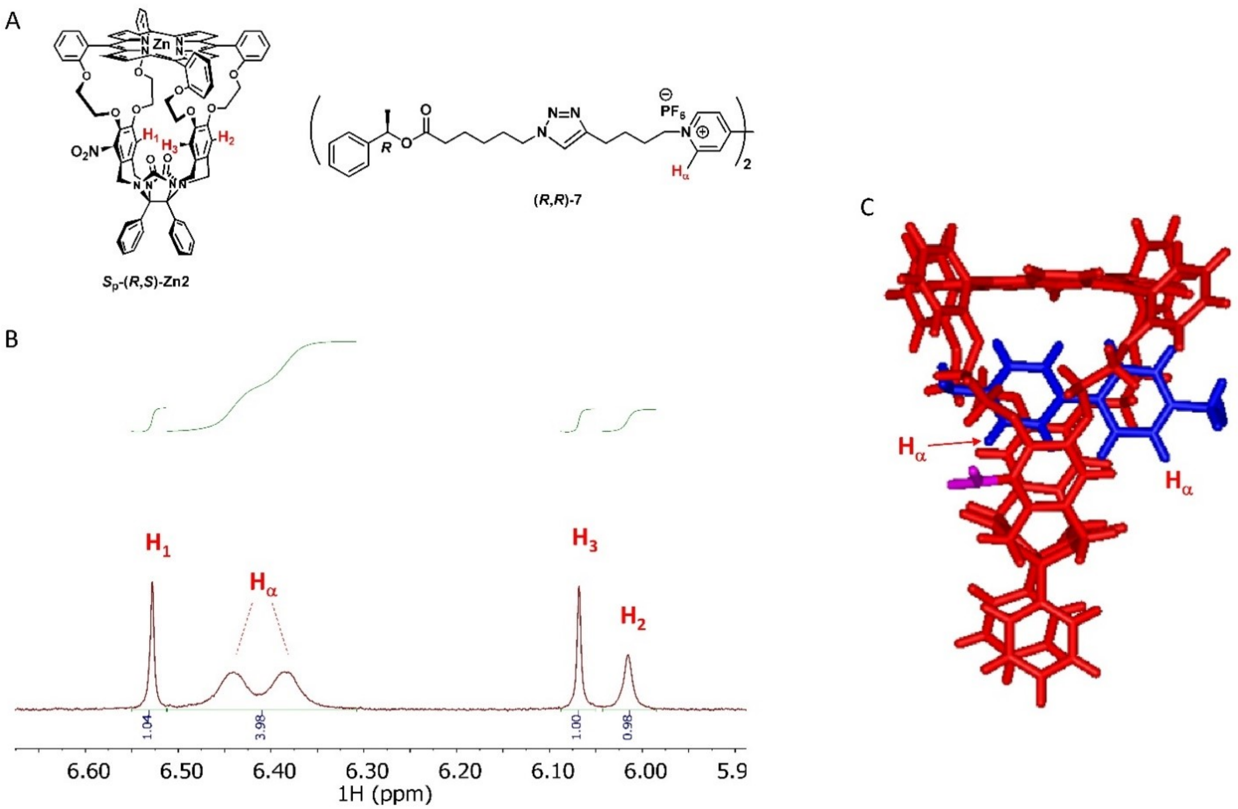

Figure S151. General binding model for the complexes between the various porphyrin cages and guest (*R*,*R*)-**7**. As an illustration, the properties of the complex between host S_p-(*R*,*S*)-**Zn2** and guest (*R*,*R*)-**7** are depicted. The complexes between the guest and all other hosts display very similar NMR properties (in all cases 1:1 complexes are formed, with the shifts in the NMR spectra varying somewhat). (A) Structure of host S_p-(*R*,*S*)-**Zn2** and guest (*R*,*R*)-**7** with the numbering of the relevant protons. (B) Part of the ^{1}H NMR spectrum (500 MHz, $CDCl_3/CD_3CN$, 1:1, v/v, 298 K) of the 1:1 complex between S_p-(*R*,*S*)-**Zn2** and (*R*,*R*)-**7** showing the signals relevant for the determination of the host-guest stoichiometry. Note that two signals are found for the α-protons of the complexed viologen, indicating an unsymmetrical binding geometry of this moiety in the cavity of the host. (C) Computer-modelled structure of the host-guest complex between S_p-(*R*,*S*)-**Zn2** (red, nitro group magenta) and the central viologen moiety of (*R*,*R*)-**7** (blue). The viologen N-substituents are omitted for clarity. The computer-modelled structure is based on 1D and 2D NMR experiments and highlights the unsymmetrical binding geometry of the viologen in the cavity of the host.

5. References

S1 K. Watanabe, T. Mino, C. Hatta, S. Ito and M. Sakamoto, *Org. Biomol. Chem.*, 2015, **13**, 11645-11650.

S2 Patent: ALMIRALL SA - WO2013/10880, 2013, A1.

S3 M.K.J. Ter Wiel, M.G. Kwit, A. Meetsma and B.L. Feringa, *Org. Biomol. Chem.*, 2007, **5,** 87-96.

S4 D.G. Botteron and G.P. Shulman, *J. Org. Chem.*, 1962, **27**, 1059–1061.

S5 X. Du, F. Xu, M.-S. Yuan, P. Xue, L. Zhao, D.-E. Wang, W. Wang, Q. Tu, S.-W. Chen and J. Wang, *J. Mater. Chem.*, 2016, ***C*4**, 8724–8730.

S6 P.J. Gilissen, P.B. White, J.A. Berrocal, N. Vanthuyne, F.P.J.T. Rutjes, B.L. Feringa, J.A.A.W. Elemans and R.J.M. Nolte, *Nat. Commun.*, 2020, **11**, 5291.

S7 E. Anger, M. Srebro, N. Vanthuyne, L. Toupet, S. Rigaut, C. Roussel, J. Autschbach, J. Crassous and R. Réau, *J. Am. Chem. Soc.*, 2012, **134**, 15628-15631.

S8 Y. Pu, M.E. Ridgeway, R.S. Glaskin, M.A. Park, C.E. Costello and C. Cheng Lin, *Anal. Chem.*, 2016, **88**, 3440-3443.

S9 F.C. Liu, S.R. Kirka and C. Bleiholder, *Analyst*, 2016, **141,** 3722-3730.

S10 V. Gabelica and E. Marklund, “Fundamentals of ion mobility spectrometry”, *Curr. Opin. Chem. Biol.*, 2018, **42**, 51-59.
